# Supplementary material for: NHC-Ni catalyzed enantioselective synthesis of 1,4-dienes by cross-hydroalkenylation of cyclic 1,3-dienes and heterosubstituted terminal olefins
Source: Nat Commun. 2020 May 8;11:2269. doi: 10.1038/s41467-020-16139-2 (PMC7210895; doi:10.1038/s41467-020-16139-2)
Supplement: Supplementary file 1 — Supplementary Information [file 41467_2020_16139_MOESM1_ESM.pdf]

Supplementary Information

NHC-Ni Catalyzed Enantioselective Synthesis of 1,4-dienes by Cross-Hydroalkenylation of Cyclic 1,3-dienes and Heterosubstituted Terminal Olefins

Chen et al.

Supplementary Methods

General Aspects. Unless otherwise indicated, all reactions were performed under a nitrogen atmosphere from which oxygen and moisture were rigidly excluded from reagents and glassware. Ni(cod)$_2$ [Bis(cyclooctadienyl)nickel(0)], KHMDS [Potassium bis(trimethylsilyl)amide] were purchased from ACROS or Aldrich, stored in glove box and used without further purification. NaBAr^F [Sodium tetrakis[3,5-bis(trifluoromethyl)phenyl]borate] was purchased from Ark, vacuumized for 12 hrs. under 110 °C and then stored in the glove box before use. Allyl chloride and 1-octene were purchased from TCI, filtered through a short plug of silica gel and dried with calcium hydride before use. Toluene was distilled over sodium and CaH_2 before use. (1R, 2R)-(+)-1,2-diphenylethylenediamine were purchased from accela, stored in the glove box and used without further purification. Chiral NHCs were obtained from the corresponding salts by deprotonation with KHMDS in the glove box at r.t. for 1h before use.

Both commercially available and synthesized cyclo-1,3-dienes, (homo)allyl ethers and allylamines were dried with CaH_2 before use. All the ethers or amines are known compounds and were synthesized according to literature.[1-8]

Analytical thin layer chromatography (TLC) was performed using EM Science silica gel 60 F254 plates. The developed chromatogram was analyzed by UV lamp (254 nm), ethanolic phosphomolybdic acid (PMA) or potassium permanganate ($KMnO_4$). Purification of the product was performed by using Silica Gel (230–400 mesh, 0.040-0.063 mm) coarse fritted glass column. Cyclo-1,3-dienes and cross-hydroalkenylation products can be isolated by column chromatography on buffered silica gel.

^{1}H and ^{13}C NMR spectra were recorded on Bruker spectrometers in $CDCl_3$ or C_6D_6 (400 or 500 MHz for ^{1}H and 100 or 125 MHz for ^{13}C). Chemical shifts in ^{1}H NMR spectra are reported in ppm on the δ scale from an internal standard of TMS. Data are reported as follows: chemical shift, multiplicity (s = singlet, d = doublet, t = triplet, q = quartet, m = multiplet, br = broad), coupling constant in hertz(Hz), and integration. Chemical shifts of ^{13}C NMR spectra are reported in ppm from the central peak of $CDCl_3$ (77.16 ppm) or C_6D_6 (128.06 ppm) on the δ scale. Yield and selectivity were determined by the integration of areas of selected peaks in crude ^{1}H NMR with relaxation time d1 = 10 seconds and nitromethane (CH_3NO_2) as standard. Enantiomer excess (ee) of chiral products were determined by high performance liquid chromatography (HPLC) using a Daicel CHIRALCEL OD-H (inner diameter: 4.6 mm, length: 250 mm, particle size: 5 μm, cellulose tris (3,5-dimethylphenylcarbamate) coated on silica

support) column or AS-3 (inner diameter: 4.6 mm, length: 250 mm, particle size: 3 μm, Amylose tris-[(S)-α-methylbenzylcarbamate] coated on silica support) column with hexane/isopropanol as solvents on Agilent Technologies 1260 Infinity instrument. HPLC Conditions were described under specific compounds. Optical rotations were recorded at the sodium D line in chloroform on a Rudolph Autopol I Polarimeter. High resolution mass spectra (HRMS) were obtained on a Finnigan MAT 95XL GC Mass Spectrometer of Southern University of Science and Technology, China.

General Procedure for Preparation of [Chiral NHC-Ni(allyl)Cl].[9-15] Unlike the enantioselective cross-hydroalkenylation of the styrene with α-olefin, the catalysts were generated from chiral NHC and $[Ni(allyl)Cl]_2$. All the chiral NHC salts were prepared according to literature. [9-15]

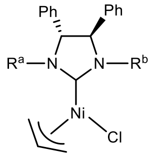

[**L**-Ni(allyl)Cl]

Supplementary Table 1 NHC structures employed in this work

	R^a	R^b
**L1**	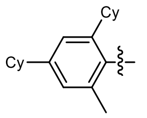	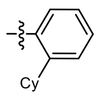
**L2**	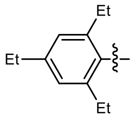	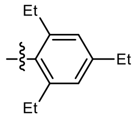
**L3**	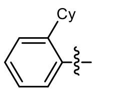	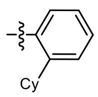

Synthesis of chiral NHC salt. All these three chiral NHC salts were synthesized by following the literature, except (1R, 2R)-(+)-1,2-diphenylethylenediamine was used. [11]

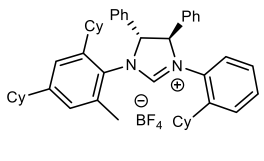

^{1}H NMR (500 MHz, $CDCl_3$) δ 8.51 (s, 0.32H), 8.32 (s, 0.68H), 7.87 – 7.81 (d, J = 8.0 Hz, 0.68H), 7.64 – 7.60 (d, J = 8.0 Hz, 0.32H), 7.58 – 7.27 (m, 13H), 7.06 – 7.00 (d, J = 19.3 Hz, 1H), 6.80 – 6.73 (d, J = 21.8, 1H), 6.10 – 5.97 (dd, J = 34.9, 9.0 Hz, 1H), 5.76 – 5.71 (d, J = 9.3 Hz, 0.68H), 5.51 – 5.46 (d, J = 9.3 Hz, 0.32H), 2.92 (m, 1H), 2.73 (m, 2.32H), 2.40 (m, 1H), 2.23 (m, 0.68H), 2.17 – 1.10 (m, 31H).^{13}C NMR (125 MHz, $CDCl_3$) δ: 157.9,

157.3, 150.7, 150.6, 145.4, 144.6, 143.3, 143.1, 136.0, 135.0, 134.8, 134.4, 132.6, 132.4, 131.2, 131.0, 130.9, 130.7, 130.6, 130.5, 130.0, 139.7, 129.5, 129.1, 129.0, 128.4, 128.3, 128.2, 128.1, 128.0, 127.9, 127.6, 127.1, 124.6, 124.2, 77.6, 75.4, 75.144.4, 40.1, 39.8, 39.5, 36.2, 35.1, 35.0, 34.4, 34.3, 34.2, 33.1, 27.5, 27.4, 27.2, 27.0, 26.9, 26.2, 26.1, 26.0, 25.9, 19.5, 18.8.HRMS-ESI (m/z): $[M-BF_4]^+$ calcd for $C_{46}H_{55}N_2$ 635.4360; found 635.4363. Optical Rotation: $[\alpha]_D^{24.8}$ = +295° (c = 0.6, $CHCl_3$).

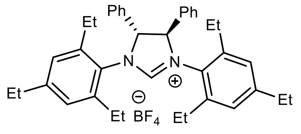

^{1}H NMR (500 MHz, $CDCl_3$) δ 8.57 (s, 1H), 7.46 – 7.31 (m, 10H), 7.08 (s, 2H), 6.83 (s, 2H), 5.97 (s, 2H), 3.06 – 2.90 (m, 4H), 2.64 – 2.57 (m, 4H), 2.50 – 2.41 (m, 2H), 2.08 – 1.99 (m, 2H), 1.60 – 1.53 (m, 6H), 1.21 (t, J = 7.6 Hz, 6H), 0.86 (t, J = 7.6 Hz, 6H). ^{13}C NMR (125 MHz, $CDCl_3$) δ 158.7, 147.2, 142.3, 140.4, 131.1, 131.1, 130.9, 129.6, 129.1, 127.4, 127.0, 126.7, 28.7, 24.9, 24.0, 15.6, 15.1, 14.6. HRMS-ESI (m/z): $[M-BF_4]^+$ calcd for $C_{39}H_{47}N_2$ 543.3734; found 543.3729. Optical Rotation: $[\alpha]_D^{25.8}$ = +155° (c = 0.6, $CHCl_3$).

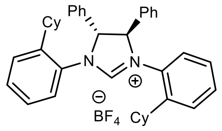

Analytical data were consistent with those reported in the literature.[15]

Preparation of chiral NHC in toluene solution. In a glove box, chiral NHC*HBF$_4$ (0.05 mmol) and KHMDS (0.065 mmol) were suspended in 1 mL toluene and was stirred for 1 hr.

Preparation of [Ni(allyl)Cl]$_2$ in toluene solution. [9,10,13] Ni(cod)$_2$ (0.05 mmol) was added to another oven-dried test tube. Six drops cod followed by two drops of allyl chloride were added, and the mixture was stirred until Ni(cod)$_2$ was dissolved completely (~10 mins). Then the excess allyl chloride and cod were then removed by vacuum.

Preparation of [chiral NHC-Ni(allyl)Cl]. [9,10,13] The chiral NHC solution from the above was then filtered to the [Ni(allyl)Cl]$_2$ solution. The residue was further rinsed by 3*0.2 ml toluene, the mixture was stirred for another 1 hr at r.t. to obtain [chiral NHC-Ni(allyl)Cl].

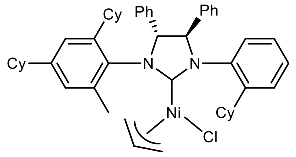

Exist as a mixture of rotamers. See NMR spectra for detail.

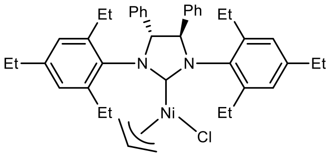

42% yield over 2 steps.

^{1}H NMR (500 MHz, C_6D_6) δ 7.48 – 6.81 (m, 14H), 5.85 – 5.57 (m, 2H), 4.67 – 4.28 (m, 1H), 4.07 – 3.81 (m, 2H), 3.30 – 2.94 (m, 4H), 2.87 – 2.78 (m, 2H), 2.68 – 2.55 (m, 2H), 2.49 – 2.36 (m, 5H), 1.53 – 1.45 (m, 3H), 1.25 (t, *J* = 7.5 Hz, 3H), 1.14 (t, *J* = 7.5 Hz, 3H), 1.08-0.98 (m, 9H), 0.93 – 0.91 (m, 1H).

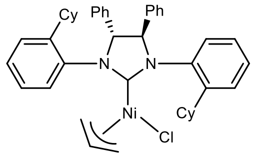

95% yield over 2 steps.

^{1}H NMR (400 MHz, C_6D_6) δ 7.82 – 7.58 (m, 2H), 7.39 – 6.76 (m, 16H), 5.27 – 5.05 (m, 2H), 4.81 – 4.45 (m, 1H), 3.39 – 3.30 (m, 1H), 3.23-2.85 (m, 2H), 2.60 – 2.37 (m, 2H), 1.99 – 0.80 (m, 21H).

General procedure for endocyclic diene synthesis. Most of the dienes employed in this work were synthesized in 1 step by using the corresponding enones.

Unsaturated cyclic enones synthesis. The following showed the synthetic methods of those enones, in which they were synthesized by the literature procedure except otherwise indicated.[16-33]

Supplementary Table 2 Summary of enone synthetic methods in this work

i) commercially available enone

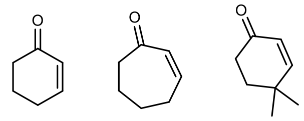

ii) From cyclohex-2-en-1-one[16-19]

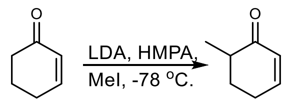

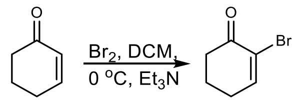

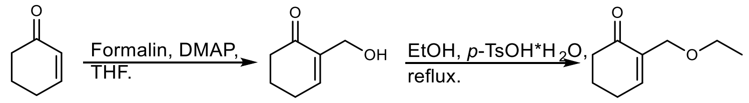

iii) From cyclohexanone[20-23]

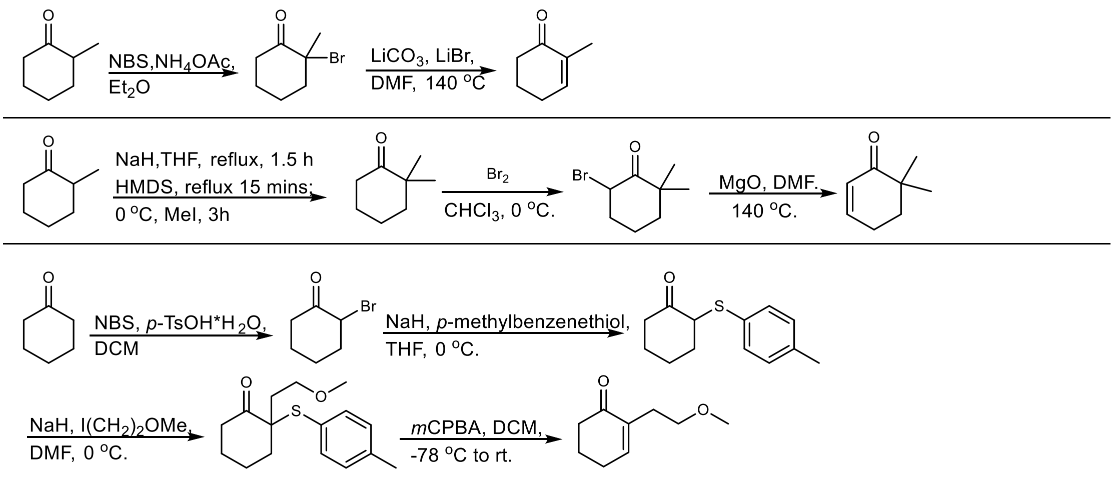

iv) From cyclo-1,3-dione[24-28]

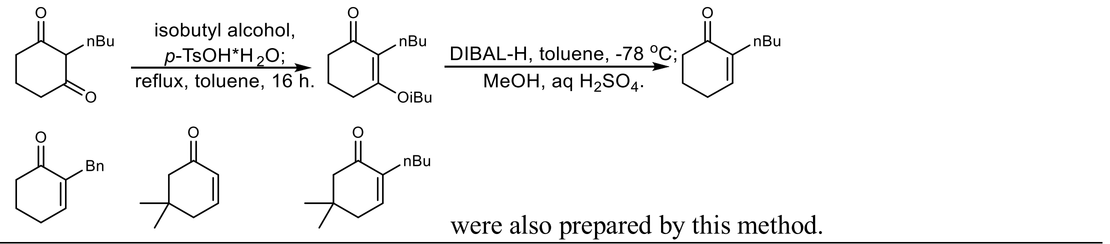

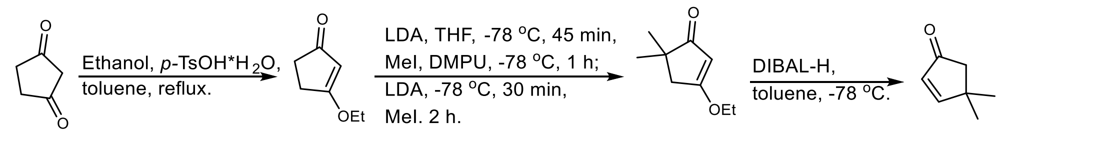

v) From 1,4-dioxaspiro[4.5]decan-8-one[29,30]

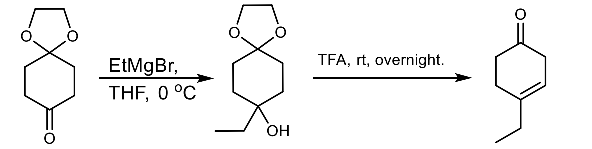

vi) For 2-D-cyclohex-2-en-1-one[31-33]

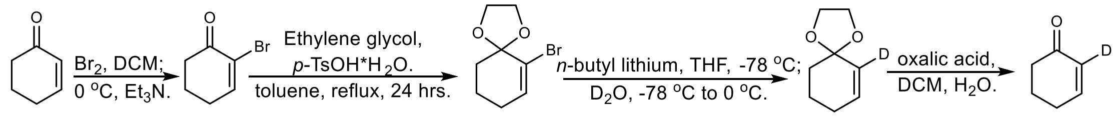

Characterization data of new unsaturated cyclic enones

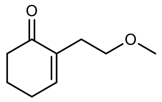

overall yield: 34%.[23]

^{1}H NMR (500 MHz, $CDCl_3$) δ 6.82 (t, *J* = 4.1 Hz, 1H), 3.45 (t, *J* = 6.5 Hz, 2H), 3.32 (s, 3H), 2.52 – 2.41 (m, 4H), 2.37 (m, 2H), 1.99 (m, 2H). ^{13}C NMR (125 MHz, $CDCl_3$) δ 199.4, 147.0, 136.6, 71.2, 58.5, 38.4, 30.0, 26.1, 23.1. HRMS-ESI (m/z): [M+H]+ calcd for $C_9H_{15}O_2$ 155.1072; found 155.1063.

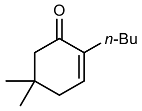

overall yield: 72%.[24,25,27]

^{1}H NMR (500 MHz, $CDCl_3$) δ 6.55 (t, *J* = 4.2 Hz, 1H), 2.27 (s, 2H), 2.23 (m, 2H), 2.18 (m, 2H), 1.37 (m, 2H), 1.33 – 1.28 (m, 2H), 1.02 (s, 6H), 0.92 – 0.88 (t, *J* = 7.0 Hz, 3H). ^{13}C NMR (125 MHz, $CDCl_3$) δ 199.7, 142.5, 138.9, 52.1, 40.2, 34.0, 30.8, 28.9, 28.3, 22.5, 14.0. HRMS-ESI (m/z): $[M+H]^+$ calcd for $C_{12}H_{21}O$ 181.1514; found 181.1583.

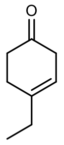

overall yield: 31%.[29,30]

^{1}H NMR (500 MHz, CDCl3) δ 5.44 (m, 1H), 2.85 (m, 2H), 2.50 (t, *J* = 7.5 Hz, 2H), 2.44 – 2.38 (t, *J* = 7.5 Hz, 2H), 2.12 – 2.04 (m, 2H), 1.04 (t, *J* = 7.5 Hz, 3H). ^{13}C NMR (125 MHz, CDCl3) δ 211.2, 140.2, 116.4, 39.6, 38.7, 29.8, 28.6, 12.2. HRMS-ESI (m/z): [M+H]+ calcd for $C_8H_{13}O$ 125.0966; found 125.0960.

Dienes synthesis and characterization data. Besides the commercially available dienes, most of the dienes employed in this work were synthesized by typical silyl enol ether synthesis procedures.[34-37] Others are synthesized accordingly.[38-40] Methods are summarized in the following table:

Supplementary Table 3 Synthetic Methods of cyclic 1,3-dienes

	Dienes
Commercially available	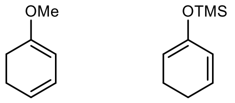
Method A[34]	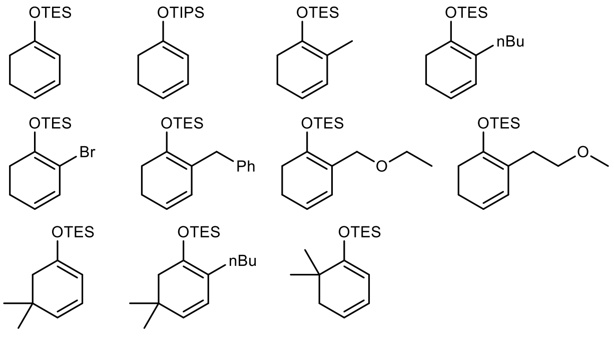
Method B[35,36]	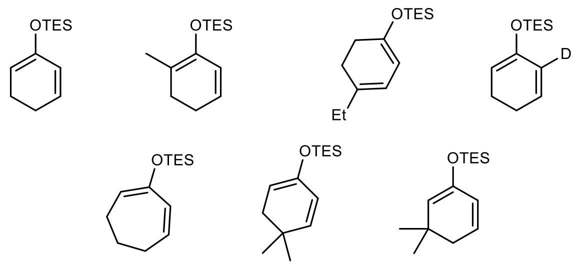
Method C[38]	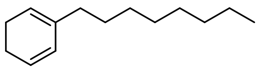
Method D[37]	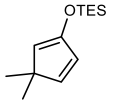
Method E[39,40]	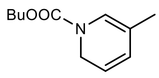

Method A[34]

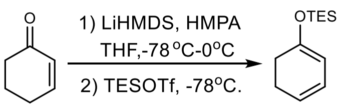

Supplementary Figure 1 Synthesis of 1-OTES-cyclohexadiene

Literature procedure was followed,[34] except TESOTf or TIPSOTf was used and products were purified

by flash column chromatography on silica gel (buffered with 3% NEt_3/n-hexane, 3% NEt_3/n-hexane as eluent).

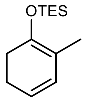

Yield: 62%.

^{1}H NMR (400 MHz, $CDCl_3$) δ 5.74 – 5.67 (d, J = 9.6 Hz, 1H), 5.46 – 5.36 (m, 1H), 2.23 – 2.15 (m, 4H), 1.64 (s, 3H), 0.97 (t, J = 8.0 Hz, 10H), 0.66 (q, J = 7.9 Hz, 6H). ^{13}C NMR (100 MHz, $CDCl_3$) δ 146.4, 129.9, 118.8, 110.3, 29.0, 24.6, 14.3, 6.9, 5.7. HRMS-ESI (m/z): $[M+H]^+$ calcd for $C_{13}H_{25}OSi$ 225.1675; found 225.1668.

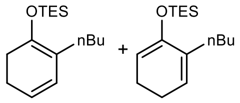

Yield: 85%, 88: 12.

^{1}H NMR (500 MHz, $CDCl_3$) δ:5.79 (d, J = 9.4 Hz, 1H), 5.45 (m, 1H), 2.23 (m, 4H), 2.08 (t, J = 7.3 Hz, 2H), 1.37 – 1.24 (m, 4H), 1.00 (t, J = 7.9Hz, 9H), 0.90 (t, J = 6.9 Hz, 3H), 0.72 – 0.65 (q, J = 7.9 Hz, 6H). ^{13}C NMR (100 MHz, $CDCl_3$) δ 149.9, 146.1, 137.2, 128.8, 123.3, 118.7, 115.0, 101.4, 31.4, 31.4, 31.1, 29.1, 28.0, 24.5, 23.2, 22.8, 22.7, 22.5, 14.3, 14.2, 6.9, 6.0, 5.8. HRMS-ESI (m/z): $[M+H]^+$ calcd for $C_{16}H_{31}OSi$ 267.2144; found 267.2154.

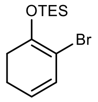

Yield: 90%.

^{1}H NMR (500 MHz, $CDCl_3$) δ 5.92 (d, J = 9.7 Hz, 1H), 5.48 (m, 1H), 2.71 (m, 0.5H), 2.59 (m, 1H), 2.48 (m, 0.5H), 2.32 (m, 1H), 2.15 (m, 1H), 1.05 – 1.00 (t, J = 8.0 Hz, 9H), 0.76 – 0.69 (q, J = 8.0 Hz, 6H). ^{13}C NMR (125 MHz, $CDCl_3$) δ 151.4 and 148.6, 129.3 and 128.6, 121.6 and 120.7, 106.1 and 99.3, 44.4 and 44.2, 31.5 and 30.3, 29.5 and 27.7, 23.9 and 19.7, 7.0 and 6. 9, 5.7. HRMS-ESI (m/z): $[M+H]^+$ calcd for $C_{12}H_{22}BrOSi$ 289.0623; found 289.0609.

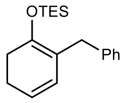

Yield: 60%.

^{1}H NMR (500 MHz, $CDCl_3$) δ 7.24 (d, J = 7.3 Hz, 2H), 7.20 – 7.13 (m, 3H), 5.68 (d, J = 9.6 Hz, 1H), 5.42 (m,1H), 3.47 (s, 2H), 2.34 – 2.24 (m, 4H), 0.99 (t, J = 8.0 Hz, 9H), 0.69 (q, J = 8.0 Hz, 6H). ^{13}C NMR (125 MHz, $CDCl_3$) δ 147.1, 141.3, 128.7, 128.2, 128.2, 125.8, 125.6, 118.9, 113.2, 33.9 28.9, 24.3, 6.8, 5.7. HRMS-ESI (m/z): $[M+H]^+$ calcd for $C_{19}H_{28}OSi$ 301.1988; found 301.1976.

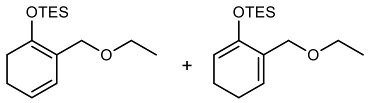

Yield: 77%, 45: 55.

^{1}H NMR (400 MHz, $CDCl_3$) δ 5.95 (d, *J* = 9.4 Hz, 1H), 5.53 – 5.47 (m, 1H), 4.05 (s, 2H), 3.44 (q, *J* = 7.0 Hz, 2H), 2.27 (m, 4H), 1.20 – 1.17 (t, *J* = 7.2 Hz, 3H), 0.99 (m, 9H), 0.70 (q, *J* = 8.0 Hz, 6H). ^{13}C NMR (100 MHz, $CDCl_3$) δ 149.8, 148.5, 133.6, 126.8, 125.8, 119.0, 112.2, 101.6, 69.2, 66.0, 65.0, 29.0, 24.1, 22.9, 22.0, 15.4, 15.3, 6.8, 5.6, 5.1. HRMS-ESI (m/z): $[M+Na]^+$ calcd for $C_{15}H_{28}NaO_2Si$ 291.1756; found 291.1746.

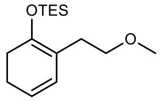

Yield: 80%.

^{1}H NMR (400 MHz, $CDCl_3$) δ 5.82 (d, *J* = 9.3 Hz, 1H), 5.46 (m, 1H), 3.44 – 3.37 (t, *J* = 8.0 Hz, 2H), 3.35 (d, *J* = 1.7 Hz, 3H), 2.45 – 2.38 (m, 2H), 2.26 (m, 4H), 1.02 (t, *J* = 7.9 Hz, 9H), 0.71 (q, *J* = 7.9 Hz, 6H). ^{13}C NMR (100 MHz, $CDCl_3$) δ 147.8, 128.6, 118.8, 111.0, 71.8, 58.5, 29.0, 28.8, 24.3, 6.8, 5.8. HRMS-ESI (m/z): $[M+H]^+$ calcd for $C_{15}H_{29}O_2Si$ 269.1937; found 269.1930.

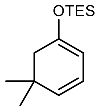

Yield: 72%.

^{1}H NMR (400 MHz, $CDCl_3$) δ 5.83 (dd, *J* = 9.4, 5.9 Hz, 1H), 5.30 (d, *J* = 9.4 Hz, 1H), 5.21 (d, *J* = 5.9 Hz, 1H), 2.25 (d, *J* = 1.2 Hz, 2H), 1.16 (s, 6H), 1.15 – 1.10 (t, *J* = 7.9 Hz, 9H), 0.83 (q, *J* = 7.9 Hz, 6H). ^{13}C NMR (100 MHz, $CDCl_3$) δ 153.2, 130.1, 121.8, 100.5, 43.9, 33.7, 28.4, 6.8, 5.2. HRMS-ESI (m/z): $[M+H]^+$ calcd for $C_{14}H_{27}OSi$ 239.1831; found 239.1825.

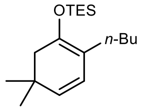

Yield: 80%.

^{1}H NMR (400 MHz, $CDCl_3$) δ 5.66 (d, *J* = 9.5 Hz, 1H), 5.19 (d, *J* = 9.5 Hz, 1H), 2.11 (s, 2H), 2.10 – 2.04 (m, 2H), 1.37 – 1.27 (m, 4H), 1.03 – 0.97 (m, 15H), 0.92 – 0.87 (m, 3H), 0.72 – 0.65 (m, 6H). ^{13}C NMR (100 MHz, $CDCl_3$) δ 145.3, 130.5, 125.9, 113.4, 44.1, 34.0, 31.0, 28.4, 27.7, 22.8, 14.2, 6.9, 6.0. HRMS-ESI (m/z): $[M+H]^+$ calcd for $C_{18}H_{35}OSi$ 295.2457; found 295.2445.

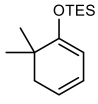

Yield: 61%.

^{1}H NMR (400 MHz, $CDCl_3$) δ 5.69 (m, 1H), 5.29 (m, 1H), 4.89 (d, *J* = 5.9 Hz, 1H), 2.06 (d, *J* = 4.4 Hz, 2H), 0.96 – 0.90 (m, 15H), 0.65 (q, *J* = 7.9 Hz, 6H). ^{13}C NMR (100 MHz, $CDCl_3$) δ 161.2, 124.2, 117.6, 99.0, 40.3, 35.4, 25.1, 6.9, 5.2. HRMS-ESI (m/z): $[M+H]^+$ calcd for $C_{14}H_{27}OSi$ 239.1831; found 239.1825.

Method B[35,36]

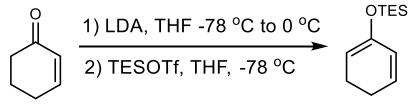

Supplementary Figure 2 Synthesis of 2-OTES or 1-OTES-4-Et cyclohexadiene

Literature procedure was followed, [35,36] except TESOTf was used and products were purified by flash column chromatography on silica gel (neutralized with 3% NEt_3/n-hexane, 3% NEt_3/n-hexane as eluent).

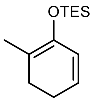

Yield: 78%.

^{1}H NMR (400 MHz, $CDCl_3$) δ 5.74 – 5.63 (m, 2H), 2.10 (s, 4H), 1.68 (s, 3H), 0.99 (t, *J* = 8.0 Hz, 9H), 0.66 (q, *J* = 8.0 Hz, 6H). ^{13}C NMR (100 MHz, $CDCl_3$) δ 142.2, 126.5, 125.5, 112.3, 28.7, 23.2, 16.1, 6.8, 5.4. HRMS-ESI (m/z): $[M+H]^+$ calcd for $C_{13}H_{25}OSi$ 225.1675; found 225.1668.

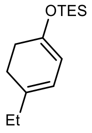

Yield: 74%.

^{1}H NMR (400 MHz, $CDCl_3$) δ 5.50 (m, 1H), 5.08 (m, 1H), 2.22 (m, 4H), 2.04 (m, 2H), 0.97 (m, 12H), 0.71 – 0.65 (m, 6H). ^{13}C NMR (100 MHz, $CDCl_3$) δ 151.6, 133.4, 117.1, 102.1, 29.5, 29.2, 28.2, 12.5, 6.8, 5.1. HRMS-ESI (m/z): $[M+H]^+$ calcd for $C_{14}H_{27}OSi$ 239.1831; found 239.1823.

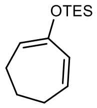

Yield: 80%.

^{1}H NMR (500 MHz, $CDCl_3$) δ 5.80 (dt, *J* = 10.9, 5.2 Hz, 1H), 5.71 – 5.64 (d, *J* = 10.9 Hz, 1H), 5.19 (t, *J* = 5.5 Hz, 1H), 2.28 (q, *J* = 5.5 Hz, 2H), 2.15 (q, *J* = 5.8 Hz, 2H), 1.82 (m, 2H), 0.98 (t, *J* = 8.0 Hz, 9H), 0.66 (q, *J* = 8.0 Hz, 6H). ^{13}C NMR (125 MHz, $CDCl_3$) δ 148.3, 133.3, 128.3, 112.4, 31.5, 27.2, 26.8, 6.8, 5.1. HRMS-ESI (m/z): $[M+H]^+$ calcd for $C_{13}H_{25}OSi$ 225.1675; found 225.1667.

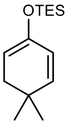

Yield: 83%.

^{1}H NMR (400 MHz, $CDCl_3$) δ 5.58 (dd, *J* = 9.9, 2.1 Hz, 1H), 5.53 (d, *J* = 9.9 Hz, 1H), 4.78 (m, 1H), 2.11 (d, *J* = 4.7 Hz, 2H), 1.01 (s, 6H), 0.98 (t, *J* = 8.0 Hz, 9H), 0.70 – 0.63 (q, *J* = 8.0 Hz, 6H). ^{13}C NMR (100 MHz, $CDCl_3$) δ 147.5, 140.1, 124.0, 101.1, 37.2, 31.3, 27.9, 6.8, 5.1. HRMS-ESI (m/z): $[M+H]^+$ calcd for $C_{14}H_{27}OSi$ 239.1831; found 239.1824.

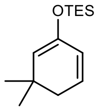

Yield: 75%.

^{1}H NMR (400 MHz, $CDCl_3$) δ 5.75 (m, 1H), 5.69 (m, 1H), 4.65 (m, 1H), 2.04 (dd, J = 4.0, 1.7 Hz, 2H), 1.01 – 0.95 (m, 15H), 0.66 (q, J = 7.9 Hz, 6H). ^{13}C NMR (100 MHz, $CDCl_3$) δ 147.0, 127.6, 125.4, 114.4, 38.2, 31.9, 28.8, 6.8, 5.1. HRMS-ESI (m/z): $[M+H]^+$ calcd for $C_{14}H_{27}OSi$ 239.1831; found 239.1824.

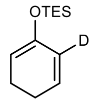

Yield: 80%, 95% D.

^{1}H NMR (400 MHz, $CDCl_3$) δ 5.85 (t, J = 4.3 Hz, 1H), 5.74 – 5.70 (m, 0.05H), 4.88 (t, J = 4.3 Hz, 1H), 2.20 – 2.03 (m, 4H), 0.98 (t, J = 8.0 Hz, 9H), 0.67 (q, J = 8.0 Hz, 6H). ^{13}C NMR (100 MHz, $CDCl_3$) δ 148.4, 128.8, 126.3 (t), 102.2, 22.7, 21.9, 6.8, 5.1. HRMS-ESI (m/z): $[M+H]^+$ calcd for $C_{12}H_{22}DOSi$ 212.1581; found 212.1576.

Method C[38]

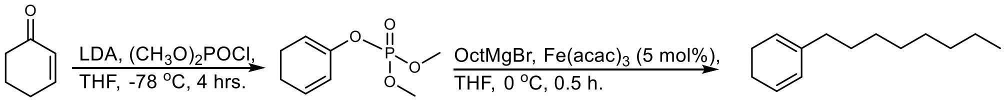

Supplementary Figure 3 Synthesis of 2-alkyl cyclic 1,3-diene

Target compound was synthesized as same as the literature.[38]

Method D[37]

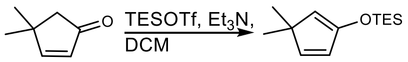

Supplementary Figure 4 Synthesis of 5,5-dimethyl-2-OTES-cyclopentadiene

Literature procedure was followed, [37] except product was purified by flash column chromatography on silica gel (buffered with 3% NEt_3/n-hexane, 3% NEt_3/n-hexane as eluent).

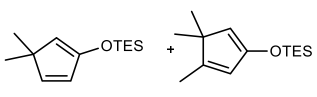

Yield: 69%, 62: 38.

^{1}H NMR (500 MHz, $CDCl_3$) δ 6.18 – 6.12 (m, 1H), 5.93 (dd, J = 5.4, 1.6 Hz, 1H), 5.14 – 5.10 (m, 1H), 1.15 (s, 3H), 1.07 – 0.97 (m, 12H), 0.75-0.65 (m, 6H). ^{13}C NMR (125 MHz, $CDCl_3$) δ 152.6, 147.4, 146.2, 144.2, 128.1, 127.8, 126.3, 118.6, 49.3, 49.2, 23.5, 22.2, 6.7 5.3, 4.9. HRMS-ESI (m/z): $[M+H]^+$ calcd for $C_{13}H_{25}OSi$ 225.1675, found 225.1663.

Method E[39,40]

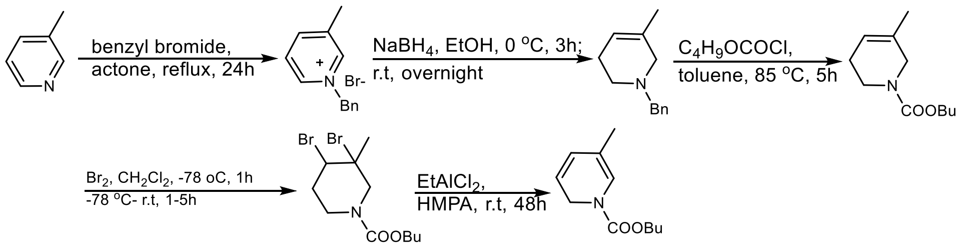

Supplementary Figure 5 Synthesis of heteroatom-containing cyclic 1,3-diene

Literature procedure was followed.[39,40]

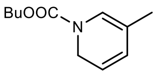

Yield: 68%.

^{1}H NMR (500 MHz, $CDCl_3$) δ 6.60 (dd, J = 55.2, 7.8 Hz, 1H), 5.57 – 5.51 (m, 1H), 5.06 (m, 1H), 4.18 (s, 2H), 4.16 – 4.11 (m, 2H), 1.67 (d, J = 9.9 Hz, 3H), 1.61 (dd, J = 14.5, 6.5 Hz, 2H), 1.37 (m, 2H), 0.94 – 0.89 (m, 3H). ^{13}C NMR (125 MHz, $CDCl_3$) δ 154.3 and 153.5, 128.8 and 128.0, 123.5 and 122.8, 117.0 and 116.6, 104.7, 66.0, 48.0 and 47.8, 31.0, 20.8, 19.2, 13.8. HRMS-ESI (m/z): $[M+H]^+$ calcd for $C_{11}H_{18}NO_2$ 196.1338, found 196.1331.

General procedure for the enantioselective cross-hydroalkenylation reaction:

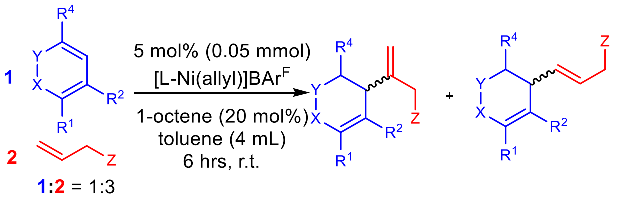

Supplementary Figure 6 General expression of the cross-hydroalkenylation reaction

In situ generation of [chiral NHC-Ni(allyl)]BAr^F catalyst. In a glove box, the [chiral NHC-Ni(allyl)Cl] complex solution was filtered to another oven-dried test tube with 0.05 mmol NaBAr^F, and the residue was rinsed by toluene (3*0.2 ml). After 0.2 mmol 1-octene was added (for NiH or its equiv. generation)[11-14], the mixture was stirred at r.t. for 1 hr before use.

General cross-hydroalkenylation procedure. In a glove box, a toluene solution of cyclic 1,3-diene and terminal olefin (1:3, 2 ml) were added into the indicated catalyst. This solution was allowed to stir for 6 hrs at r.t., except otherwise indicated.

General workup procedure. After stirring for the indicated time at r.t., the reaction mixture was added a spatula of K_2CO_3(s), diluted with 4 mL of hexane, and was stirred in open air for 1 hr. The mixture was

then filtered through a short plug of silica gel and rinsed with 75 mL EA/hexane (3: 2, buffered with 0.5 mol% NEt_3). The solvent was removed carefully on rotary evaporation at below 30 °C.

General procedure for product analysis. The residue was then subjected to ^1H NMR analysis (d1 = 10s) by using nitromethane as standard. Product structure was confirmed by chromatography isolation on silica gel (0.5% EA/Hex as eluent, buffered with 0.5% NEt_3) and NMR spectra. Ee was determined by HPLC (HPLC condition was described under specific compound). Optical rotation was measured by a Rudolph Autopol I Polarimeter at the sodium D line in chloroform.

Representative procedure for cross-hydroalkenylation of 1a and 2a to give 3aa. A toluene solution of 1a and 2a (1:3, 2 ml) was added in one-pot to the above in situ generated [**L3**-Ni(allyl)]BAr^F catalyst (0.05 mmol) in toluene (2 ml) sequentially. (For Table 1, entry 3) After stirring for 6 hrs at r.t., the reaction mixture was added a spatula of K_2CO_3(s), diluted with 4 mL of hexane, and was stirred in open air for 1 hr. The mixture was then filtered through a short plug of silica gel and rinsed with 75 mL EA/hexane (3: 2, buffered with 0.5 mol% NEt_3). The solvent was removed carefully on rotary evaporation at below 30 °C, and the residual was collected for analysis. Yield: >95%, **3**:**4** = 95:5. After column chromatography, the pure product was collected to do HPLC analysis (OD-H, hexane/iPrOH = 100/0, 0.7 ml/min, 20 °C, 210 nm) and to measure optical rotation. Ee = 87%, $[\alpha]_D^{20.0}$ = +65 (c = 0.40, $CHCl_3$).

The absolute configuration of the product was determined by comparison with relevant literature.[41-44]

Supplementary Table 4 Optical rotation of known compounds (for comparison)

Compound	Optical Rotation	Reference
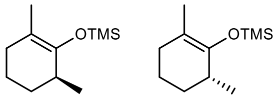 72: 28	$[\alpha]_D$ = -9.7 (c = 1.8, $CHCl_3$)	*Tetrahedron* **2002**, *58,* 4573.
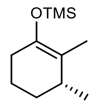	+	*Tetrahedron Lett.* **2009**, *50,* 5723.
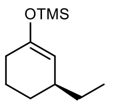	$[\alpha]_D^{20.0}$ = -4.7 (c = 5.01, $CHCl_3$)	*Org. Lett.* **2002**, *4,* 3835.
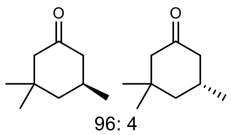 96: 4	$[\alpha]_D^{20.0}$ = +22 (c = 1.0, $CHCl_3$)	*Org. Lett.* **2010**, *12,* 300.

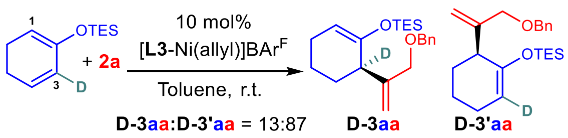

Supplementary Figure 7 Chiral L3-Ni(II) 1,3-allylic shift investigation

D-Labeling Experiment. The procedure is the same as the reaction with **1a** and **2a** in the representative procedure, except 10 mol% catalyst [**L3**-Ni(allyl)]BAr^F and **D-1a** were used.

Yield: >95%, **D-3aa**: **D-3’aa**= 13: 87.

D-3’aa, formed only after 1,3-allylic shift, was confirmed as major product according to isolation and NMR spectra.

^{1}H NMR (500 MHz, $CDCl_3$) δ 7.37 – 7.32 (m, 4H), 7.28 (m, 1H), 5.21 (d, J = 1.8 Hz, 1H), 5.06 – 5.03 (m, 1H), 4.95-4.92 (m, 0.13H), 4.56 (d, J = 12.0 Hz, 1H), 4.45 (d, J = 11.9 Hz, 1H), 4.10 (d, J = 13.1 Hz, 1H), 3.96 (d, J = 13.2 Hz, 1H), 2.87 (t, J = 5.8 Hz, 0.87H), 2.01 (m,2H), 1.74 (m, 1H), 1.64 (m,1H), 1.56 (m, 1H), 1.45 (m, 1H), 0.94 (t, J = 8.0 Hz, 9H), 0.66 – 0.60 (m, 6H). ^{13}C NMR (125 MHz, $CDCl_3$) δ 150.6, 146.8, 138.8, 128.4, 127.8, 127. 6, 113.4, 72.3, 71.6, 43.2, 28.8, 23.9, 19.2, 7.0, 5.2.

HRMS-ESI (m/z): $[M+H]^+$ calcd for $C_{22}H_{34}DO_2Si$ 360.2469, found 360.2462.

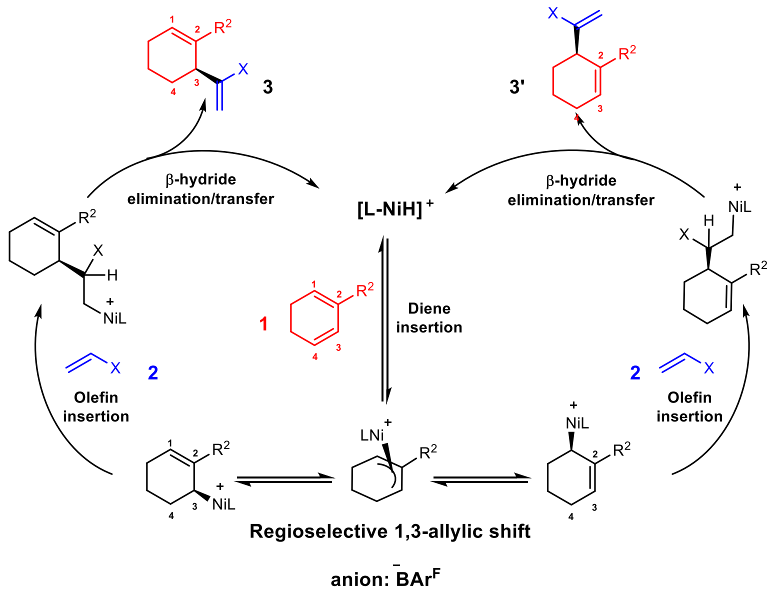

Supplementary Figure 8 Proposed catalytic cycle at this stage

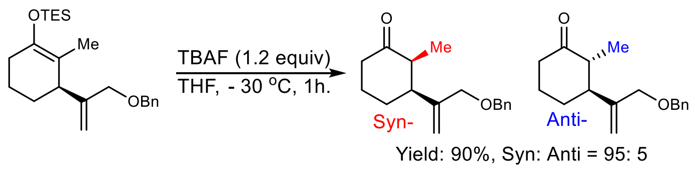

Supplementary Figure 9 Deprotection of silyl enol ether for *syn*-product synthesis

Selective Deprotection Method I.[15] An oven-dried 25 ml round bottom flask equipped with a magnetic stir bar, was sealed with a rubber septum and then evacuated and backfilled with nitrogen (3 cycles). The flask was charged with a solution of the cross-hydroalkenylation product (0.1 mmol, 1.0 eq.) in anhydrous THF (2 ml) and then cooled to -30 °C. After stirring for 5 min, a solution of TBAF (1.0 M soln in THF, 0.12 mmol, 1.2 eq.) was added dropwise over 3 mins. Stirring was continued for 1 hr at -30 °C at which time TLC indicated complete consumption of the cross-hydroalkenylation product. The reaction was quenched with H_2O and then hexanes was added. After extraction, the organic phase was collected, and the aqueous phase was extracted twice with hexanes. Then, the combined organic phase was washed with brine, dried over anhydrous Na_2SO_4, filtered and concentrated under reduced pressure. The residue was purified by flash chromatography on silica gel (5% EA/hexane) to get the desired syn-product.

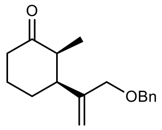

^{1}H NMR (400 MHz, $CDCl_3$) δ 7.38 – 7.27 (m, 5H), 5.26(s, 1H), 4.88 (s, 1H), 4.48 (s, 2H), 3.90 (s, 2H), 2.77 (m, 1H), 2.64 (m, 1H), 2.52 – 2.43 (m, 1H), 2.30 – 2.22 (m, 1H), 2.02 (m, 1H), 1.85 (m, 1H), 1.79 – 1.72 (m, 1H), 1.70 (m, 1H), 1.01 (d, J = 7.1 Hz, 3H). ^{13}C NMR (100 MHz, $CDCl_3$) δ 214.8, 145.0, 138.2, 128.5, 127.8, 127.7, 114.0, 72.9, 72.3, 47.1, 43.9, 38.8, 25.2, 24.0, 12.1. HRMS-ESI (m/z): $[M+H]^+$ calcd for $C_{17}H_{23}O_2$ 259.1698, found 259.1692.

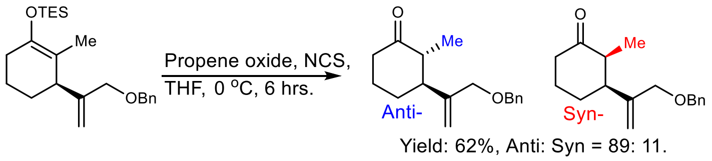

Supplementary Figure 10 Deprotection of silyl enol ether for *anti*-product synthesis

Selective Deprotection Method II.[45] An oven-dried 25 ml round bottom flask equipped with a magnetic stir bar, was sealed with a rubber septum and then evacuated and backfilled with nitrogen (3 cycles). The flask was charged with a solution of the cross-hydroalkenylation product (0.1 mmol, 1.0 eq.) in

anhydrous THF (2 ml), 5 drops propylene epoxide was added, the solution was cooled to 0 °C. After stirring the mixture for 5 mins, NCS (1.1 eq.) was added. Stirring was continued for 3h at 0 °C and then additional NCS (1.1 eq.) was added. The solution was warm to room temperature slowly and stirred overnight. The reaction was quenched with saturated $NaHCO_3$ solution and stirred for 30 mins. Hexanes was added. After extraction, the organic phase was collected, and the aqueous phase was extracted twice with hexanes. Then the combined organic phase was washed with brine, dried over anhydrous Na_2SO_4, filtered and concentrated under reduced pressure. The residue was purified by flash chromatography on silica gel (5% EA/hexane) to get the desired anti-product.

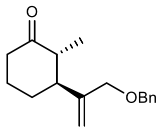

^{1}H NMR (500 MHz, $CDCl_3$) δ 7.37 – 7.29 (m, 5H), 5.22 (s, 1H), 5.03 (s, 1H), 4.56 – 4.49 (dd, *J* = 17.5, 12.0 Hz, 2H), 4.06 – 3.95 (dd, *J* = 30.5, 12.5 Hz, 2H), 2.54 – 2.45 (m, 1H), 2.43 (m, 1H), 2.34 (m, 1H), 2.13 (td, *J* = 11.8, 3.5 Hz, 1H), 2.06 (m, 1H), 1.92 (m, 1H), 1.81 – 1.72 (m, 1H), 1.65 (m, 1H), 0.99 (d, *J* = 6.4 Hz, 3H). ^{13}C NMR (125 MHz, $CDCl_3$) δ 212.9, 147.3, 138.3, 128.6, 127.8, 127.7, 113.3, 72.4, 72.2, 50.9, 48.8, 41.9, 32.5, 26.4, 12.4. HRMS-ESI (m/z): $[M+H]^+$ calcd for $C_{17}H_{23}O_2$ 259.1698, found 259.1692.

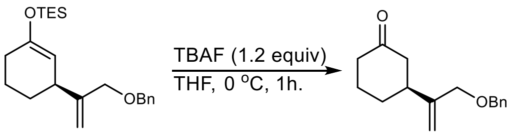

Yield: >95%.

Supplementary Figure 11 Deprotection of silyl enol ether to ketone for HPLC analysis

General Deprotection Method III.[15] An oven-dried 25 ml round bottom flask equipped with a magnetic stir bar, was sealed with a rubber septum and then evacuated and backfilled with nitrogen (3 cycles). The flask was charged with a solution of the cross-hydroalkenylation product (0.1 mmol, 1.0 eq.) in anhydrous THF (2 ml) and then cooled to 0 °C. After stirring for 5 min, a solution of TBAF (1.0 M soln in THF, 0.12 mmol, 1.2 eq.) was added dropwise over 3 mins. Stirring was continued for 1 hr at 0 °C at which time TLC indicated complete consumption of the cross-hydroalkenylation product. The reaction was quenched with H_2O and then hexanes was added. After extraction, the organic phase was collected, and the aqueous phase was extracted twice with hexanes. Then, the combined organic phase was washed

with brine, dried over anhydrous Na_2SO_4, filtered and concentrated under reduced pressure. The residue was purified by flash chromatography on silica gel (5% EA/hexane) to get the corresponding ketone.

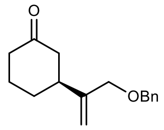

^{1}H NMR (500 MHz, $CDCl_3$) δ 7.42 – 7.23 (m, 5H), 5.15 (s, 1H), 4.99 (s, 1H), 4.52 – 4.45 (dd, J = 16.5, 11.5 Hz, 2H), 4.01 (dd, J = 21.0, 12.5 Hz, 2H), 2.64 – 2.46 (m, 2H), 2.44 – 2.25 (m, 3H), 2.11 – 1.92 (m,2H), 1.73 – 1.56 (m, 2H). ^{13}C NMR (125 MHz, $CDCl_3$) δ 211.5, 148.2, 138.2, 128.6, 127.8, 127.8, 112.4, 72.3, 72.2, 46.9, 41.5, 41.4, 30.4, 25.6. HRMS-ESI (m/z): $[M+H]^+$ calcd for $C_{16}H_{21}O_2$ 245.1542; found 245.1532.

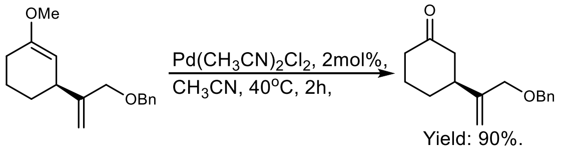

Supplementary Figure 12 Deprotection of methyl enol ether for ketone synthesis

General Deprotection Method IV.[46] An oven-dried 25 ml round bottom flask equipped with a magnetic stir bar, was sealed with a rubber septum and then evacuated and backfilled with nitrogen (3 cycles). The flask was charged with a solution of the cross-hydroalkenylation product (0.1 mmol, 1.0 equiv) in anhydrous THF (2 ml) and then warm to 40 °C. $Pd(CH_3CN)_2Cl_2$ (2 mol%) was added, stirring was continued for 2 hrs at 40 °C at which time TLC indicated complete consumption of the cross-hydroalkenylation product. The reaction was quenched with saturated NH_4Cl solution and then hexanes was added. After extraction, the organic phase was collected, and the aqueous phase was extracted twice with hexanes. Then, the combined organic phase was washed with brine, dried over anhydrous Na_2SO_4, filtered and concentrated under reduced pressure. The residue was purified by flash chromatography on silica gel (5% EA/hexane) to get the corresponding ketone.

Characterization Data of Cross-hydroalkenylation Product and Corresponding Ketone Product by Deprotection

I) Table 1

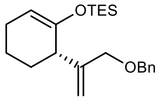

(3aa): ^{1}H NMR (400 MHz, $CDCl_3$) δ 7.37 – 7.33 (m, 4H), 7.32 – 7.27 (m, 1H), 5.21 (s, 1H), 5.05 (s, 1H), 4.94 (t, J = 3.9 Hz, 1H), 4.56 (d, J = 11.9 Hz, 1H), 4.45 (d, J = 11.9 Hz, 1H), 4.10 (d, J = 13.1 Hz, 1H), 3.96 (d, J = 13.1 Hz, 1H), 2.88 (m, 1H), 2.02 (m, 2H), 1.81 – 1.63 (m, 2H), 1.55 – 1.39 (m, 2H), 0.97 – 0.87 (m, 9H), 0.69 – 0.57 (m, 6H). ^{13}C NMR (100 MHz, $CDCl_3$) δ 150.5, 146.7, 138.6, 128.3, 127.6, 127.4, 113.2, 104.6, 72.1, 71.5, 43.1, 28.6, 23.9, 19.0, 6.8, 5.0. HRMS-ESI (m/z): $[M+H]^+$ calcd for $C_{22}H_{35}O_2Si$ 359.2406; found 359.2418. Optical Rotation: $[\alpha]_D^{20.0}$ = +65 for L3 (c = 0.40, $CHCl_3$).

HPLC condition: OD-H, hexane/iPrOH = 100/0, 0.7 ml/min, 20 °C, 210 nm.

From L1, Ee = 78%., From L2, ee = 54% From L3, ee = 87%.

Supplementary Figure 13 HPLC spectra for racemic and chiral 3aa

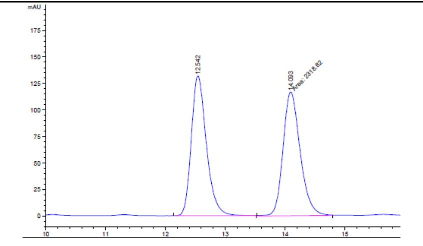

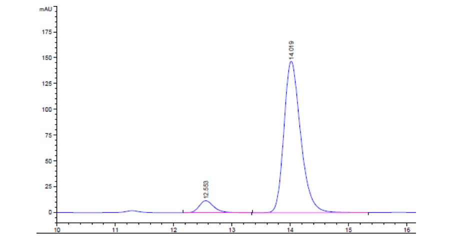

Signal 1: DAD1 B, Sig=210,4 Ref=360,100

Peak #	RetTime [min]	Type	Width [min]	Area [mAU*s]	Area %
1	12.542	BB	0.2641	2306.42578	49.8660
2	14.093	MM	0.3312	2318.82202	50.1340

Totals : 4625.24780

Signal 1: DAD1 B, Sig=210,4 Ref=360,100

Peak #	RetTime [min]	Type	Width [min]	Area [mAU*s]	Area %	Name
1	12.553	BB	0.2692	205.84492	6.5593	?
2	14.019	BB	0.3049	2932.38550	93.4407	?

Totals : 3138.23042

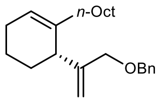

(3ba): ^{1}H NMR (400 MHz, $CDCl_3$) δ 7.38 – 7.32 (m, 4H), 7.31 – 7.26 (m, 1H), 5.56 (m, 1H), 5.22 (s, 1H), 4.90 (s, 1H), 4.57 (d, *J* = 11.9 Hz, 1H), 4.46 (d, *J* = 11.9 Hz, 1H), 4.09 (d, *J* = 12.8 Hz, 1H), 3.91 (d, *J* = 12.9 Hz, 1H), 2.82 (m, 1H), 1.98 (m, 2H), 1.88 (t, *J* = 7.7 Hz, 2H), 1.62 (m, 2H), 1.56 – 1.26 (m, 8H), 0.87 (t, *J* = 6.8 Hz, 3H). ^{13}C NMR (100 MHz, $CDCl_3$) δ 147.5, 138.6, 138.4, 128.5, 127.7, 127.6, 123.2, 113.8, 72.6, 72.0, 40.5, 35.9, 32.0, 29.7, 29.5, 28.3, 27.7, 25.3, 22.8, 18.2, 14.3. HRMS-ESI (m/z): $[M+H]^+$ calcd for. $C_{24}H_{37}O$ 341.2844; found 341.2832. Optical Rotation: $[\alpha]_D^{27.1}$ = +80 (*c* = 0.40, $CHCl_3$).

HPLC condition: OD-H+OD-H, hexane/iPrOH = 100/0, 0.5 ml/min, 20 °C, 210 nm.

Ee = 6%.

Supplementary Figure 14 HPLC spectra for racemic and chiral 3ba

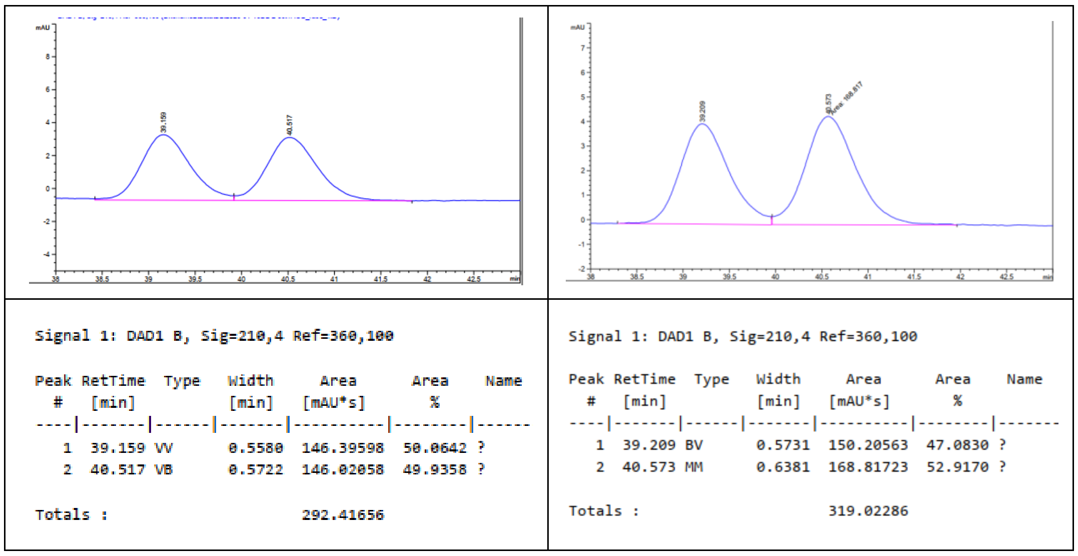

Signal 1: DAD1 B, Sig=210,4 Ref=360,100

Peak #	RetTime [min]	Type	Width [min]	Area [mAU*s]	Area %	Name
1	39.159	VV	0.5580	146.39598	50.0642	?
2	40.517	VB	0.5722	146.02058	49.9358	?
Totals :				292.41656		

Signal 1: DAD1 B, Sig=210,4 Ref=360,100

Peak #	RetTime [min]	Type	Width [min]	Area [mAU*s]	Area %	Name
1	39.209	BV	0.5731	150.20563	47.0830	?
2	40.573	MM	0.6381	168.81723	52.9170	?
Totals :				319.02286		

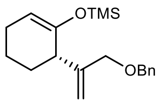

(3ca): ^{1}H NMR (400 MHz, $CDCl_3$) δ 7.42 – 7.34 (m, 4H), 7.32 – 7.28 (m, 1H), 5.24 (s, 1H), 5.06 (s, 1H), 4.98 (t, J = 3.8 Hz, 1H), 4.59 (d, J = 11.8 Hz, 1H), 4.46 (d, J = 11.8 Hz, 1H), 4.13 (d, J = 13.0 Hz, 1H), 3.97 (d, J = 13.0 Hz, 1H), 2.90 (t, J = 5.1 Hz, 1H), 2.10 – 1.99 (m, 2H), 1.84 – 1.72 (m, 1H), 1.71 – 1.53 (m, 3H), 1.54 – 1.42 (m, 1H), 0.18 (s, 9H). ^{13}C NMR (100 MHz, $CDCl_3$) δ 150.6, 146.8, 138.7, 128.4, 127.9, 127.6, 113.5, 105.5, 72.4, 71.5, 42.8, 29.9, 28.5, 24.0, 19.0, 0.44. HRMS-ESI (m/z): $[M+H]^+$ calcd for $C_{19}H_{29}O_2Si$ 317.1937; found 317.1928. Optical Rotation: $[\alpha]_D^{26.2}$ = +6.25° (c = 0.16, $CHCl_3$).

HPLC condition: OD-H, hexane/iPrOH = 100/0, 0.7 ml/min, 20 °C, 210 nm.

Ee = 75%.

Supplementary Figure 15 HPLC spectra for racemic and chiral 3ca

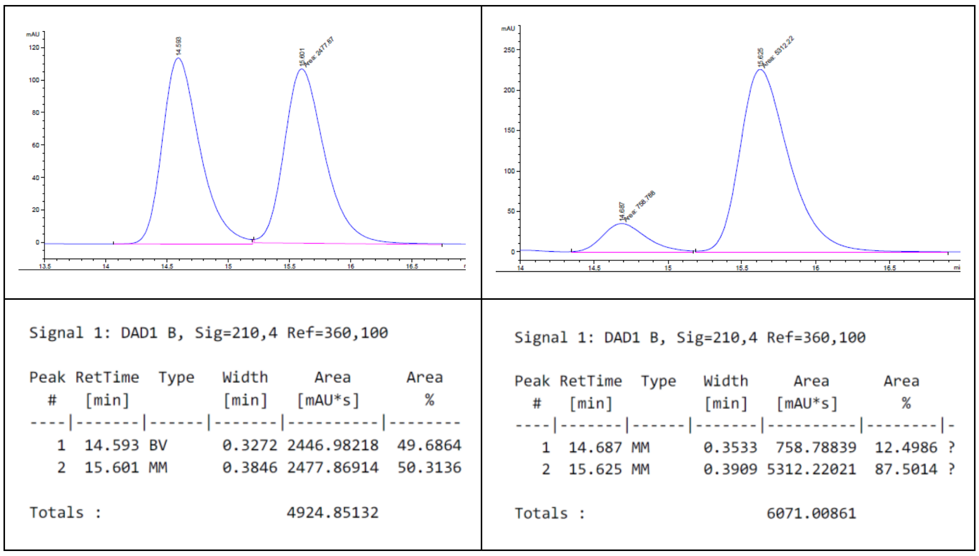

Signal 1: DAD1 B, Sig=210,4 Ref=360,100

Peak #	RetTime [min]	Type	Width [min]	Area [mAU*s]	Area %
1	14.593	BV	0.3272	2446.98218	49.6864
2	15.601	MM	0.3846	2477.86914	50.3136
Totals :				4924.85132	

Signal 1: DAD1 B, Sig=210,4 Ref=360,100

Peak #	RetTime [min]	Type	Width [min]	Area [mAU*s]	Area %
1	14.687	MM	0.3533	758.78839	12.4986 ?
2	15.625	MM	0.3909	5312.22021	87.5014 ?
Totals :				6071.00861	

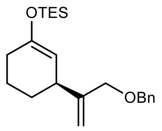

(3da): ^{1}H NMR (400 MHz, $CDCl_3$) δ 7.40 – 7.31 (m, 4H), 7.33 – 7.22 (m, 1H), 5.13 (s, 1H), 5.00 (s, 1H), 4.78 (m, 1H), 4.50 (dd, J = 22.0, 12.0 Hz, 2H), 4.05 (d, J = 12.7 Hz, 1H), 3.96 (d, J = 12.7 Hz, 1H), 2.96 (m, 1H), 2.14 – 1.94 (m, 2H), 1.81 – 1.66 (m, 2H), 1.62 – 1.51 (m, 1H), 1.41 – 1.30 (m, 1H), 0.98 (t, J = 7.9 Hz, 9H), 0.67 (q, J = 7.9 Hz, 6H). ^{13}C NMR (100 MHz, $CDCl_3$) δ 151.8, 150.1, 138.6, 128.5, 127.8, 127.7, 112.3, 106.6, 72.3, 72.2, 38.3, 29.9, 28.2, 21.1, 6.9, 5.2. HRMS-ESI (m/z): $[M+H]^+$ calcd for $C_{22}H_{35}O_2Si$ 359.2406; found 359.2398. Optical Rotation: $[\alpha]_D^{22.6}$ = -1.92° (c = 3.64, $CHCl_3$).

Ee was determined by the corresponding ketone product by silyl enol ether deprotection method III. Yield: >95%.

HPLC condition: AS-3, hexane/iPrOH = 99.0/1.0, 1.0 ml/min, 20 °C, 210 nm.

Ee = 48%.

Supplementary Figure 16 HPLC spectra for racemic and chiral 3da's deprotection product

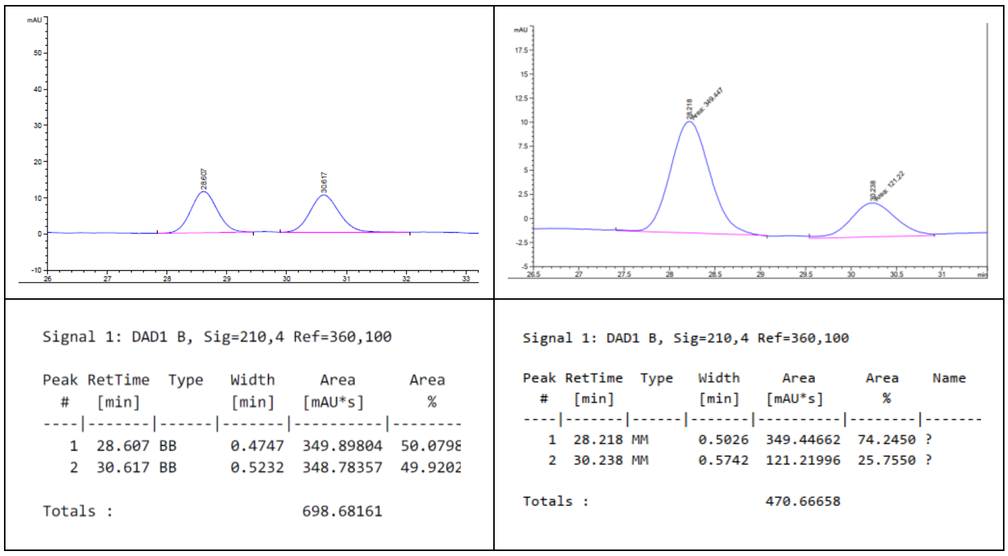

Signal 1: DAD1 B, Sig=210,4 Ref=360,100

Peak #	RetTime [min]	Type	Width [min]	Area [mAU*s]	Area %
1	28.607	BB	0.4747	349.89804	50.0798
2	30.617	BB	0.5232	348.78357	49.9202

Totals : 698.68161

Signal 1: DAD1 B, Sig=210,4 Ref=360,100

Peak #	RetTime [min]	Type	Width [min]	Area [mAU*s]	Area %	Name
1	28.218	MM	0.5026	349.44662	74.2450	?
2	30.238	MM	0.5742	121.21996	25.7550	?

Totals : 470.66658

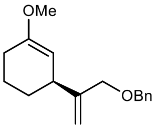

(3ea): ^{1}H NMR (400 MHz, $CDCl_3$) δ 7.39 – 7.31 (m, 4H), 7.30 – 7.23 (m, 1H), 5.14 (s, 1H), 5.03 (s, 1H), 4.57 – 4.42 (m, 3H), 4.07 (d, J = 12.7 Hz, 1H), 3.99 (d, J = 12.7 Hz, 1H), 3.51 (s, 3H), 3.16 – 2.95 (m, 1H), 2.18 – 1.97 (m, 2H), 1.84 – 1.68 (m, 2H), 1.64 – 1.51 (m, 1H), 1.47 – 1.33 (m, 1H). ^{13}C NMR (100 MHz, $CDCl_3$) δ 156.3, 150.3, 138.5, 128.4, 127.8, 127.6, 112.3, 96.3, 72.3, 72.1, 54.0, 37.8, 28.5, 27.8, 20.9. HRMS-ESI (m/z): $[M+H]^+$ calcd for $C_{17}H_{23}O_2$ 259.1698; found 259.1689. Optical Rotation: $[\alpha]_D^{22.8}$ = -2.53° (c = 2.10, $CHCl_3$).

Ee was determined by the corresponding ketone product by methyl enol ether deprotection method IV.

Yield: 90%.

HPLC condition: AS-3, hexane/iPrOH = 99.0/1.0, 1.0 ml/min, 20 °C, 210 nm.

Ee = 39%.

Supplementary Figure 17 HPLC spectra for racemic and chiral 3ea's deprotection product

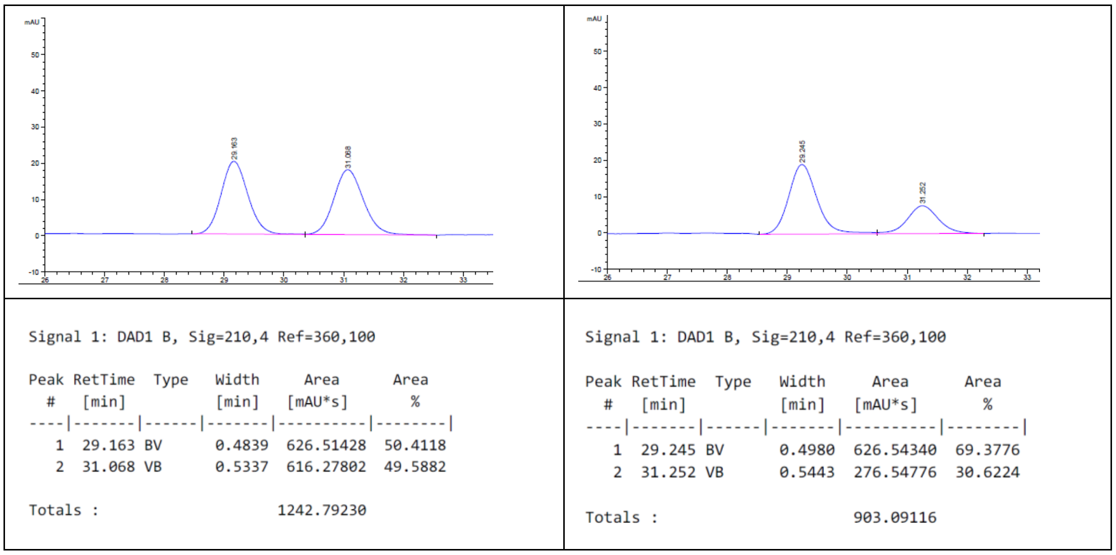

Signal 1: DAD1 B, Sig=210,4 Ref=360,100

Peak #	RetTime [min]	Type	Width [min]	Area [mAU*s]	Area %
1	29.163	BV	0.4839	626.51428	50.4118
2	31.068	VB	0.5337	616.27802	49.5882
Totals :				1242.79230	

Signal 1: DAD1 B, Sig=210,4 Ref=360,100

Peak #	RetTime [min]	Type	Width [min]	Area [mAU*s]	Area %
1	29.245	BV	0.4980	626.54340	69.3776
2	31.252	VB	0.5443	276.54776	30.6224
Totals :				903.09116	

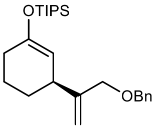

(3fa): ^{1}H NMR (400 MHz, $CDCl_3$) δ 7.34 (m, 4H), 7.30 – 7.24 (m, 1H), 5.12 (s, 1H), 5.00 (s, 1H), 4.79 (m, 1H), 4.55 – 4.44 (dd, J = 20.4, 12.0 Hz, 2H), 4.04 (d, J = 12.8 Hz, 1H), 3.96 (d, J = 12.8 Hz, 1H), 2.97 (m, 1H), 2.06 (m, 2H), 1.74 (m, 2H), 1.59 – 1.51 (m, 1H), 1.42 – 1.30 (m, 1H), 1.21 – 1.11 (m, 3H), 1.08 (d, J = 6.4 Hz, 18H). ^{13}C NMR (100 MHz, $CDCl_3$) δ 151.9, 150.1, 138.6, 128.5, 127.8, 127.6, 112.2, 106.1, 72.3, 72.2, 38.5, 30.0, 28.3, 21.3, 18.1, 12.7. HRMS-ESI (m/z): $[M+H]^+$ calcd for $C_{25}H_{41}O_2Si$ 401.2876; found 401.2868. Optical Rotation: $[\alpha]_D^{23.0}$ = -3.72^o (c = 2.69, $CHCl_3$).

Ee was determined by the corresponding ketone product by silyl enol ether deprotection method III.

Yield: >95%.

HPLC condition: AS-3, hexane/iPrOH = 99.0/1.0, 1.0 ml/min, 20 ^{o}C, 210 nm.

Ee = 29%.

Supplementary Figure 18 HPLC spectra for racemic and chiral 3fa's deprotection product

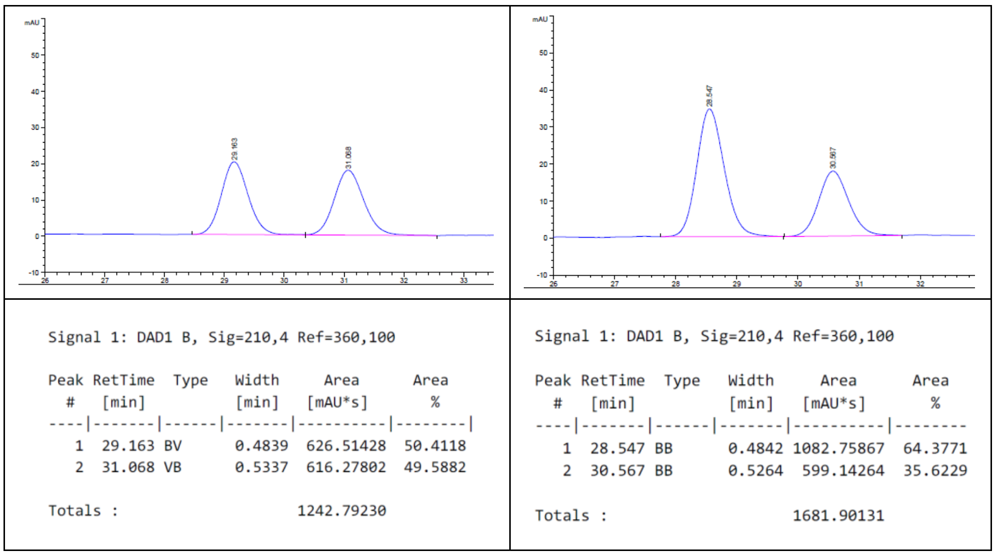

Signal 1: DAD1 B, Sig=210,4 Ref=360,100

Peak #	RetTime [min]	Type	Width [min]	Area [mAU*s]	Area %
1	29.163	BV	0.4839	626.51428	50.4118
2	31.068	VB	0.5337	616.27802	49.5882
Totals :				1242.79230	

Signal 1: DAD1 B, Sig=210,4 Ref=360,100

Peak #	RetTime [min]	Type	Width [min]	Area [mAU*s]	Area %
1	28.547	BB	0.4842	1082.75867	64.3771
2	30.567	BB	0.5264	599.14264	35.6229
Totals :				1681.90131	

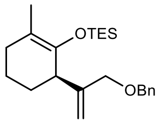

(3ga): ^{1}H NMR (500 MHz, $CDCl_3$) δ 7.37 – 7.32 (m, 4H), 7.31 – 7.26 (m, 1H), 5.23 (s, 1H), 5.00 (s, 1H), 4.53 (dd, J = 21.5, 12.0 Hz, 2H), 3.99 (dd, J = 22.5, 13.5 Hz, 2H), 2.84 (m, 1H), 1.99 – 1.90 (m, 2H), 1.73 – 1.65 (m, 2H), 1.61 (s, 3H), 1.59 – 1.52 (m, 1H), 1.47 – 1.39 (m, 1H), 0.93 (t, J = 7.9 Hz, 9H), 0.64 – 0.59 (q, J = 7.9 Hz, 6H). ^{13}C NMR (125 MHz, $CDCl_3$) δ 146.5, 143.2, 138.8, 128.4, 127.7, 127.5, 114.0, 113.32, 72.2, 72.1, 44.4, 30.4, 29.3, 19.2, 16.8, 7.0, 5.7. HRMS-ESI (m/z): $[M+H]^+$ calcd for $C_{23}H_{37}O_2Si$ 373.2563; found 373.2555. Optical Rotation: $[\alpha]_D^{23.0}$ = -21.12° (c = 1.36, $CHCl_3$).

HPLC condition: OD-H, hexane/iPrOH = 100/0, 0.5 ml/min, 20 °C, 210 nm.

Ee = 92%.

Supplementary Figure 19 HPLC spectra for racemic and chiral 3ga

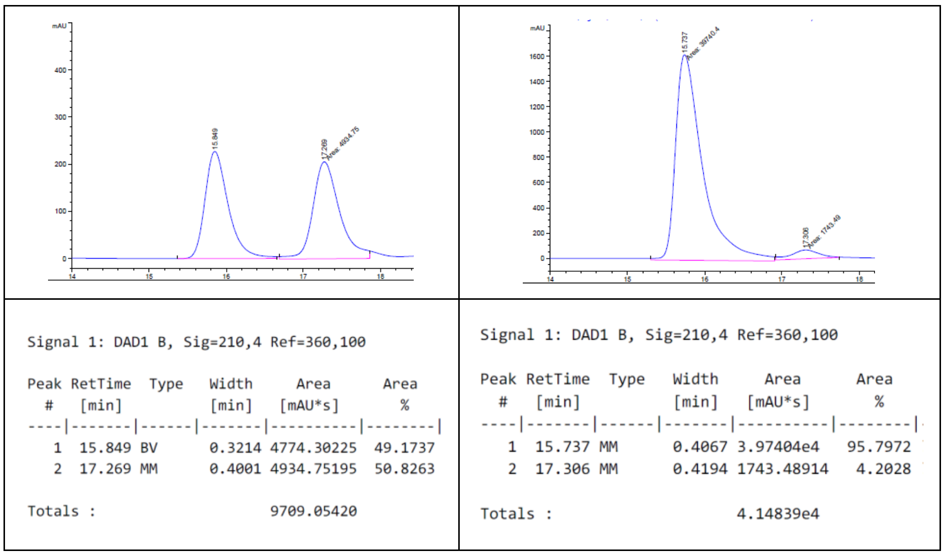

Signal 1: DAD1 B, Sig=210,4 Ref=360,100

Peak #	RetTime [min]	Type	Width [min]	Area [mAU*s]	Area %
1	15.849	BV	0.3214	4774.30225	49.1737
2	17.269	MM	0.4001	4934.75195	50.8263
Totals :				9709.05420	

Signal 1: DAD1 B, Sig=210,4 Ref=360,100

Peak #	RetTime [min]	Type	Width [min]	Area [mAU*s]	Area %
1	15.737	MM	0.4067	3.97404e4	95.7972
2	17.306	MM	0.4194	1743.48914	4.2028
Totals :				4.14839e4	

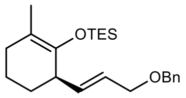

(4ga): E:Z > 95 : 5.

^{1}H NMR (500 MHz, $CDCl_3$) δ 7.34 (m, 4H), 7.31 – 7.26 (m, 1H), 5.74 (dd, *J* = 15.4, 7.3 Hz, 1H), 5.63 (dt, *J* = 15.5, 6.1 Hz, 1H), 4.50 (s, 2H), 4.05 – 3.97 (m, 2H), 2.76 (m, 1H), 1.95 (m, 2H), 1.76 (m, 1H), 1.62 (m, 2H), 1.59 (s, 3H), 1.48 (m, 1H), 0.97 (t, *J* = 8.0 Hz, 9H), 0.65 (q, *J* = 8.0 Hz, 6H). ^{13}C NMR (125 MHz, $CDCl_3$) δ 144.2, 138.7, 136.7, 128.4, 127.9, 127.6, 127.0, 113.0, 71.8, 71.1, 42.9, 30.7, 30.6, 19.7, 16.9, 7.0, 5.8. HRMS-ESI (m/z): $[M+H]^+$ calcd for $C_{23}H_{37}O_2Si$ 373.2563; found 373.2555. Optical Rotation: $[\alpha]_D^{23.0}$ = -7.46° (*c* = 1.00, $CHCl_3$).

HPLC condition: OD-H, hexane/iPrOH = 100/0, 0.5 ml/min, 20 °C, 210 nm.

Ee = 98%.

Supplementary Figure 20 HPLC spectra for racemic and chiral 4ga

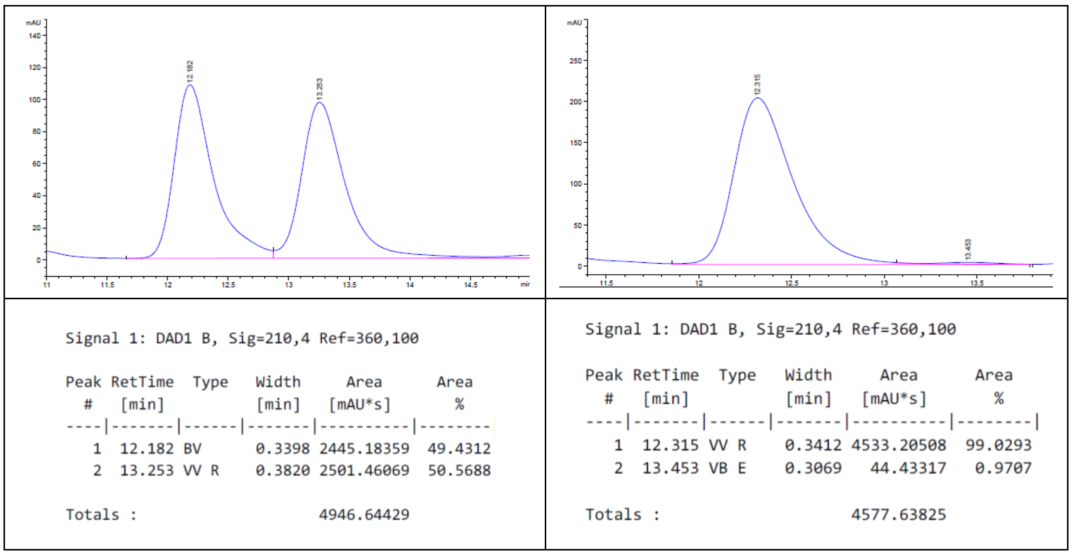

Signal 1: DAD1 B, Sig=210,4 Ref=360,100

Peak #	RetTime [min]	Type	Width [min]	Area [mAU*s]	Area %
1	12.182	BV	0.3398	2445.18359	49.4312
2	13.253	VV R	0.3820	2501.46069	50.5688
Totals :				4946.64429	

Signal 1: DAD1 B, Sig=210,4 Ref=360,100

Peak #	RetTime [min]	Type	Width [min]	Area [mAU*s]	Area %
1	12.315	VV R	0.3412	4533.20508	99.0293
2	13.453	VB E	0.3069	44.43317	0.9707
Totals :				4577.63825	

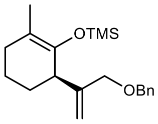

(3ha): ^{1}H NMR (500 MHz, $CDCl_3$) δ 7.37 – 7.32 (m, 4H), 7.31 – 7.26 (m, 1H), 5.23 (s, 1H), 5.00 (s, 1H), 4.53 (dd, J = 33.0, 12.0 Hz, 2H), 3.99 (dd, J = 36.5, 13.0 Hz, 2H), 2.84 (m, 1H), 1.99 – 1.90 (m, 2H), 1.73 – 1.65 (m, 2H), 1.61 (s, 3H), 1.59 – 1.52 (m, 1H), 1.47 – 1.39 (m, 1H), 0.12 (s, 9H). ^{13}C NMR (125 MHz, $CDCl_3$) δ 146.8, 142.9, 138.7, 128.5, 127.7, 127.6, 114.5, 113.4, 72.1, 44.3, 30.5, 29.9, 19.4, 17.0, 0.9. HRMS-ESI (m/z): $[M+H]^+$ calcd for $C_{20}H_{31}O_2Si$ 331.2093; found 331.2087. Optical Rotation: $[\alpha]_D^{23.0}$ = -8.69º (c = 2.65, $CHCl_3$).

HPLC condition: OD-H, hexane/iPrOH = 100/0, 1.0 ml/min, 20 °C, 210 nm.

Ee = 86%.

Supplementary Figure 21 HPLC spectra for racemic and chiral 3ha

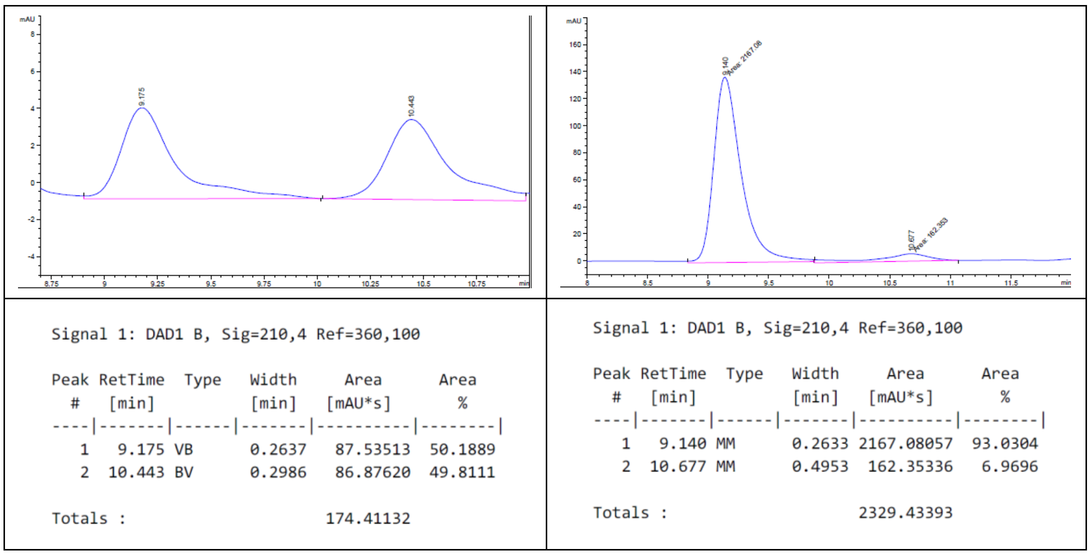

Signal 1: DAD1 B, Sig=210,4 Ref=360,100

Peak #	RetTime [min]	Type	Width [min]	Area [mAU*s]	Area %
1	9.175	VB	0.2637	87.53513	50.1889
2	10.443	BV	0.2986	86.87620	49.8111
Totals :				174.41132	

Signal 1: DAD1 B, Sig=210,4 Ref=360,100

Peak #	RetTime [min]	Type	Width [min]	Area [mAU*s]	Area %
1	9.140	MM	0.2633	2167.08057	93.0304
2	10.677	MM	0.4953	162.35336	6.9696
Totals :				2329.43393	

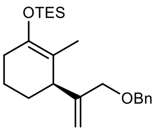

(3ia): ^{1}H NMR (400 MHz, $CDCl_3$) δ 7.35 (m, 4H), 7.32 – 7.27 (m, 1H), 5.21 (s, 1H), 4.92 (s, 1H), 4.56 (d, *J* = 11.9 Hz, 1H), 4.46 (d, *J* = 11.9 Hz, 1H), 4.06 (d, *J* = 12.9 Hz, 1H), 3.90 (d, *J* = 12.9 Hz, 1H), 2.81 (m, 1H), 2.04 (m, 2H), 1.63 (m, 2H), 1.55 – 1.50 (m, 5H), 0.99 (t, *J* = 8.0 Hz, 9H), 0.72 – 0.61 (q, *J* = 8.0 Hz, 6H). ^{13}C NMR (100 MHz, $CDCl_3$) δ 147.7, 145.8, 138.6, 128.5, 127.8, 127.6, 113.7, 112.4, , 72.5, 72.0, 43.3, 30.4, 27.6, 19.6, 15.1, 6.9, 5.8. HRMS-ESI (m/z): $[M+H]^+$ calcd for $C_{23}H_{36}O_2Si$ 373.2563; found 373.2556. Optical Rotation: $[\alpha]_D^{21.2}$ = -120° for L3 (*c* = 0.40, $CHCl_3$).

Ee was determined by the corresponding ketone product by silyl enol ether deprotection method III.

Yield: >95%.

HPLC condition: OD-H, hexane/iPrOH = 99.8/0.2, 0.2 ml/min, 20 °C, 210 nm.

From L2, ee = 90%, From L3, ee = 96%. (the right two peaks were used to calculate ee.)

Supplementary Figure 22 HPLC spectra for racemic and chiral 3ia's deprotection product

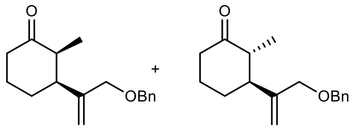

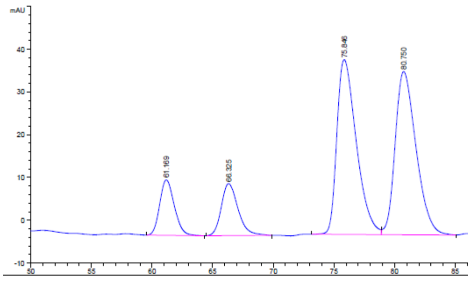

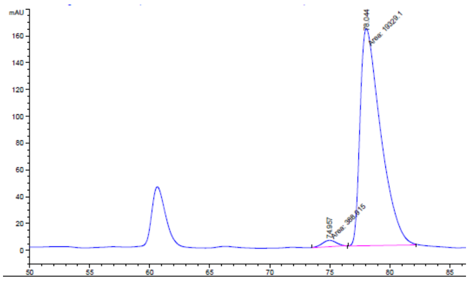

Signal 1: DAD1 B, Sig=210,4 Ref=360,100

Peak #	RetTime [min]	Type	Width [min]	Area [mAU*s]	Area %
1	61.169	BB	1.1538	1090.37097	9.5809
2	66.325	BB	1.2904	1168.18237	10.2646
3	75.846	BV	1.6076	4554.35742	40.0184
4	80.750	VB	1.7221	4567.74854	40.1361

Totals : 1.13807e4

Signal 1: DAD1 B, Sig=210,4 Ref=360,100

Peak #	RetTime [min]	Type	Width [min]	Area [mAU*s]	Area %
1	74.957	MM	1.3592	388.91528	1.9724
2	78.044	MM	1.9909	1.93291e4	98.0276

Totals : 1.97180e4

II) Figure 2

Set 1 Scope of R^1 & R^2

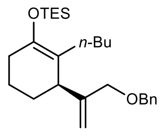

(3ja): ^{1}H NMR (400 MHz, $CDCl_3$) δ 7.34 (m, 4H), 7.28 (m, 1H), 5.21 (s, 1H), 4.92 (s, 1H), 4.56 (d, *J* = 11.9 Hz, 1H), 4.46 (d, *J* = 11.9 Hz, 1H), 4.06 (d, *J* = 12.8 Hz, 1H), 3.90 (d, *J* = 12.8 Hz, 1H), 2.95 (m, 1H), 2.38 (m, 1H), 2.15 – 1.95 (m, 2H), 1.67 – 1.48 (m, 4H), 1.39 – 1.17 (m, 5H), 1.00 (t, *J* = 8.0 Hz, 9H), 0.85 (t, *J* = 6.9 Hz, 3H), 0.67 (q, *J* = 8.0 Hz, 6H). ^{13}C NMR (100 MHz, $CDCl_3$) δ 148.0, 145.7, 138.7, 128.5, 127.7, 127.62, 116.7, 113.9, 72.6, 72.0, 40.4, 30.5, 30.4, 28.5, 27.6, 23.2, 19.4, 14.2, 7.0, 5.9. HRMS-ESI (m/z): $[M+H]^+$ calcd for $C_{26}H_{43}O_2Si$ 415.3032; found 415.3020. Optical Rotation: $[\alpha]_D^{25.0}$ = -77.5° (*c* = 0.40, $CHCl_3$).

Ee was determined by the corresponding ketone product by silyl enol ether deprotection method III. Yield: >95%.

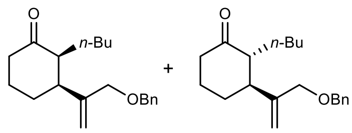

^{1}H NMR (400 MHz, $CDCl_3$) δ 7.39 – 7.26 (m, 5H), 5.26 (s, 0.52H), 5.22 (s, 0.48H), 5.01 (s, 0.48H), 4.91 (s, 0.52H), 4.57 – 4.45 (m, 2H), 4.06 – 3.92 (m, 1H), 3.90 (s, 1H), 2.76 (m, 0.52H), 2.50 – 1.04 (m, 13.48H), 0.84 (m, 3H). ^{13}C NMR (100 MHz, $CDCl_3$) δ 214.6, 212.9, 147.4, 145.2, 138.3, 138.2, 128.5, 127.8, 127.7, 114.0, 113.4, 72.8, 72.4, 72.1, 54.0, 53.0, 48.6, 43.8, 42.1, 39.0, 31.7, 30.2, 29.6, 27.2, 26.1, 25.4, 24.6, 23.1, 22.7, 14.1. HRMS-ESI (m/z): $[M+H]^+$ calcd for $C_{20}H_{29}O_2$ 301.2168; found 301.2161.

See next page for HPLC result.

HPLC condition: OD-H, hexane/iPrOH = 99.8/0.2, 0.2 ml/min, 20 °C, 210 nm.

Ee = 97%. (the left two peaks were used to calculate ee due to more effective separation.)

Supplementary Figure 23 HPLC spectra for racemic and chiral 3ja's deprotection product

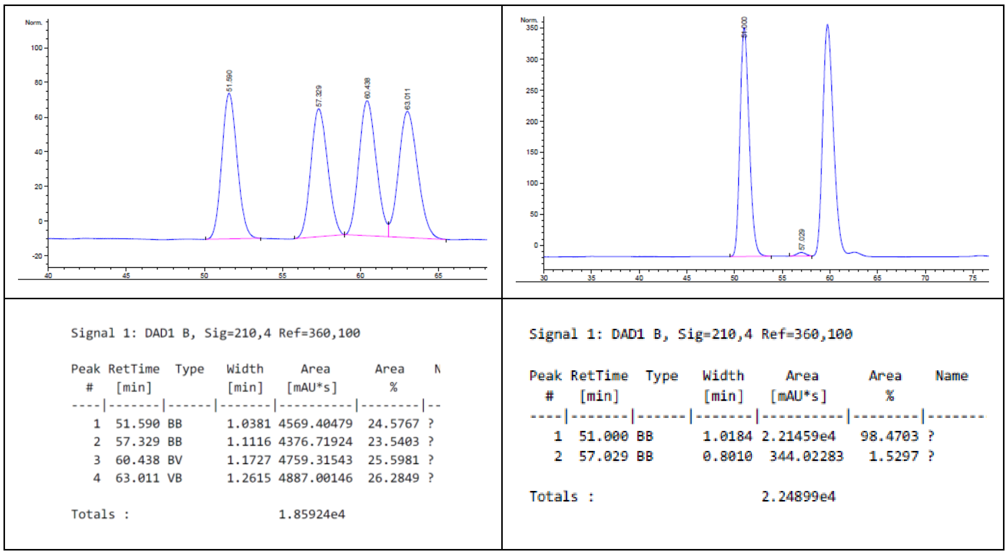

Signal 1: DAD1 B, Sig=210,4 Ref=360,100

Peak #	RetTime [min]	Type	Width [min]	Area [mAU*s]	Area %	N
1	51.590	BB	1.0381	4569.40479	24.5767	?
2	57.329	BB	1.1116	4376.71924	23.5403	?
3	60.438	BV	1.1727	4759.31543	25.5981	?
4	63.011	VB	1.2615	4887.00146	26.2849	?

Totals : 1.85924e4

Signal 1: DAD1 B, Sig=210,4 Ref=360,100

Peak #	RetTime [min]	Type	Width [min]	Area [mAU*s]	Area %	Name
1	51.000	BB	1.0184	2.21459e4	98.4703	?
2	57.029	BB	0.8010	344.02283	1.5297	?

Totals : 2.24899e4

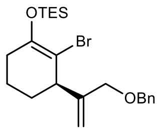

(3ka): ^{1}H NMR (400 MHz, $CDCl_3$) δ 7.43-7.34 (m, 4H), 7.33 – 7.28 (m, 1H), 5.34 (s, 1H), 5.09 (s, 1H), 4.64 (d, *J* = 11.7 Hz, 1H), 4.46 (d, *J* = 11.7 Hz, 1H), 4.14 (d, *J* = 12.9 Hz, 1H), 3.97 (d, *J* = 12.9 Hz, 1H), 3.35 (m, 1H), 2.32 – 2.13 (m, 2H), 1.89 – 1.63 (m, 4H), 1.05 (t, *J* = 7.9 Hz, 9H), 0.76 (q, *J* = 7.9 Hz, 6H). ^{13}C NMR (125 MHz, $CDCl_3$) δ 149.0, 146.8, 138.9, 128.5, 128.0, 127.7, 115.3, 104.2, 72.2, 71.7, 46.3, 31.8, 28.7, 19.0, 6.9, 5.7. HRMS-ESI (m/z): $[M+H]^+$ calcd for $C_{22}H_{34}BrO_2Si$ 437.1511; found 437.1506. Optical Rotation: $[\alpha]_D^{23.0}$ = -19.2° (*c* = 1.67, $CHCl_3$).

HPLC condition: OD-H, hexane/iPrOH = 100/0, 1.0 ml/min, 20 °C, 210 nm.

Ee = 97%.

Supplementary Figure 24 HPLC spectra for racemic and chiral 3ka

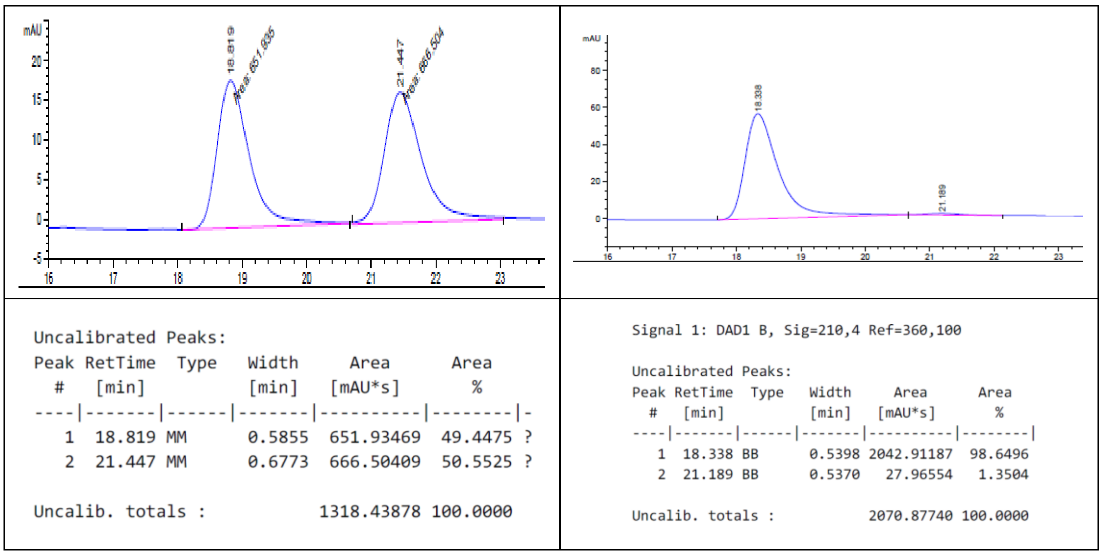

Uncalibrated Peaks:

Peak #	RetTime [min]	Type	Width [min]	Area [mAU*s]	Area %	
1	18.819	MM	0.5855	651.93469	49.4475	?
2	21.447	MM	0.6773	666.50409	50.5525	?

Uncalib. totals : 1318.43878 100.0000

Signal 1: DAD1 B, Sig=210,4 Ref=360,100

Uncalibrated Peaks:

Peak #	RetTime [min]	Type	Width [min]	Area [mAU*s]	Area %
1	18.338	BB	0.5398	2042.91187	98.6496
2	21.189	BB	0.5370	27.96554	1.3504

Uncalib. totals : 2070.87740 100.0000

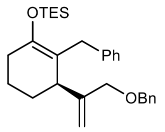

(3la): ^{1}H NMR (400 MHz, $CDCl_3$) δ 7.39 – 7.26 (m, 5H), 7.21 – 7.10 (m, 5H), 5.27 (s, 1H), 4.95 (s, 1H), 4.51 (d, *J* = 11.9 Hz, 1H), 4.43 (d, *J* = 11.9 Hz, 1H), 4.07 – 3.73 (m, 3H), 2.85 – 2.63 (m, 2H), 2.32 – 1.99 (m, 2H), 1.76 – 1.55 (m, 2H), 1.53 – 1.45 (m, 2H), 1.00 (t, *J* = 7.9 Hz, 9H), 0.69 (q, *J* = 7.9 Hz, 6H). ^{13}C NMR (100 MHz, $CDCl_3$) δ 147.8, 146.8, 141.9, 138.6, 129.1, 128.5, 128.2, 127.7, 127.6, 125.5, 115.4, 113.6, 72.6, 72.2, 39.7, 34.0, 30.3, 27.5, 19.2, 7.0, 6.0. HRMS-ESI (m/z): $[M+H]^+$ calcd for $C_{29}H_{41}O_2Si$ 449.2876; found 449.2866. Optical Rotation: $[\alpha]_D^{23.0}$ = -78.66° (*c* = 2.96, $CHCl_3$).

Ee was determined by the corresponding ketone product by silyl enol ether deprotection method III. Yield: >95%.

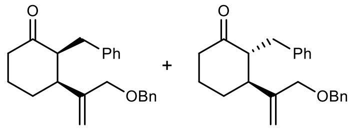

^{1}H NMR (400 MHz, $CDCl_3$) δ 7.38 – 7.26 (m, 5H), 7.22 – 7.03 (m, 5H), 5.38 (s, 0.44H), 5.31 (s, 0.56H), 5.10 (s, 0.57H), 4.91 (s, 0.43H), 4.56 – 4.44 (m, 2H), 4.06 – 3.80 (m, 2H), 3.10 (dd, *J* = 13.6, 6.8 Hz, 0.44H), 3.00 (dd, *J* = 13.6, 9.2 Hz, 0.57H), 2.83 – 2.62 (m, 2.52H), 2.56 - 2.48 (m, 0.46H), 2.43 – 2.36 (m, 0.52H), 2.36 – 2.25 (m, 1.60H), 2.08 – 1.59 (m, 4H). ^{13}C NMR (100 MHz, $CDCl_3$) δ 212.9, 211.8, 147.3, 144.4, 141.4, 140.2, 129.3, 128.9, 128.6, 128.5, 128.2, 127.8, 127.8, 126.2, 125.8, 114.5, 113.8, 73.2, 72.7, 72.5, 72.2, 56.5, 55.1, 49.4, 43.1, 42.4, 40.6, 32.7, 32.5, 32.3, 27.3, 26.5, 23.4. HRMS-ESI (m/z): $[M+H]^+$ calcd for $C_{23}H_{27}O_2$ 335.2011; found 335.2004.

See next page for HPLC result.

HPLC condition: OD-H, hexane/iPrOH = 99.7/0.3, 0.2 ml/min, 20 °C, 210 nm.

Ee = 88%. (the right two peaks were used to calculate ee due to more effective separation.)

Supplementary Figure 25 HPLC spectra for racemic and chiral 3la's deprotection product

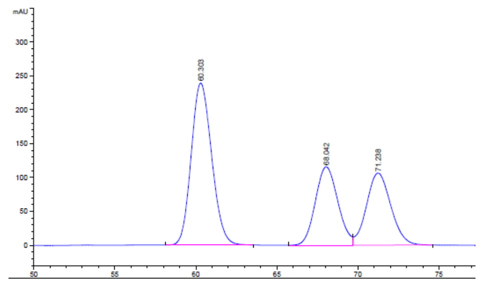

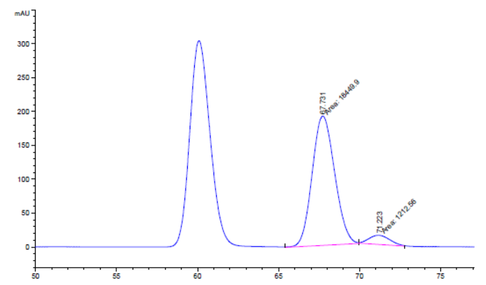

Signal 1: DAD1 B, Sig=210,4 Ref=360,100

Peak #	RetTime [min]	Type	Width [min]	Area [mAU*s]	Area %
1	60.303	BB	1.3556	2.10094e4	48.5403
2	68.042	BV	1.4598	1.11781e4	25.8259
3	71.238	VB	1.5452	1.10949e4	25.6338

Totals : 4.32824e4

Signal 1: DAD1 B, Sig=210,4 Ref=360,100

Peak #	RetTime [min]	Type	Width [min]	Area [mAU*s]	Area %
1	67.731	MM	1.6101	1.84499e4	93.8331
2	71.223	MM	1.4816	1212.56018	6.1669

Totals : 1.96625e4

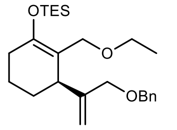

(3ma): ^{1}H NMR (500 MHz, $CDCl_3$) δ 7.35 (m, 4H), 7.29 – 7.27 (m, 1H), 5.23 (s, 1H), 4.87 (s, 1H), 4.56 (d, J = 11.8 Hz, 1H), 4.46 (d, J = 11.8 Hz, 1H), 4.33 (d, J = 10.5 Hz, 1H), 4.08 (d, J = 13.1 Hz, 1H), 3.95 (d, J = 13.1 Hz, 1H), 3.58 (d, J = 10.5 Hz, 1H), 3.41 (m, 1H), 3.32 (m, 1H), 3.14 (m, 1H), 2.21 – 1.99 (m, 2H), 1.67 – 1.54 (m, 4H), 1.13 (t, J = 7.0 Hz, 3H), 1.00 (t, J = 7.9 Hz, 9H), 0.68 (q, J = 7.9 Hz, 6H). ^{13}C NMR (125 MHz, $CDCl_3$) δ 149.6, 147.9, 138.6, 128.4, 127.8, 127.6, 114.0, 113.3, 72.7, 72.0, 65.8, 65.2, 38.0, 30.3, 27.0, 18.9, 15.4, 6.9, 5.7. HRMS-ESI (m/z): $[M+H]^+$ calcd for $C_{25}H_{41}O_3Si$ 417.2825; found 417.2816. Optical Rotation: $[\alpha]_D^{25.0}$ = -40° (c = 0.40, $CHCl_3$).

Ee was determined by the corresponding ketone product by silyl enol ether deprotection method III. Yield: >95%.

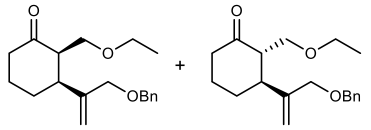

^{1}H NMR (500 MHz, $CDCl_3$) δ 7.38 – 7.27 (m, 5H), 5.26 (s, 0.40H), 5.23 (s, 0.60H), 5.06 (s, 0.60H), 4.84 (s, 0.40H), 4.57 – 4.45 (m, 2H), 4.06 – 3.88 (m, 2H), 3.82 - 3.77 (m, 0.40H), 3.60 – 3.53 (m, 1.23H), 3.41 (m, 2.47H), 2.96 (m, 0.40H), 2.84 (m, 0.40H), 2.59 (m, 0.62H), 2.52 – 2.41 (m, 1.68H), 2.38 – 2.29 (m, 1H), 2.06 – 1.88 (m, 2H), 1.86 – 1.64 (m, 2H), 1.12 (m, 3H). ^{13}C NMR (125 MHz, $CDCl_3$) δ 212.5, 210.6, 147.3, 144.9, 138.3, 138.2, 128.6, 127.8, 127.7, 113.8, 113.1, 73.1, 72.5, 72.3, 72.2, 67.7, 66.6, 66.5, 54.5, 52.9, 45.1, 41.9, 41.8, 40.7, 31.9, 27.0, 25.6, 23.5, 15.2. HRMS-ESI (m/z): $[M+H]^+$ calcd for $C_{19}H_{27}O_3$ 303.1960; found 303.1954.

See next page for HPLC result.

HPLC condition: OD-H, hexane/iPrOH = 99.3/0.7, 1.0 ml/min, 20 °C, 210 nm.

Ee = 97%. (the right two peaks were used to calculate ee due to more effective separation.)

Supplementary Figure 26 HPLC spectra for racemic and chiral 3ma's deprotection product

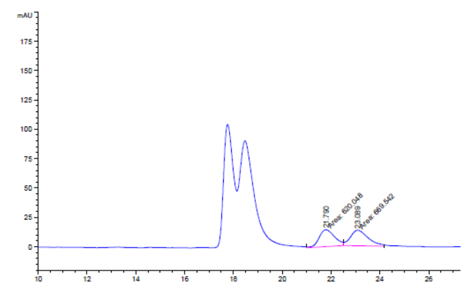

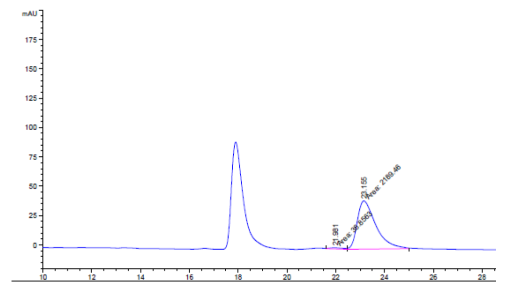

Signal 1: DAD1 B, Sig=210,4 Ref=360,100

Peak #	RetTime [min]	Type	Width [min]	Area [mAU*s]	Area %
1	21.790	MM	0.7273	620.04779	48.0810
2	23.089	MM	0.8467	669.54205	51.9190

Totals : 1289.58984

Signal 1: DAD1 B, Sig=210,4 Ref=360,100

Peak #	RetTime [min]	Type	Width [min]	Area [mAU*s]	Area %
1	21.981	MM	0.6437	38.85627	1.7437
2	23.155	MM	0.8888	2189.46167	98.2563

Totals : 2228.31794

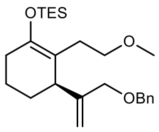

(3na): ^{1}H NMR (400 MHz, $CDCl_3$) δ 7.38 – 7.25 (m, 5H), 5.23 (s, 1H), 4.91 (s, 1H), 4.59 – 4.44 (m, 2H), 4.10 – 4.02 (d, *J* = 12.8 Hz, 1H), 3.95 – 3.88 (d, *J* = 12.8 Hz, 1H), 3.44 – 3.26 (m, 5H), 2.92 (m, 1H), 2.63 (m, 1H), 2.13 – 1.91 (m, 3H), 1.70 – 1.51 (m, 4H), 1.00 (t, *J* = 8.0 Hz, 9H), 0.68 (q, *J* = 8.0 Hz, 6H). ^{13}C NMR (100 MHz, $CDCl_3$) δ 147.7, 147.5, 138.6, 128.5, 127.8, 127.6, 114.0, 112.7, 72.6, 72.1, 71.4, 58.4, 41.1, 30.3, 29.3, 27.4, 19.1, 7.0, 5.9. HRMS-ESI (m/z): $[M+H]^+$ calcd for $C_{25}H_{41}O_3Si$ 417.2825; found 417.2812. Optical Rotation: $[\alpha]_D^{23.9}$ = -69.12° (*c* = 1.14, $CHCl_3$).

HPLC condition: OD-H, hexane/iPrOH = 99.5/0.5, 0.6 ml/min, 20 °C, 210 nm.

Ee = 96%.

Supplementary Figure 27 HPLC spectra for racemic and chiral 3na

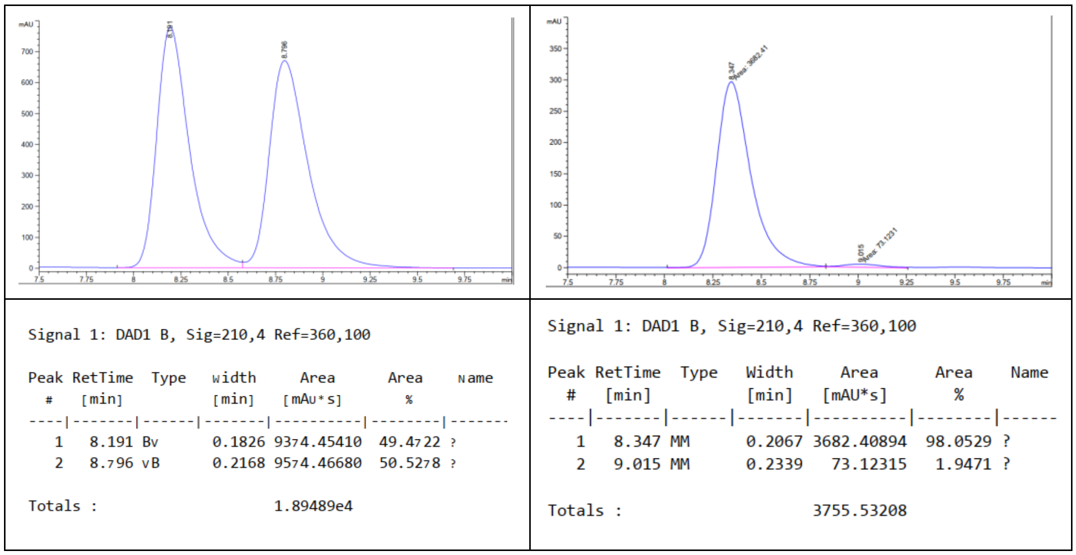

Signal 1: DAD1 B, Sig=210,4 Ref=360,100

Peak #	RetTime [min]	Type	Width [min]	Area [mAU*s]	Area %	Name
1	8.191	BV	0.1826	9374.45410	49.4722	?
2	8.796	VB	0.2168	9574.46680	50.5278	?

Totals : 1.89489e4

Signal 1: DAD1 B, Sig=210,4 Ref=360,100

Peak #	RetTime [min]	Type	Width [min]	Area [mAU*s]	Area %	Name
1	8.347	MM	0.2067	3682.40894	98.0529	?
2	9.015	MM	0.2339	73.12315	1.9471	?

Totals : 3755.53208

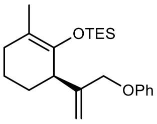

(3gb): ^{1}H NMR (500 MHz, $CDCl_3$) δ 7.28 (m, 2H), 6.93 (m, 3H), 5.27 (s, 1H), 5.05 (s, 1H), 4.49 (s, 2H), 2.95 (m, 1H), 1.97 (t, J = 6.4 Hz, 2H), 1.81 – 1.67 (m, 2H), 1.64 – 1.61 (s, 3H), 1.59 (m, 1H), 1.51 – 1.43 (m, 1H), 0.96 (t, J = 8.0 Hz, 9H), 0.66 (q, J = 8.0 Hz, 6H). ^{13}C NMR (125 MHz, $CDCl_3$) δ 159.1, 145.4, 143.0, 129.5, 120.7, 114.8, 114.4, 114.0, 69.5, 44.4, 30.5, 29.5, 19.5, 16.9, 7.0, 5.7. HRMS-ESI (m/z): $[M+H]^+$ calcd for $C_{22}H_{35}O_2Si$ 359.2406; found 359.2399. Optical Rotation: $[\alpha]_D^{23.0}$ = -34.18° (c = 3.22, $CHCl_3$).

HPLC condition: OD-H+AS-3, hexane/iPrOH = 100/0, 0.4 ml/min, 20 °C, 210 nm.

Ee = 96%.

Supplementary Figure 28 HPLC spectra for racemic and chiral 3gb

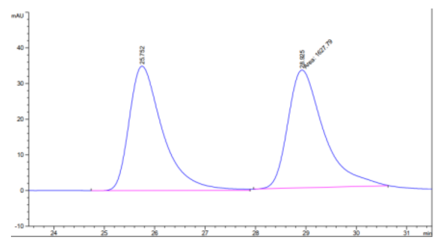

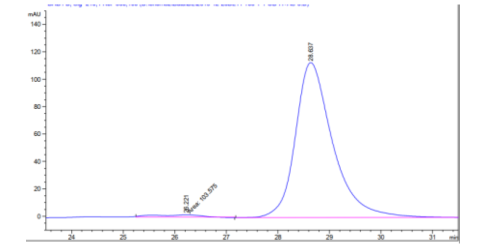

Signal 1: DAD1 B, Sig=210,4 Ref=360,100

Peak #	RetTime [min]	Type	Width [min]	Area [mAU*s]	Area %	Name
1	25.752	BV	0.6940	1609.54358	49.7182	?
2	28.925	MM	0.8210	1627.79114	50.2818	?
Totals :				3237.33472		

Signal 1: DAD1 B, Sig=210,4 Ref=360,100

Peak #	RetTime [min]	Type	Width [min]	Area [mAU*s]	Area %	Name
1	26.221	MM	1.0163	103.57502	1.7874	?
2	28.637	BB	0.7513	5691.22314	98.2126	?
Totals :				5794.79816		

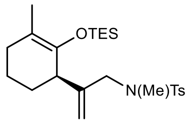

(3gc): ^{1}H NMR (500 MHz, $CDCl_3$) δ 7.68 (d, J = 8.2 Hz, 2H), 7.32 (d, J = 8.2 Hz, 2H), 5.08 (s, 1H), 5.02 (s, 1H), 3.77 (d, J = 14.6 Hz, 1H), 3.27 (d, J = 14.6 Hz, 1H), 2.84 (m, 1H), 2.64 (s, 3H), 2.43 (s, 3H), 2.03 – 1.86 (m, 2H), 1.75 – 1.62 (m, 2H), 1.60 (s, 3H), 1.55-1.40 (m, 2H), 0.95 (t, J = 7.9 Hz, 9H), 0.63 (q, J = 7.9 Hz, 6H). ^{13}C NMR (125 MHz, $CDCl_3$) δ 144.0, 143.4, 143.1, 134.6, 129.8, 127.6, 115.7, 114.4, 54.0, 44.3, 34.6, 30.4, 28.7, 21.6, 19.0, 16.9, 7.0, 5.7. HRMS-ESI (m/z): $[M+H]^+$ calcd for $C_{24}H_{40}NO_3SSi$ 450.2498; found 450.2491**.** Optical Rotation: $[\alpha]_D^{23.0}$ = -45.3° (c = 1.56, $CHCl_3$).

HPLC condition: OD-H+OD-H, hexane/iPrOH = 99.5/0.5, 0.5 ml/min, 20 °C, 236 nm.

Ee = 97%.

Supplementary Figure 29 HPLC spectra for racemic and chiral 3gc

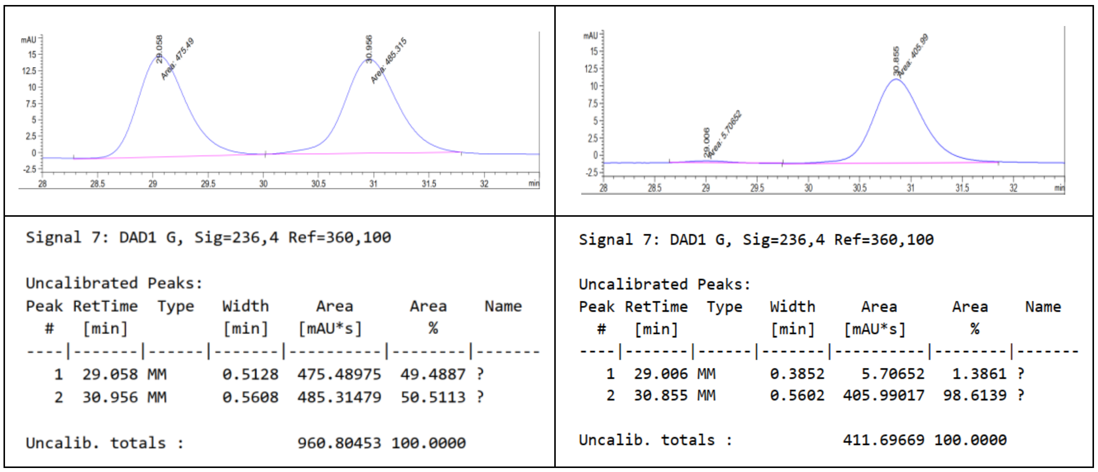

Signal 7: DAD1 G, Sig=236,4 Ref=360,100

Uncalibrated Peaks:

Peak #	RetTime [min]	Type	Width [min]	Area [mAU*s]	Area %	Name
1	29.058	MM	0.5128	475.48975	49.4887	?
2	30.956	MM	0.5608	485.31479	50.5113	?

Uncalib. totals : 960.80453 100.0000

Signal 7: DAD1 G, Sig=236,4 Ref=360,100

Uncalibrated Peaks:

Peak #	RetTime [min]	Type	Width [min]	Area [mAU*s]	Area %	Name
1	29.006	MM	0.3852	5.70652	1.3861	?
2	30.855	MM	0.5602	405.99017	98.6139	?

Uncalib. totals : 411.69669 100.0000

Set 2 Heterocyclic

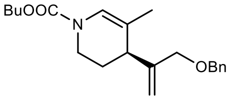

(3oa): ^{1}H NMR (500 MHz, $CDCl_3$) δ 7.39 – 7.32 (m, 4H), 7.31 – 7.27 (m, 1H), 6.79 (d, J = 65.3 Hz, 1H), 5.26 (s, 1H), 4.93 (d, J = 7.1 Hz, 1H), 4.56 (d, J = 11.9 Hz, 1H), 4.46 (d, J = 11.9 Hz, 1H), 4.14 (m, 2H), 4.09 (d, J = 12.7 Hz, 1H), 3.93 (d, J = 12.7 Hz, 1H), 3.87 – 3.69 (m, 1H), 3.21 – 3.12 (m, 1H), 2.79 (m, 1H), 1.90 –1.72 (m, 2H), 1.69-1.58 (m, 5H), 1.41 (m, 2H), 1.04 – 0.92 (m, 3H). ^{13}C NMR (125 MHz, $CDCl_3$) δ 153.8, 153.4, 146.2, 146.1, 138.3, 128.5, 127.8, 122.1, 121.7, 115.1, 115.0, 114.8, 114.5, 72.3, 72.1, 65.8, 65.7, 39.4, 39.3, 37.7, 37.5, 31.7, 31.2, 31.1, 29.8, 25.9, 22.8, 19.8, 19.7, 19.3, 14.3, 13.9. HRMS-ESI (m/z): $[M+H]^+$ calcd for $C_{21}H_{30}NO_3$ 344.2226; found 344.2217**.** Optical Rotation: $[\alpha]_D^{23.0}$ = -125° (c = 0.89, $CHCl_3$).

HPLC condition: AS-3, hexane/iPrOH = 95.0/5.0, 1.0 ml/min, 20 °C, 210 nm.

Ee = 98%.

Supplementary Figure 30 HPLC spectra for racemic and chiral 3oa

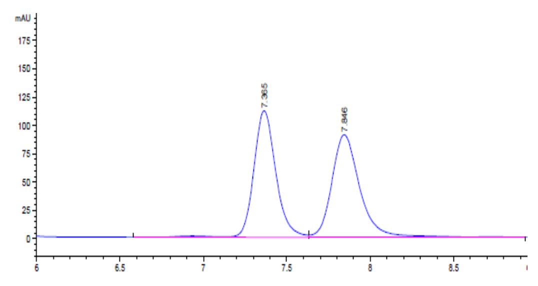

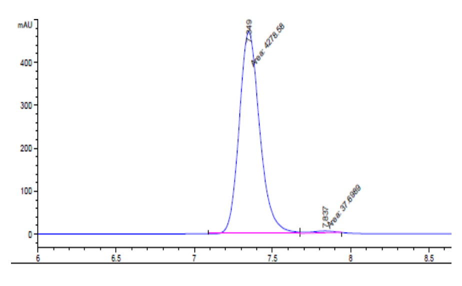

Signal 1: DAD1 B, Sig=210,4 Ref=360,100

Uncalibrated Peaks:

Peak #	RetTime [min]	Type	Width [min]	Area [mAU*s]	Area %
1	7.365	VV R	0.1421	1029.42517	49.3578
2	7.846	VV R	0.1803	1056.21521	50.6422

Uncalib. totals : 2085.64038 100.0000

Signal 1: DAD1 B, Sig=210,4 Ref=360,100

Uncalibrated Peaks:

Peak #	RetTime [min]	Type	Width [min]	Area [mAU*s]	Area %
1	7.349	MM	0.1514	4278.58154	99.1266
2	7.837	MM	0.1594	37.69886	0.8734

Uncalib. totals : 4316.28040 100.0000

Set 3 1,4-Disubstitutions

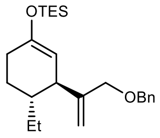

(3pa): ^{1}H NMR (500 MHz, $CDCl_3$) δ 7.37 – 7.32 (m, 4H), 7.28 (m, 1H), 5.19 (s, 1H), 5.00 (s, 1H), 4.64 (m, 1H), 4.57 – 4.47 (dd, J = 27.0, 11.5 Hz, 2H), 4.02 – 3.91 (dd, J = 29.5, 13.0 Hz, 2H), 2.69 (m, 1H), 2.01 (m, 2H), 1.79 (m, 1H), 1.49 (m, 1H), 1.33 (m, 2H), 1.23 – 1.13 (m, 1H), 0.96 (t, J = 8.0 Hz, 9H), 0.88 (t, J = 7.4 Hz, 3H), 0.65 (q, J = 8.0 Hz, 6H). ^{13}C NMR (125 MHz, $CDCl_3$) δ 151.2, 149.0, 138.6, 128.5, 127.7, 127.6, 113.0, 106.2, 72.4, 71.6, 45.2, 37.7, 28.4, 25.6, 25.1, 11.8, 6.9, 5.2. HRMS-ESI (m/z): $[M+H]^+$ calcd for $C_{24}H_{39}O_2Si$ 387.2719; found 387.2709**.** Optical Rotation: $[\alpha]_D^{23.0}$ = -2.9° (c = 1.36, $CHCl_3$).

HPLC condition: OD-H, hexane/iPrOH = 100/0, 0.7 ml/min, 20 °C, 210 nm.

Ee = 84%.

Supplementary Figure 31 HPLC spectra for racemic and chiral 3pa

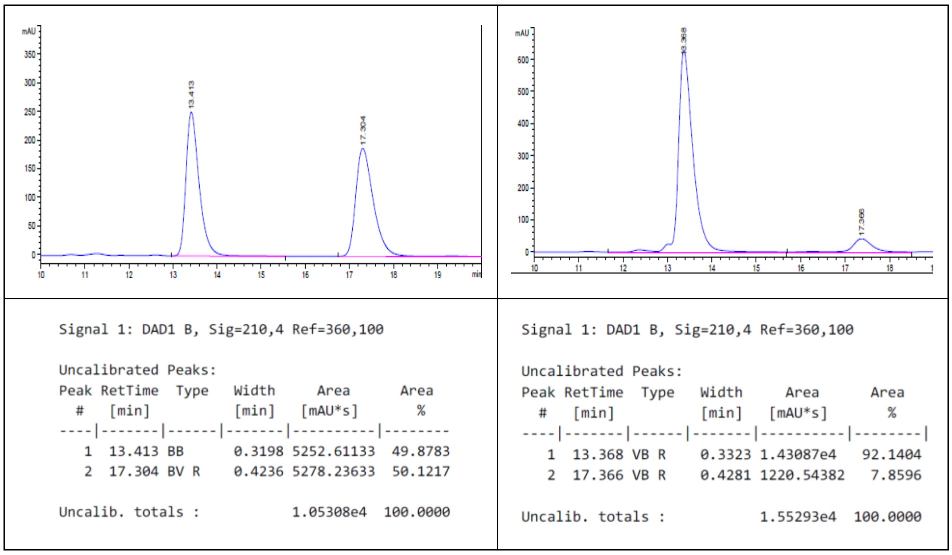

Signal 1: DAD1 B, Sig=210,4 Ref=360,100

Uncalibrated Peaks:

Peak #	RetTime [min]	Type	Width [min]	Area [mAU*s]	Area %
1	13.413	BB	0.3198	5252.61133	49.8783
2	17.304	BV R	0.4236	5278.23633	50.1217

Uncalib. totals : 1.05308e4 100.0000

Signal 1: DAD1 B, Sig=210,4 Ref=360,100

Uncalibrated Peaks:

Peak #	RetTime [min]	Type	Width [min]	Area [mAU*s]	Area %
1	13.368	VB R	0.3323	1.43087e4	92.1404
2	17.366	VB R	0.4281	1220.54382	7.8596

Uncalib. totals : 1.55293e4 100.0000

Set 4 Scope of Olefin Donors

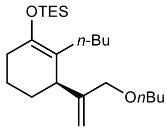

(3jd):^{1}H NMR (500 MHz, CDCl$_3$) δ 5.14 (s, 1H), 4.86 (s, 1H), 4.00 (d, J = 13.0 Hz, 1H), 3.82 (d, J = 13.0 Hz, 1H), 3.45 (m, 1H), 3.35 (m, 1H), 2.90 (m, 1H), 2.47 – 2.33 (m, 1H), 2.15 – 1.93 (m, 2H), 1.67 –1.20 (m, 13H), 1.04 – 0.98 (m, 9H), 0.92 (t, J = 7.4 Hz, 3H), 0.86 (m, 3H), 0.67 (m, 6H). ^{13}C NMR (125 MHz, CDCl$_3$) δ 148.2, 145.6, 116.8, 113.3, 77.4, 77.1, 76.9, 40.1, 32.1, 30.5, 30.4, 28.4, 27.50, 23.2, 19.6, 19.3, 14.3, 14.1, 7.0, 5.9. HRMS-ESI (m/z): [M+H]$^+$ calcd for $C_{23}H_{45}O_2Si$ 381.3189 ; found 381.3178. Optical Rotation: $[\alpha]_D^{25.0}$ = -31° (c = 0.40, CHCl$_3$).

Ee was determined by the corresponding ketone product by silyl enol ether deprotection method III. Yield: >95%.

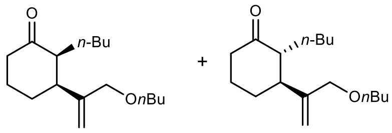

^{1}H NMR (400 MHz, CDCl$_3$) δ 5.21 (s, 0.57H), 5.15 (m, 0.43H), 4.96 (s, 0.43H), 4.87 (s, 0.57H), 3.99 – 3.80 (m, 2H), 3.46 – 3.35 (m, 2H), 2.72 (m, 0.57H), 2.51 – 0.86 (m, 23.43H). ^{13}C NMR (100 MHz, CDCl$_3$) δ 214.7, 213.0, 147.8, 145.6, 113.6, 112.8, 54.1, 53.1, 48.9, 43.7, 42.1, 39.1, 32.0, 31.9, 31.7, 30.3, 29.7, 27.2, 26.1, 25.5, 24.7, 23.1, 22.8, 19.6, 19.5, 14.2, 14.1. HRMS-ESI (m/z): [M+H]$^+$ calcd for $C_{17}H_{31}O_2$ 267.2324 ; found 267.2317.

HPLC condition: OD-H, hexane/iPrOH = 99.8/0.2, 0.3 ml/min, 20 °C, 210 nm.

Ee = 93%. (the right two peaks were used to calculate ee due to more effective separation.)

Supplementary Figure 32 HPLC spectra for racemic and chiral 3jd's deprotection product

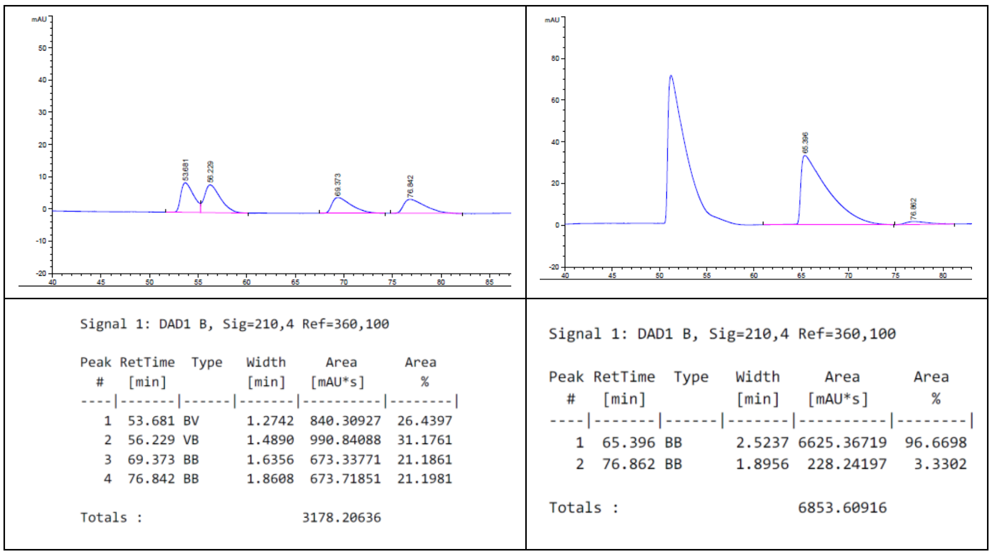

Signal 1: DAD1 B, Sig=210,4 Ref=360,100

Peak #	RetTime [min]	Type	Width [min]	Area [mAU*s]	Area %
1	53.681	BV	1.2742	840.30927	26.4397
2	56.229	VB	1.4890	990.84088	31.1761
3	69.373	BB	1.6356	673.33771	21.1861
4	76.842	BB	1.8608	673.71851	21.1981
Totals :				3178.20636	

Signal 1: DAD1 B, Sig=210,4 Ref=360,100

Peak #	RetTime [min]	Type	Width [min]	Area [mAU*s]	Area %
1	65.396	BB	2.5237	6625.36719	96.6698
2	76.862	BB	1.8956	228.24197	3.3302
Totals :				6853.60916	

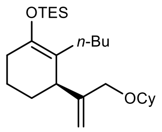

(3je): ^{1}H NMR (500 MHz, $CDCl_3$) δ 5.15 (s, 1H), 4.84 (s, 1H), 3.94 (dd, J = 24.5, 13.0 Hz, 2H), 3.35 – 3.18 (m, 1H), 2.91 (m, 1H), 2.41 – 2.33 (m, 1H), 2.15 – 2.06 (m, 1H), 2.03 – 1.85 (m, 3H), 1.77 – 1.70 (m, 2H), 1.68 – 1.59 (m, 5H), 1.37 – 1.19 (m, 10H), 1.00 (t, J = 7.9 Hz, 9H), 0.87 (t, J = 6.9 Hz, 3H), 0.67 (q, J = 7.9 Hz, 6H). ^{13}C NMR (100 MHz, $CDCl_3$) δ 148.8, 145.6 116.9, 113.0, 69.8, 40.2, 32.7, 31.9, 30.5, 30.4, 28.4, 27.6, 26.0, 24.3, 23.2, 19.3, 14.3, 7.0, 5.9. HRMS-ESI (m/z): $[M+H]^+$ calcd for $C_{26}H_{49}O_2Si$ 421.3502; found 421.3496. Optical Rotation: $[\alpha]_D^{25.0}$ = -21.3° (c = 0.40, $CHCl_3$).

HPLC condition: OD-H, hexane/iPrOH = 100/0, 0.3 ml/min, 20 °C, 210 nm.

Ee = 88%.

Supplementary Figure 33 HPLC spectra for racemic and chiral 3je

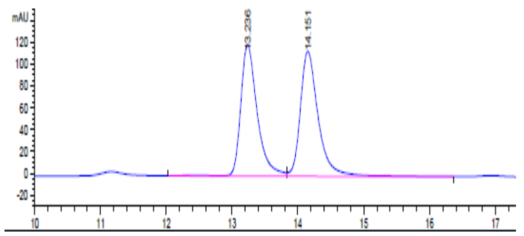

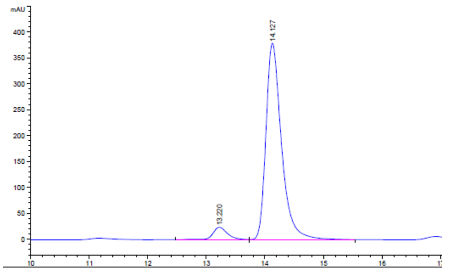

Signal 2: DAD1 B, Sig=210,4 Ref=360,100

Peak #	RetTime [min]	Type	Width [min]	Area [mAU*s]	Area %
1	13.236	VV R	0.2595	2091.52930	49.0703
2	14.151	VB	0.2837	2170.78516	50.9297
Totals :				4262.31445	

Signal 1: DAD1 B, Sig=210,4 Ref=360,100

Peak #	RetTime [min]	Type	Width [min]	Area [mAU*s]	Area %
1	13.220	VV R	0.2542	429.05405	5.7818
2	14.127	VB	0.2790	6991.71777	94.2182
Totals :				7420.77182	

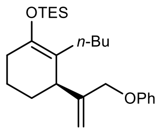

(3jb): ^{1}H NMR (400 MHz, $CDCl_3$) δ 7.31 – 7.25 (m, 2H), 6.97 – 6.89 (m, 3H), 5.29 (s, 1H), 4.98 (s, 1H), 4.63 – 4.38 (m, 2H), 3.00 (m, 1H), 2.45 – 2.29 (m, 1H), 2.08 (m, 2H), 1.77 – 1.15 (m, 9H), 0.99 (t, J = 7.9 Hz, 9H), 0.86 (t, J = 7.0 Hz, 3H), 0.67 (q, J = 7.9 Hz, 6H). ^{13}C NMR (100 MHz, $CDCl_3$) δ 159.0, 146.8, 145.9, 129.5, 120.8, 116.5, 114.9, 114.2, 70.1, 40.4, 30.4, 30.4, 28.2, 27.8, 23.2, 19.4, 14.2, 7.0, 5.9. HRMS-ESI (m/z): $[M+H]^+$ calcd for $C_{25}H_{41}O_2Si$ 401.2876; found 401.2866. Optical Rotation: $[\alpha]_D^{25.0}$ = -56.0° (c = 0.40, $CHCl_3$).

HPLC condition: OD-H, hexane/iPrOH = 100/0, 0.3 ml/min, 20 °C, 210 nm.

Ee = 89%.

Supplementary Figure 34 HPLC spectra for racemic and chiral 3jb

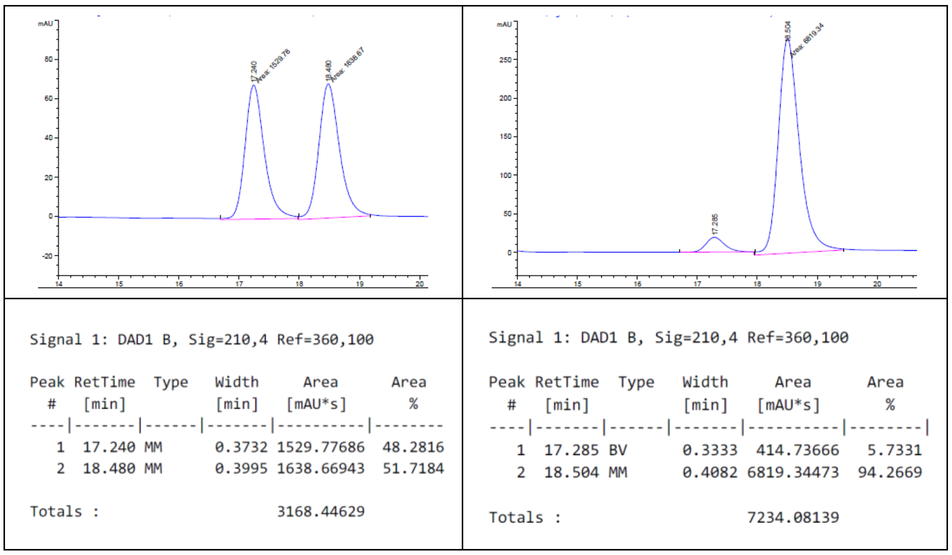

Signal 1: DAD1 B, Sig=210,4 Ref=360,100

Peak #	RetTime [min]	Type	Width [min]	Area [mAU*s]	Area %
1	17.240	MM	0.3732	1529.77686	48.2816
2	18.480	MM	0.3995	1638.66943	51.7184
Totals :				3168.44629	

Signal 1: DAD1 B, Sig=210,4 Ref=360,100

Peak #	RetTime [min]	Type	Width [min]	Area [mAU*s]	Area %
1	17.285	BV	0.3333	414.73666	5.7331
2	18.504	MM	0.4082	6819.34473	94.2669
Totals :				7234.08139	

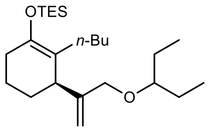

(3jf): ^{1}H NMR (400 MHz, CDCl$_3$) δ 5.18 (s, 1H), 4.84 (s, 1H), 3.90 (dd, J = 22.8, 12.8 Hz, 2H), 3.19 (m, 1H), 2.91 (m, 1H), 2.43 – 2.30 (m, 1H), 2.14 – 1.94 (m, 2H), 1.69 – 1.18 (m, 13H), 1.00 (t, J = 7.9 Hz, 9H), 0.89 (t, J = 8.0 Hz, 9H), 0.67 (q, J = 8.0 Hz, 6H). ^{13}C NMR (100 MHz, CDCl$_3$) δ 148.9, 145.5, 117.0, 112.8, 81.5, 70.7, 40.7, 30.5, 30.4, 28.4, 27.7, 26.0, 25.5, 23.2, 19.5, 14.3, 9.8, 9.6, 7.0, 5.9. HRMS-ESI (m/z): [M+H]$^+$ calcd for $C_{22}H_{47}O_2Si$ 395.3345; found 395.3343. Optical Rotation: $[\alpha]_D^{25.0}$ = -13.6° (c = 0.40, CHCl$_3$).

Ee was determined by the corresponding ketone product by silyl enol ether deprotection method III. Yield: >95%.

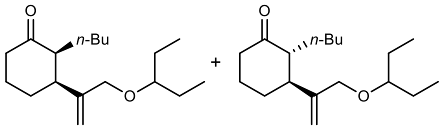

^{1}H NMR (400 MHz, CDCl$_3$) δ 5.23 (s, 0.67H), 5.19 (m, 0.33H), 4.93 (s, 0.33H), 4.86 (s, 0.67H), 3.88 (m, 2H), 3.16 (m, 1H), 2.74 (m, 0.67H), 2.50 – 1.06 (m, 17.33H), 0.92 – 0.83 (m, 9H). ^{13}C NMR (100 MHz, CDCl$_3$) δ 214.7, 213.2, 148.4, 146.2, 113.1, 112.3, 82.0, 81.9, 71.2, 70.8, 54.3, 53.0, 48.6, 43.8, 42.1, 38.9, 31.6, 30.3, 29.7, 27.3, 26.1, 26.0, 25.9, 25.8, 25.7, 25.2, 24.9, 23.1, 22.8, 14.1, 9.8, 9.7, 9.6. HRMS-ESI (m/z): [M+H]$^+$ calcd for $C_{18}H_{33}O_2$ 281.2481; found 281.2474. HPLC condition: OD-H, hexane/iPrOH = 99.8/0.2, 0.3 ml/min, 20 °C, 210 nm.

Ee = 90%. (the left two peaks were used to calculate ee due to more effective separation)

Supplementary Figure 35 HPLC spectra for racemic and chiral 3jf's deprotection product

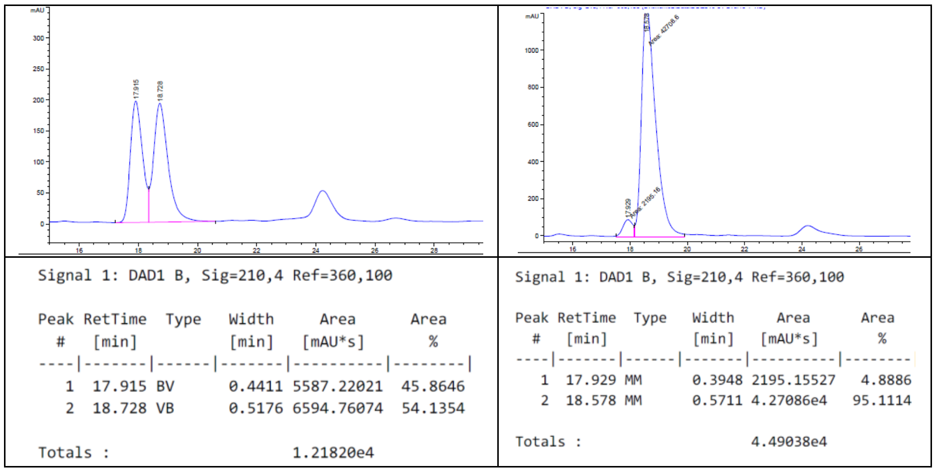

Signal 1: DAD1 B, Sig=210,4 Ref=360,100

Peak #	RetTime [min]	Type	Width [min]	Area [mAU*s]	Area %
1	17.915	BV	0.4411	5587.22021	45.8646
2	18.728	VB	0.5176	6594.76074	54.1354

Totals : 1.21820e4

Signal 1: DAD1 B, Sig=210,4 Ref=360,100

Peak #	RetTime [min]	Type	Width [min]	Area [mAU*s]	Area %
1	17.929	MM	0.3948	2195.15527	4.8886
2	18.578	MM	0.5711	4.27086e4	95.1114

Totals : 4.49038e4

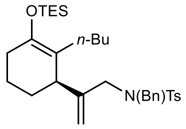

(3jh): ^{1}H NMR (400 MHz, $CDCl_3$) δ 7.72 (d, J = 8.3 Hz, 2H), 7.30 – 7.24 (m, 5H), 7.17 (dd, J = 7.0, 2.5 Hz, 2H), 4.83 (s, 1H), 4.81 (s, 1H), 4.59 (d, J = 15.2 Hz, 1H), 4.21 (d, J = 15.2 Hz, 1H), 3.95 (d, J = 16.2 Hz, 1H), 3.53 (d, J = 16.2 Hz, 1H), 2.55 (m, 1H), 2.42 (s, 3H), 2.30 (m, 1H), 2.10 – 1.86 (m, 2H), 1.50 – 1.10 (m, 9H), 0.97 (t, J = 7.9 Hz, 9H), 0.88 (t, J = 7.1 Hz, 3H), 0.63 (q, J = 7.9 Hz, 6H). ^{13}C NMR (100 MHz, $CDCl_3$) δ 145.9, 145.1, 143.2, 138.2, 135.9, 129.7, 128.8, 128.6, 127.8, 127.3, 116.2, 115.7, 50.2, 50.0, 40.0, 30.5, 30.2, 28.5, 27.0, 23.2, 21.6, 19.0, 14.3, 7.0, 5.9. HRMS-ESI (m/z): $[M+H]^+$ calcd for $C_{33}H_{50}NO_3SSi$ 568.3281; found 568.3270. Optical Rotation: $[\alpha]_D^{25.0}$ = -50.0° (c = 1.20, $CHCl_3$).

HPLC condition: OD-H, hexane/iPrOH = 99.3/0.7, 0.5 ml/min, 20 °C, 210 nm.

Ee = 45%.

Supplementary Figure 36 HPLC spectra for racemic and chiral 3jh

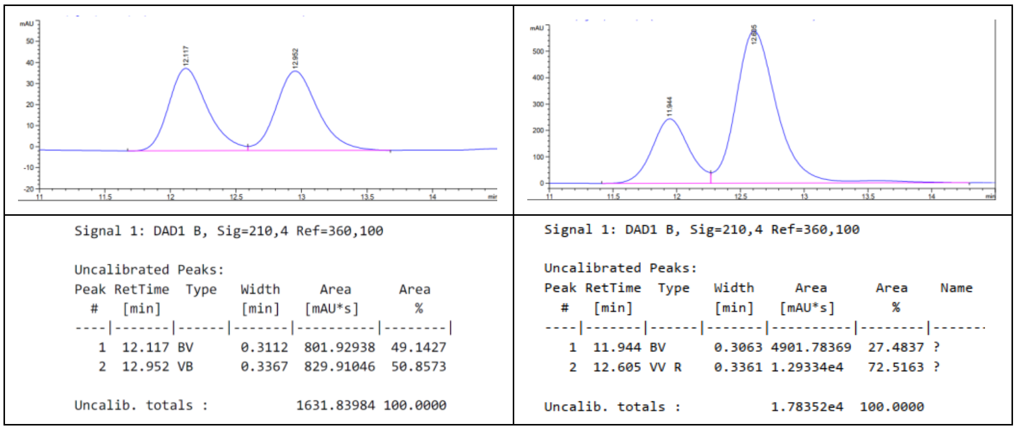

Signal 1: DAD1 B, Sig=210,4 Ref=360,100

Uncalibrated Peaks:

Peak #	RetTime [min]	Type	Width [min]	Area [mAU*s]	Area %
1	12.117	BV	0.3112	801.92938	49.1427
2	12.952	VB	0.3367	829.91046	50.8573

Uncalib. totals : 1631.83984 100.0000

Signal 1: DAD1 B, Sig=210,4 Ref=360,100

Uncalibrated Peaks:

Peak #	RetTime [min]	Type	Width [min]	Area [mAU*s]	Area %	Name
1	11.944	BV	0.3063	4901.78369	27.4837	?
2	12.605	VV R	0.3361	1.29334e4	72.5163	?

Uncalib. totals : 1.78352e4 100.0000

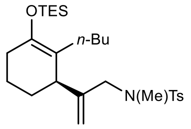

(3jc): ^{1}H NMR (400 MHz, $CDCl_3$) δ 7.67 (dd, J = 14.1, 7.5 Hz, 2H), 7.32 (d, J = 7.3 Hz, 2H), 5.05 (s, 1H), 4.90 (s, 1H), 3.99 (d, J = 13.9 Hz, 1H), 3.06 (d, J = 13.9 Hz, 1H), 2.97 (s, 1H), 2.61 (s, 3H), 2.43 (s, 3H), 2.40 – 2.31 (m, 1H), 2.17 – 2.04 (m, 1H), 2.04 - 1.90 (m, 1H), 1.69 –1.20 (m, 9H), 0.99 (t, J = 7.8 Hz, 9H), 0.90 – 0.85 (m, 3H), 0.66 (q, J = 7.8 Hz, 6H). ^{13}C NMR (100 MHz, $CDCl_3$) δ 146.0, 145.6, 143.4, 134.5, 129.8, 127.6, 116.4, 116.3, 54.7, 39.7, 34.1, 30.4, 30.3, 28.5, 26.9, 23.2, 21.6, 19.0, 14.2, 7.0, 5.9. HRMS-ESI (m/z): HRMS-ESI (m/z): $[M+H]^+$ calcd for $C_{27}H_{46}NO_3SSi$ 492.2968; found 492.2956. Optical Rotation: $[\alpha]_D^{23.2}$ = -51.3° (c = 0.88, $CHCl_3$).

HPLC condition: OD-H, hexane/iPrOH = 99.0/1.0, 1.0 ml/min, 20 °C, 210 nm.

Ee = 86%.

Supplementary Figure 37 HPLC spectra for racemic and chiral 3jc

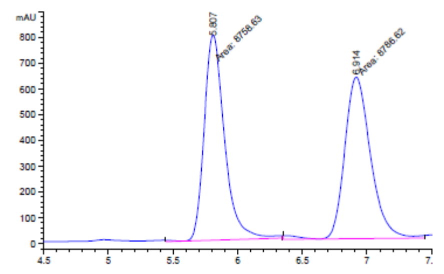

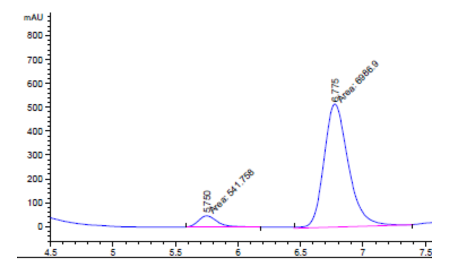

Uncalibrated Peaks:

Peak #	RetTime [min]	Type	Width [min]	Area [mAU*s]	Area %
1	5.807	MM	0.1828	8758.62891	49.9202
2	6.914	MM	0.2329	8786.62109	50.0798
Uncalib. totals :				1.75453e4	100.0000

Uncalibrated Peaks:

Peak #	RetTime [min]	Type	Width [min]	Area [mAU*s]	Area %
1	5.750	MM	0.1862	541.75848	7.1959
2	6.775	MM	0.2258	6986.90137	92.8041
Uncalib. totals :				7528.65985	100.0000

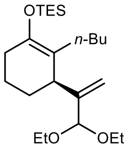

(3ji): ^{1}H NMR (500 MHz, $CDCl_3$) δ 5.31 (s, 1H), 4.95 (s, 1H), 4.76 (s, 1H), 3.61 (m, 2H), 3.51 – 3.39 (m, 2H), 3.05 (m, 1H), 2.44 – 2.30 (m, 1H), 2.14 – 1.94 (m, 2H), 1.67 – 1.17 (m, 15H), 1.00 (t, J = 7.9 Hz, 9H), 0.88 (q, J = 6.7 Hz, 3H), 0.67 (q, J = 7.9 Hz, 6H). ^{13}C NMR (125 MHz, $CDCl_3$) δ 147.9, 145.7, 117.3, 115.1, 103.6, 62.7, 60.7, 37.9, 30.4, 30.3, 28.4, 27.8, 23.3, 18.6, 15.3, 15.2, 14.3, 7.0, 5.9. HRMS-ESI (m/z): $[M+H]^+$ calcd for $C_{23}H_{45}O_3Si$ 397.3138; found 397.3126.

Optical Rotation: $[\alpha]_D^{25.0}$ = -30° (c = 1.56, $CHCl_3$).

Ee was determined by the corresponding ketone product by silyl enol ether deprotection method III. Yield: >95%.

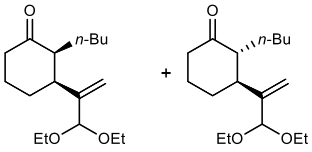

^{1}H NMR (500 MHz, $CDCl_3$) δ 5.36 (s, 0.80H), 5.32 (s, 0.20H), 5.07 (s, 0.20H), 4.91 (s, 0.80H), 4.70 (s, 0.20H), 4.63 (s, 0.80H), 3.66 – 3.39 (m, 4H), 2.91 (m, 0.80H), 2.54 – 1.10 (m, 19.20H), 0.85 (t, J = 7.2 Hz, 3H). ^{13}C NMR (125 MHz, $CDCl_3$) δ 214.8, 213.4, 148.3, 145.2, 115.1, 113.7, 104.1, 104.0, 62.6, 62.3, 62.2, 62.0, 54.9, 53.5, 45.5, 42.1, 39.5, 32.9, 30.3, 29.8, 29.6, 27.2, 26.7, 26.0, 26.0, 24.2, 23.1, 22.8, 15.2, 14.1. HRMS-ESI (m/z): $[M+Na]^+$ calcd for $C_{17}H_{30}NaO_3$ 305.2093; found 305.2085.

HPLC condition: OD-H+OD-H, hexane/iPrOH = 99.6/0.4, 0.5 ml/min, 20 °C, 210 nm.

Ee = 96%. (the right two peaks were used to calculate ee dur to more effective separation)

Supplementary Figure 38 HPLC spectra for racemic and chiral 3ji's deprotection product

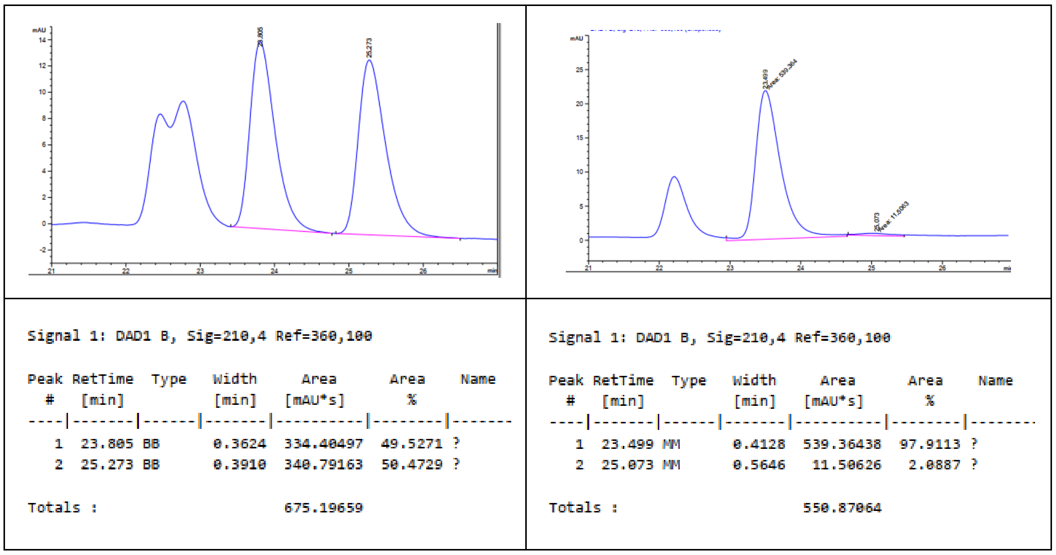

Signal 1: DAD1 B, Sig=210,4 Ref=360,100

Peak #	RetTime [min]	Type	Width [min]	Area [mAU*s]	Area %	Name
1	23.805	BB	0.3624	334.40497	49.5271	?
2	25.273	BB	0.3910	340.79163	50.4729	?

Totals : 675.19659

Signal 1: DAD1 B, Sig=210,4 Ref=360,100

Peak #	RetTime [min]	Type	Width [min]	Area [mAU*s]	Area %	Name
1	23.499	MM	0.4128	539.36438	97.9113	?
2	25.073	MM	0.5646	11.50626	2.0887	?

Totals : 550.87064

Set 5 Chemoselectivity

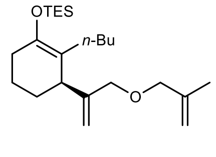

(3jj): ^{1}H NMR (500 MHz, $CDCl_3$) δ 5.17 (s, 1H), 4.97 (s, 1H), 4.88 (m, 2H), 3.99 (d, J = 12.9 Hz, 1H), 3.91 (d, J = 12.9 Hz, 1H), 3.83 (d, J = 13.1 Hz, 2H), 2.92 (m, 1H), 2.41 – 2.32 (m, 1H), 2.14 – 1.96 (m, 2H), 1.75 (s, 3H), 1.67 – 1.49 (m, 5H), 1.37 – 1.21 (m, 4H), 1.00 (t, J = 7.9 Hz, 9H), 0.87 (t, J = 7.1 Hz, 3H), 0.67 (q, J = 7.9 Hz, 6H). ^{13}C NMR (125 MHz, $CDCl_3$) δ 148.0, 145.6, 142.5, 116.8, 113.5, 111.9, 73.9, 72.3, 40.3, 30.5, 30.4, 28.4, 27.6, 23.2, 19.7, 19.3, 14.3, 7.0, 5.9. HRMS-ESI (m/z): $[M+H]^+$ calcd for $C_{23}H_{43}O_2Si$ 397.3138; found 397.3126. Optical Rotation: $[\alpha]_D^{25.0}$ = -15.7° (c = 1.30, $CHCl_3$).

Ee was determined by the corresponding ketone product by silyl enol ether deprotection method III. Yield: >95%.

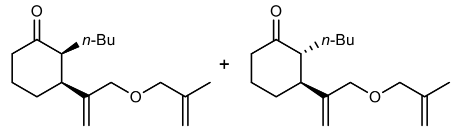

^{1}H NMR (500 MHz, $CDCl_3$) δ 5.23 (s, 0.67H), 5.18 (m, 0.33H), 4.99 – 4.87 (m, 3H), 3.99 – 3.80 (m, 4H), 2.73 (m, 0.67H), 2.50 – 1.07 (m, 16.33H), 0.86 (m, 3H). ^{13}C NMR (125 MHz, $CDCl_3$) δ 214.6, 212.9, 147.5, 145.3, 142.2, 113.7, 112.9, 112.3, 112.2, 74.3, 72.5, 71.9, 54.1, 53.1, 48.6, 43.8, 42.1, 39.1, 31.7, 30.4, 30.2, 29.7, 27.2, 26.1, 26.0, 25.5, 24.6, 23.1, 22.8, 19.6, 14.1. HRMS-ESI (m/z): $[M+H]^+$ calcd for $C_{17}H_{29}O_2$ 265.2168; found 265.2163.

See next page for HPLC result.

HPLC condition: AS-3, hexane/iPrOH = 99.3/0.7, 1.0 ml/min, 20 ˚C, 210 nm.

Ee = 89%. (the right two peaks were used to calculate ee due to more effective separation)

Supplementary Figure 39 HPLC spectra for racemic and chiral 3jj's deprotection product

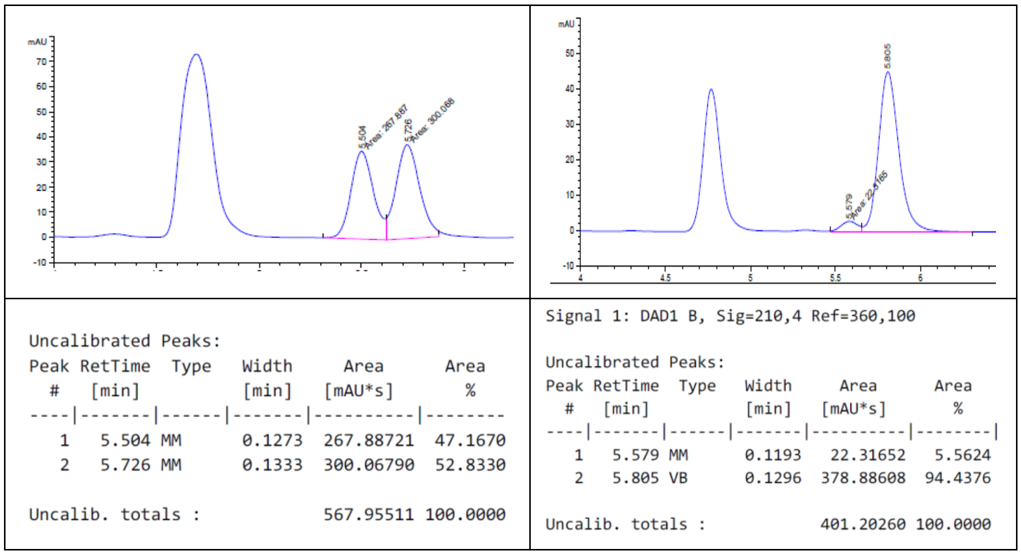

Uncalibrated Peaks:

Peak #	RetTime [min]	Type	Width [min]	Area [mAU*s]	Area %
1	5.504	MM	0.1273	267.88721	47.1670
2	5.726	MM	0.1333	300.06790	52.8330

Uncalib. totals : 567.95511 100.0000

Signal 1: DAD1 B, Sig=210,4 Ref=360,100

Uncalibrated Peaks:

Peak #	RetTime [min]	Type	Width [min]	Area [mAU*s]	Area %
1	5.579	MM	0.1193	22.31652	5.5624
2	5.805	VB	0.1296	378.88608	94.4376

Uncalib. totals : 401.20260 100.0000

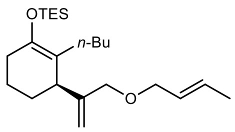

(3jk): ^{1}H NMR (500 MHz, $CDCl_3$) δ 5.71 (m, 1H), 5.59 (m, 1H), 5.15 (s, 1H), 4.87 (s, 1H), 3.98 (d, J = 13.0 Hz, 1H), 3.93 (m, 1H), 3.87 – 3.81 (m, 2H), 2.90 (m, 1H), 2.37 (m, 1H), 2.16 – 1.94 (m, 2H), 1.71 (d, J = 6.4 Hz, 3H), 1.67 – 1.20 (m, 9H), 1.00 (t, J = 8.0 Hz, 9H), 0.87 (t, J = 7.0 Hz, 3H), 0.67 (q, J = 8.0 Hz, 6H). ^{13}C NMR (125 MHz, $CDCl_3$) δ 148.0, 145.6, 129.4, 127.8, 116.8, 113.5, 72.3, 70.7, 40.2, 30.5, 30.4, 28.4, 27.5, 23.2, 19.3, 17.9, 14.3, 7.0, 5.9. HRMS-ESI (m/z): $[M+H]^+$ calcd for $C_{23}H_{43}O_2Si$ 397.3138; found 397.3128. Optical Rotation: $[\alpha]_D^{25.0}$ = -32.6° (c = 3.56, $CHCl_3$).

Ee was determined by the corresponding ketone product by silyl enol ether deprotection method III. Yield: >95%.

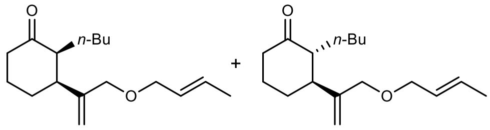

^{1}H NMR (500 MHz, $CDCl_3$) δ 5.77 – 5.53 (m, 2H), 5.21 (s, 0.37H), 5.17 (s, 0.63H), 4.98 (s, 0.63H), 4.88 (s, 0.37H), 3.98 – 3.80 (m, 4H), 2.72 (m, 0.37 H), 2.52 – 1.06 (m, 16.63H), 0.86 (m, 3H). ^{13}C NMR (125 MHz, $CDCl_3$) δ 214.7, 213.0, 147.6, 145.4, 129.8, 127.5, 113.8, 113.0, 72.5, 71.9, 71.1, 54.1, 53.1, 48.6, 43.8, 42.1, 39.0, 31.7, 30.3, 29.7, 27.2, 26.1, 25.4, 24.7, 23.1, 22.8, 18.0, 17.9, 14.2, 14.1. HRMS-ESI (m/z): $[M+H]^+$ calcd for $C_{17}H_{29}O_2$ 265.2168; found 265.2163.

HPLC condition: OD-H, hexane/iPrOH = 99.8/0.2, 0.3 ml/min, 20 °C, 210 nm.

Ee = 96%. (the right two peaks were used to calculate ee due to more effective separation)

Supplementary Figure 40 HPLC spectra for racemic and chiral 3jk's deprotection product

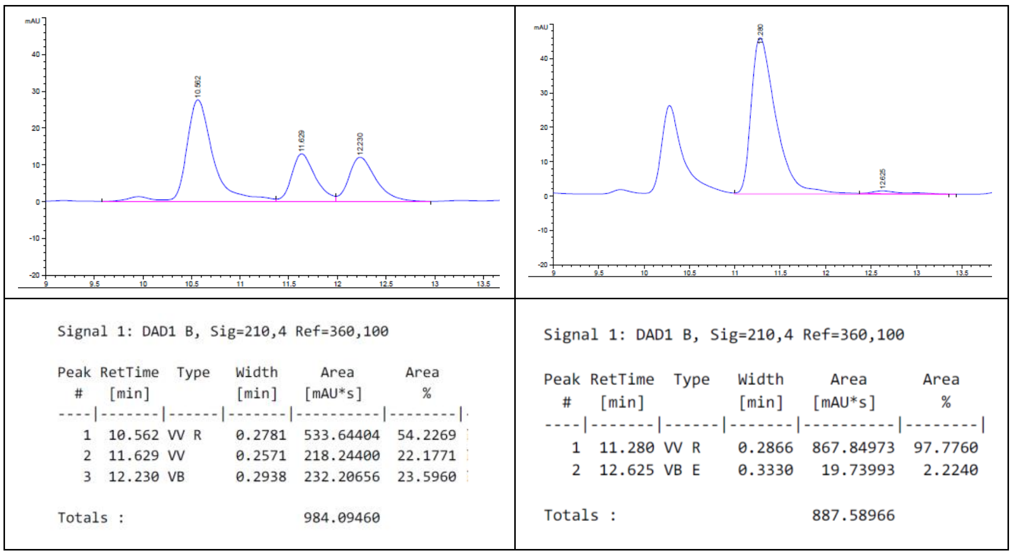

Signal 1: DAD1 B, Sig=210,4 Ref=360,100

Peak #	RetTime [min]	Type	Width [min]	Area [mAU*s]	Area %
1	10.562	VV R	0.2781	533.64404	54.2269
2	11.629	VV	0.2571	218.24400	22.1771
3	12.230	VB	0.2938	232.20656	23.5960
Totals :				984.09460	

Signal 1: DAD1 B, Sig=210,4 Ref=360,100

Peak #	RetTime [min]	Type	Width [min]	Area [mAU*s]	Area %
1	11.280	VV R	0.2866	867.84973	97.7760
2	12.625	VB E	0.3330	19.73993	2.2240
Totals :				887.58966	

Set 6 Heteroatom effect on regioselectivity

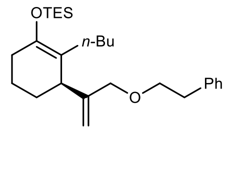

(3jl): ^{1}H NMR (400 MHz, $CDCl_3$) δ 7.30-7.18 (m, 5H), 5.12 (s, 1H), 4.85 (s, 1H), 4.01 (d, *J* = 12.9 Hz, 1H), 3.84 (d, *J* = 12.9 Hz, 1H), 3.67 (m, 1H), 3.57 (m, 1H), 2.97 – 2.84 (m, 3H), 2.42-2.31 (m, 1H), 2.15-1.94 (m, 2H), 1.68-1.19 (m, 9H), 1.00 (t, *J* = 7.9 Hz, 9H), 0.87 (t, *J* = 6.7 Hz, 3H), 0.67 (q, *J* = 7.9 Hz, 6H). ^{13}C NMR (100 MHz, $CDCl_3$) δ 148.0, 145.7, 139.2, 129.1, 128.4, 126.3, 116.7, 113.5, 73.4, 71.3, 40.3, 36.4, 30.5, 30.4, 28.4, 27.5, 23.2, 19.3, 14.3, 7.0, 5.9. HRMS-ESI (m/z): $[M+H]^+$ calcd for $C_{27}H_{45}O_2Si$ 429.3189; found 429.3176. Optical Rotation: $[\alpha]_D^{22.4}$ = -51.67° (*c* = 1.59, $CHCl_3$).

Ee was determined by the corresponding ketone product by silyl enol ether deprotection method III. Yield: >95%.

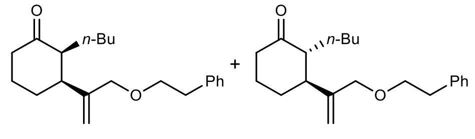

^{1}H NMR (500 MHz, $CDCl_3$) δ 7.31 – 7.19 (m, 5H), 5.17 (s, 0.56H), 5.12 (s. 0.44H), 4.94 (s, 0.44H), 4.85 (s, 0.56H), 4.00 – 3.82 (m, 2H), 3.69 – 3.56 (m, 2H), 2.89 (q, *J* = 7.3 Hz, 2H), 2.63 (m, 0.56H), 2.48 – 1.05 (m, 13.44H), 0.85 (m, 3H). ^{13}C NMR (125 MHz, $CDCl_3$) δ 214.5, 213.0, 147.5, 145.3, 139.1, 129.1, 128.5, 126.4, 126.3, 113.8, 113.2, 73.6, 73.2, 71.5, 71.4, 54.0, 53.0, 48.5, 43.6, 42.0, 39.0, 36.5, 31.6, 30.4, 30.2, 29.7, 27.2, 26.1, 25.4, 24.6, 23.1, 22.8, 14.2, 14.1. HRMS-ESI (m/z): $[M+H]^+$ calcd for $C_{21}H_{31}O_2$ 315.2324; found 315.2318.

See next page for HPLC result.

HPLC condition: OD-H, hexane/iPrOH = 99.5/0.5, 0.5 ml/min, 20 °C, 210 nm.

Ee = 93%. (the right two peaks were used to calculate ee due to more effective separation)

Supplementary Figure 41 HPLC spectra for racemic and chiral 3jl's deprotection product

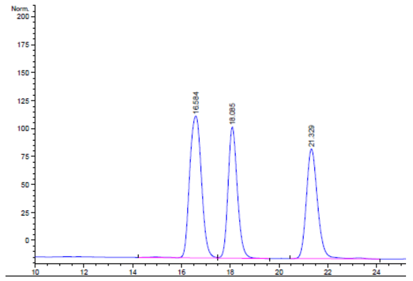

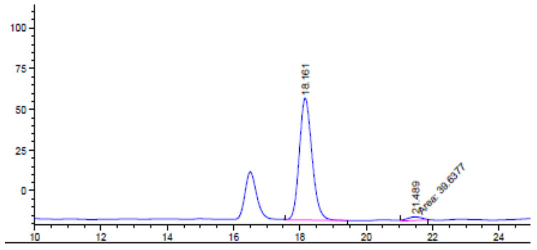

Signal 1: DAD1 B, Sig=210,4 Ref=360,100

Peak #	RetTime [min]	Type	Width [min]	Area [mAU*s]	Area %
1	16.584	VV R	0.5386	3303.00562	40.6206
2	18.085	VB	0.4085	2394.98389	29.4537
3	21.329	BV R	0.4914	2433.35693	29.9256

Totals : 8131.34644

Signal 1: DAD1 B, Sig=210,4 Ref=360,100

Peak #	RetTime [min]	Type	Width [min]	Area [mAU*s]	Area %
1	18.161	VB	0.4110	1080.55945	96.4615
2	21.489	MM	0.4055	39.63771	3.5385

Totals : 1120.19716

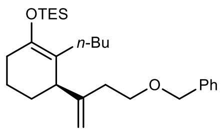

(3jm): ^{1}H NMR (500 MHz, $CDCl_3$) δ 7.34 – 7.25 (m, 5H), 4.89 (s, 1H), 4.77 (s, 1H), 4.51 (m, 2H), 3.65 – 3.55 (m, 2H), 2.82 (m, 1H), 2.40 – 1.20 (m, 14H), 1.00 (t, J = 8.0 Hz, 9H), 0.88 (m, 3H), 0.66 (q, J = 8.0 Hz, 6H). ^{13}C NMR (100 MHz, $CDCl_3$) δ 148.5, 145.6, 138.6, 128.4, 127.8, 127.6, 116.9, 112.4, 73.1, 69.9, 44.1, 34.8, 30.5, 28.4, 27.5, 23.2, 19.5, 14.3, 7.0, 5.9. HRMS-ESI (m/z): $[M+H]^+$ calcd for $C_{27}H_{45}O_2Si$ 429.3189; found 429.3182. Optical Rotation: $[\alpha]_D^{25.0}$ = -18.8° (c = 1.23, $CHCl_3$).

HPLC: OD-H, hexane/iPrOH = 100/0, 0.4 ml/min, 20 °C, 210 nm.

Ee = 89%.

Supplementary Figure 42 HPLC spectra for racemic and chiral 3jm

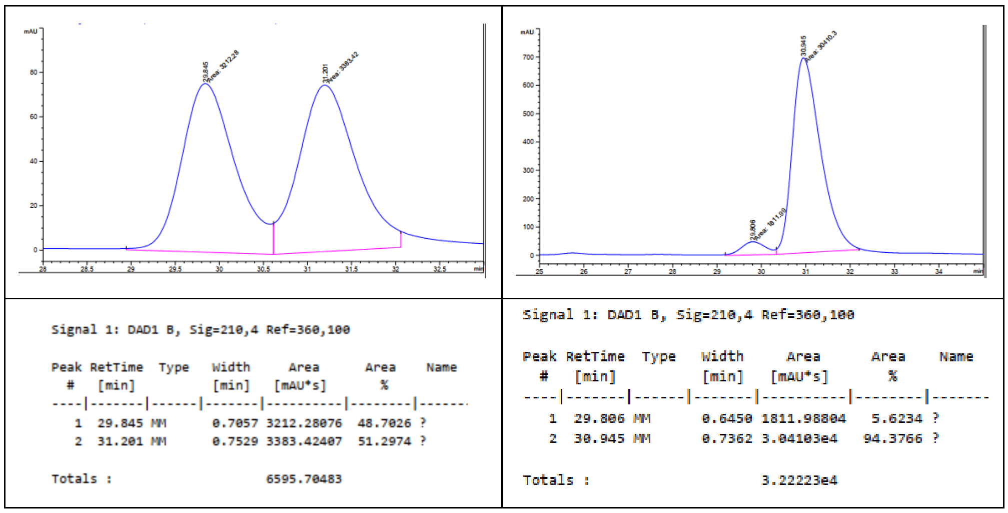

Signal 1: DAD1 B, Sig=210,4 Ref=360,100

Peak #	RetTime [min]	Type	Width [min]	Area [mAU*s]	Area %	Name
1	29.845	MM	0.7057	3212.28076	48.7026	?
2	31.201	MM	0.7529	3383.42407	51.2974	?
Totals :				6595.70483		

Signal 1: DAD1 B, Sig=210,4 Ref=360,100

Peak #	RetTime [min]	Type	Width [min]	Area [mAU*s]	Area %	Name
1	29.806	MM	0.6450	1811.98804	5.6234	?
2	30.945	MM	0.7362	3.04103e4	94.3766	?
Totals :				3.22223e4		

Set 7 Gem-Dialkyl substitutions

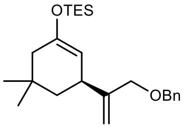

(3qa): ^{1}H NMR (400 MHz, $CDCl_3$) δ 7.42 – 7.29 (m, 5H), 5.13 (s, *J* = 1.6 Hz, 1H), 5.05 (s, 1H), 4.80 (s, 1H), 4.55 (dd, *J* = 22.4, 12.0 Hz, 2H), 4.11 – 3.96 (dd, *J* = 28.8, 12.8 Hz, 2H), 3.08 – 2.96 (m, 1H), 2.01 (m, 1H), 1.72 (d, *J* = 16.9 Hz, 1H), 1.54 (m, 1H), 1.07 (m, 1H), 1.05 – 0.93 (m, 15H), 0.76 – 0.64 (m, 6H). ^{13}C NMR (100 MHz, $CDCl_3$) δ 150.6, 150.4, 138.5, 128.4, 127.7, 127.5, 111.1, 105.3, 72.1, 71.9, 43.5, 42.6, 37.2, 31.6, 31.1, 25.2, 6.7, 5.1. HRMS-ESI (m/z): $[M+H]^+$ calcd for $C_{24}H_{39}O_2Si$: 387.2719; found 387.2709. Optical Rotation: $[\alpha]_D^{26.9}$ = +3.6º for L1 (*c* = 1.11, $CHCl_3$).

Ee was determined by the corresponding ketone product by silyl enol ether deprotection method III. Yield: >95%.

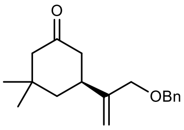

^{1}H NMR (400 MHz, $CDCl_3$) δ 7.42 – 7.29 (m, 5H), 5.17 (s, 1H), 5.03 (s, 1H), 4.56 – 4.46 (m, 2H), 4.08 – 3.98 (m, 2H), 2.69 (m, 1H), 2.45 (m, 1H), 2.31 – 2.20 (m, 2H), 2.13 (m, 1H), 1.73 (m, 1H), 1.60 (d, *J* = 12.9 Hz, 1H), 1.10 (s, 3H), 0.94 (s, 3H). ^{13}C NMR (100 MHz, $CDCl_3$) δ 211.4, 148.4, 138.2, 128.5, 127.8, 112.0, 72.3, 72.2, 54.5, 46.2, 44.0, 37.3, 35.3, 32.3, 25.7. HRMS-ESI (m/z): $[M+H]^+$ calcd for $C_{18}H_{25}O_2$ 273.1855; found 273.1845. Optical Rotation: $[\alpha]_D^{25.5}$ = +12.7º for L1 (*c* = 0.32, $CHCl_3$).

HPLC condition: OD-H, hexane/iPrOH = 99.0/1.0, 1.0 ml/min, 20 ˚C, 210 nm.

From L1, ee = 67%; From L3, ee = 59%.

Supplementary Figure 43 HPLC spectra for racemic and chiral 3qa's deprotection product

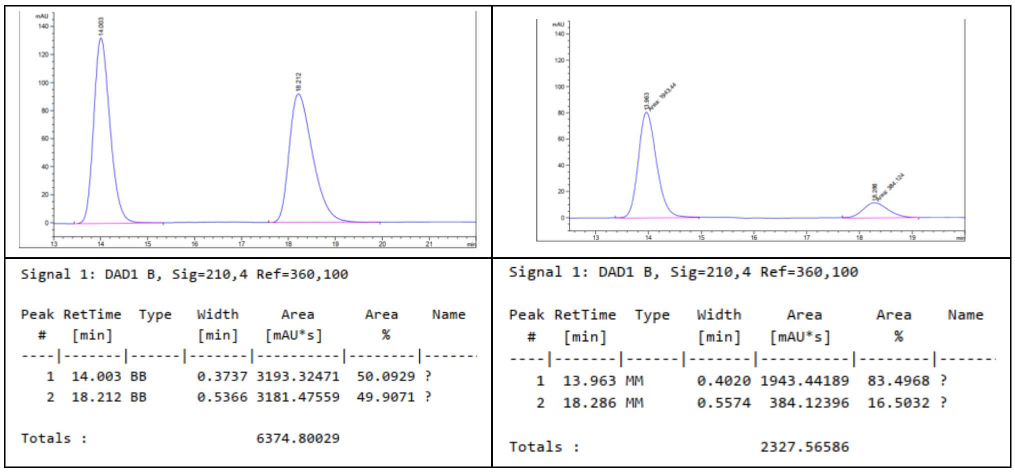

Signal 1: DAD1 B, Sig=210,4 Ref=360,100

Peak #	RetTime [min]	Type	Width [min]	Area [mAU*s]	Area %	Name
1	14.003	BB	0.3737	3193.32471	50.0929	?
2	18.212	BB	0.5366	3181.47559	49.9071	?
Totals :				6374.80029		

Signal 1: DAD1 B, Sig=210,4 Ref=360,100

Peak #	RetTime [min]	Type	Width [min]	Area [mAU*s]	Area %	Name
1	13.963	MM	0.4020	1943.44189	83.4968	?
2	18.286	MM	0.5574	384.12396	16.5032	?
Totals :				2327.56586		

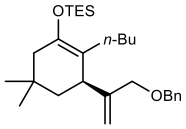

(3ra): ^{1}H NMR (400 MHz, $CDCl_3$) δ 7.37 – 7.26 (m, 5H), 5.17 (s, 1H), 5.04 – 4.96 (s, 1H), 4.58 – 4.47 (m, 2H), 3.94 (d, J = 1.3 Hz, 1H), 3.83 (d, J = 1.3 Hz, 1H), 2.98 (m, 1H), 2.40 (m, 1H), 1.99 (m, 1H), 1.68 (m, 1H), 1.61 – 1.51 (m, 1H), 1.48 – 1.41 (m, 1H), 1.37 – 1.29 (m, 1H), 1.27 – 1.17 (m, 4H), 0.96 (t, J = 8.0 Hz, 9H), 0.91 (d, J = 13.3 Hz, 6H), 0.90 (s, 3H), 0.87 (s, 3H), 0.63 (q, J = 8.0 Hz, 6H). ^{13}C NMR (100 MHz, $CDCl_3$) δ 149.1, 144.5, 138.7, 128.5, 127.7, 127.6, 115.3, 112.4, 72.4, 71.1, 44.5, 43.4, 41.7, 31.7, 30.8, 30.2, 26.9, 24.8, 23.1, 14.3, 7.1, 5.8. HRMS-ESI (m/z): $[M+H]^+$ calcd for $C_{28}H_{47}O_2Si$: 443.3345; found 443.3331. Optical Rotation: $[\alpha]_D^{26.0}$ = -24.49° (c = 0.49, $CHCl_3$).

HPLC condition: OD-H, hexane/iPrOH = 100/0, 1.0 ml/min, 20 °C, 210 nm.

Ee = 96%.

Supplementary Figure 44 HPLC spectra for racemic and chiral 3ra

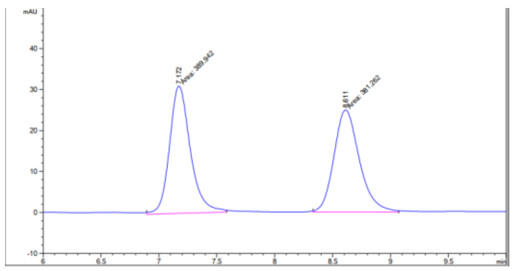

Signal 1: DAD1 B, Sig=210,4 Ref=360,100

Peak #	RetTime [min]	Type	Width [min]	Area [mAU*s]	Area %	Name
1	7.172	MM	0.2084	389.94156	50.5627	?
2	8.611	MM	0.2550	381.26230	49.4373	?
Totals :				771.20386		

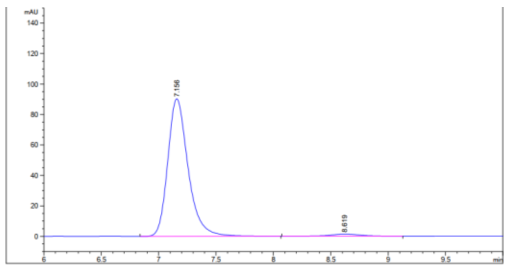

Signal 1: DAD1 B, Sig=210,4 Ref=360,100

Peak #	RetTime [min]	Type	Width [min]	Area [mAU*s]	Area %	Name
1	7.156	BB	0.1878	1112.38635	97.7822	?
2	8.619	BB	0.2625	25.22964	2.2178	?
Totals :				1137.61600		

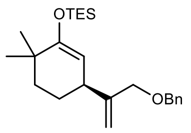

(3sa): ^{1}H NMR (400 MHz, $CDCl_3$) δ 7.39 – 7.26 (m, 5H), 5.12 (s, 1H), 4.99 (s, 1H), 4.56 – 4.46 (m, 3H), 4.08 – 3.93 (m, 2H), 2.94 (m, 1H), 1.79 – 1.69 (m, 1H), 1.54 (m, 1H), 1.46 – 1.36 (m, 2H), 1.04 (s, 6H), 0.98 (t, J = 7.9 Hz, 9H), 0.71 – 0.64 (q, J = 7.9 Hz, 6H). ^{13}C NMR (100 MHz, $CDCl_3$) δ 158.1, 150.2, 138.6, 128.5, 127.8, 127.7, 112.1, 102.7, 72.3, 72.3, 39.3, 36.9, 35.3, 27.3, 27.2, 25.4, 7.0, 5.4. HRMS-ESI (m/z): $[M+H]^+$ calcd for $C_{24}H_{39}O_2Si$: 387.2719; found 387.2708. Optical rotation was not determined due to its low ee.

HPLC condition: OD-H, hexane/iPrOH = 100/0, 0.4 ml/min, 20 °C, 210 nm.

Ee = 4%.

Supplementary Figure 45 HPLC spectra for racemic and chiral 3sa

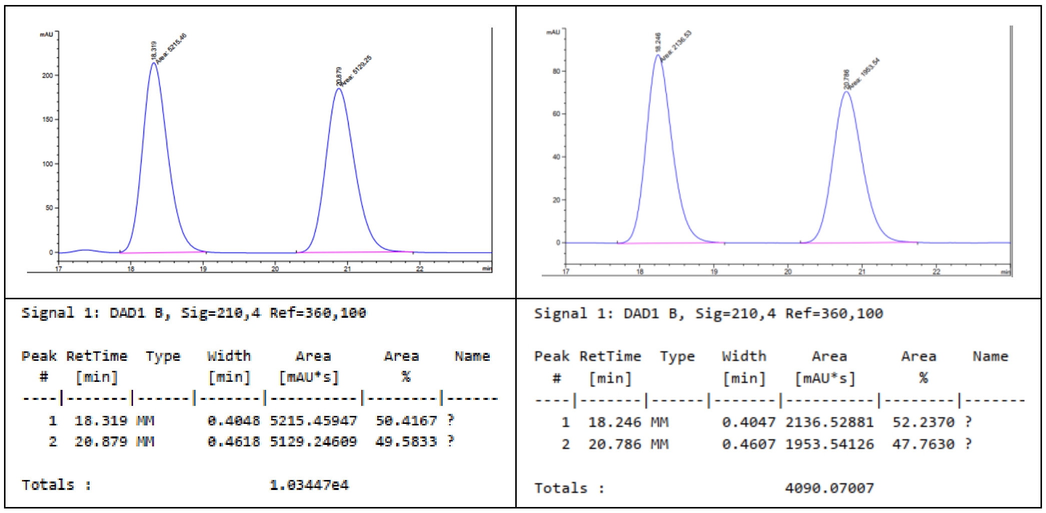

Signal 1: DAD1 B, Sig=210,4 Ref=360,100

Peak #	RetTime [min]	Type	Width [min]	Area [mAU*s]	Area %	Name
1	18.319	MM	0.4048	5215.45947	50.4167	?
2	20.879	MM	0.4618	5129.24609	49.5833	?
Totals :				1.03447e4		

Signal 1: DAD1 B, Sig=210,4 Ref=360,100

Peak #	RetTime [min]	Type	Width [min]	Area [mAU*s]	Area %	Name
1	18.246	MM	0.4047	2136.52881	52.2370	?
2	20.786	MM	0.4607	1953.54126	47.7630	?
Totals :				4090.07007		

Set 8 Control of 1,3-allylic shift

a) Cyclo-Heptadiene & -hexadiene

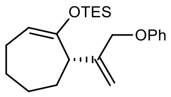

(3tb): ^{1}H NMR (400 MHz, $CDCl_3$) δ 7.30 – 7.24 (m, 2H), 6.96 – 6.90 (m, 3H), 5.29 (s, 1H), 5.16 (s, 1H), 5.09 (dd, J = 7.5, 5.7 Hz, 1H), 4.54 – 4.50 (s, 2H), 3.14 (m, 1H), 2.12 – 2.01 (m, 1H), 1.97 (m, 1H), 1.90 – 1.73 (m, 2H), 1.71 – 1.59 (m, 3H), 1.49 (m, 1H), 0.95 (t, J = 7.9 Hz, 9H), 0.69 – 0.61 (q, J = 7.9 Hz, 6H). ^{13}C NMR (100 MHz, $CDCl_3$) δ 159.0, 155.0, 143.9, 129.5, 120.8, 114.9, 113.3, 108.5, 70.2, 49.4, 28.8, 27.6, 26.4, 24.1, 7.0, 5.2. HRMS-ESI (m/z): $[M+H]^+$ calcd for $C_{22}H_{35}O_2Si$: 359.2406; found 359.2400**.** Optical Rotation: $[\alpha]_D^{21.1}$ = +48.08° (c = 0.52, $CHCl_3$).

HPLC condition: OD-H, hexane/iPrOH = 100/0, 1.0 ml/min, 20 °C, 210 nm.

Ee = 74%.

Supplementary Figure 46 HPLC spectra for racemic and chiral 3tb

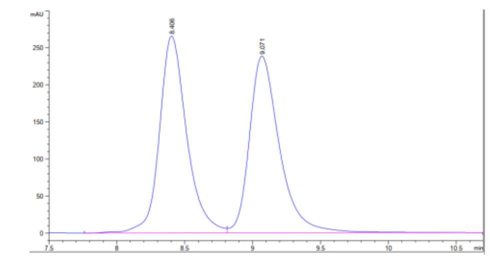

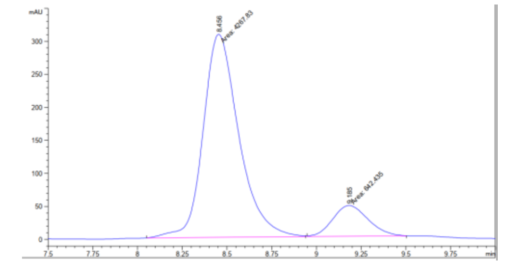

Signal 1: DAD1 B, Sig=210,4 Ref=360,100

Peak #	RetTime [min]	Type	Width [min]	Area [mAU*s]	Area %	Name
1	8.406	BV	0.2112	3718.15918	50.2768	?
2	9.071	VB	0.2319	3677.21753	49.7232	?

Totals : 7395.37671

Signal 1: DAD1 B, Sig=210,4 Ref=360,100

Peak #	RetTime [min]	Type	Width [min]	Area [mAU*s]	Area %	Name
1	8.456	MM	0.2312	4267.82764	86.9165	?
2	9.185	MM	0.2296	642.43524	13.0835	?

Totals : 4910.26288

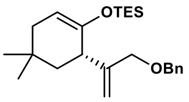

(3ua): ^{1}H NMR (400 MHz, $CDCl_3$) δ 7.38 – 7.26 (m, 5H), 5.18 (m, 1H), 5.01 (m, 1H), 4.77 (m, 1H), 4.56 – 4.46 (m, 2H), 4.02 (m, 2H), 2.95 (m, 1H) 1.97 (m, 1H), 1.68 (m, 1H), 1.51 – 1.43 (m, 2H), 0.98 – 0.89 (m, 15H), 0.62 (q, J = 7.6 Hz, 6H). ^{13}C NMR (125 MHz, $CDCl_3$) δ 149.7, 147.5, 138.9, 128.4, 127.8, 127.5, 112.6, 103.3, 72.0, 71.4, 43.9, 43.4, 38.2, 31.6, 29.8, 24.9, 6.9, 5.2. HRMS-ESI (m/z): $[M+H]^+$ calcd for $C_{24}H_{39}O_2Si$: 387.2719; found 387.2706. Optical Rotation: $[\alpha]_D^{26.1}$ = +20.92° for L1 at 0 °C (c = 0.72, $CHCl_3$).

HPLC condition: OD-H, hexane/iPrOH = 100/0, 1.0 ml/min, 20 °C, 210 nm.

From L3, ee = 39%; from L1 at 0 °C, ee = 85%.

Supplementary Figure 47 HPLC spectra for racemic and chiral 3ua

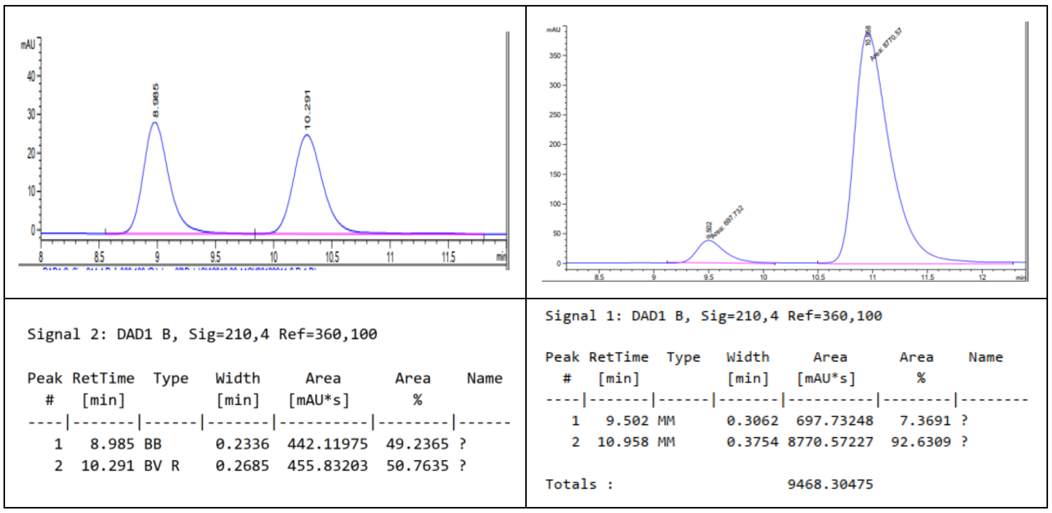

Signal 2: DAD1 B, Sig=210,4 Ref=360,100

Peak #	RetTime [min]	Type	Width [min]	Area [mAU*s]	Area %	Name
1	8.985	BB	0.2336	442.11975	49.2365	?
2	10.291	BV R	0.2685	455.83203	50.7635	?

Signal 1: DAD1 B, Sig=210,4 Ref=360,100

Peak #	RetTime [min]	Type	Width [min]	Area [mAU*s]	Area %	Name
1	9.502	MM	0.3062	697.73248	7.3691	?
2	10.958	MM	0.3754	8770.57227	92.6309	?
Totals :				9468.30475		

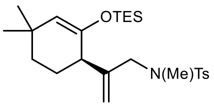

(3vc): ^{1}H NMR (400 MHz, $CDCl_3$) δ 7.70 – 7.65 (m, 2H), 7.32 (d, J = 8.0 Hz, 2H), 5.03 (s, 1H), 5.00 (s, 1H), 4.71 (s, 1H), 3.97 (d, J = 13.8 Hz, 1H), 3.13 (d, J = 13.8 Hz, 1H), 2.83 (m, 1H), 2.62 (s, 3H), 2.43 (s, 3H), 1.82 (m, 1H), 1.62 – 1.53 (m, 1H), 1.37 (m, 1H), 1.25 (m, 1H), 0.98 (d, J = 6.1 Hz, 6H), 0.94 (t, J = 8.0 Hz, 9H), 0.67 – 0.60 (q, J = 8.0 Hz, 6H). ^{13}C NMR (100 MHz, $CDCl_3$) δ 148.7, 144.0, 143.4, 134.3, 129.7, 127.6, 116.4, 115.4, 54.4, 42.5, 34.0, 33.3, 32.0, 31.1, 30.1, 25.0, 21.6, 6.9, 5.2. HRMS-ESI (m/z): $[M+H]^+$ calcd for $C_{25}H_{42}NO_3SSi$: 464.2655; found 464.2641. Optical Rotation: $[\alpha]_D^{25.0}$ = -55.8° (c = 0.42, $CHCl_3$).

HPLC condition: OD-H+OD-H, hexane/iPrOH = 99.3/0.7, 0.6 ml/min, 20 °C, 210 nm.

Ee > 99%.

Supplementary Figure 48 HPLC spectra for racemic and chiral 3vc

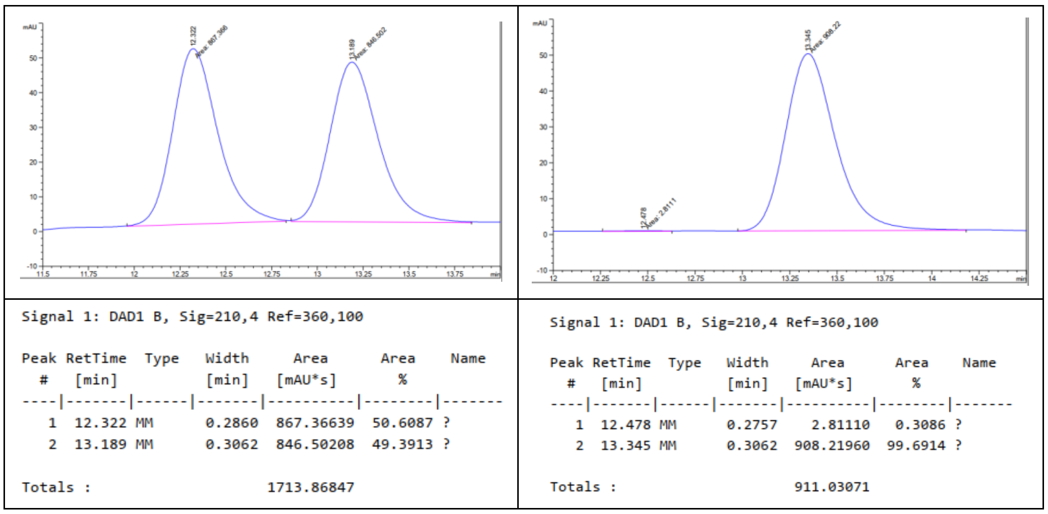

Signal 1: DAD1 B, Sig=210,4 Ref=360,100

Peak #	RetTime [min]	Type	Width [min]	Area [mAU*s]	Area %	Name
1	12.322	MM	0.2860	867.36639	50.6087	?
2	13.189	MM	0.3062	846.50208	49.3913	?
Totals :				1713.86847		

Signal 1: DAD1 B, Sig=210,4 Ref=360,100

Peak #	RetTime [min]	Type	Width [min]	Area [mAU*s]	Area %	Name
1	12.478	MM	0.2757	2.81110	0.3086	?
2	13.345	MM	0.3062	908.21960	99.6914	?
Totals :				911.03071		

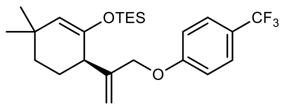

(3vn): ^{1}H NMR (400 MHz, $CDCl_3$) δ 7.51 (d, *J* = 8.5 Hz, 2H), 6.99 (d, *J* = 8.5 Hz, 2H), 5.20 (s, 1H), 5.08 (s, 1H), 4.75 (s, 1H), 4.58 (s, 2H), 2.86 (t, *J* = 6.1 Hz, 1H), 1.86 (m, 1H), 1.75 – 1.65 (m, 1H), 1.46 (m, 1H), 1.35 – 1.27 (m, 1H), 1.04 – 0.92 (m, 15H), 0.64 (q, *J* = 7.7 Hz, 6H). ^{13}C NMR (100 MHz, $CDCl_3$) δ 161.5, 148.4, 144.7, 126.9(q), 116.9, 115.0, 113.6, 70.0, 43.9, 34.3, 32.1, 30.7, 30.6, 26.1, 6.9, 5.2. HRMS-ESI (m/z): $[M+H]^+$ calcd for $C_{24}H_{36}F_3O_2Si$: 441.2437; found 441.2422.

Optical Rotation: $[\alpha]_D^{23.4}$ = -45.95° (*c* = 0.37, $CHCl_3$).

HPLC condition: OD-H+OD-H, hexane/iPrOH = 100/0, 1.0 ml/min, 20 °C, 210 nm.

Ee = 99%.

Supplementary Figure 49 HPLC spectra for racemic and chiral 3vn

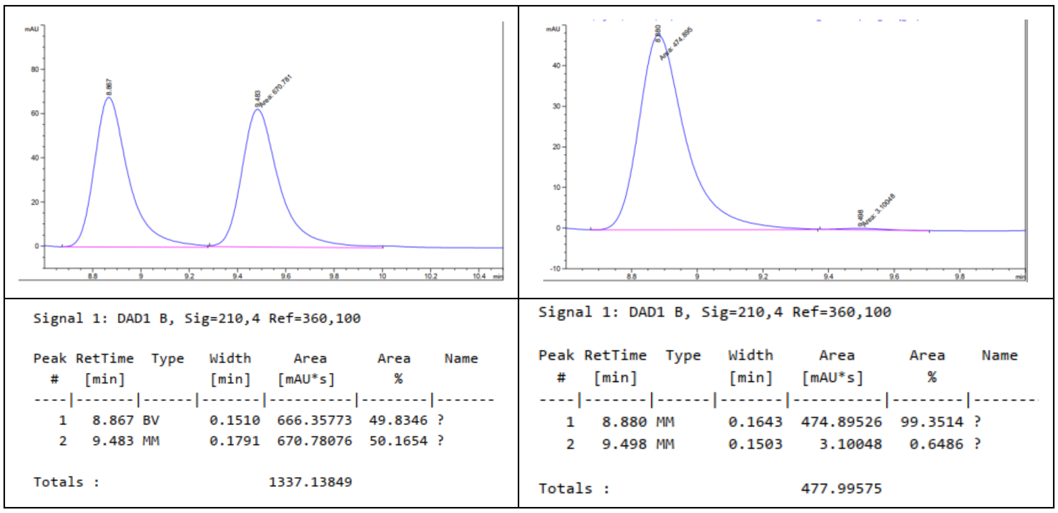

Signal 1: DAD1 B, Sig=210,4 Ref=360,100

Peak #	RetTime [min]	Type	Width [min]	Area [mAU*s]	Area %	Name
1	8.867	BV	0.1510	666.35773	49.8346	?
2	9.483	MM	0.1791	670.78076	50.1654	?

Totals : 1337.13849

Signal 1: DAD1 B, Sig=210,4 Ref=360,100

Peak #	RetTime [min]	Type	Width [min]	Area [mAU*s]	Area %	Name
1	8.880	MM	0.1643	474.89526	99.3514	?
2	9.498	MM	0.1503	3.10048	0.6486	?

Totals : 477.99575

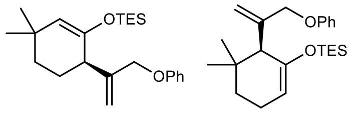

(3vb): Inseparable mixture, 58: 42.

^{1}H NMR (500 MHz, $CDCl_3$) δ 7.26 (m, 2H), 6.97 – 6.89 (m, 3H), 5.32 (s, 0.42H), 5.23 (s, 0.58H), 5.09 (s, 0.42H), 5.05 (s, 0.58H), 4.89 (s, 0.42H), 4.75 (s, 0.58H), 4.55 – 4.49 (m, 1.61H), 4.45 (d, J = 14.2 Hz, 0.44H), 2.86 (t, J = 6.0 Hz, 0.61H), 2.35 (s, 0.43H), 2.08 (m, 0.84H), 1.85 (m, 0.66H), 1.71 (m, 0.70H), 1.54 – 1.42 (m, 1.20H), 1.29 (m, 1.23H), 1.17 – 1.11 (m, 0.54H), 1.01 (d, J = 3.3 Hz, 3.23H), 0.98 (s, 2.03H), 0.96 (t, J = 8.0 Hz, 9H), 0.91 (s, 1.22H), 0.70 – 0.60 (m, 6H). ^{13}C NMR (125 MHz, $CDCl_3$) δ 159.1, 150.5, 148.6, 145.3, 144.5, 129.4, 120.7, 120.7, 116.8, 115.0, 114.9, 113.8, 113.1, 102.6, 71.4, 69.8, 54.5, 43.8, 34.2, 33.8, 32.0, 31.2, 30.8, 30.6, 28.1, 28.0, 26.0, 21.2, 7.0, 7.0, 5.2, 5.2. HRMS-ESI (m/z): $[M+H]^+$ calcd for $C_{23}H_{37}O_2Si$ 373.2563; found: 373.2548.

Optical Rotation: $[\alpha]_D^{23.4}$ = +18.32° (c = 1.22, $CHCl_3$).

HPLC condition: OD-H, hexane/iPrOH = 100/0, 1.0 ml/min, 20 °C, 210 nm.

Ee of the major product (before allylic shift) was not determined due to ineffective separation.

Ee = 96%. [minor product (after allylic shift)]

Supplementary Figure 50 HPLC spectra for racemic and chiral 3vb

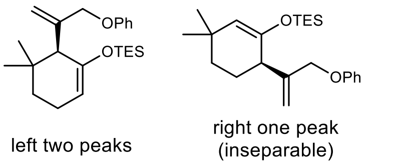

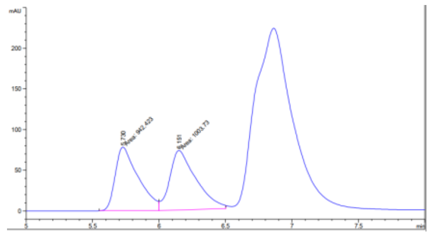

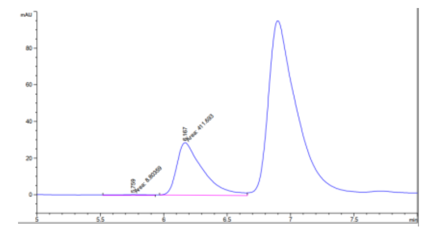

Signal 1: DAD1 B, Sig=210,4 Ref=360,100

Peak #	RetTime [min]	Type	Width [min]	Area [mAU*s]	Area %	Name
1	5.730	MM	0.2008	942.42310	48.4249	?
2	6.151	MM	0.2283	1003.72955	51.5751	?

Totals : 1946.15265

Signal 1: DAD1 B, Sig=210,4 Ref=360,100

Peak #	RetTime [min]	Type	Width [min]	Area [mAU*s]	Area %	Name
1	5.759	MM	0.3193	8.80359	2.0936	?
2	6.167	MM	0.2388	411.69263	97.9064	?

Totals : 420.49622

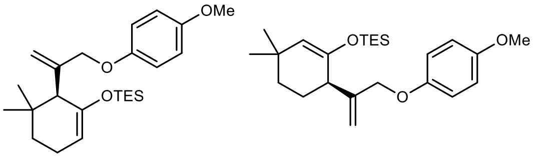

(3vo): Inseparable mixture, 82: 18.

^{1}H NMR (400 MHz, $CDCl_3$) δ 6.91 – 6.78 (m, 4H), 5.31 (s, 0.82H), 5.22 (s, 0.18H), 5.08 (s, 1H), 5.04 (s, 0.18H), 4.88 (t, J = 3.9 Hz, 1H), 4.74 (s, 0.18H), 4.51 – 4.37 (m, 2H), 3.77 (s, 3H), 2.85 (t, J = 12 Hz, 0.18H*),* 2.33 (s, 0.82H), 2.07 (m, 1.62H), 1.88 – 1.81 (m, 0.18H), 1.72 – 1.55 (m, 0.18H), 1.57 – 1.42 (m, 1H), 1.25 – 1.34 (m, 0.36H), 1.13 (m, 1H), 1.02 – 0.88 (m, 15H), 0.69 – 0.61 (m, 6H). ^{13}C NMR (100 MHz, $CDCl_3$) δ 153.8, 153.3, 150.5, 148.7, 145.5, 144.8, 117.8, 116.7, 115.8, 114.6, 113.6, 113.0, 102.6, 72.1, 70.6, 55.8, 54.4, 43.8, 34.1, 33.8, 32.0, 31.1, 30.8, 30.5, 28.1, 28.0, 26.0, 21.2, 7.0, 5.2. HRMS-ESI (m/z): $[M+H]^+$ calcd for $C_{24}H_{39}O_3Si$ 403.2668; found: 403.2660. Optical Rotation: $[\alpha]_D^{24.6}$ = +85.80° (c = 0.54, $CHCl_3$).

HPLC condition: OD-H+OD-H, hexane/iPrOH=100/0, 0.5 ml/min, 20 °C, 210 nm.

At rt, ee > 99%; at 0 °C, ee > 99%. [major product (after allylic shift)]

Supplementary Figure 51 HPLC spectra for racemic and chiral 3vo

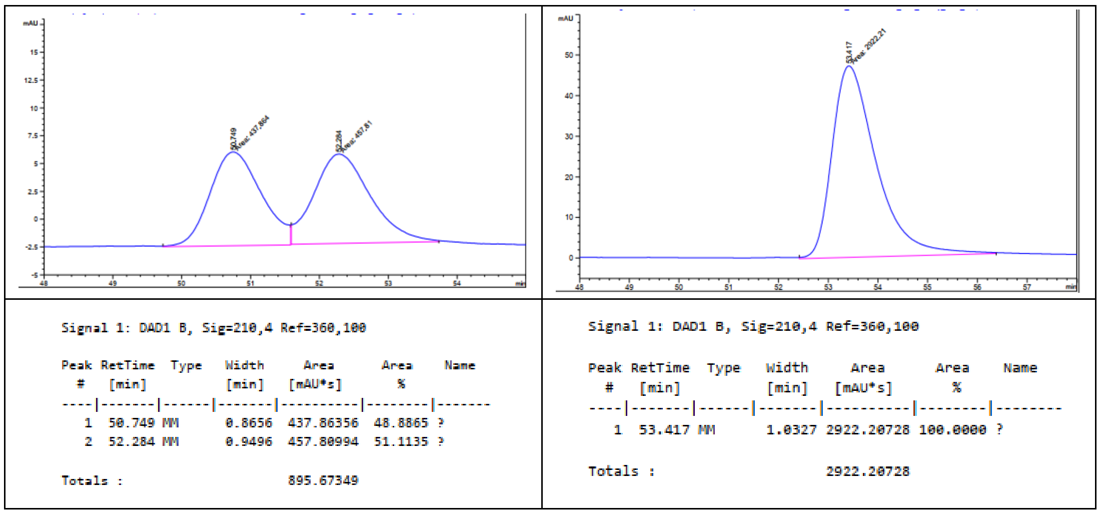

Signal 1: DAD1 B, Sig=210,4 Ref=360,100

Peak #	RetTime [min]	Type	Width [min]	Area [mAU*s]	Area %	Name
1	50.749	MM	0.8656	437.86356	48.8865	?
2	52.284	MM	0.9496	457.80994	51.1135	?

Totals : 895.67349

Signal 1: DAD1 B, Sig=210,4 Ref=360,100

Peak #	RetTime [min]	Type	Width [min]	Area [mAU*s]	Area %	Name
1	53.417	MM	1.0327	2922.20728	100.0000	?

Totals : 2922.20728

See next page for further HPLC analysis about the minor product (**3vo'**).

HPLC condition: OD-H, hexane/iPrOH=100/0, 1.0 ml/min, 20 ºC, 210 nm.

At rt, ee = 97%; at 0 ºC, ee = 97%. [minor product (before allylic shift)]

Supplementary Figure 52 HPLC spectra for racemic and chiral 3vo’

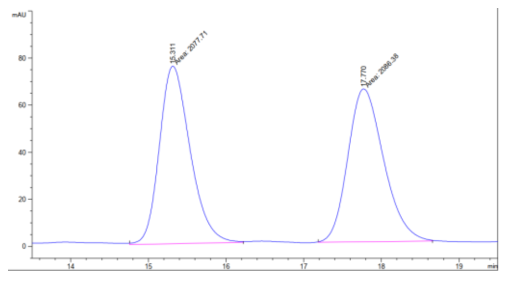

Signal 1: DAD1 B, Sig=210,4 Ref=360,100

Peak #	RetTime [min]	Type	Width [min]	Area [mAU*s]	Area %	Name
1	15.311	MM	0.4587	2077.71436	49.8960	?
2	17.770	MM	0.5353	2086.37817	50.1040	?
Totals :				4164.09253		

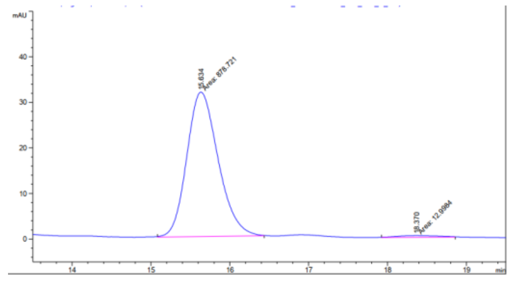

Signal 1: DAD1 B, Sig=210,4 Ref=360,100

Peak #	RetTime [min]	Type	Width [min]	Area [mAU*s]	Area %	Name
1	15.634	MM	0.4613	878.72083	98.5423	?
2	18.370	MM	0.5935	12.99844	1.4577	?
Totals :				891.71927		

b) Cyclo-Pentadiene

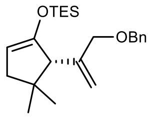

(3wa): ^{1}H NMR (500 MHz, $CDCl_3$) δ 7.37 – 7.32 (m, 4H), 7.30-7.27 (m, 1H), 5.29 (s, 1H), 4.97 (s, 1H), 4.61 (s, 1H), 4.57 (d, J = 11.9 Hz, 1H), 4.46 (d, J = 11.9 Hz, 1H), 3.95 (s, 2H), 2.67 (s, 1H), 2.13 – 2.03 (d, J = 14.8 Hz, 1H), 1.91 (d, J = 14.8 Hz, 1H), 1.14 (s, 3H), 1.00 – 0.85 (m, 12H), 0.66 (m, 6H). ^{13}C NMR (125 MHz, $CDCl_3$) δ 154.7, 144.2, 138.8, 128.4, 127.8, 127.5, 113.0, 101.4, 73.0, 71.9, 59.9, 43.4, 40.2, 32.3, 25.2, 6.8, 4.9. HRMS-ESI (m/z): $[M+H]^+$ calcd for $C_{23}H_{37}O_2Si$ 373.2563; found 373.2556**.** Optical Rotation: $[\alpha]_D^{23.0}$ = +28.76 (c = 1.18, $CHCl_3$).

HPLC condition: OD-H, hexane/iPrOH = 100/0, 0.7 ml/min, 20 °C, 210 nm.

Ee = 94%.

Supplementary Figure 53 HPLC spectra for racemic and chiral 3wa

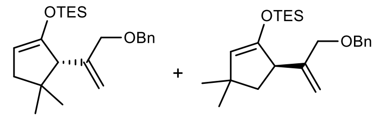

left two peaks   right two peaks

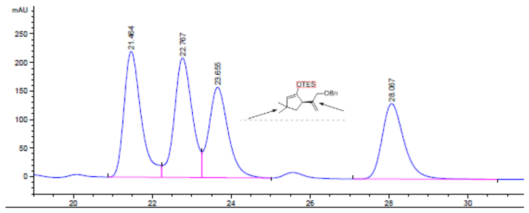

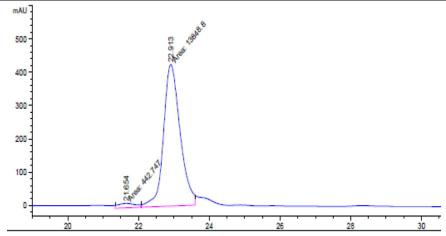

Signal 1: DAD1 B, Sig=210,4 Ref=360,100

Uncalibrated Peaks:

Peak #	RetTime [min]	Type	Width [min]	Area [mAU*s]	Area %
1	21.464	BV	0.4589	6627.94775	27.9613
2	22.767	VV	0.4830	6588.15283	27.7934
3	23.655	VB	0.5115	5372.94043	22.6668
4	28.067	BB	0.5959	5114.95313	21.5784

Uncalib. totals : 2.37040e4 100.0000

Signal 1: DAD1 B, Sig=210,4 Ref=360,100

Uncalibrated Peaks:

Peak #	RetTime [min]	Type	Width [min]	Area [mAU*s]	Area %
1	21.654	MM	0.5645	442.74689	3.0980
2	22.913	MM	0.5439	1.38488e4	96.9020

Uncalib. totals : 1.42915e4 100.0000

[Chiral NHC-Ni(allyl)Cl]

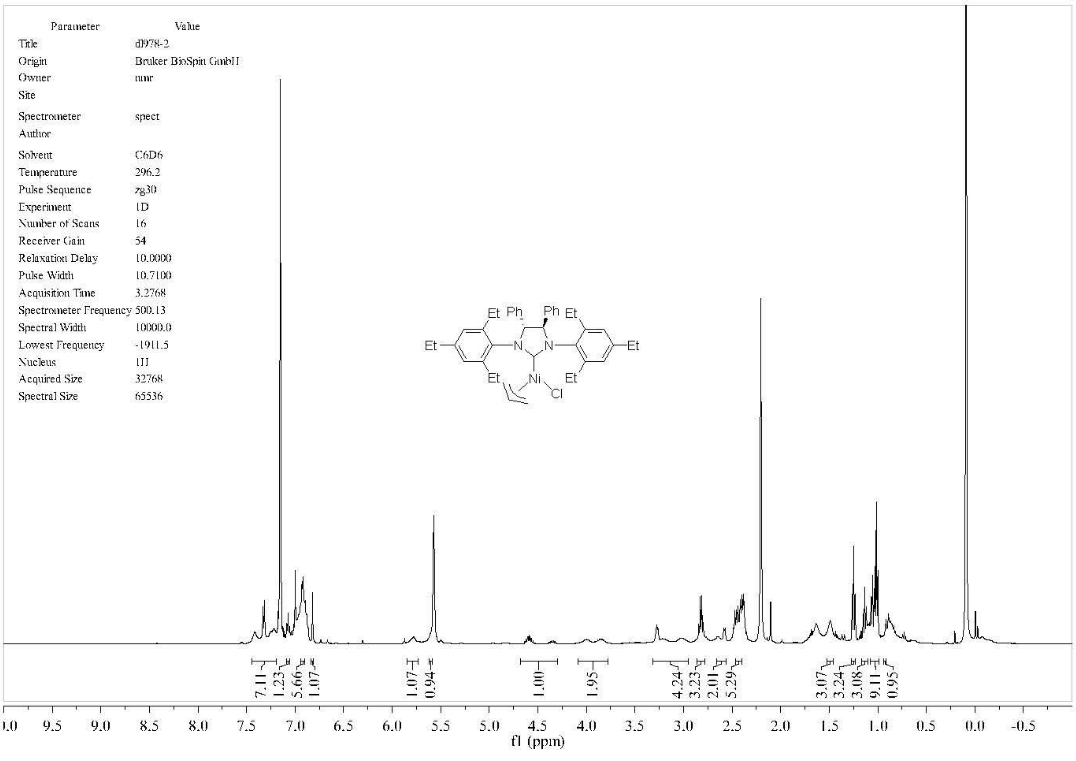

Supplementary Figure 54 ^{1}H NMR of [L2-Ni(allyl)Cl]

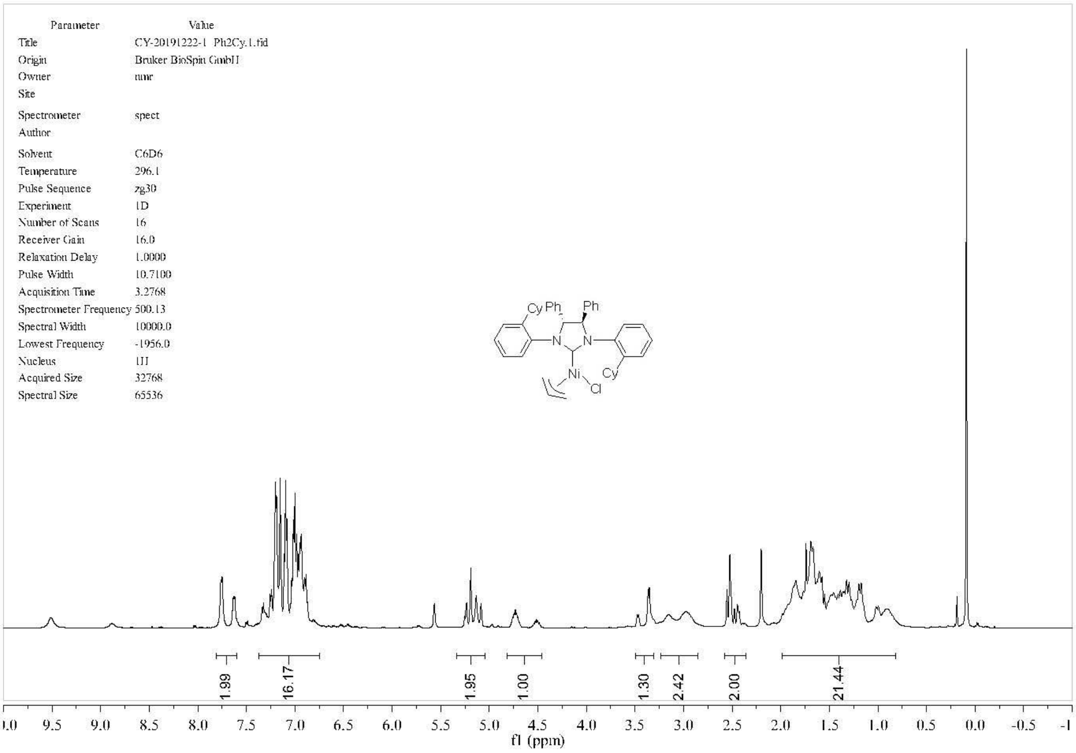

Supplementary Figure 55 ^{1}H NMR of [L3-Ni(allyl)Cl]

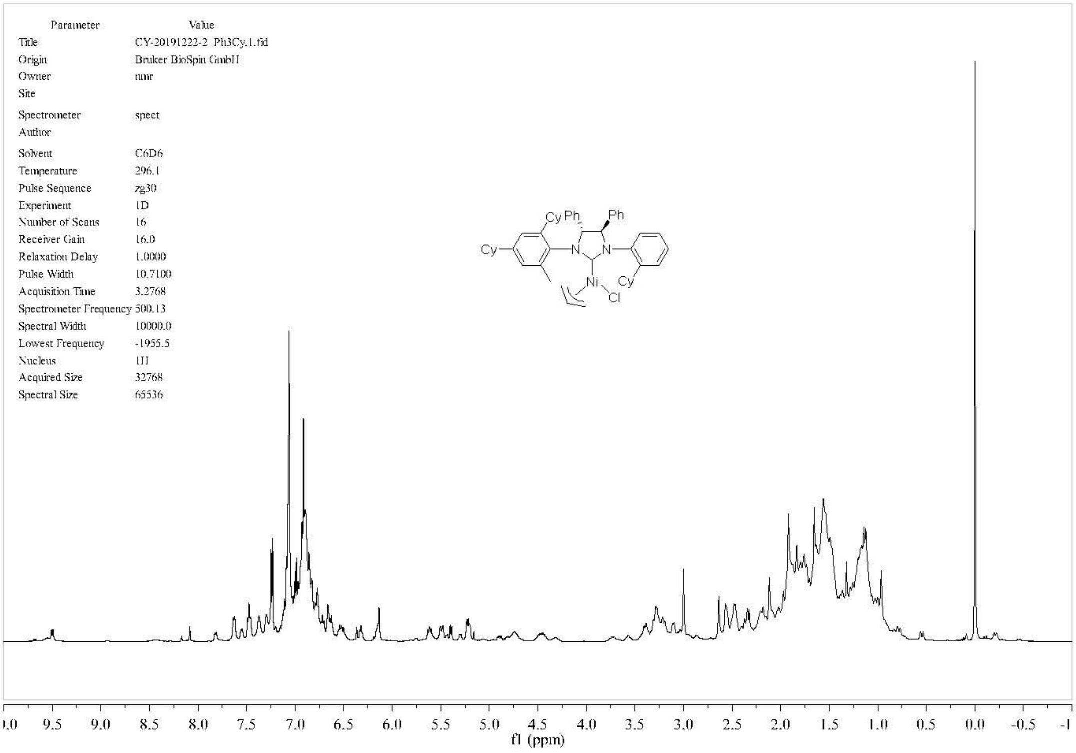

Supplementary Figure 56 ^{1}H NMR of [L1-Ni(allyl)Cl]

Diene

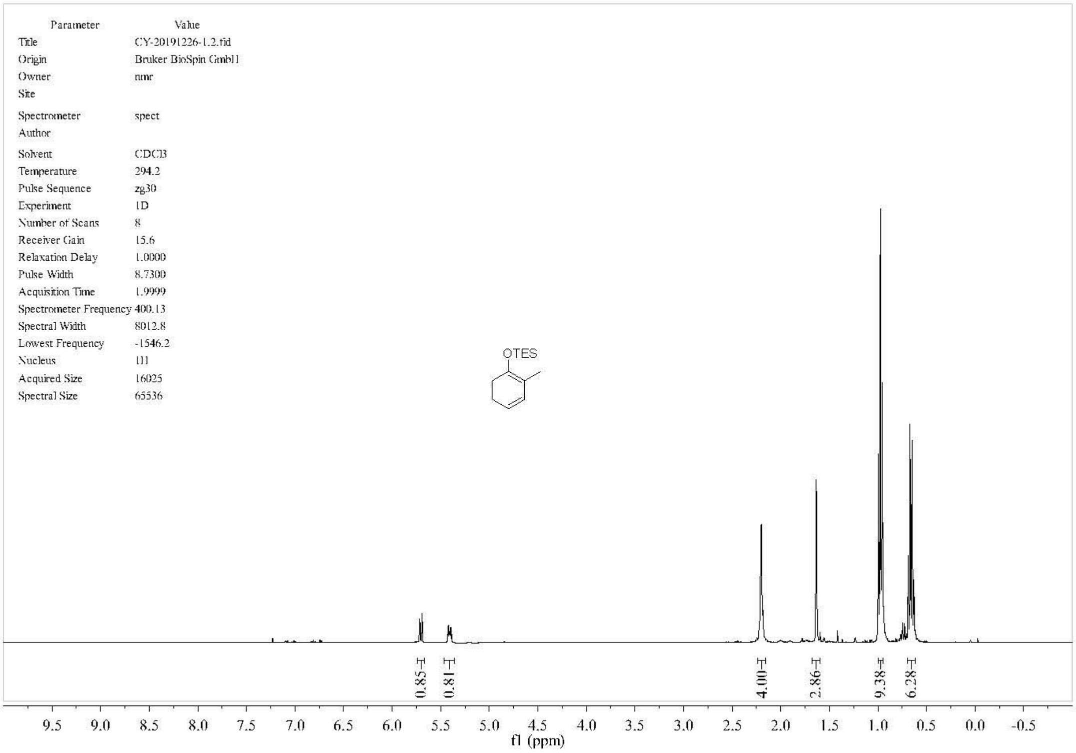

Supplementary Figure 57 ^{1}H NMR of 1i

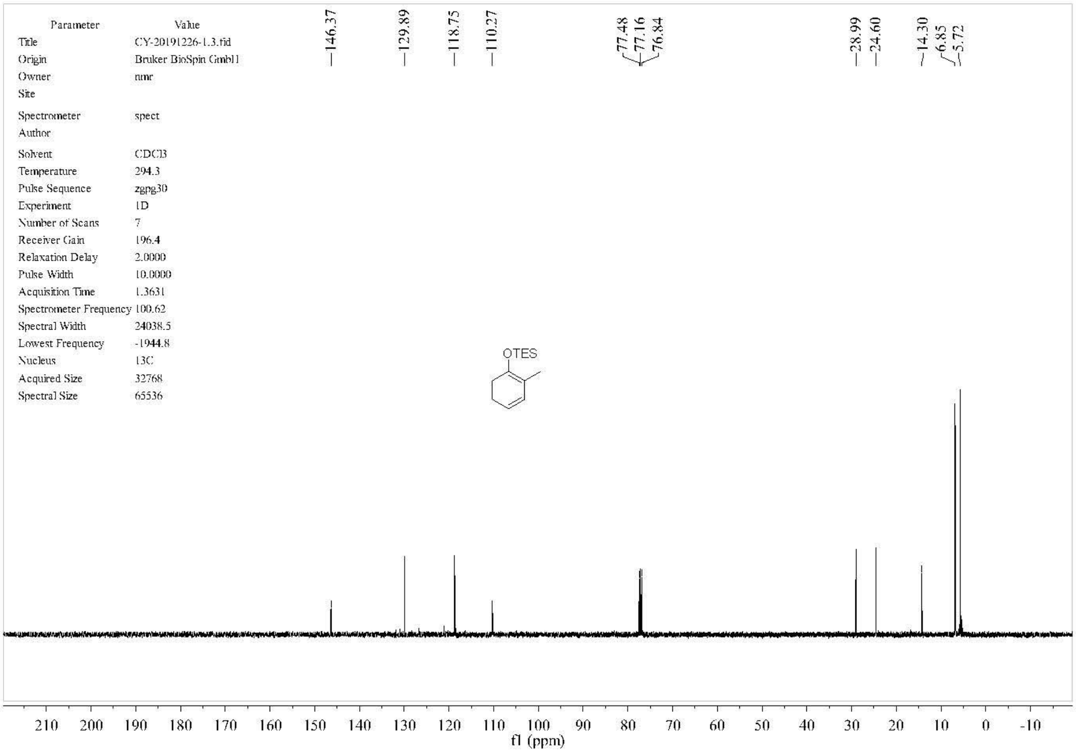

Supplementary Figure 58 ^{13}C NMR of 1i

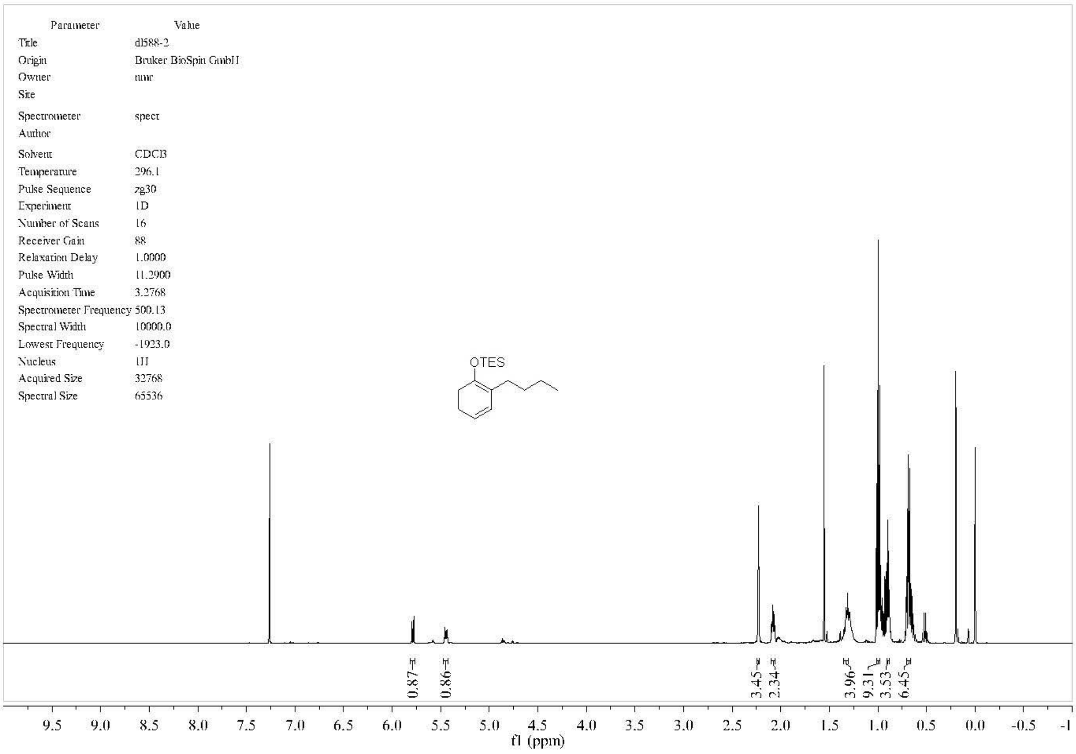

Supplementary Figure 59 ^{1}H NMR of 1j

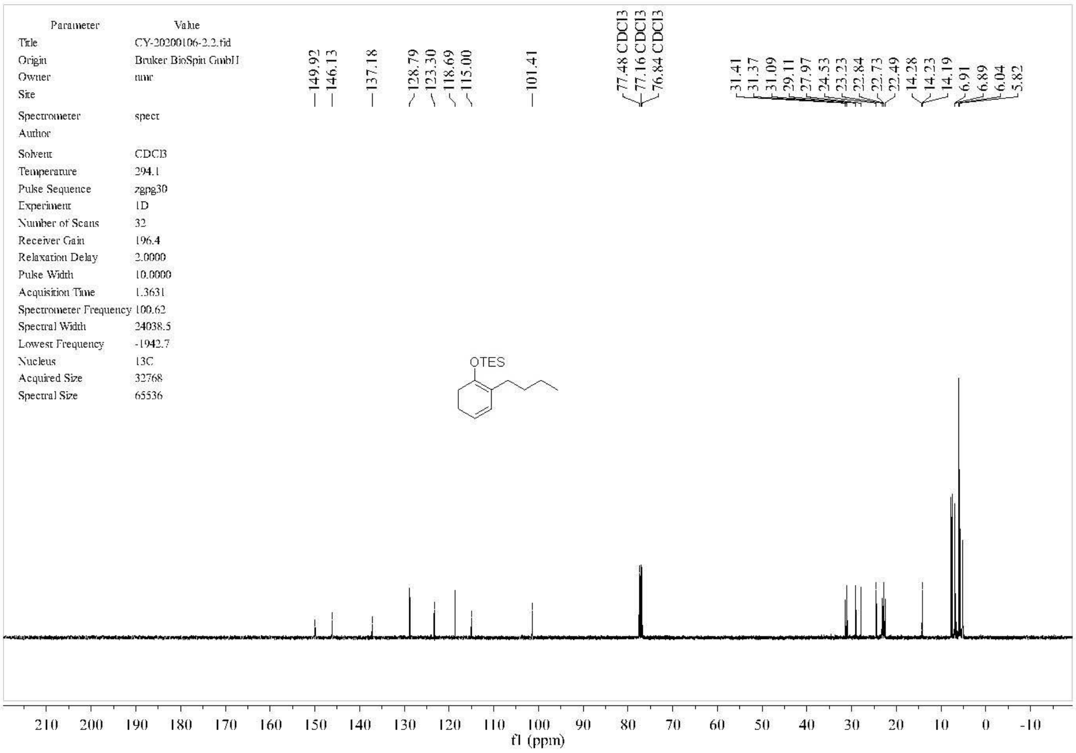

Supplementary Figure 60 ^{13}C NMR of 1j

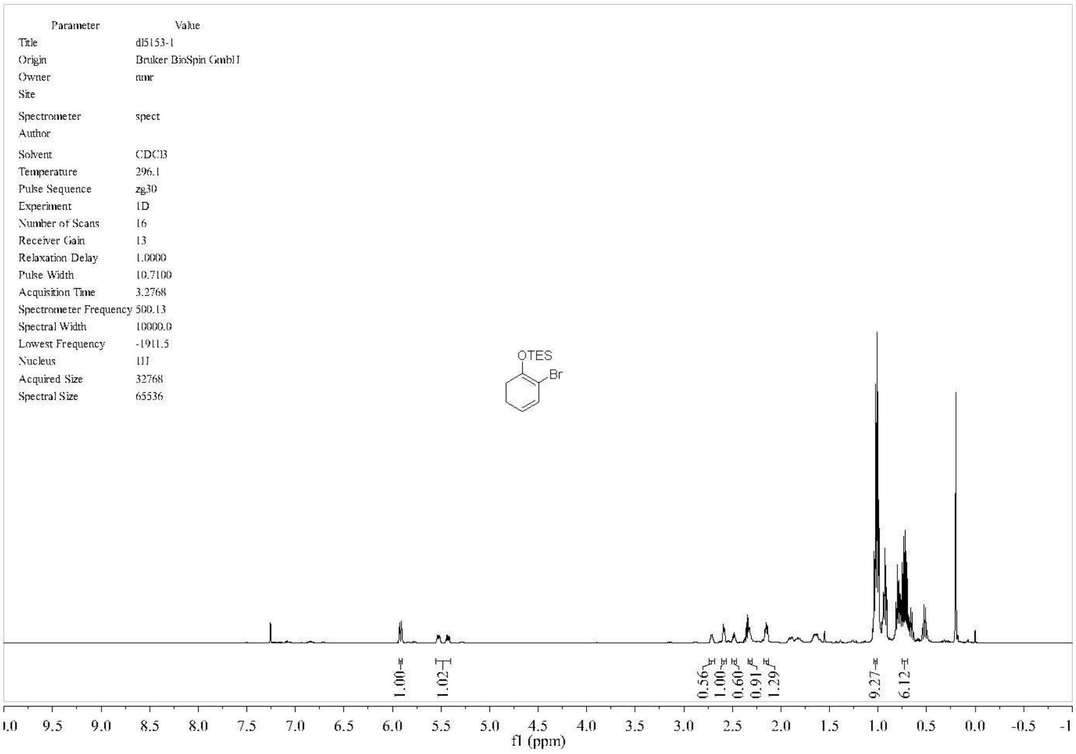

Supplementary Figure 61 ^{1}H NMR of 1k

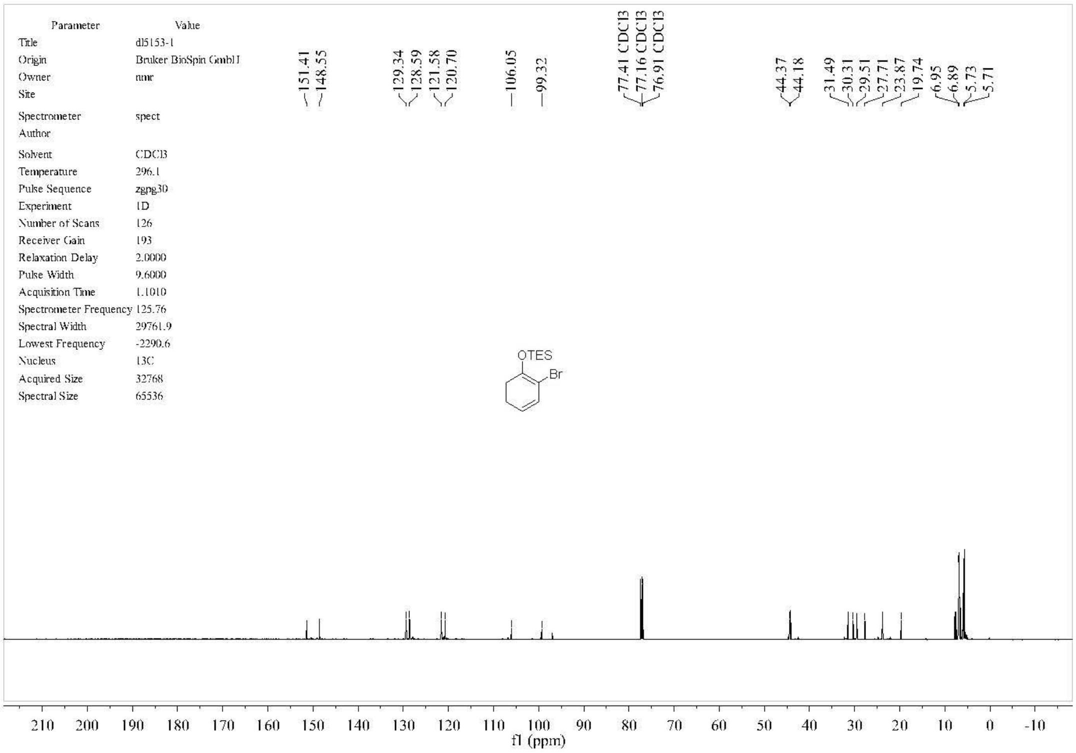

Supplementary Figure 62 ^{13}C NMR of 1k

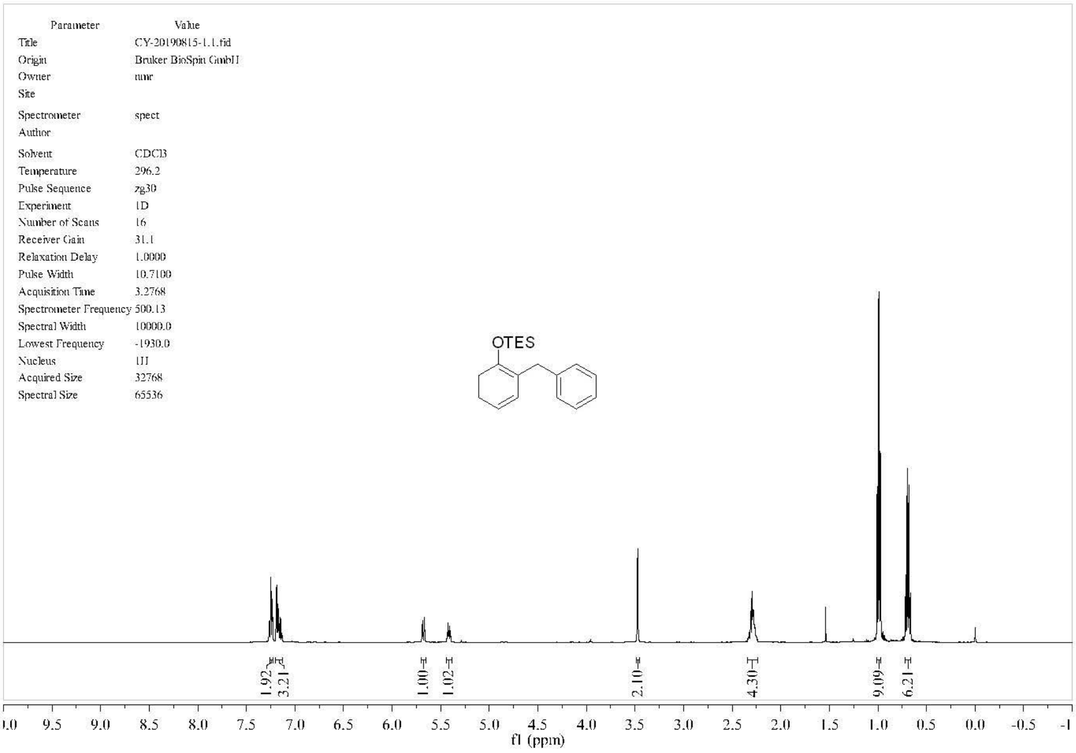

Supplementary Figure 63 ^{1}H NMR of 1l

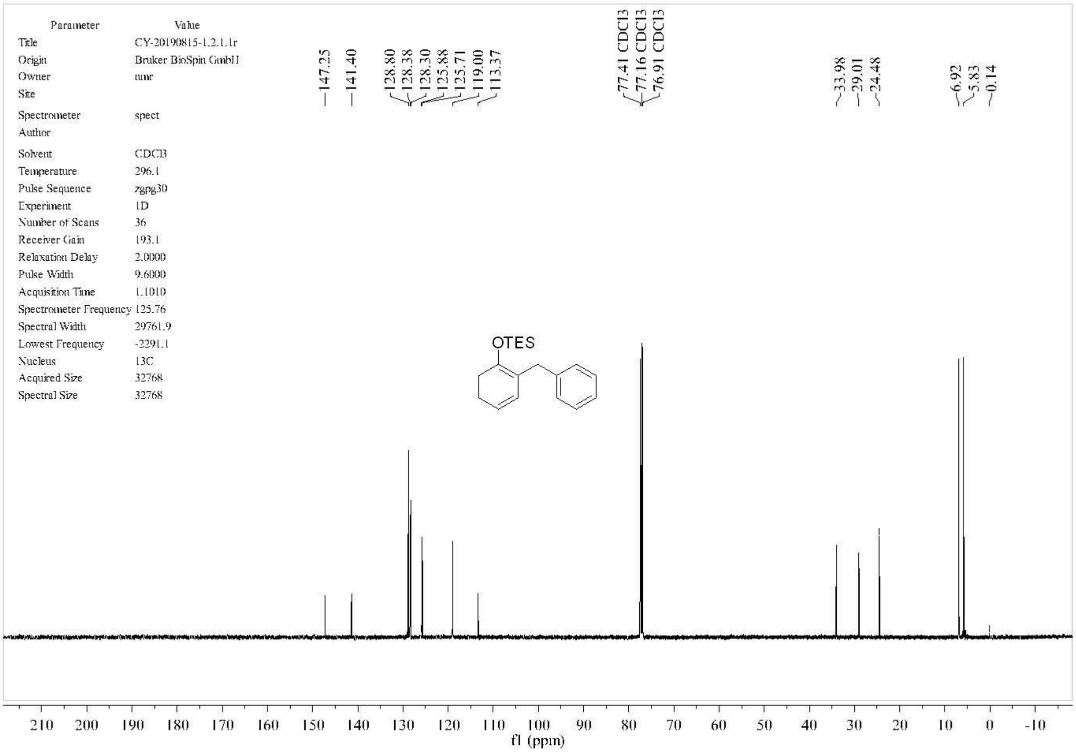

Supplementary Figure 64 ^{13}C NMR of 1l

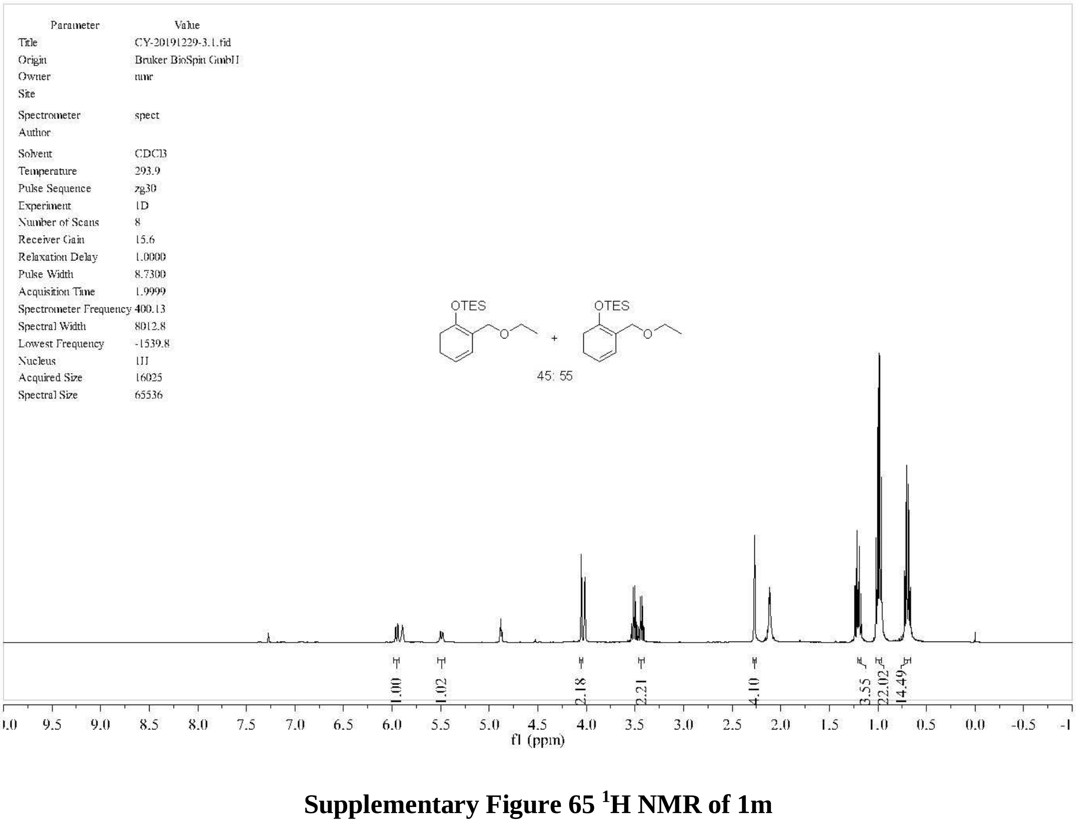

Supplementary Figure 65 ^{1}H NMR of 1m

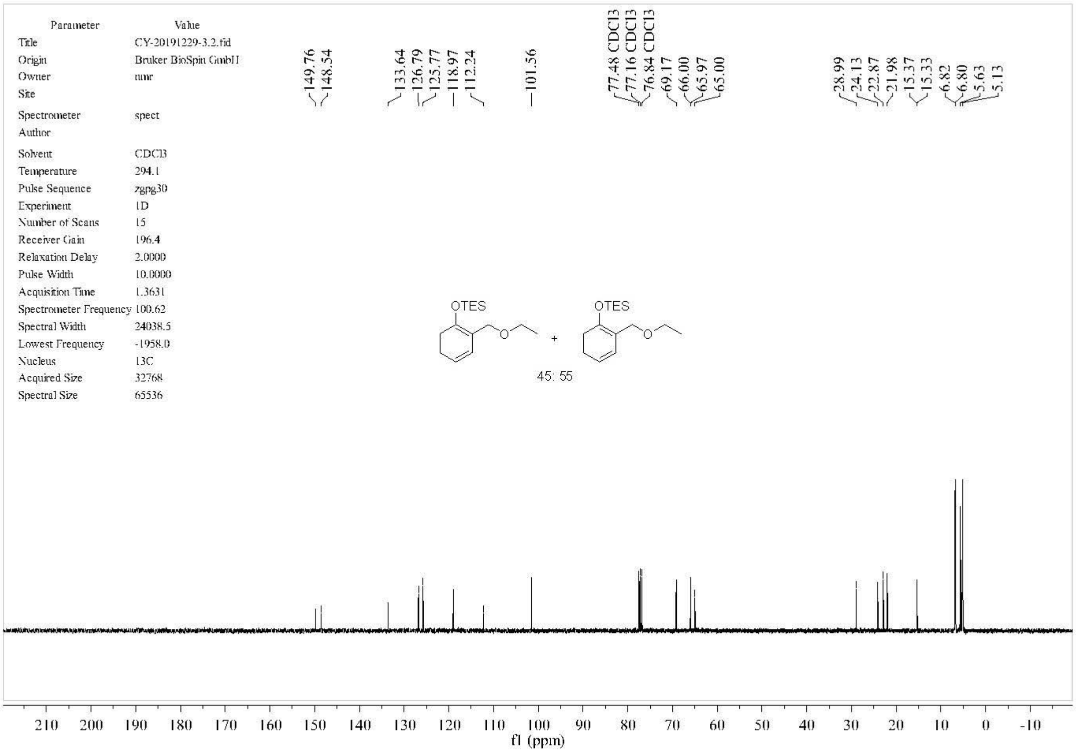

Supplementary Figure 66 ^{13}C NMR of 1m

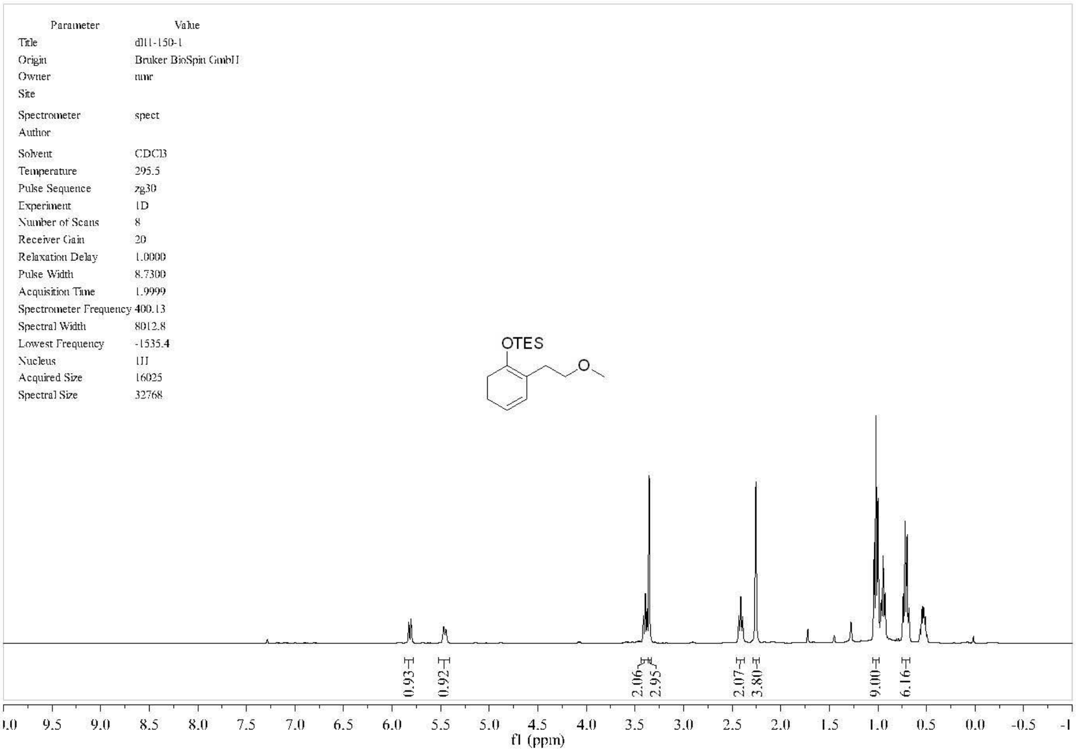

Supplementary Figure 67 ^{1}H NMR of 1n

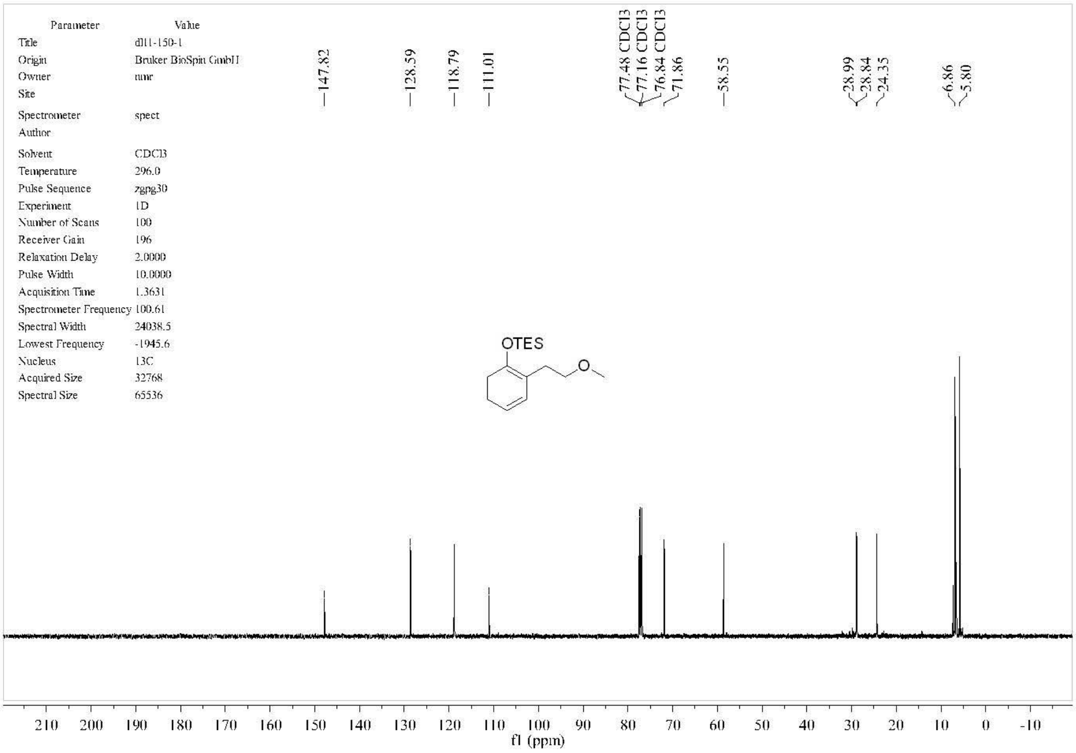

Supplementary Figure 68 ^{13}C NMR of 1n

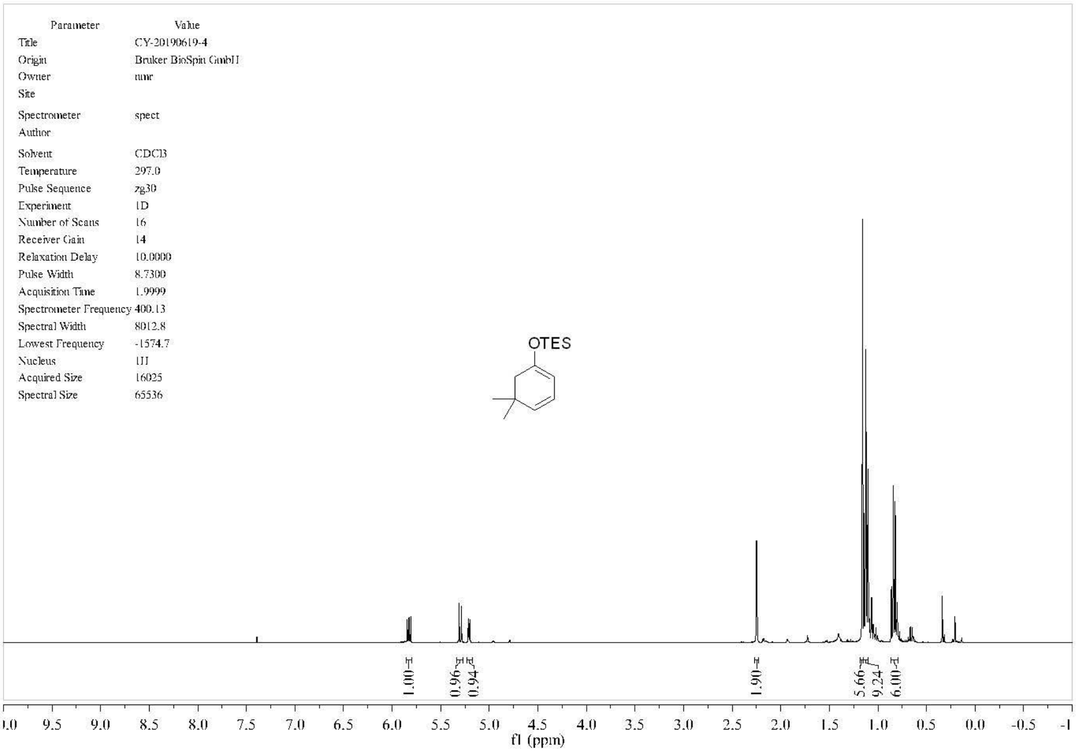

Supplementary Figure 69 ^{1}H NMR of 1q

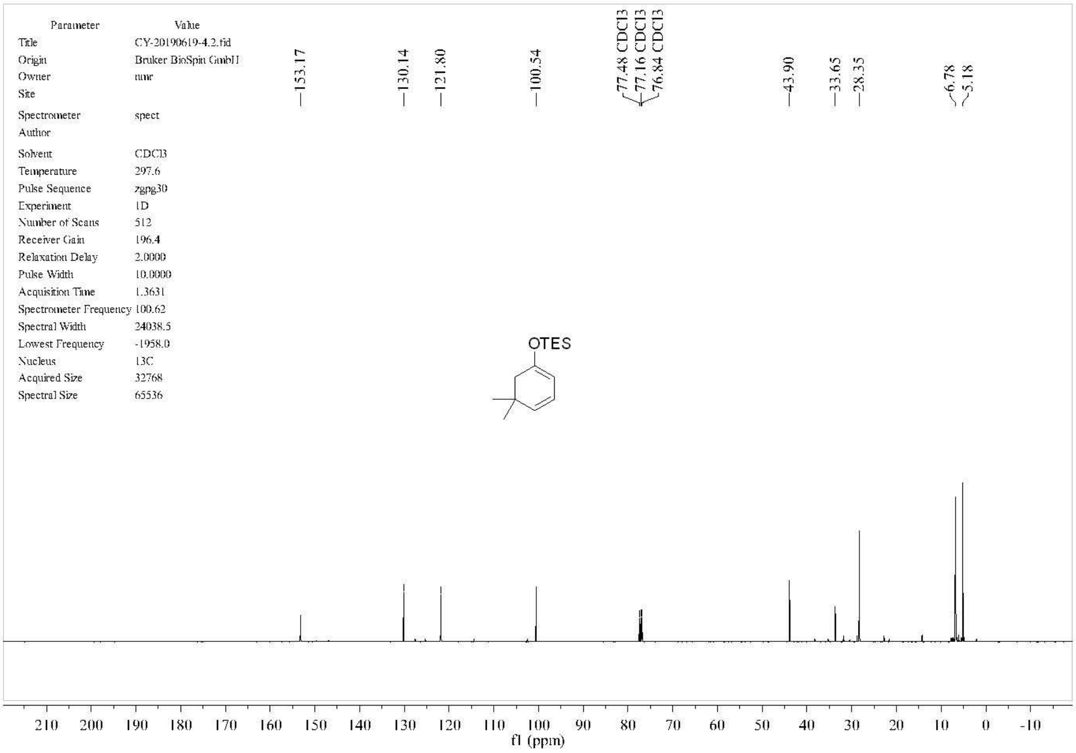

Supplementary Figure 70 ^{13}C NMR of 1q

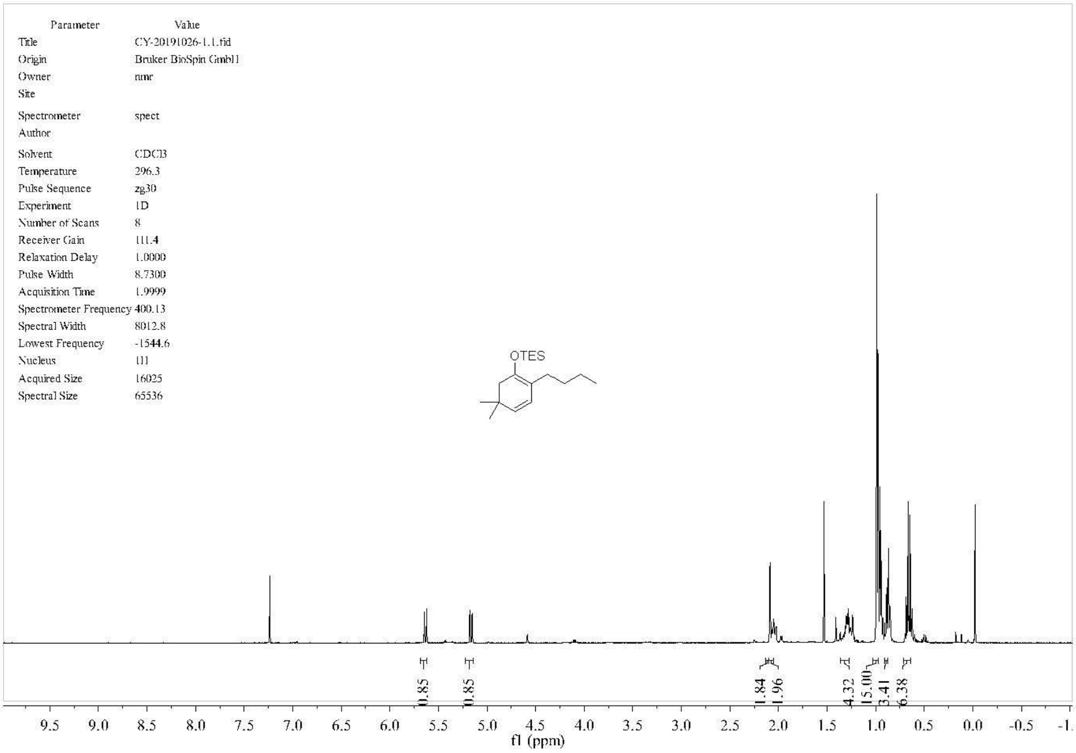

Supplementary Figure 71 ^{1}H NMR of 1r

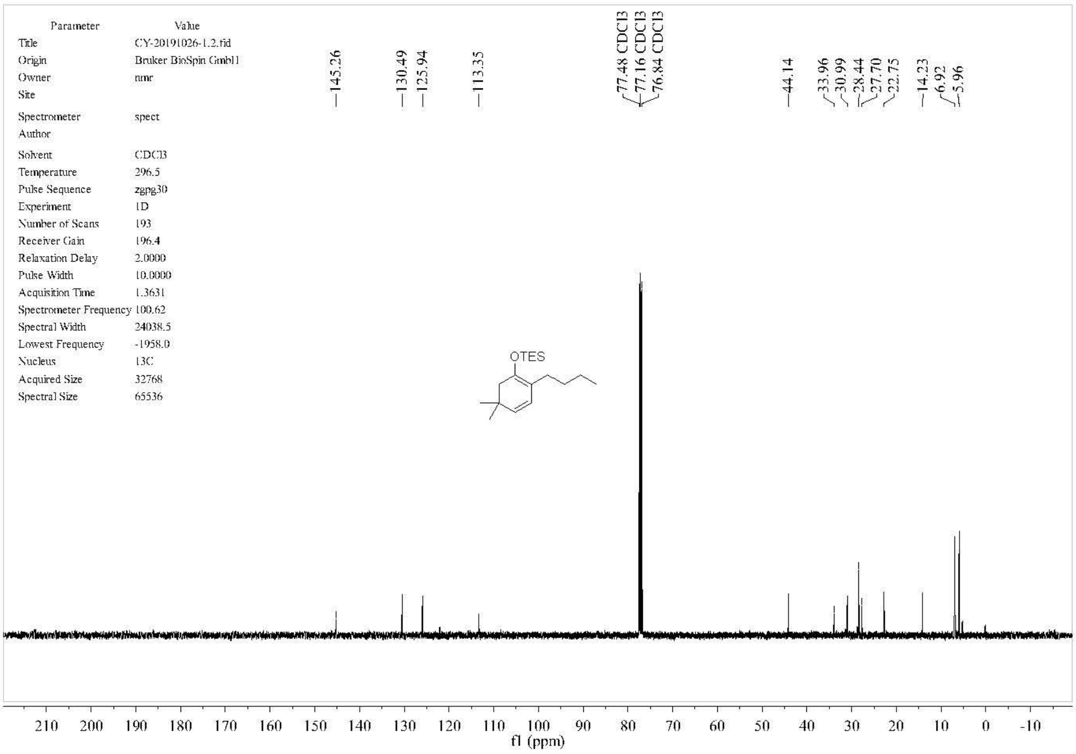

Supplementary Figure 72 ^{13}C NMR of 1r

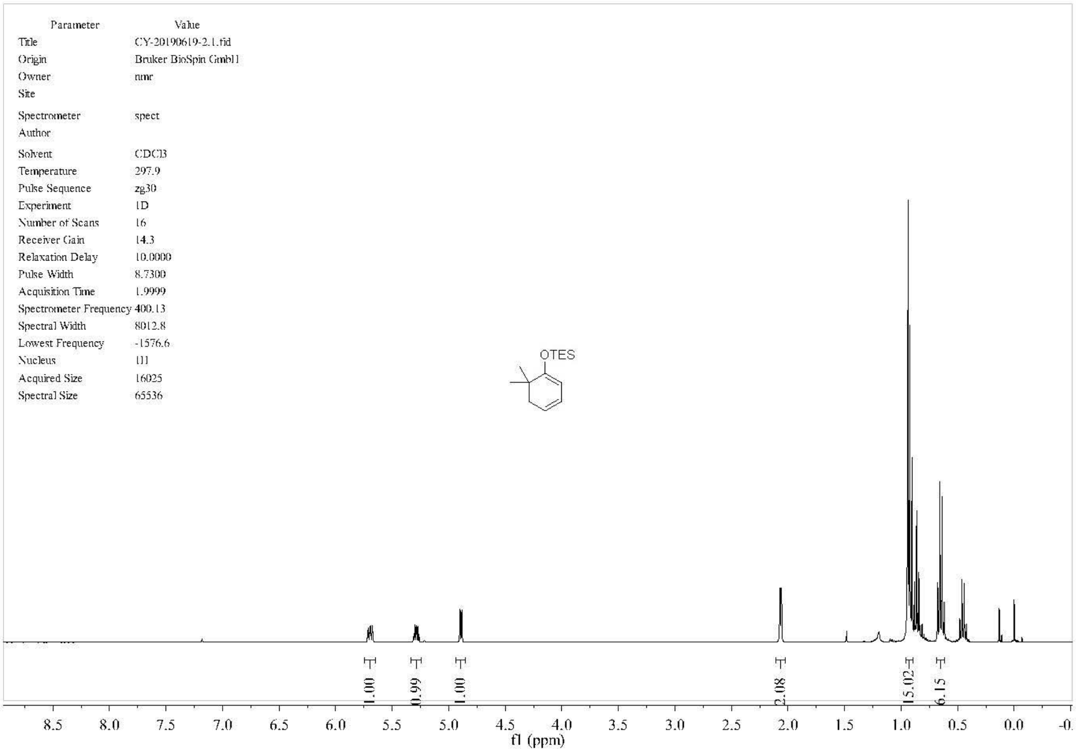

Supplementary Figure 73 ^{1}H NMR of 1s

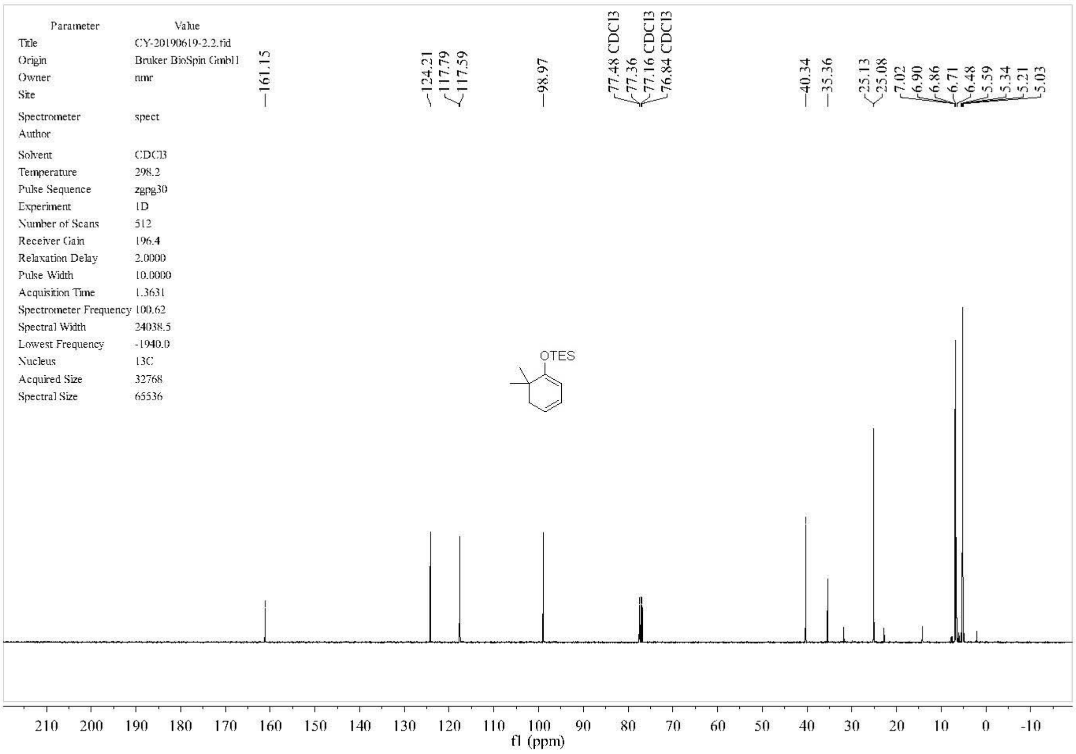

Supplementary Figure 74 ^{13}C NMR of 1s

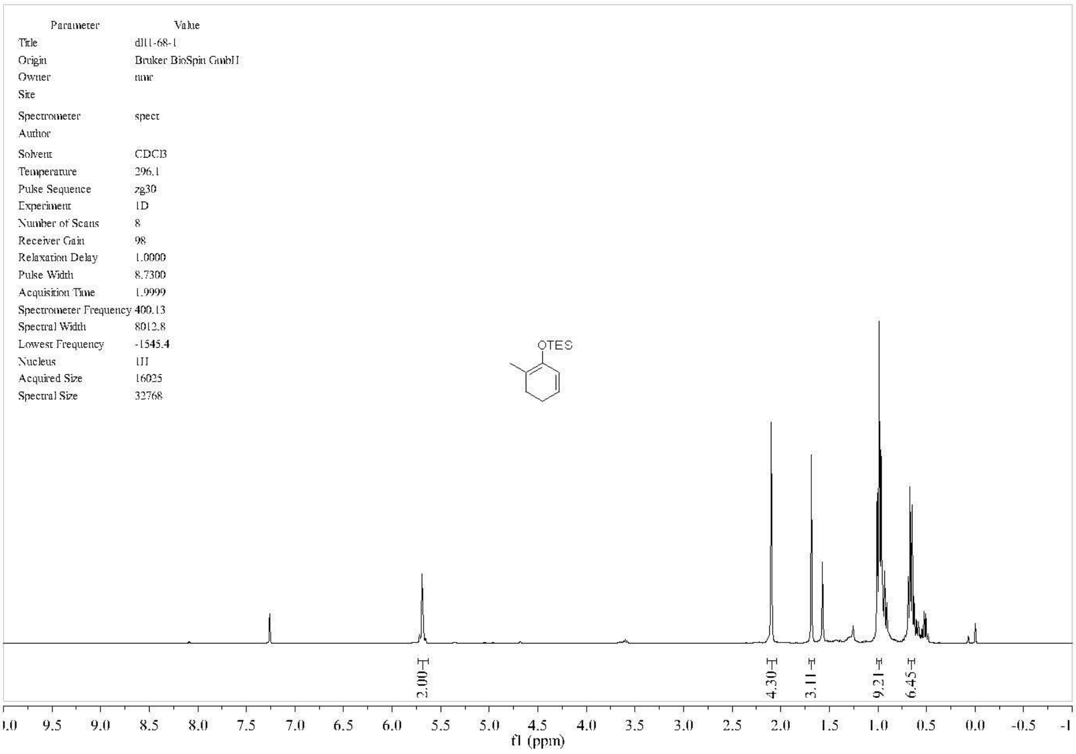

Supplementary Figure 75 ^{1}H NMR of 1g

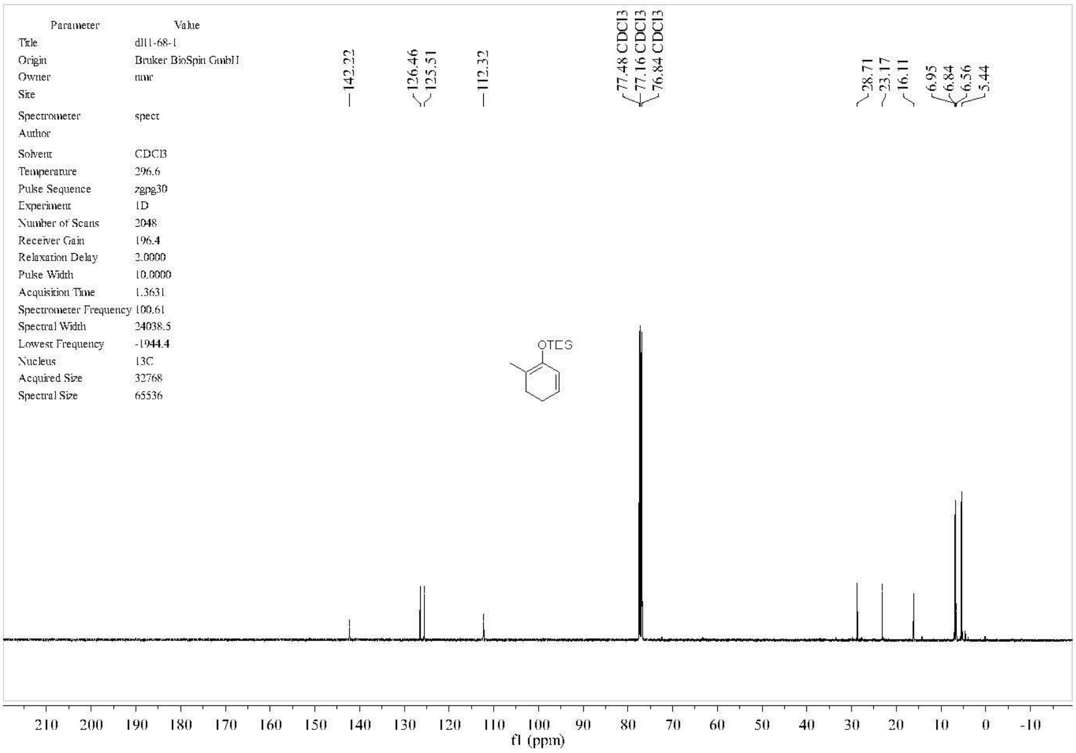

Supplementary Figure 76 ^{13}C NMR of 1g

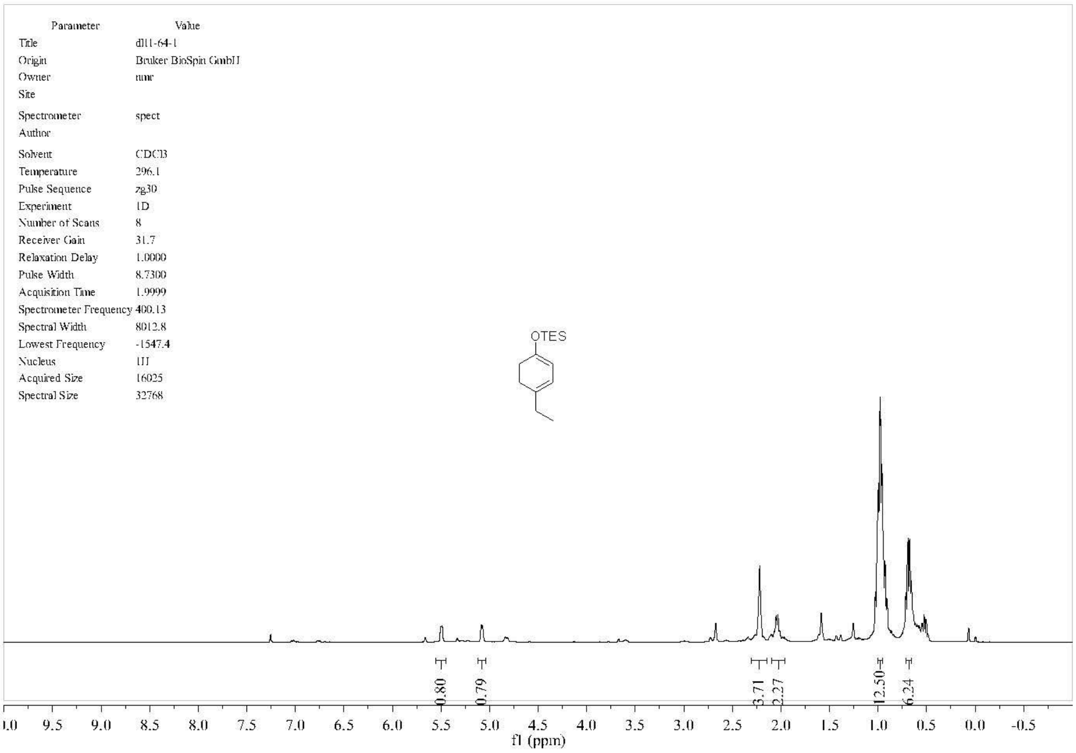

Supplementary Figure 77 ^{1}H NMR of 1p

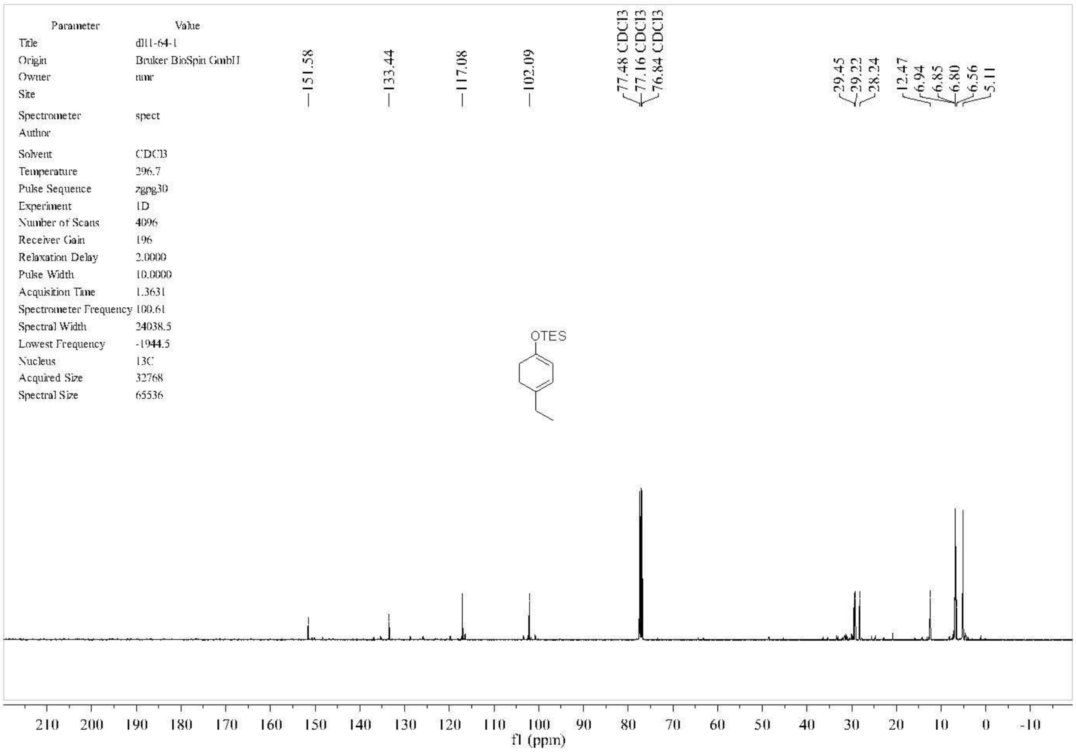

Supplementary Figure 78 ^{13}C NMR of 1p

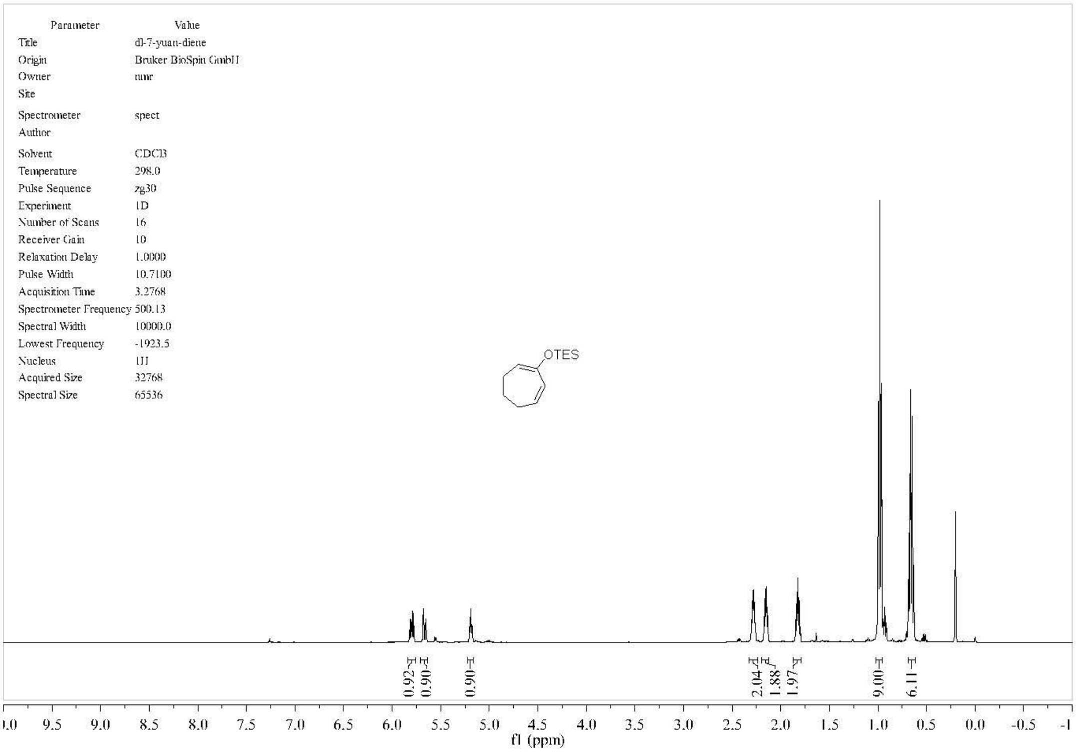

Supplementary Figure 79 ^{1}H NMR of 1t

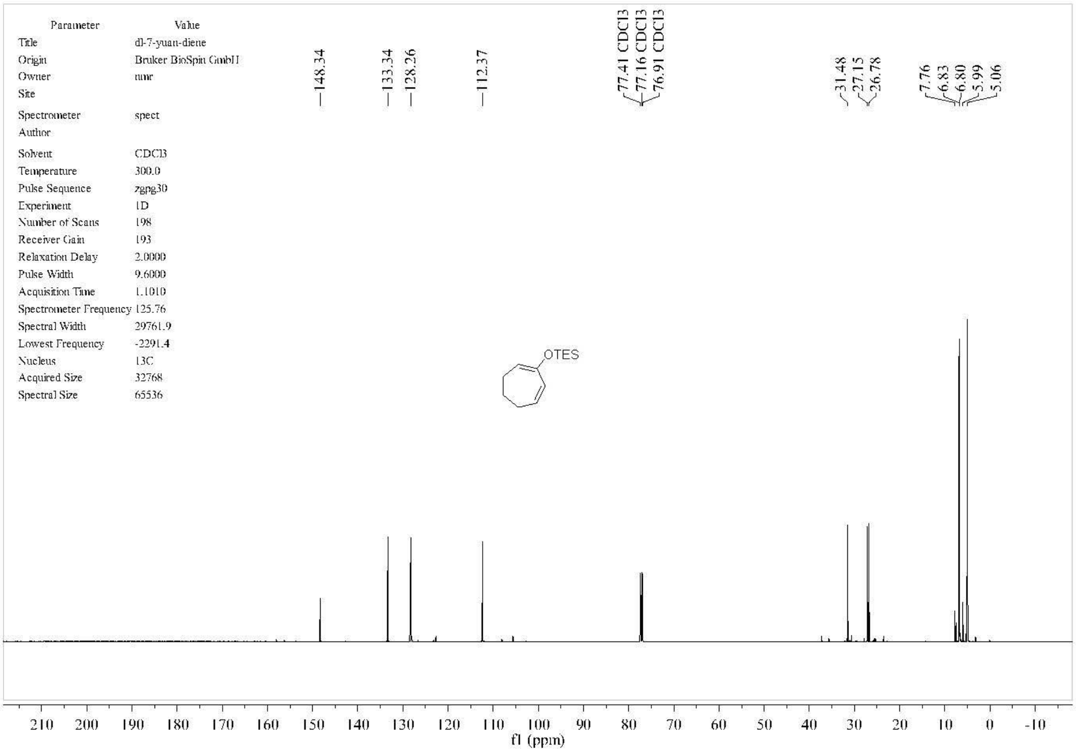

Supplementary Figure 80 ^{13}C NMR of 1t

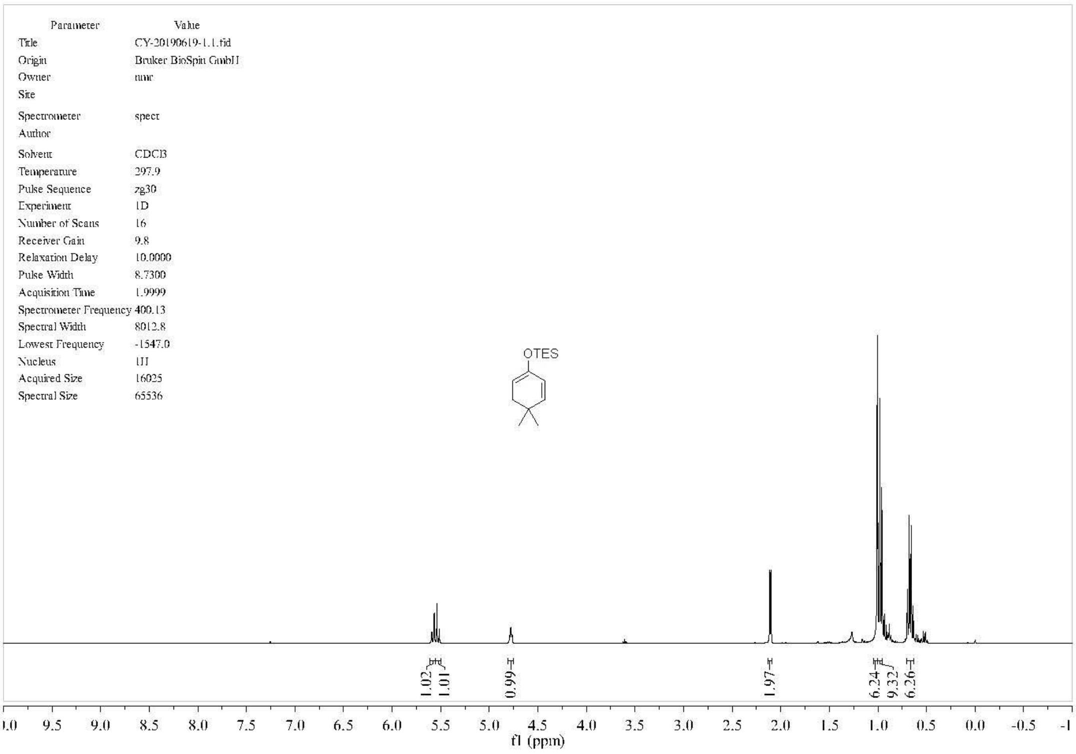

Supplementary Figure 81 ^{1}H NMR of 1u

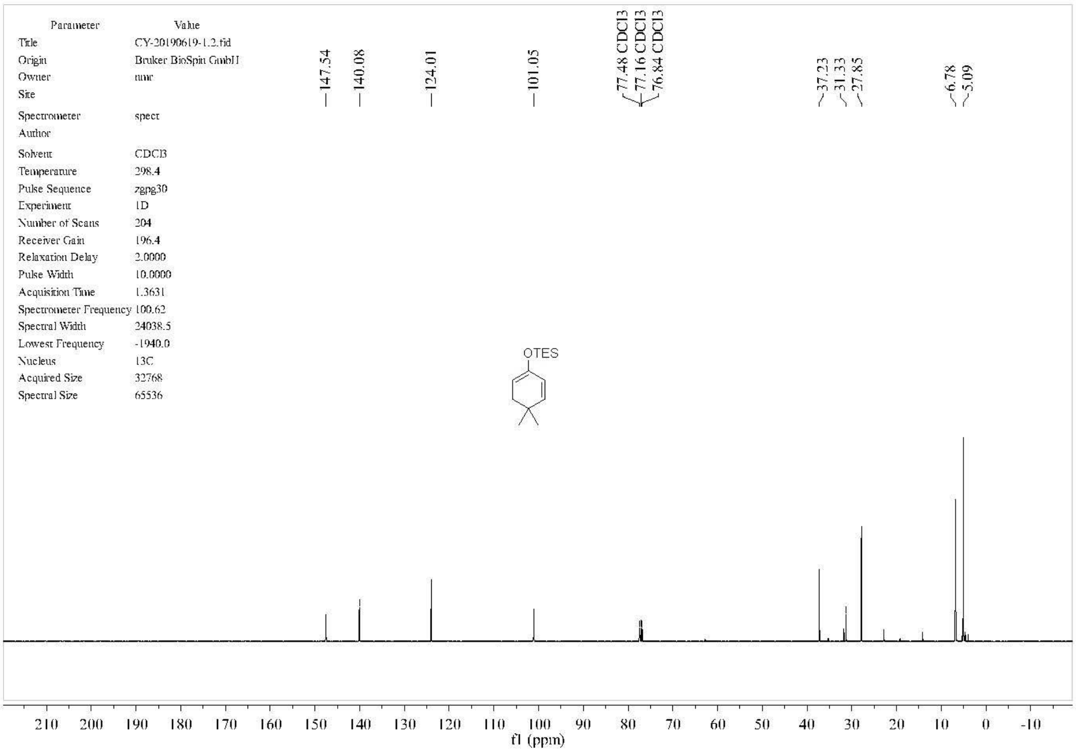

Supplementary Figure 82 ^{13}C NMR of 1u

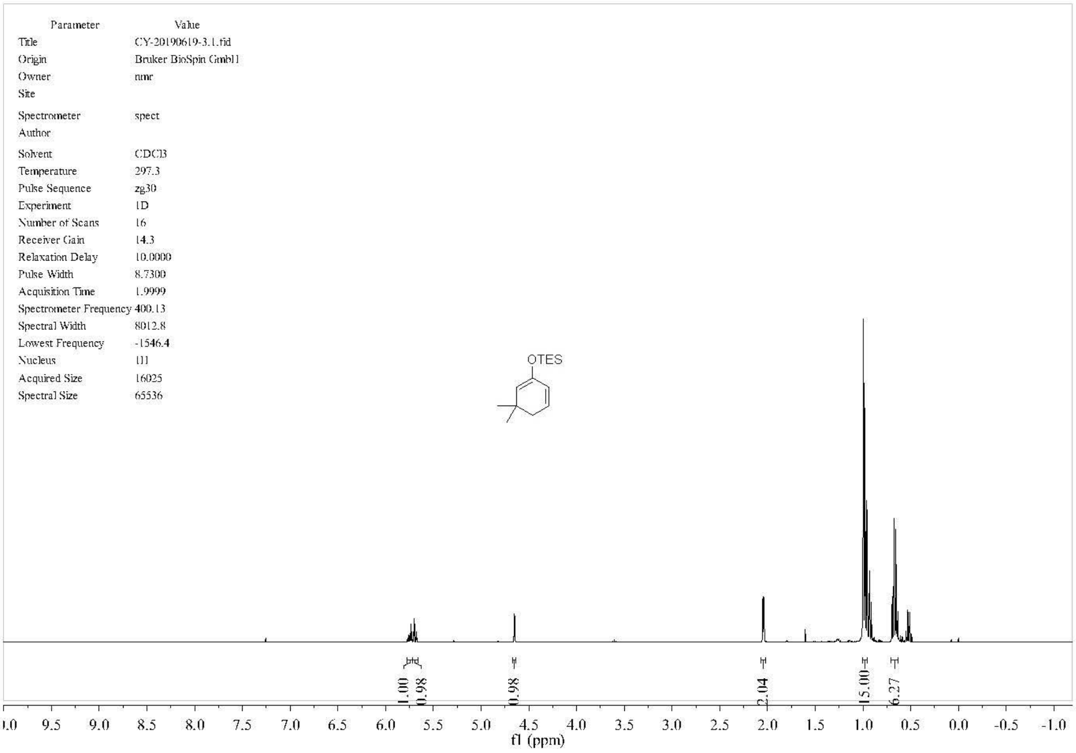

Supplementary Figure 83 ^{1}H NMR of 1v

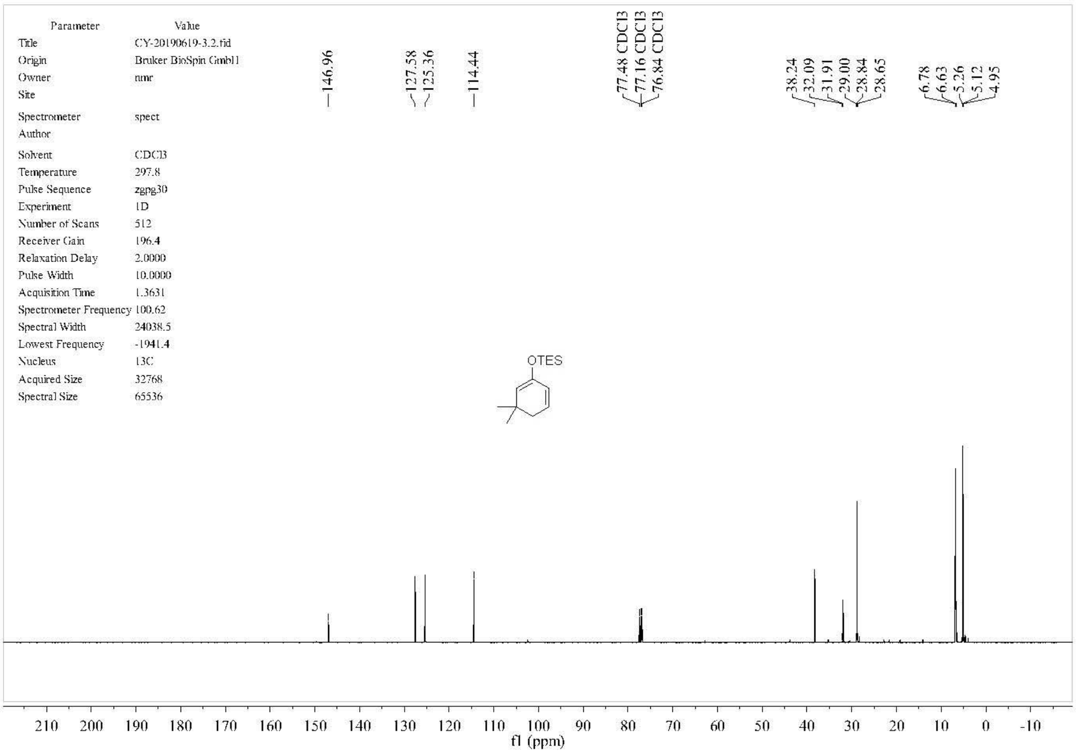

Supplementary Figure 84 ^{13}C NMR of 1v

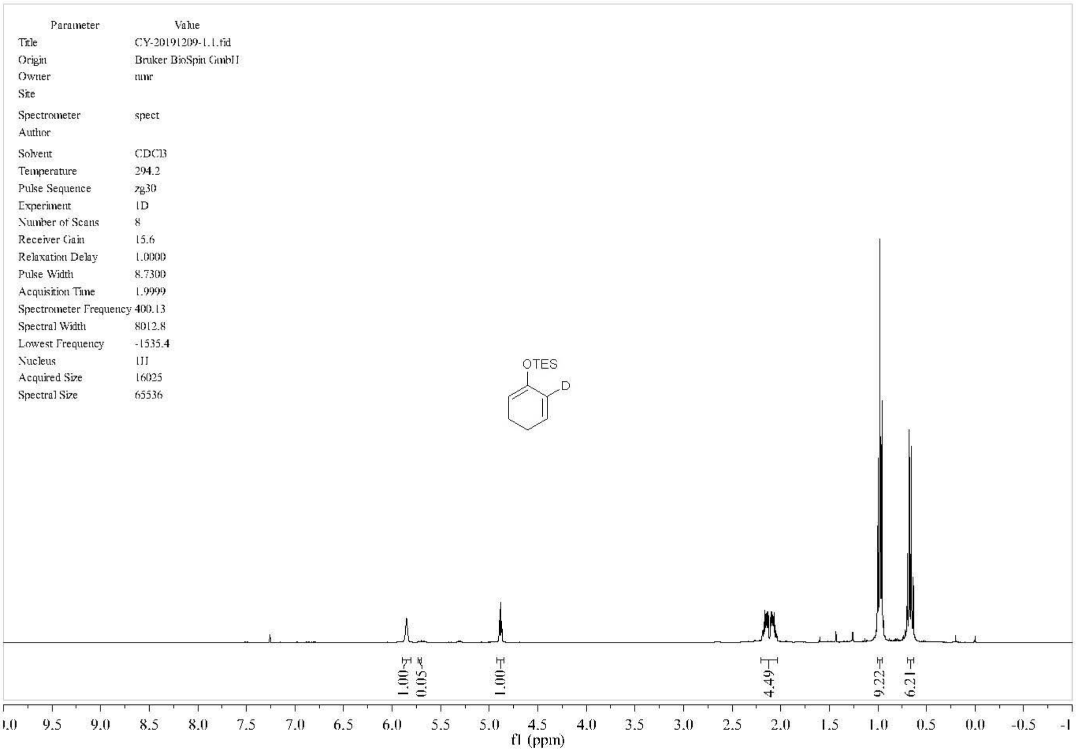

Supplementary Figure 85 ^{1}H NMR of D-1a

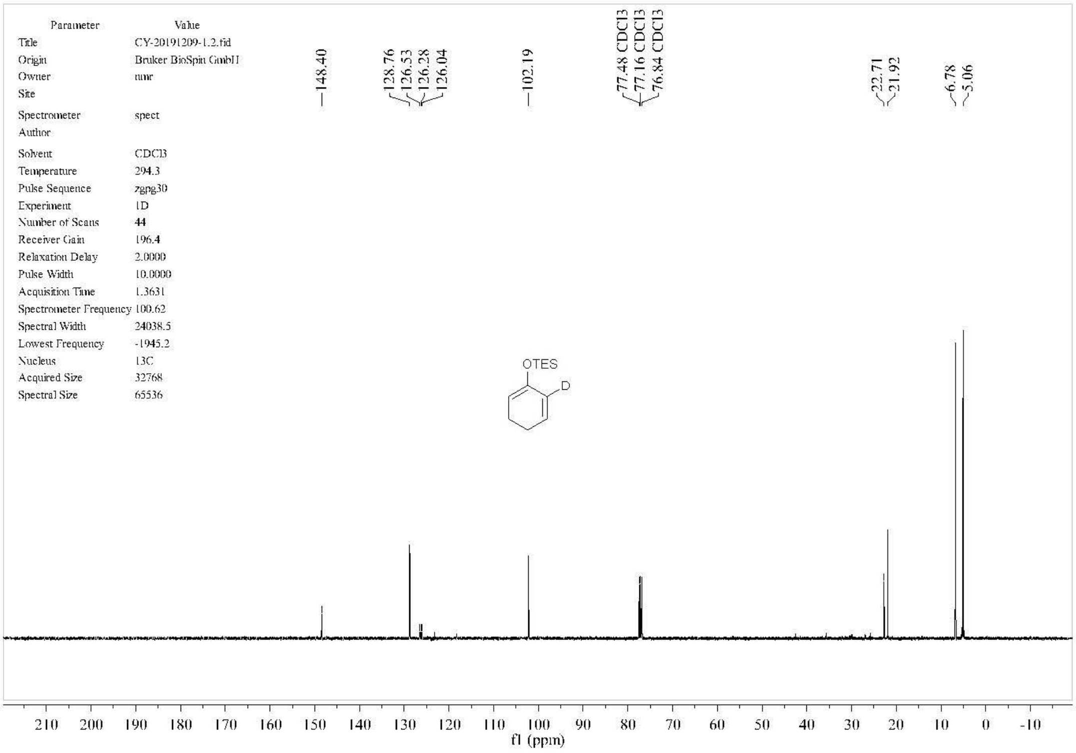

Supplementary Figure 86 ^{13}C NMR of D-1a

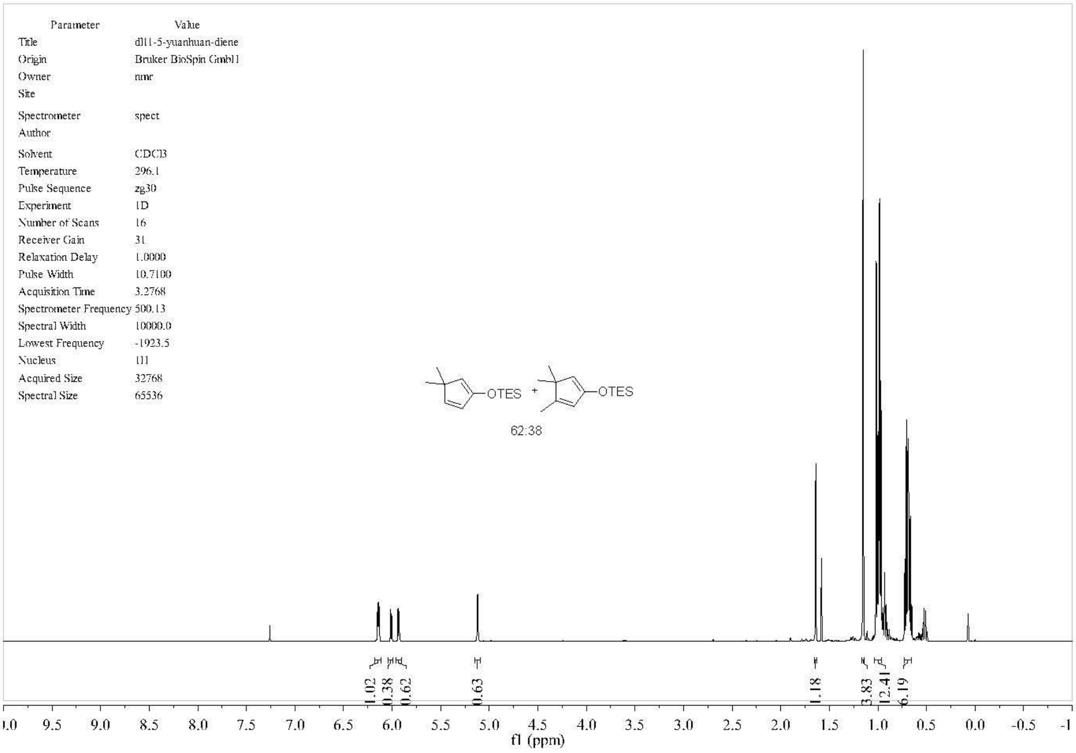

Supplementary Figure 87 ^{1}H NMR of 1w

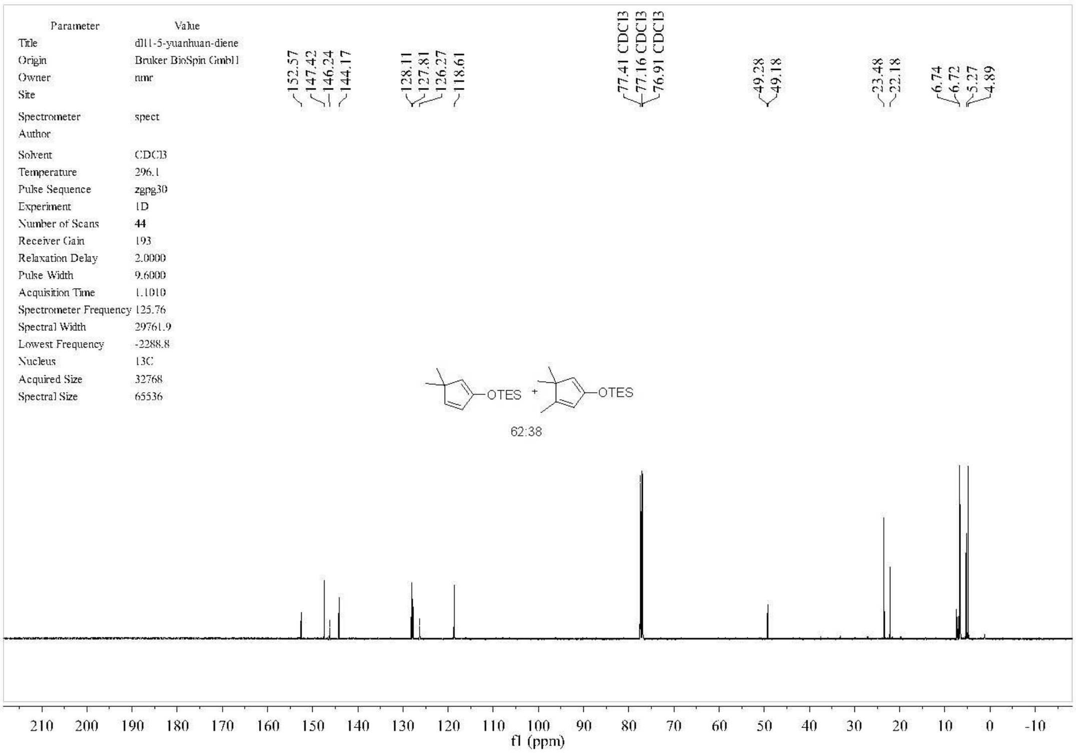

Supplementary Figure 88 ^{13}C NMR of 1w

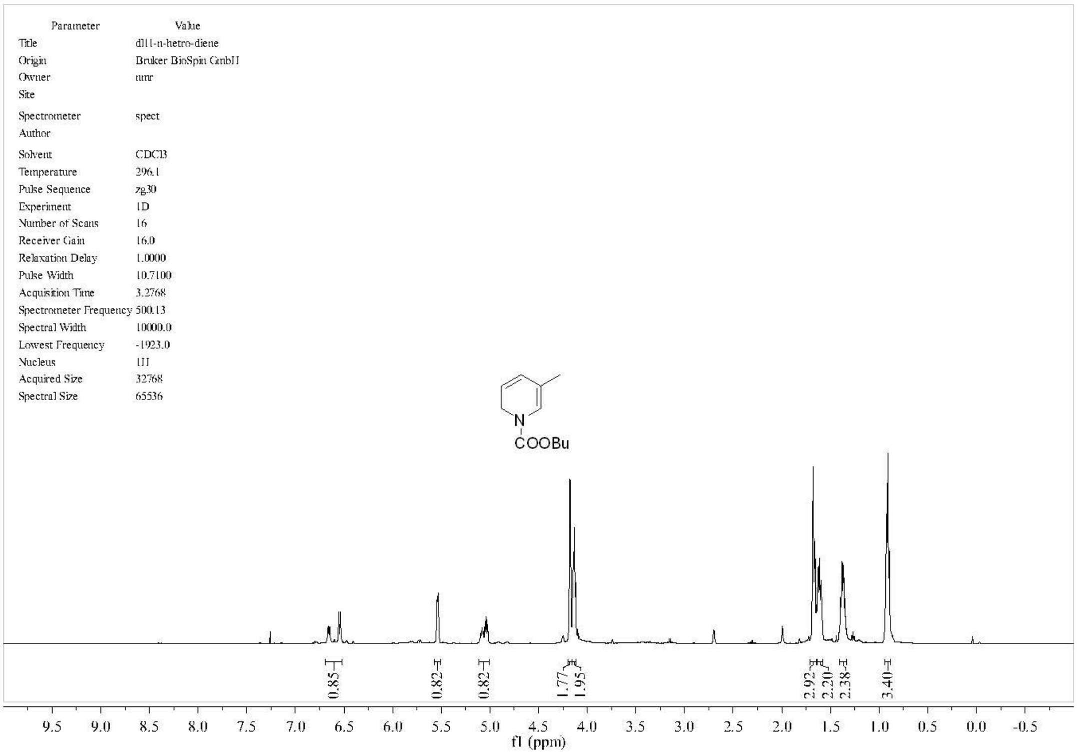

Supplementary Figure 89 ^{1}H NMR of 1o

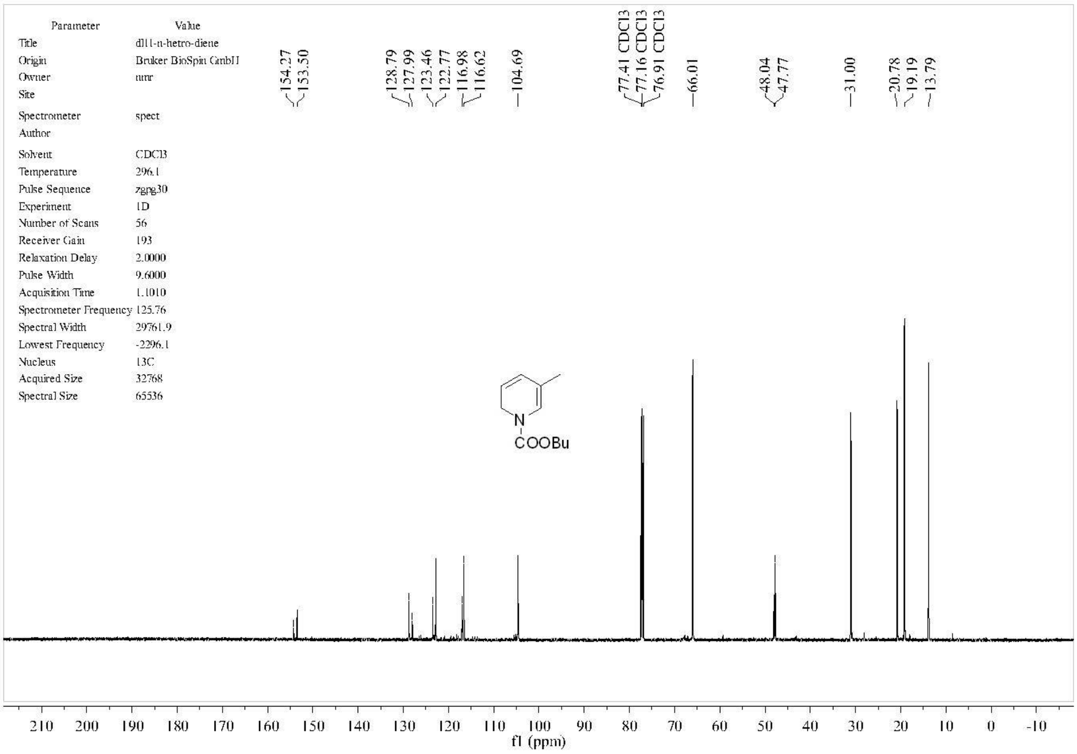

Supplementary Figure 90 ^{13}C NMR of 1o

Cross hydroalkenylation Product

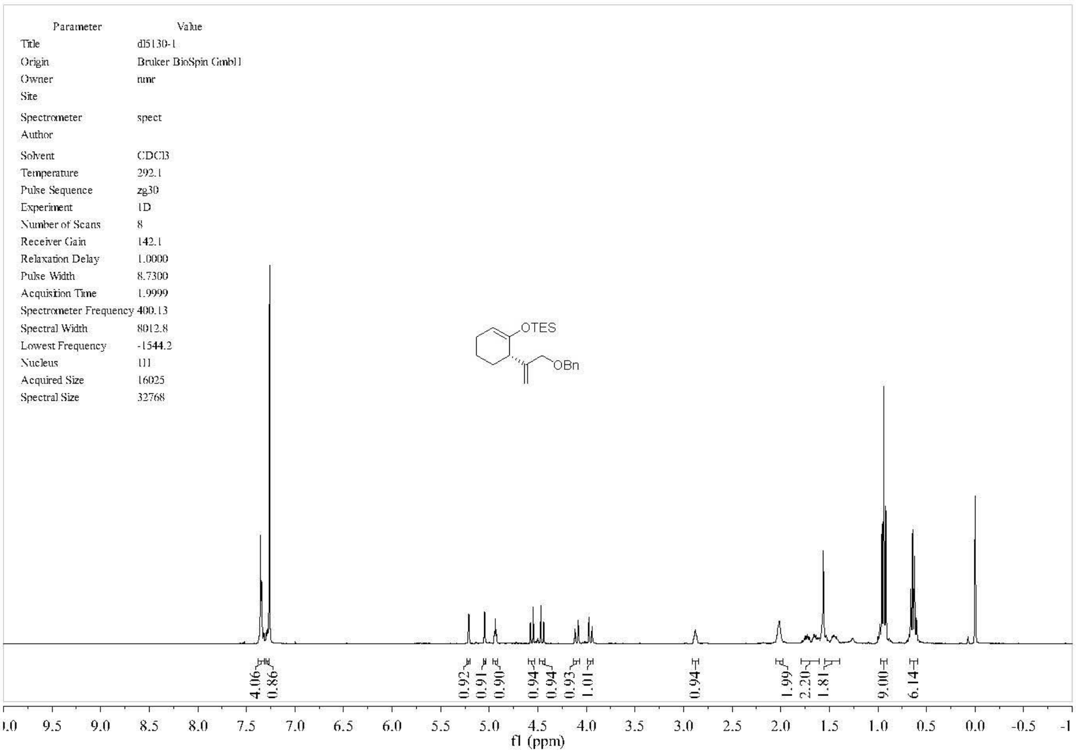

Supplementary Figure 91 ^{1}H NMR of 3aa

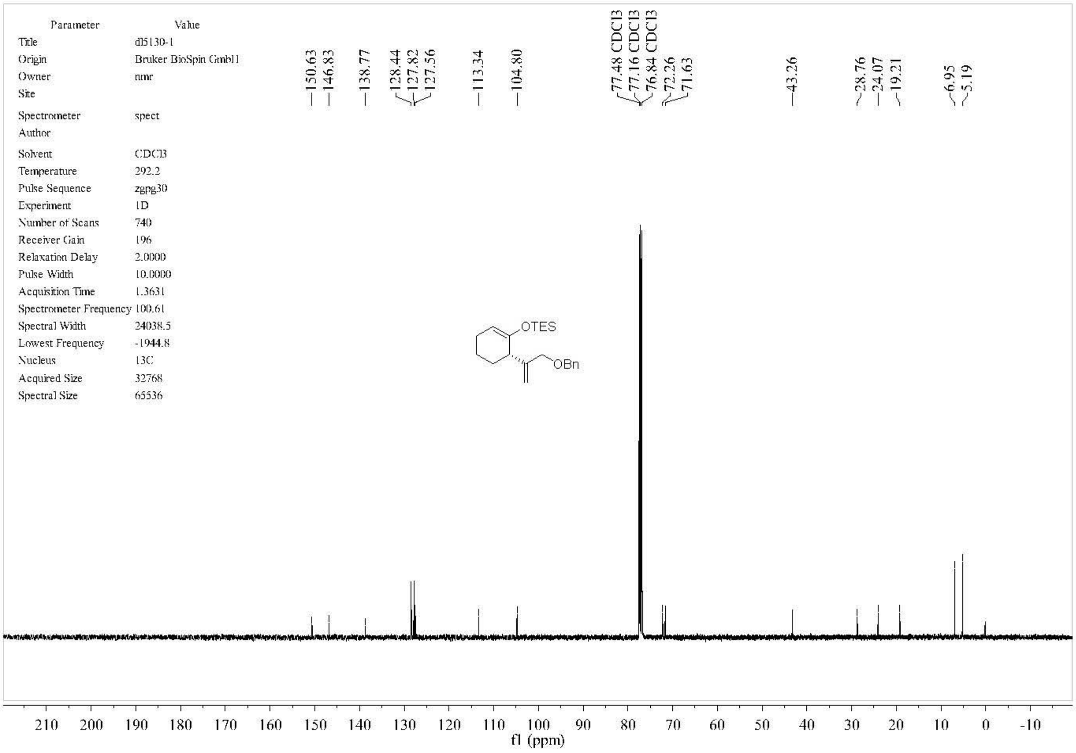

Supplementary Figure 92 ^{13}C NMR of 3aa

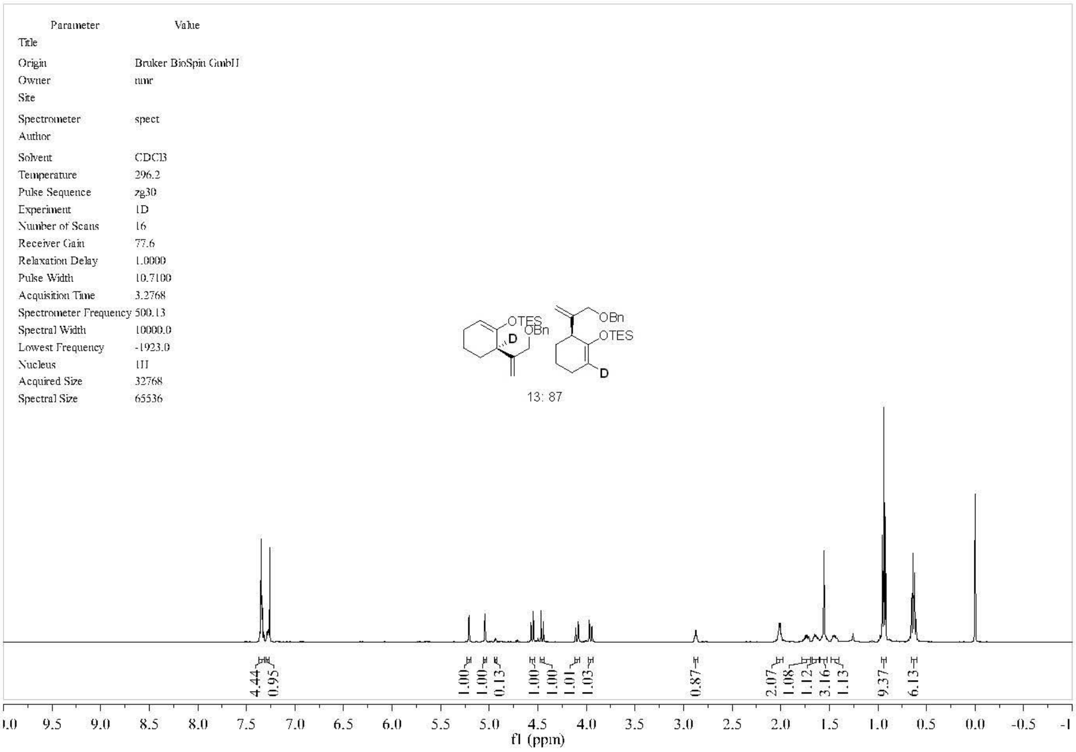

Supplementary Figure 93 ^{1}H NMR of D-3aa

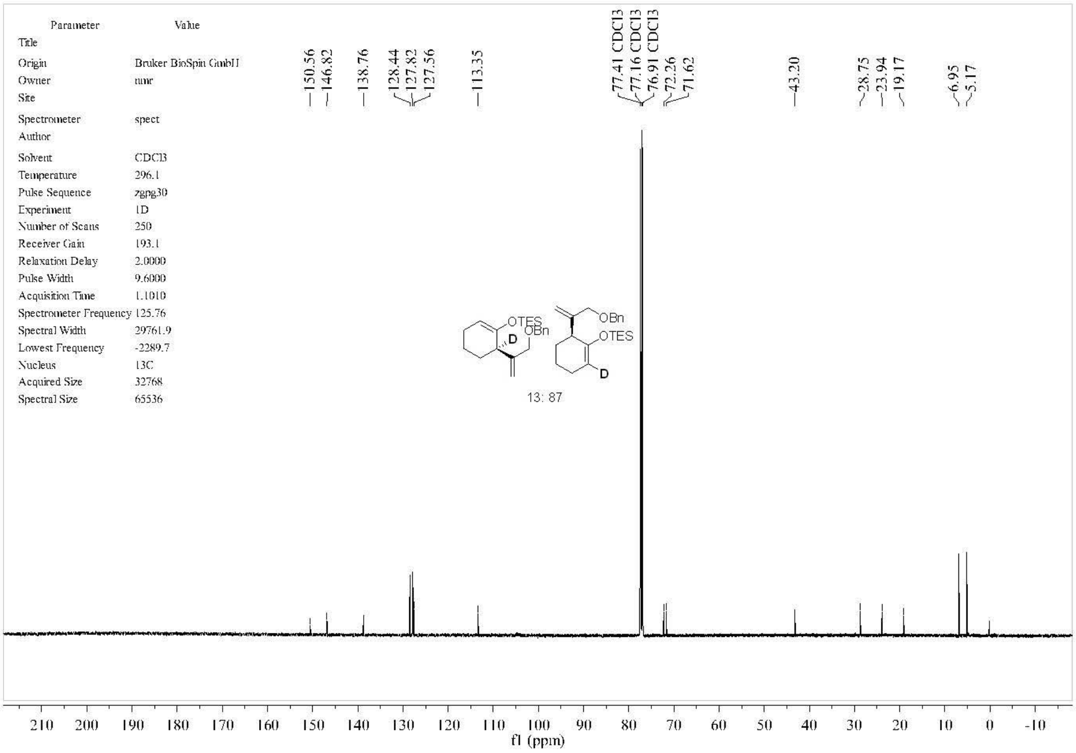

Supplementary Figure 94 ^{13}C NMR of D-3aa

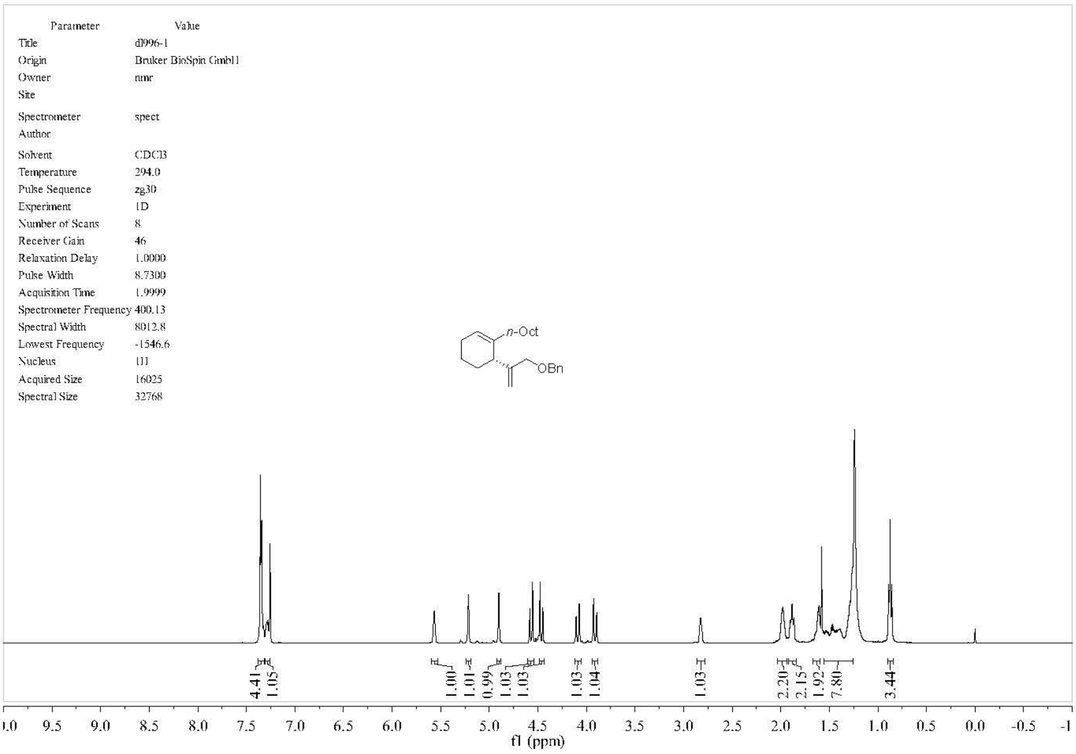

Supplementary Figure 95 ^{1}H NMR of 3ba

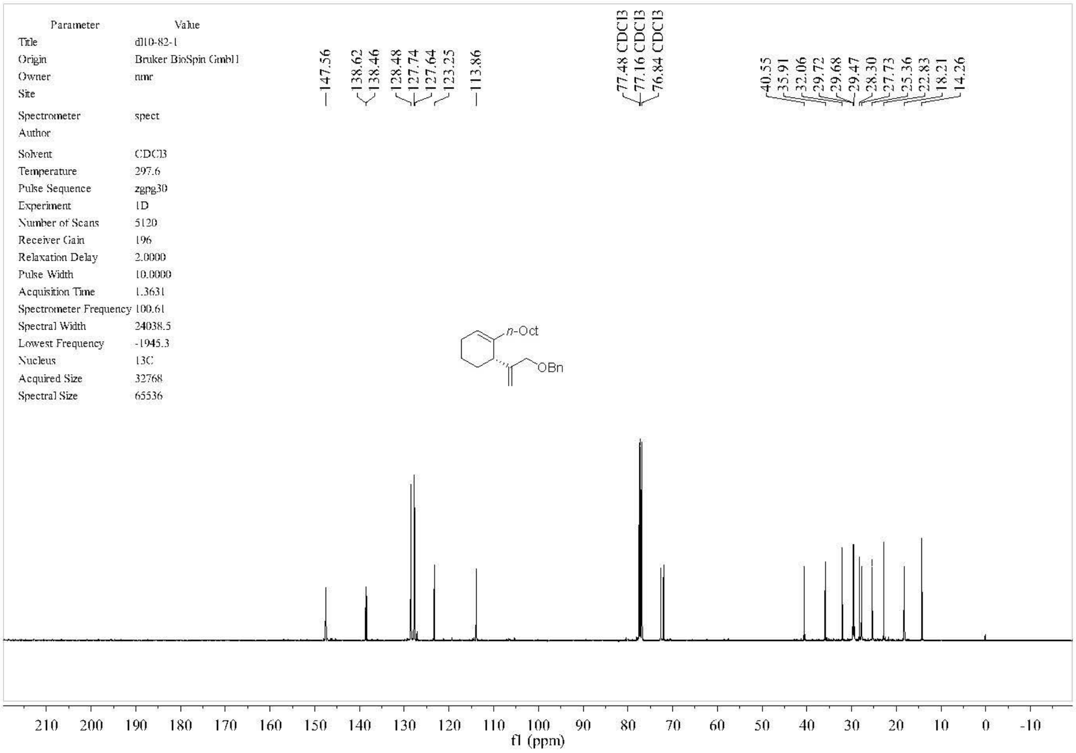

Supplementary Figure 96 ^{13}C NMR of 3ba

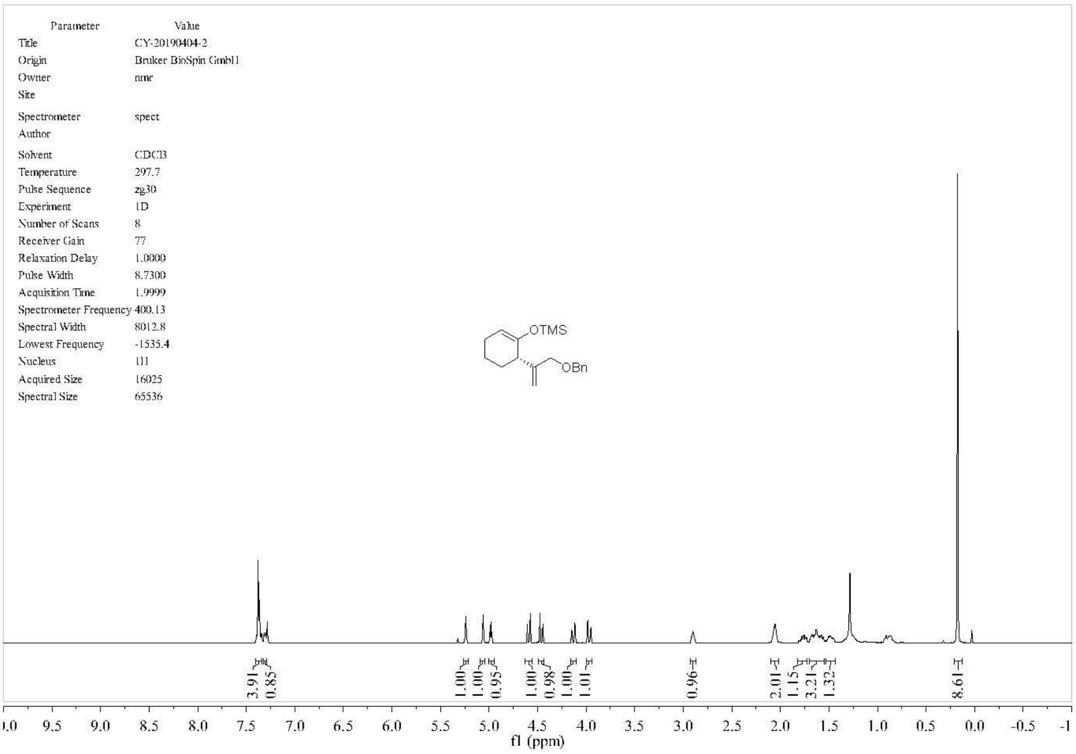

Supplementary Figure 97 ^{1}H NMR of 3ca

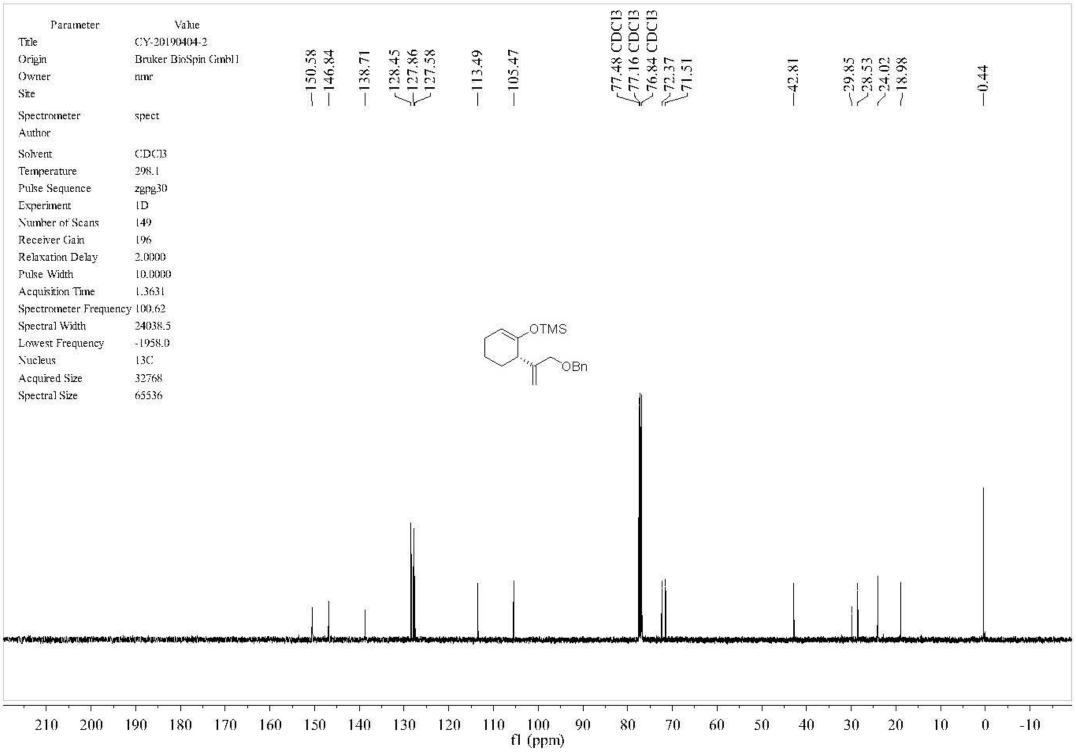

Supplementary Figure 98 ^{13}C NMR of 3ca

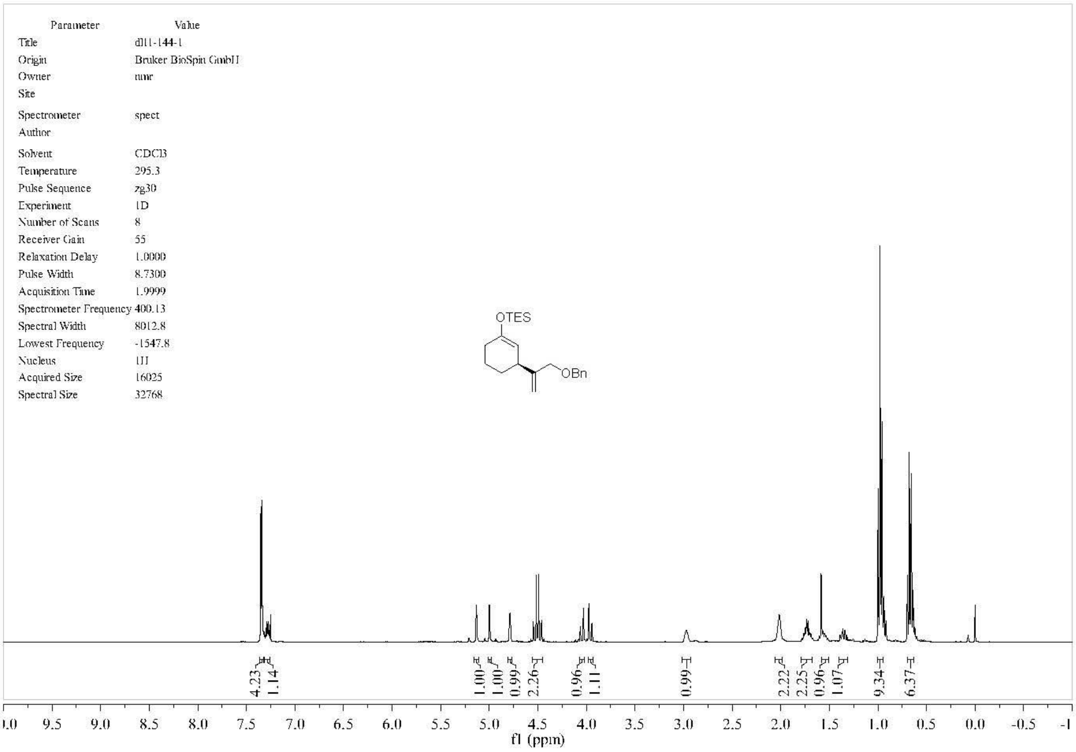

Supplementary Figure 99 ^{1}H NMR of 3da

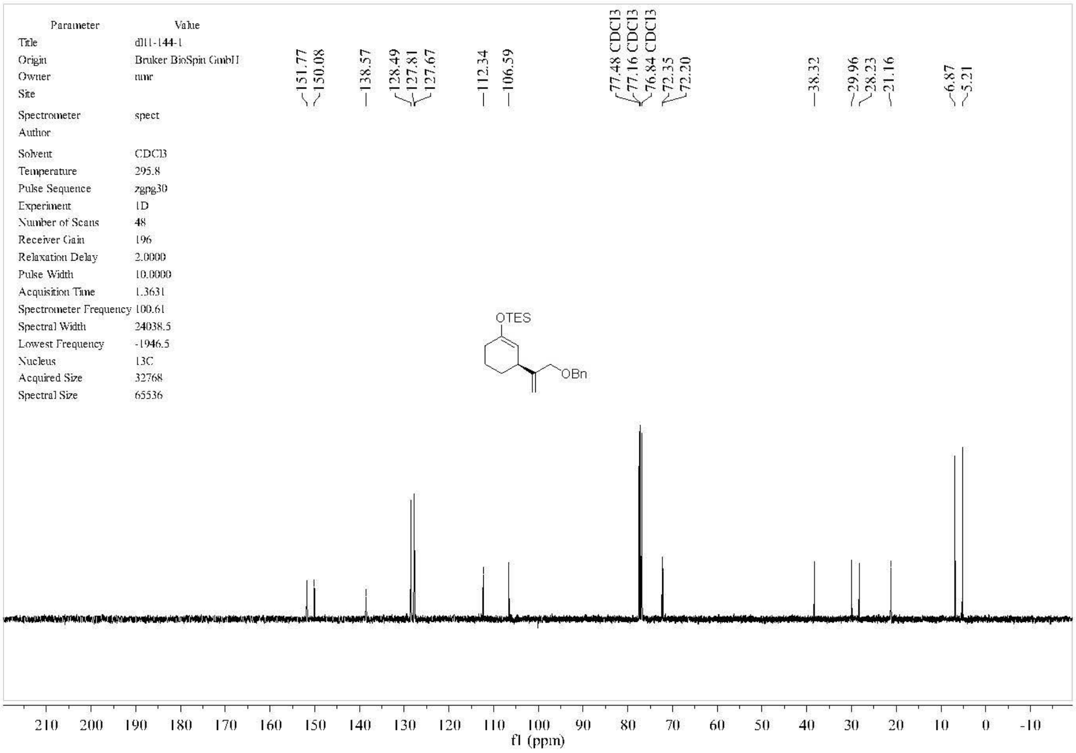

Supplementary Figure 100 ^{13}C NMR of 3da

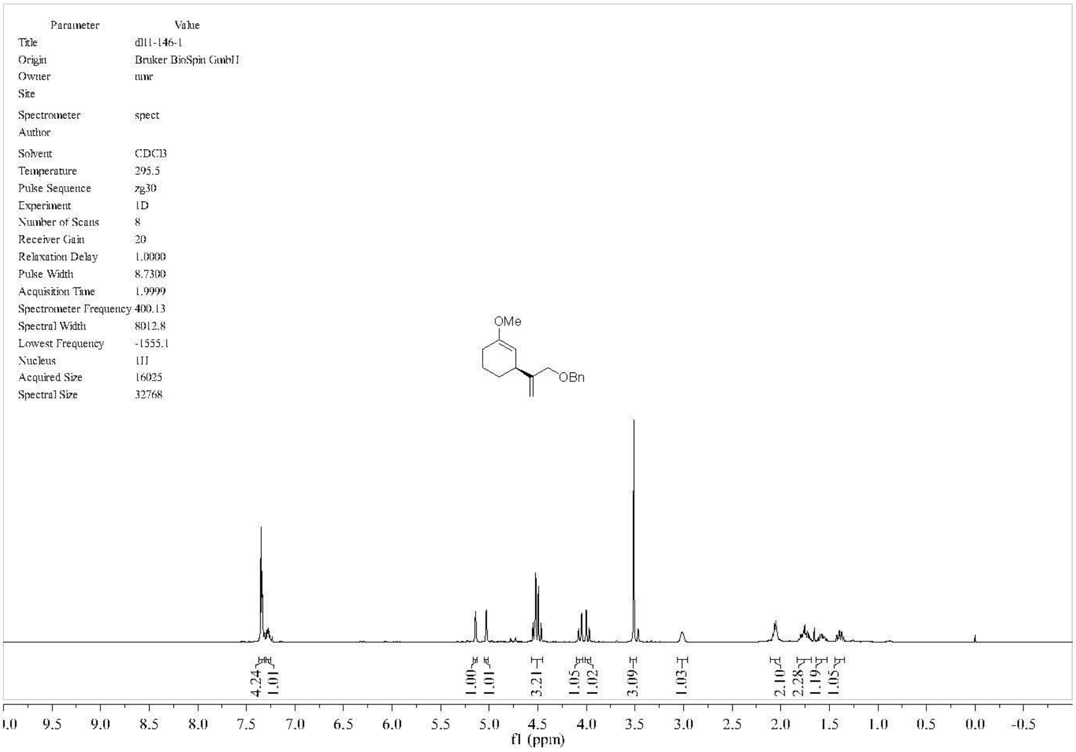

Supplementary Figure 101 ^{1}H NMR of 3ea

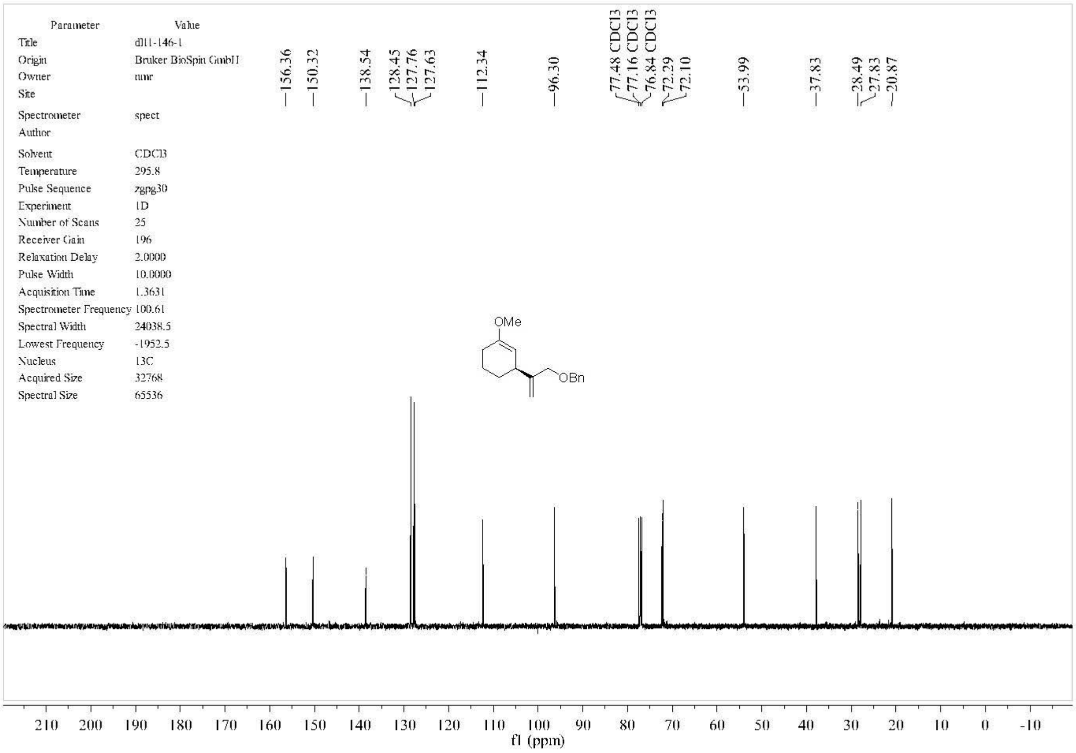

Supplementary Figure 102 ^{13}C NMR of 3ea

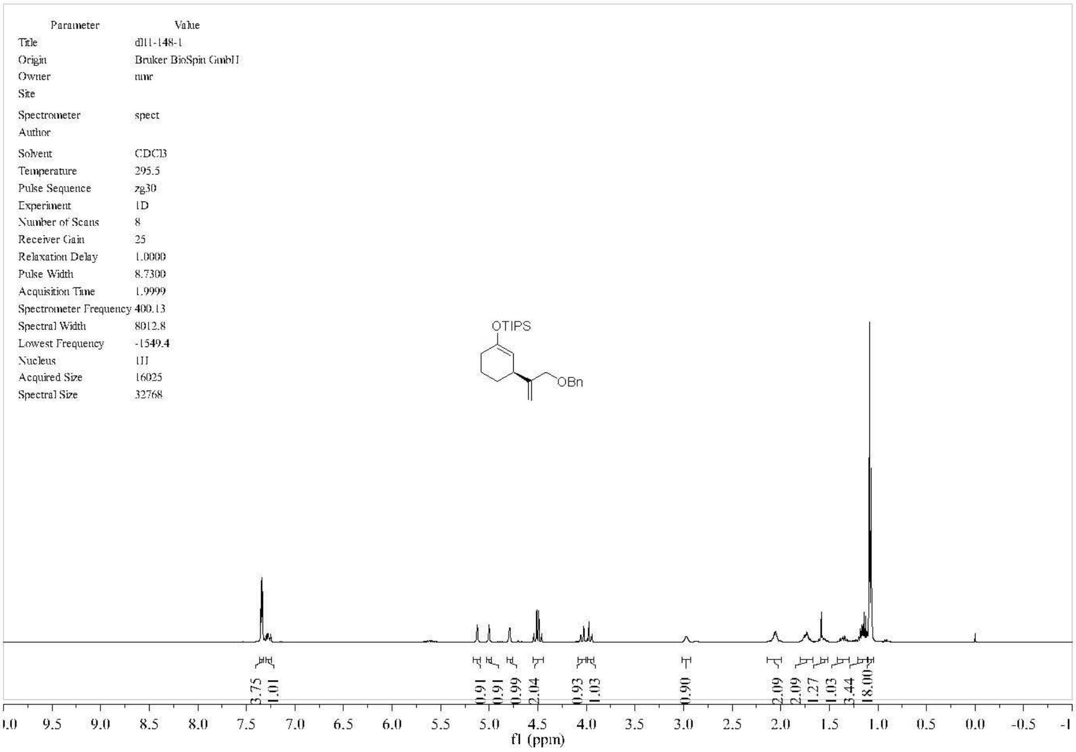

Supplementary Figure 103 ^{1}H NMR of 3fa

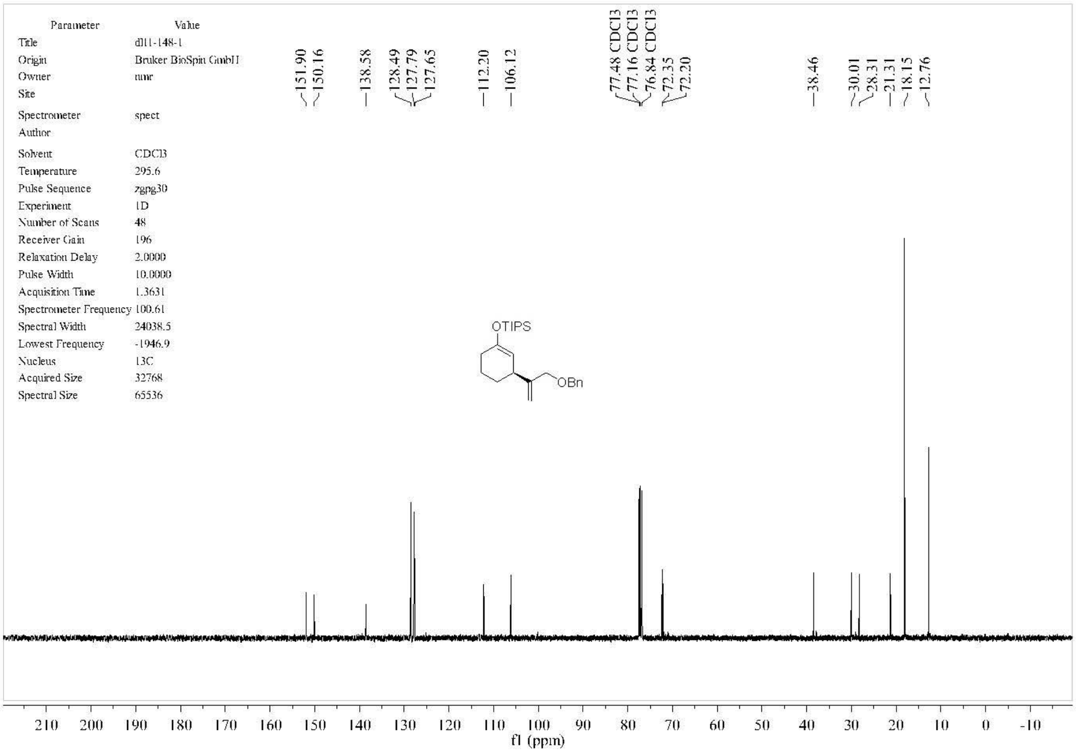

Supplementary Figure 104 ^{13}C NMR of 3fa

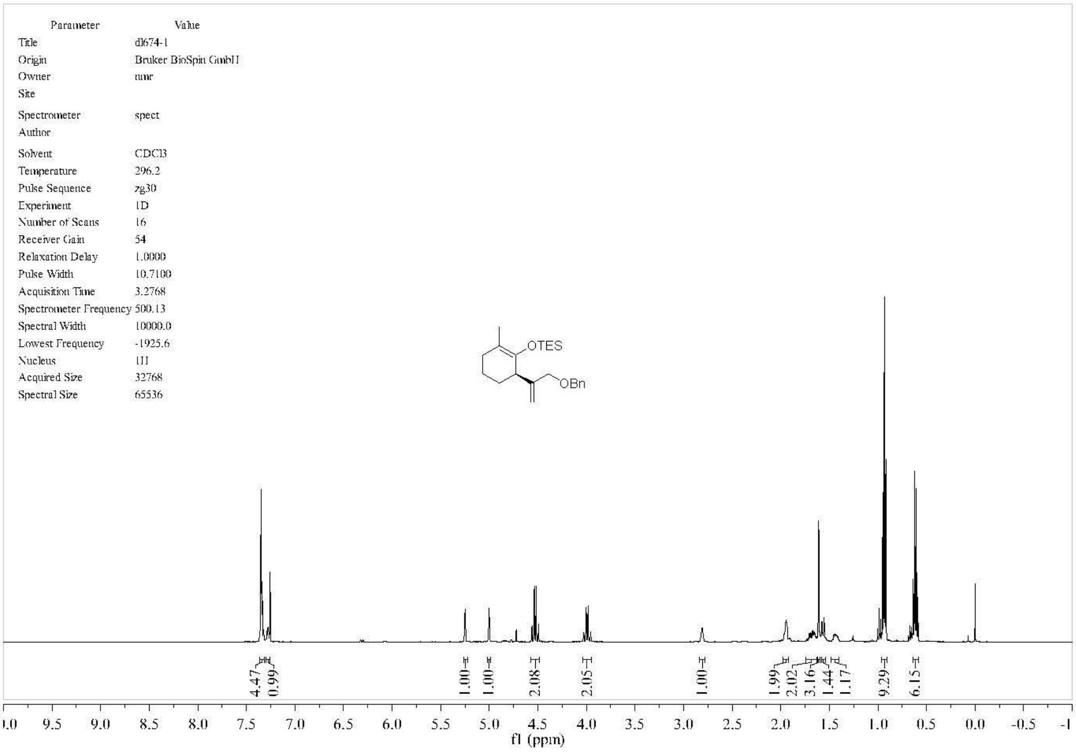

Supplementary Figure 105 ^{1}H NMR of 3ga

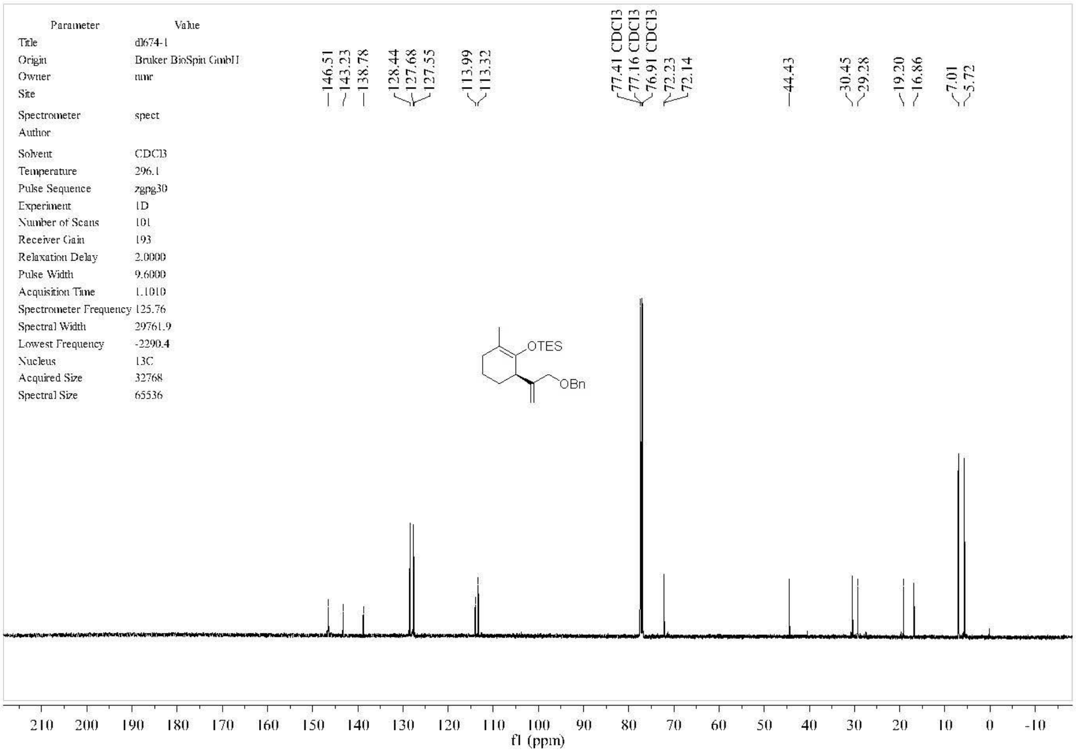

Supplementary Figure 106 ^{13}C NMR of 3ga

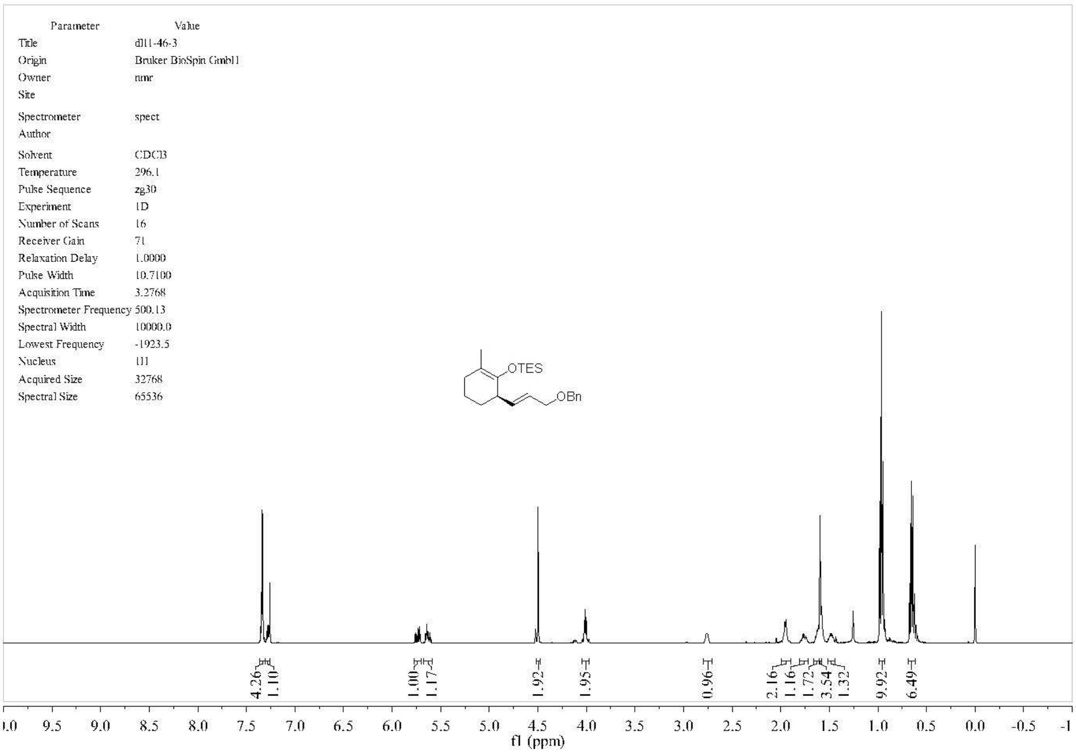

Supplementary Figure 107 ^{1}H NMR of 4ga

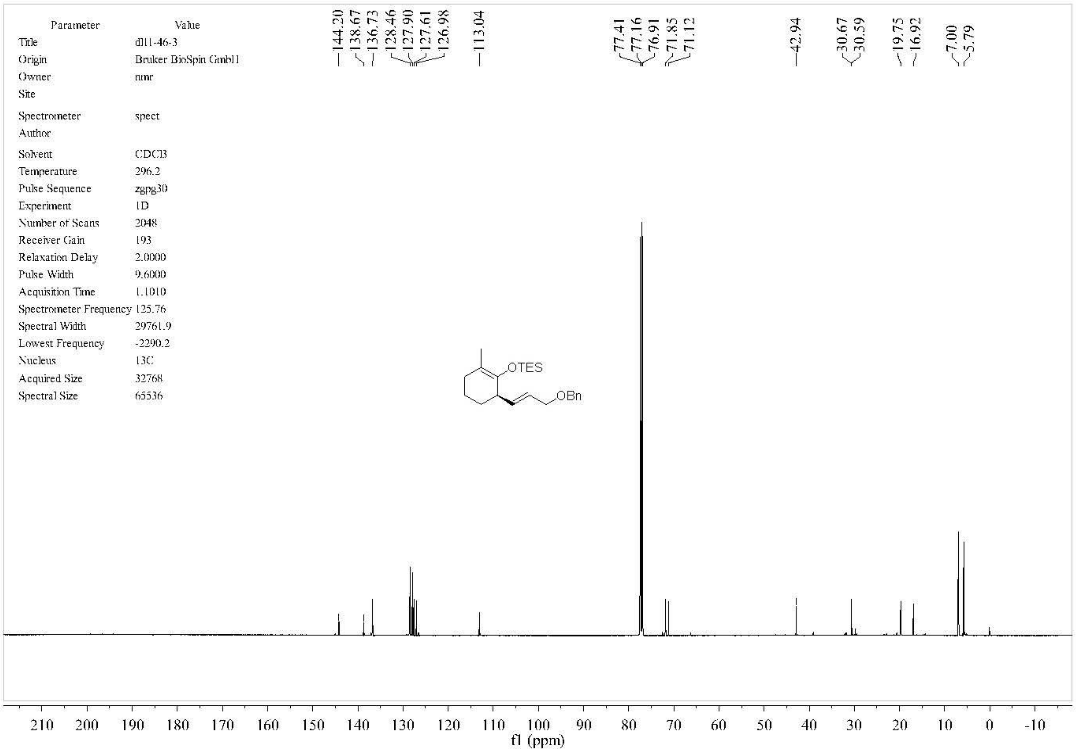

Supplementary Figure 108 ^{13}C NMR of 4ga

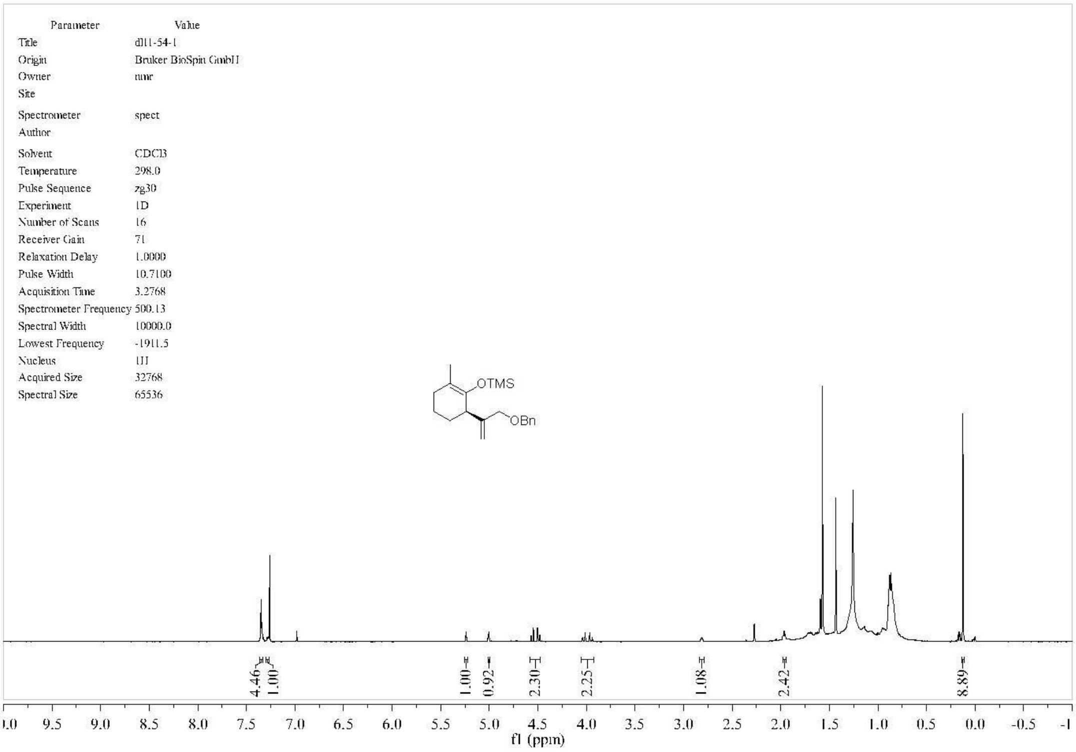

Supplementary Figure 109 ^{1}H NMR of 3ha

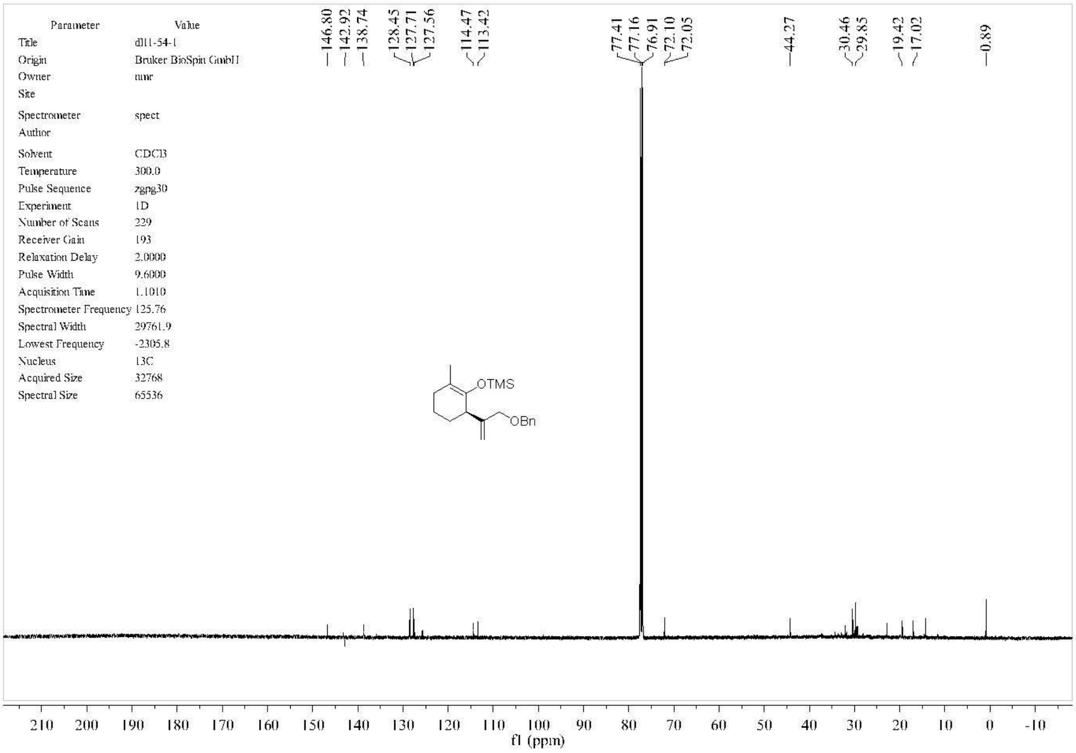

Supplementary Figure 110 ^{13}C NMR of 3ha

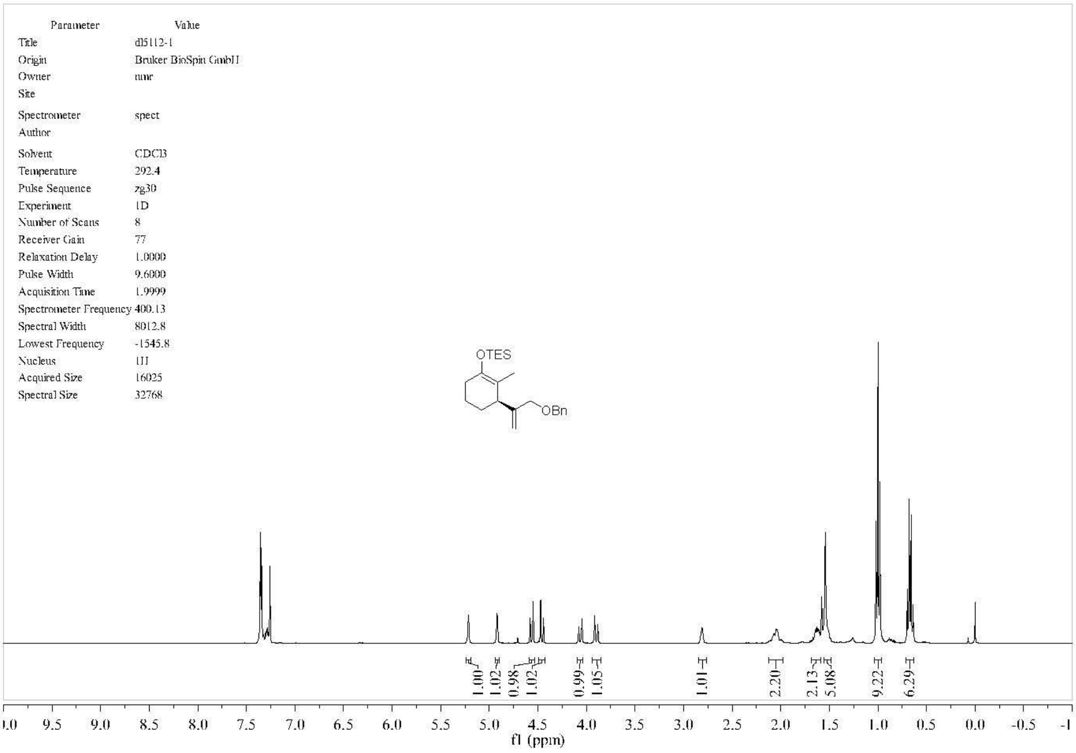

Supplementary Figure 111 ^{1}H NMR of 3ia

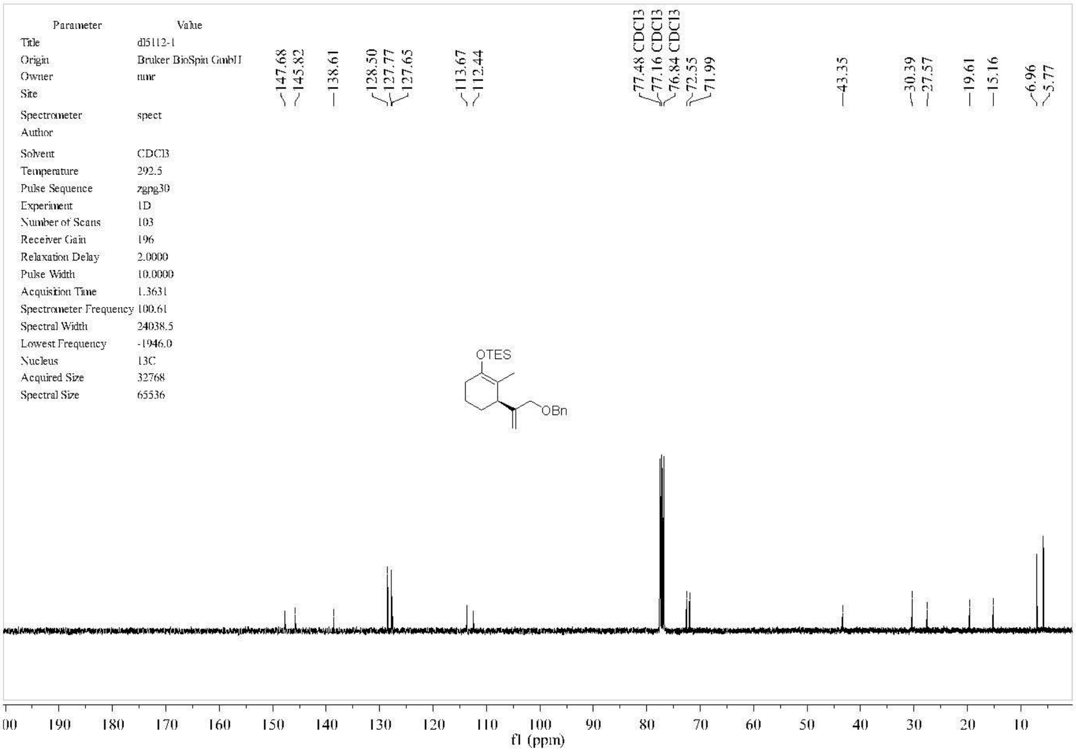

Supplementary Figure 112 ^{13}C NMR of 3ia

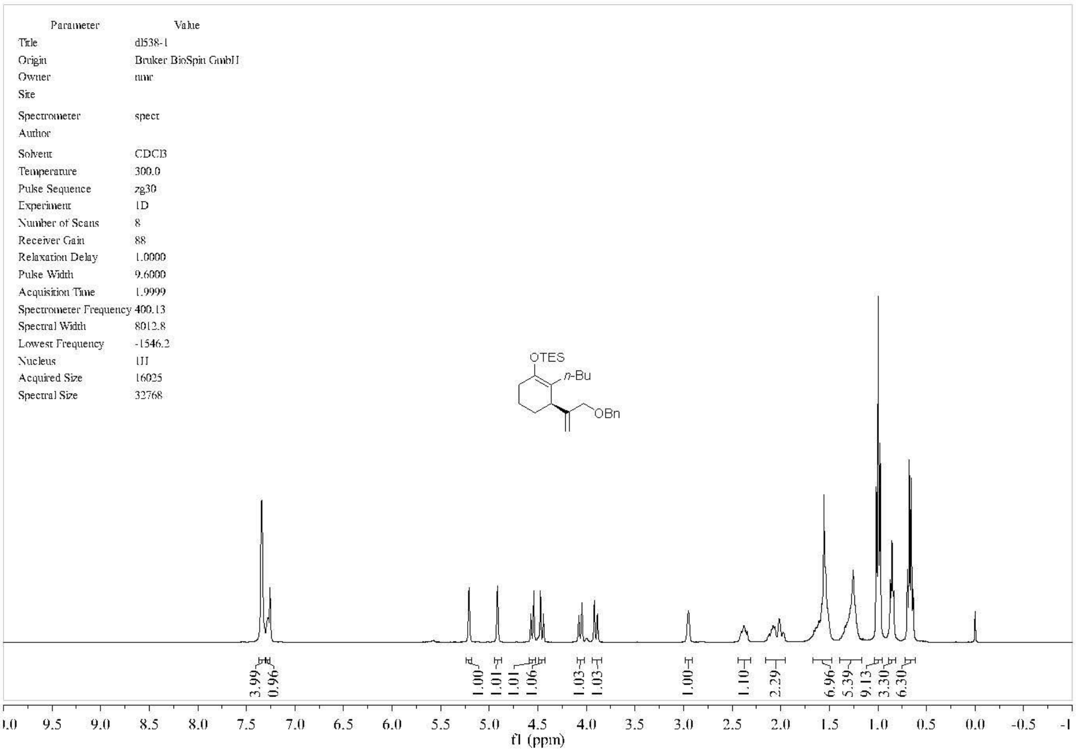

Supplementary Figure 113 ^{1}H NMR of 3ja

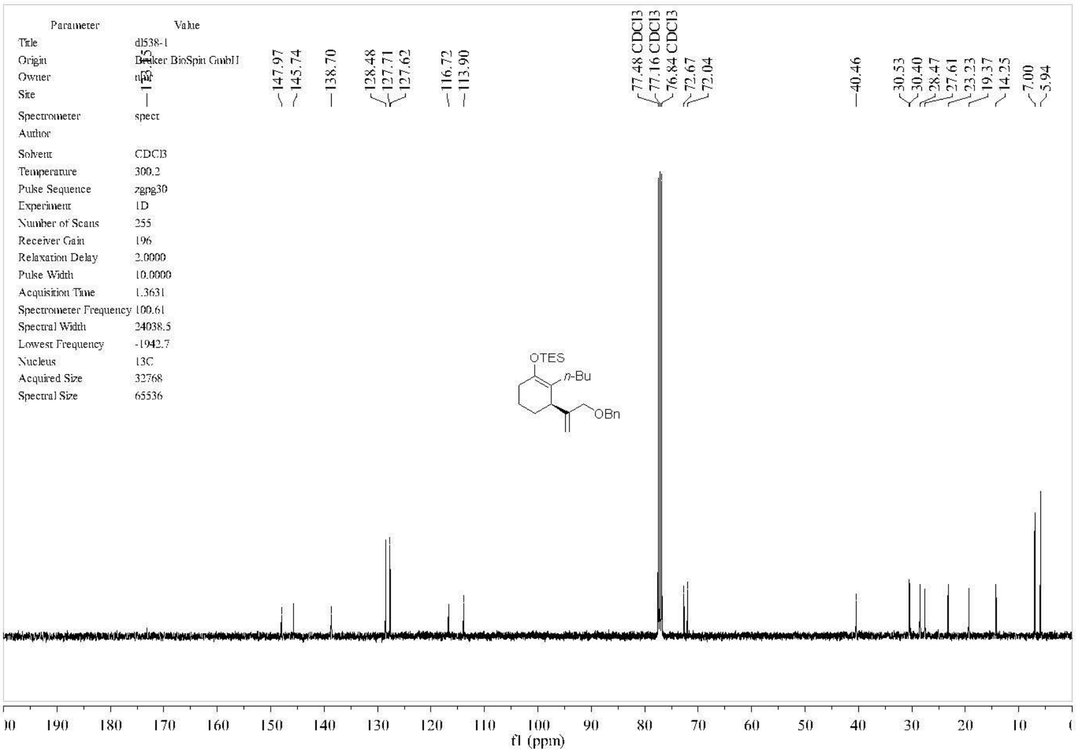

Supplementary Figure 114 ^{13}C NMR of 3ja

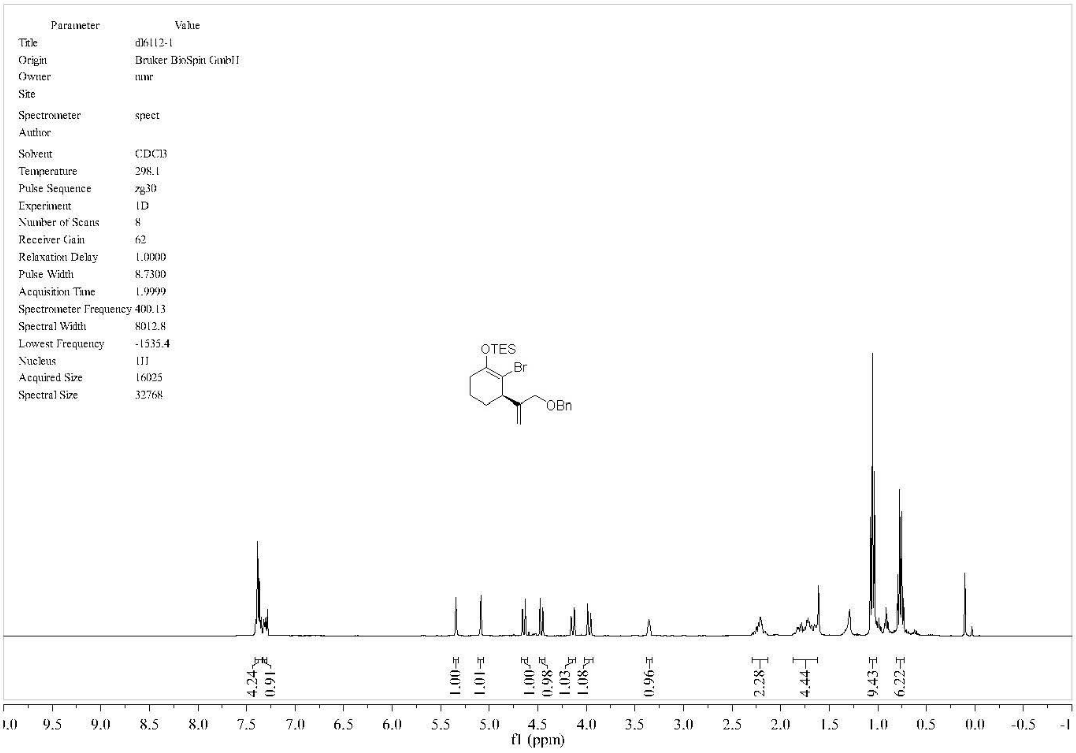

Supplementary Figure 115 ^{1}H NMR of 3ka

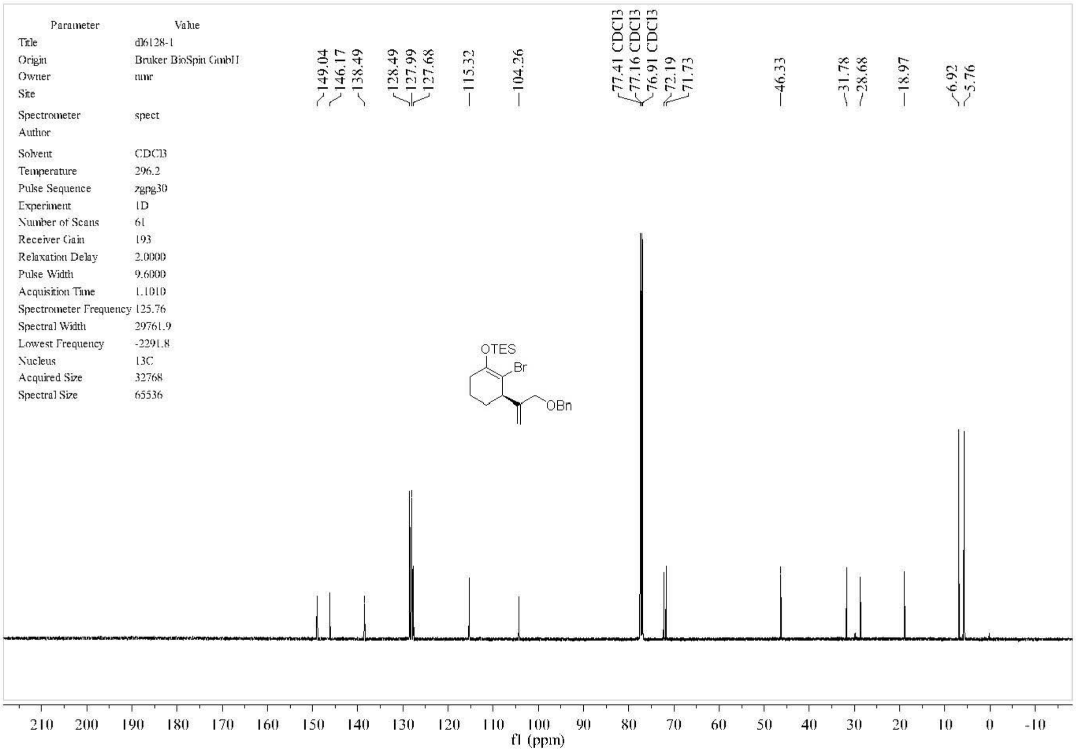

Supplementary Figure 116 ^{13}C NMR of 3ka

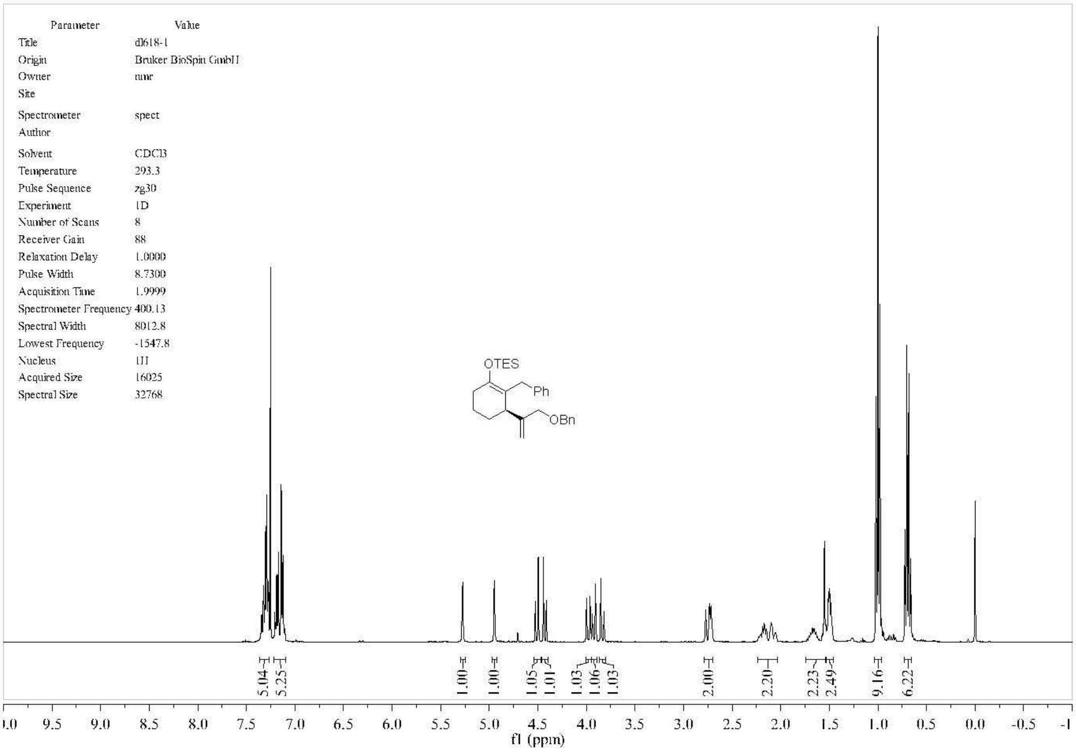

Supplementary Figure 117 ^{1}H NMR of 3la

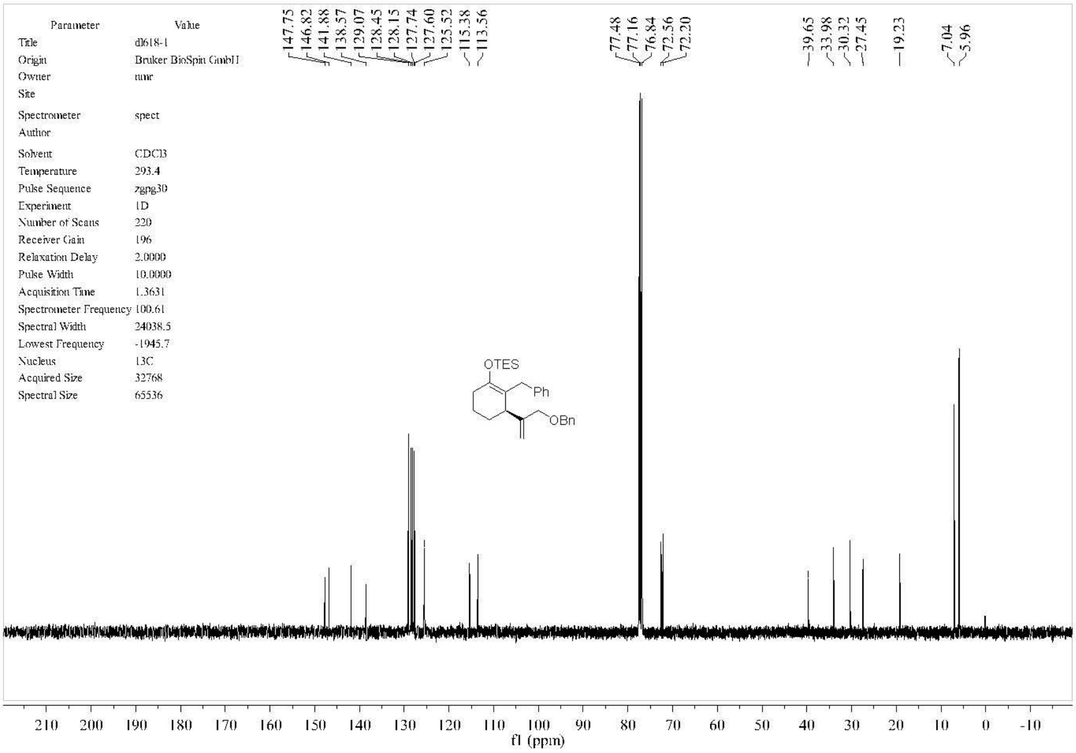

Supplementary Figure 118 ^{13}C NMR of 3la

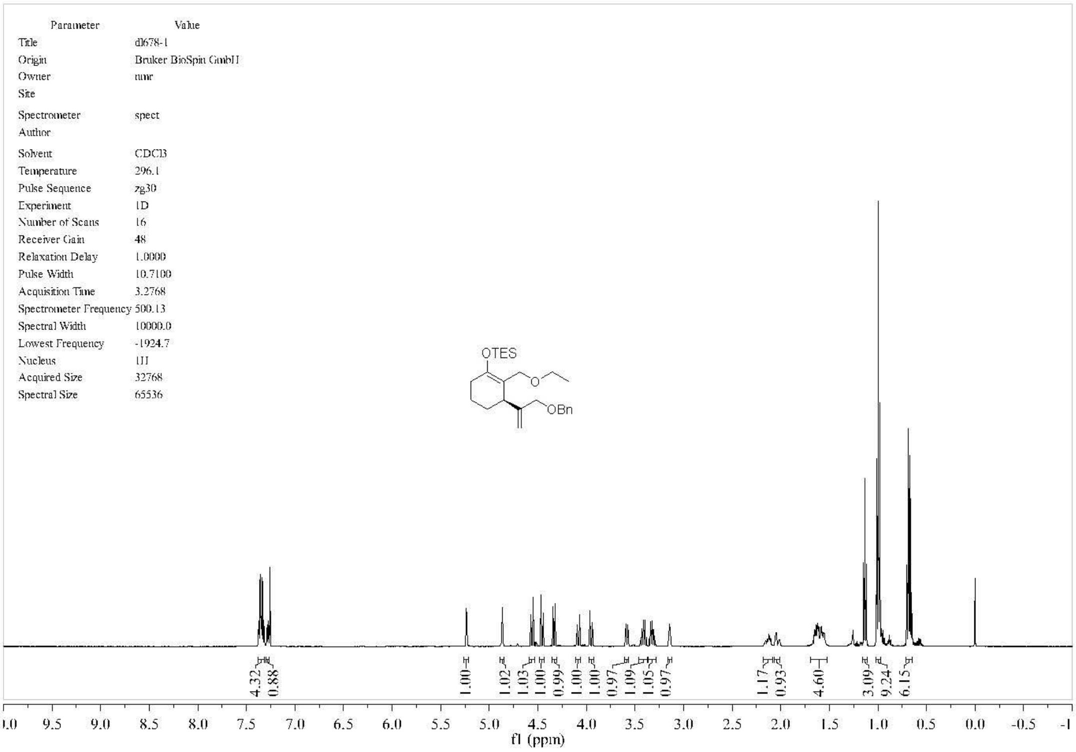

Supplementary Figure 119 ^{1}H NMR of 3ma

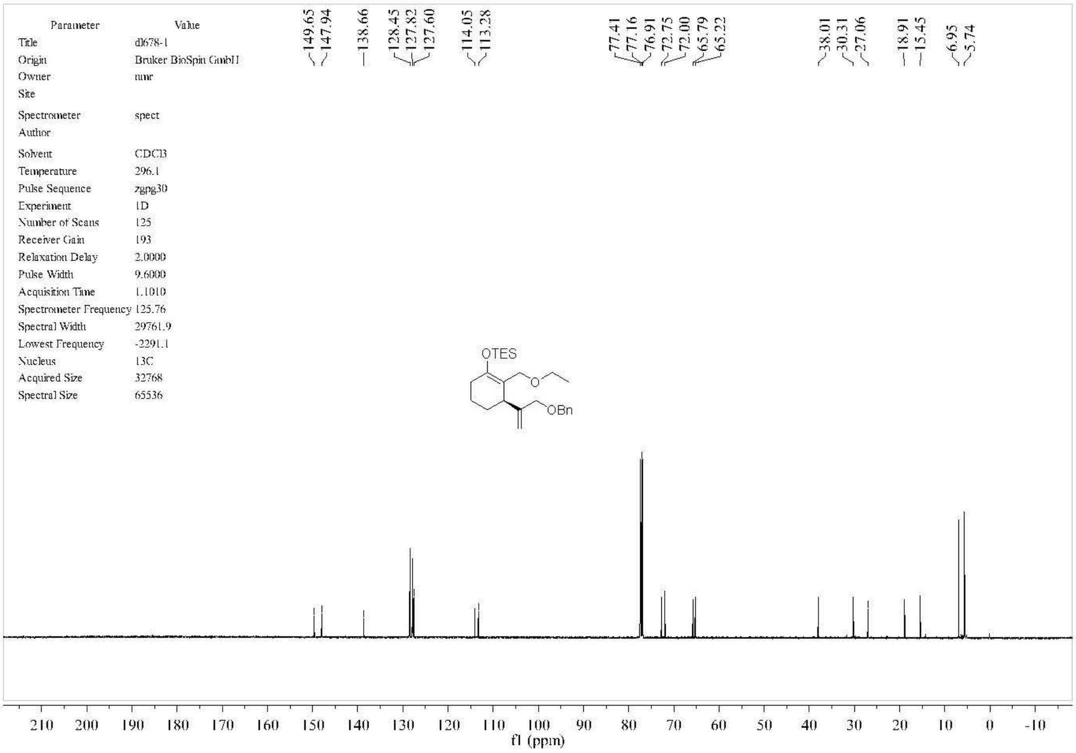

Supplementary Figure 120 ^{13}C NMR of 3ma

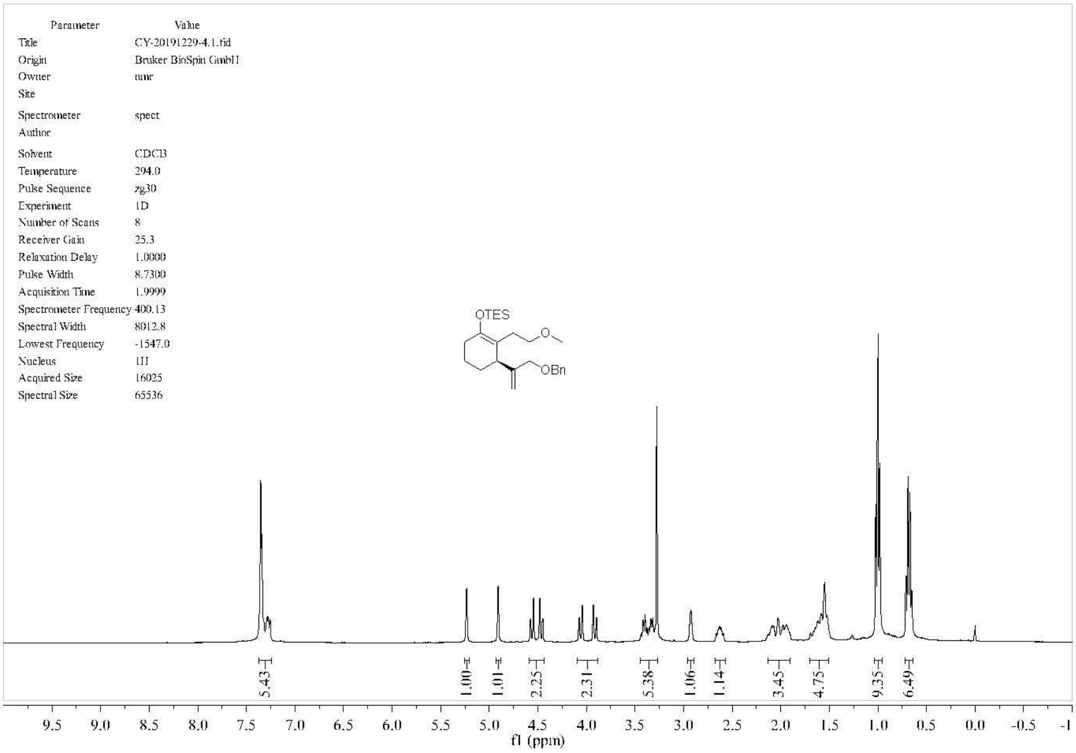

Supplementary Figure 121 ^{1}H NMR of 3na

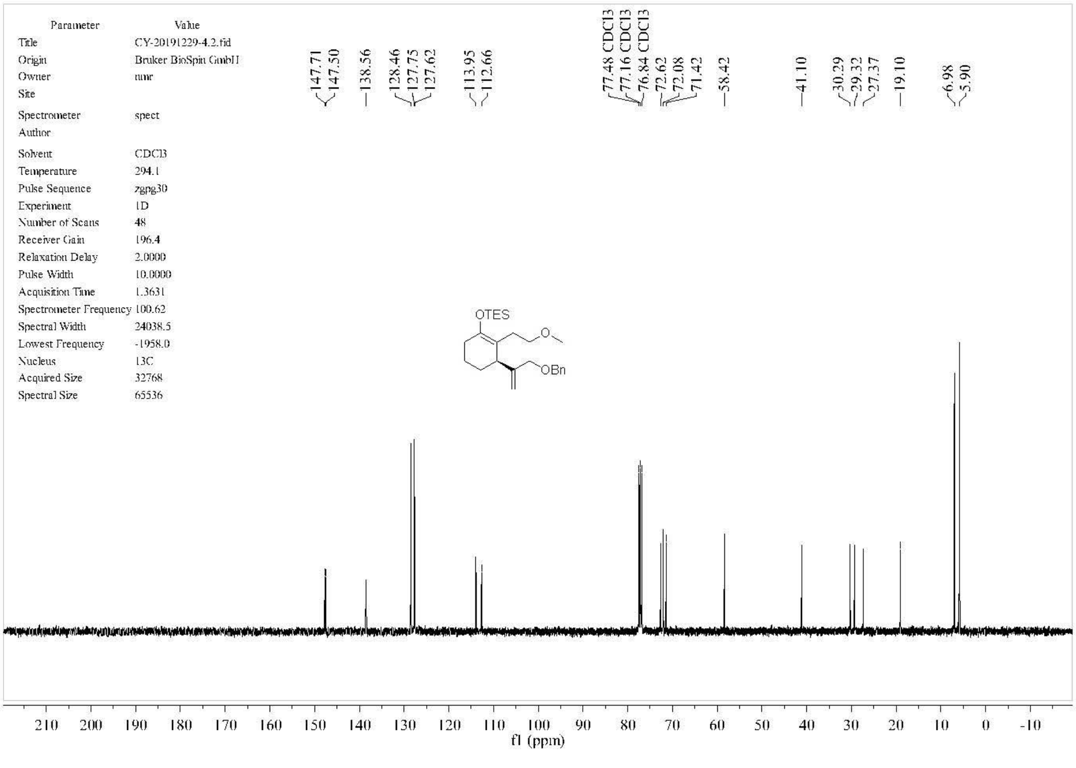

Supplementary Figure 122 ^{13}C NMR of 3na

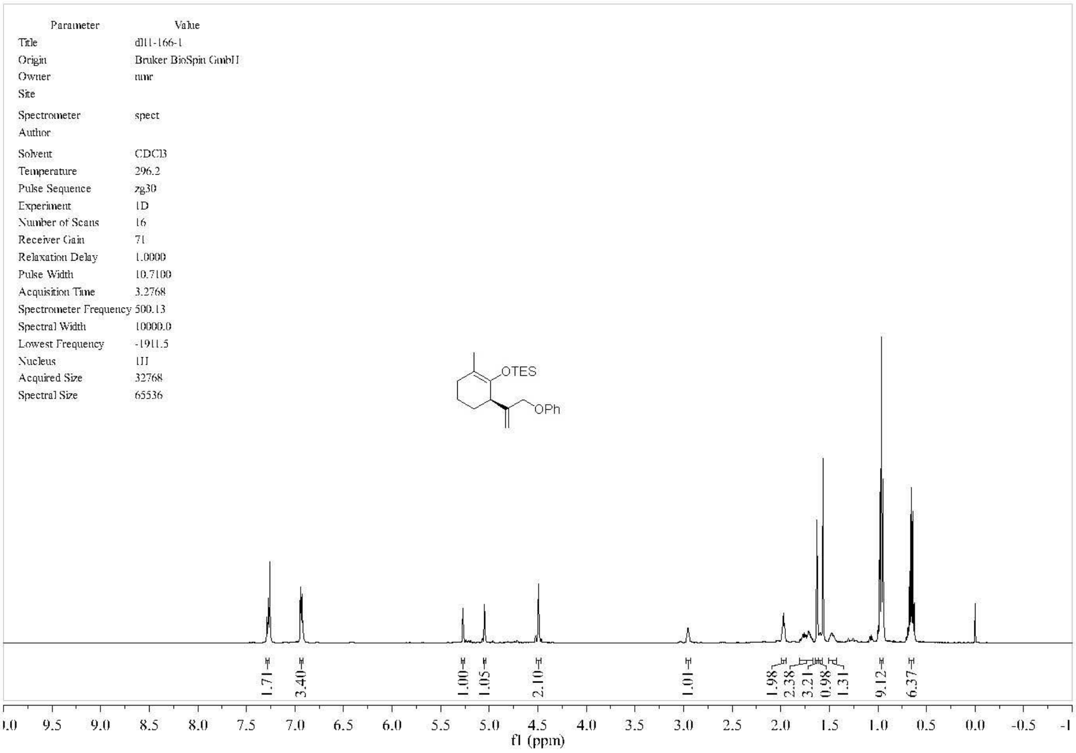

Supplementary Figure 123 ^{1}H NMR of 3gb

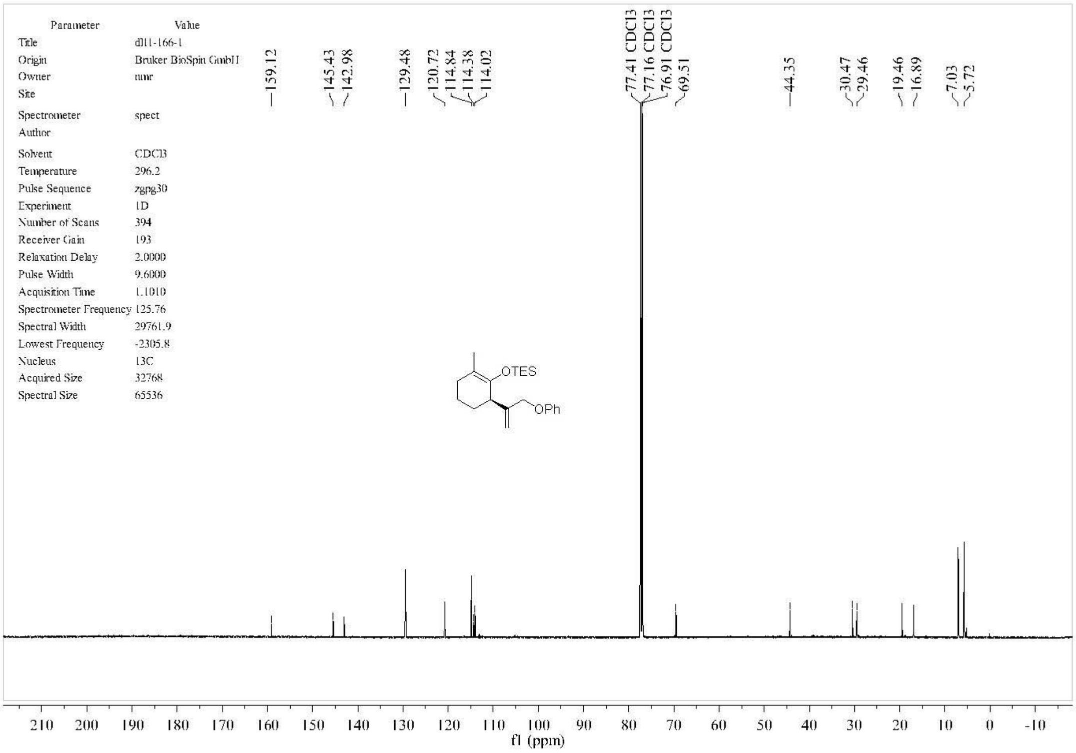

Supplementary Figure 124 ^{13}C NMR of 3gb

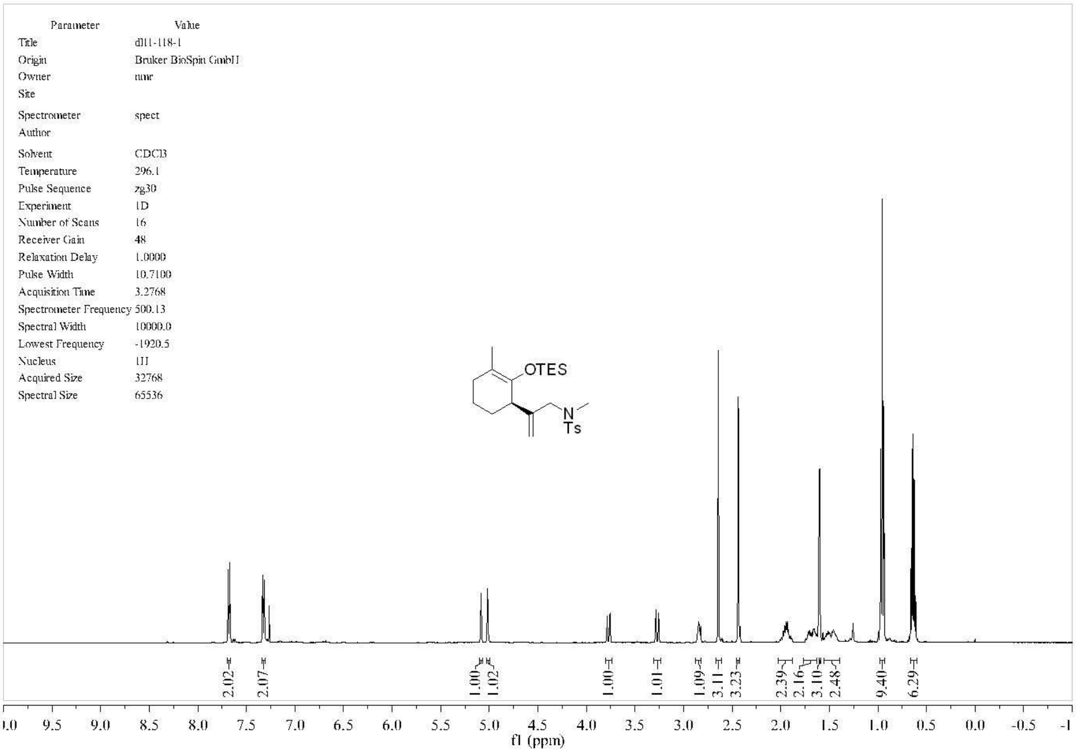

Supplementary Figure 125 ^{1}H NMR of 3gc

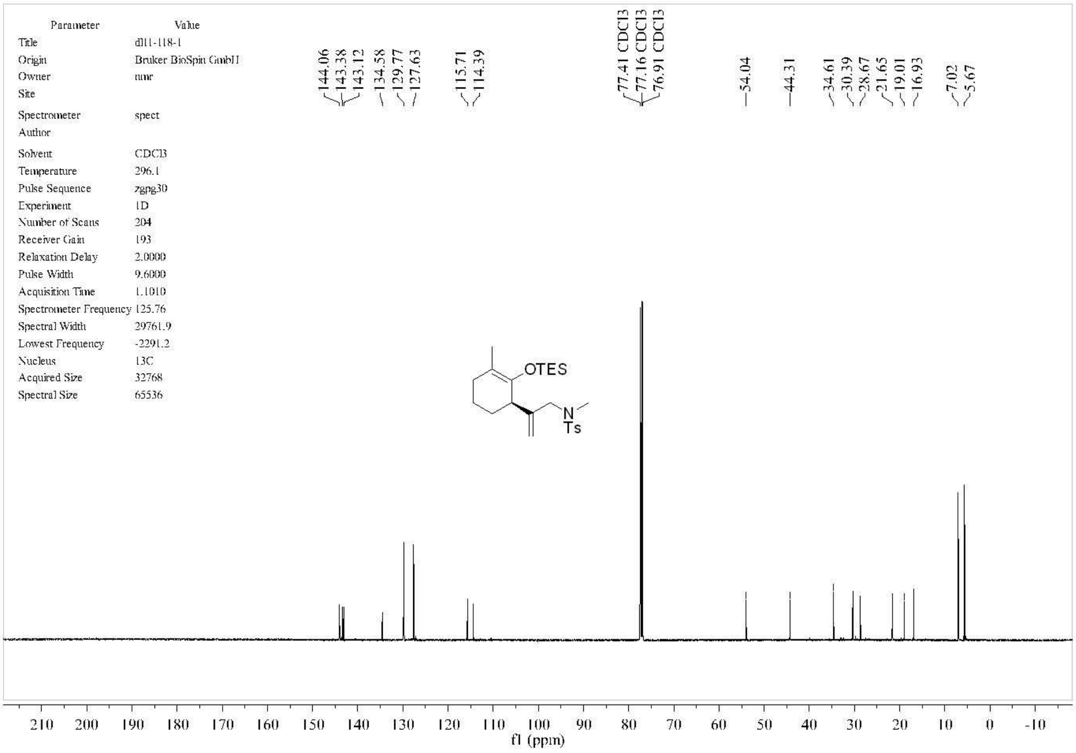

Supplementary Figure 126 ^{13}C NMR of 3gc

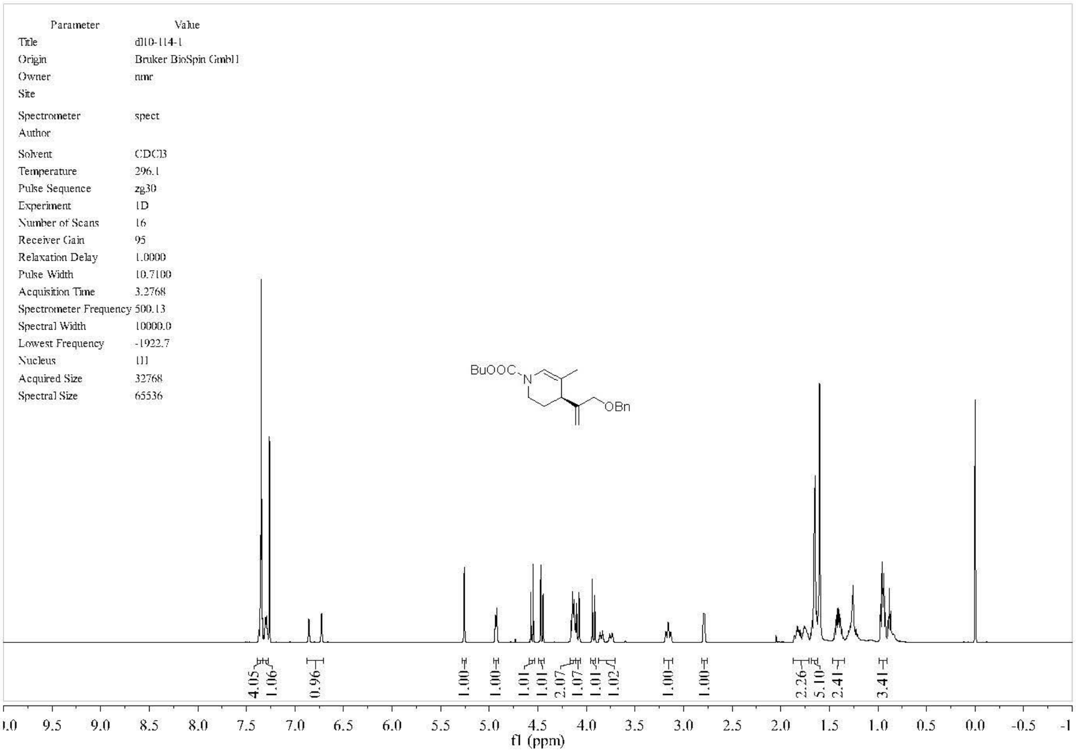

Supplementary Figure 127 ^{1}H NMR of 3oa

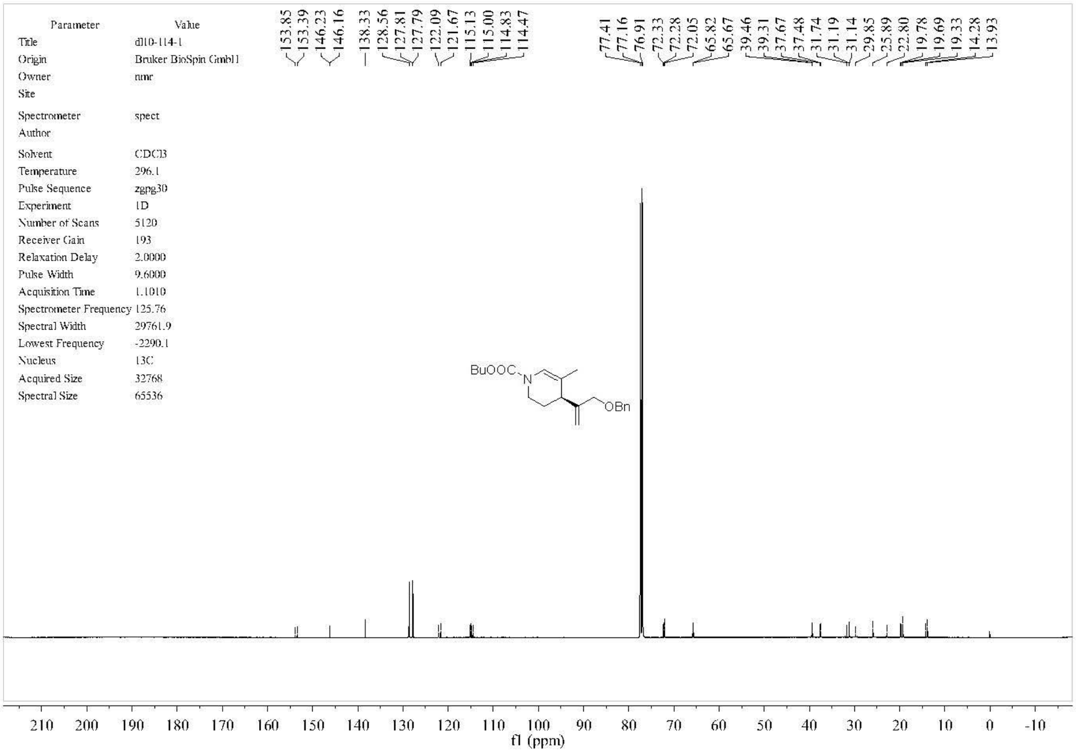

Supplementary Figure 128 ^{13}C NMR of 3oa

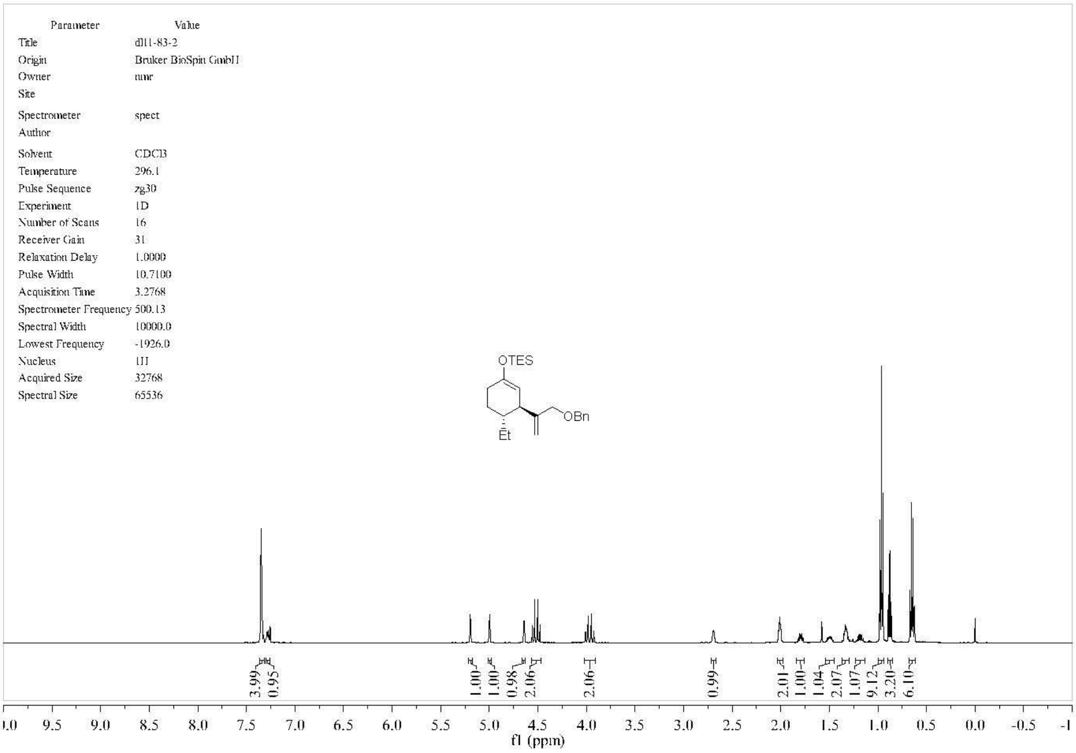

Supplementary Figure 129 ^{1}H NMR of 3pa

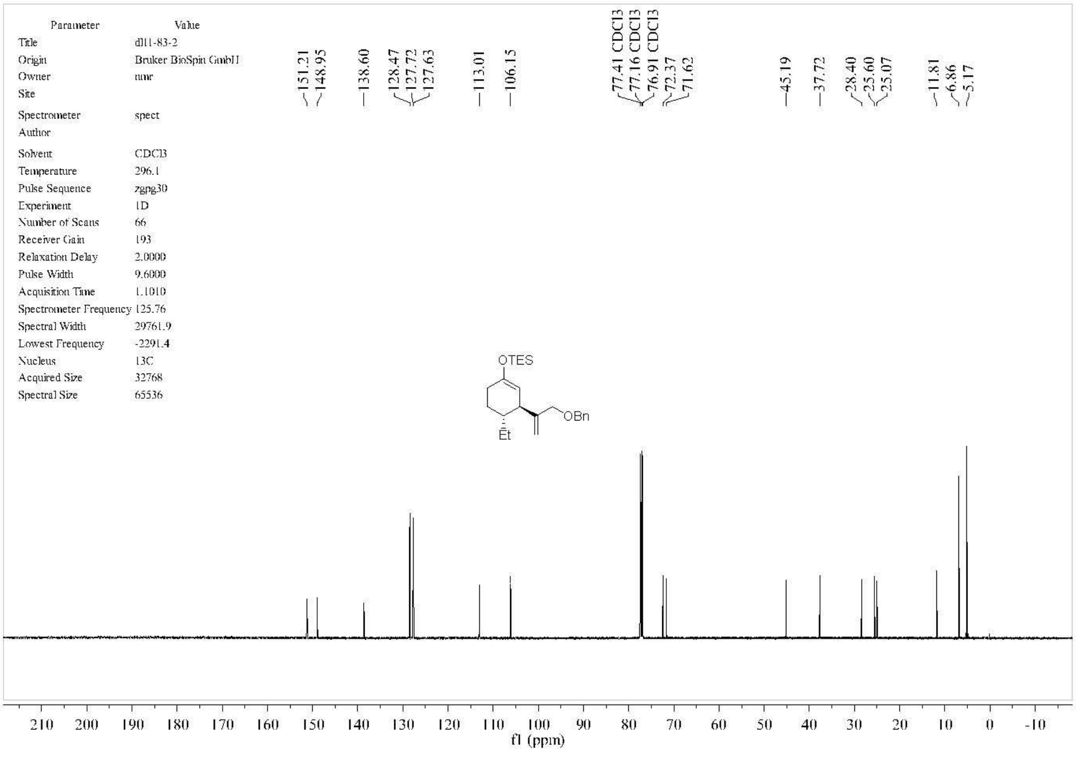

Supplementary Figure 130 ^{13}C NMR of 3pa

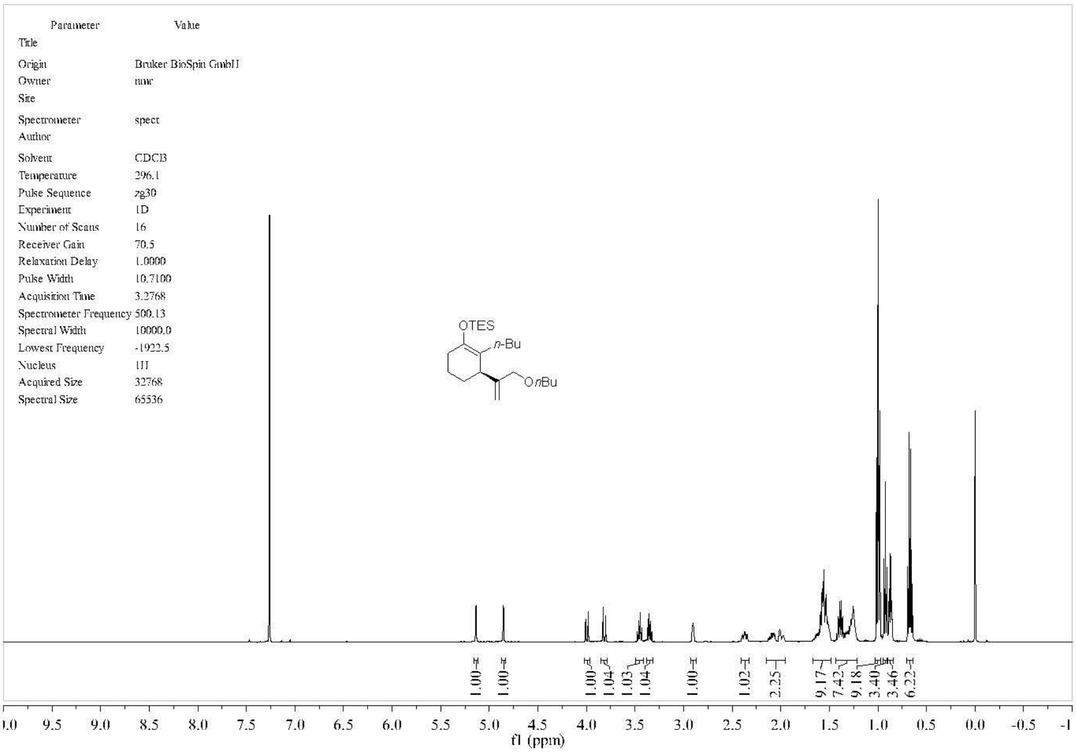

Supplementary Figure 131 ^{1}H NMR of 3jd

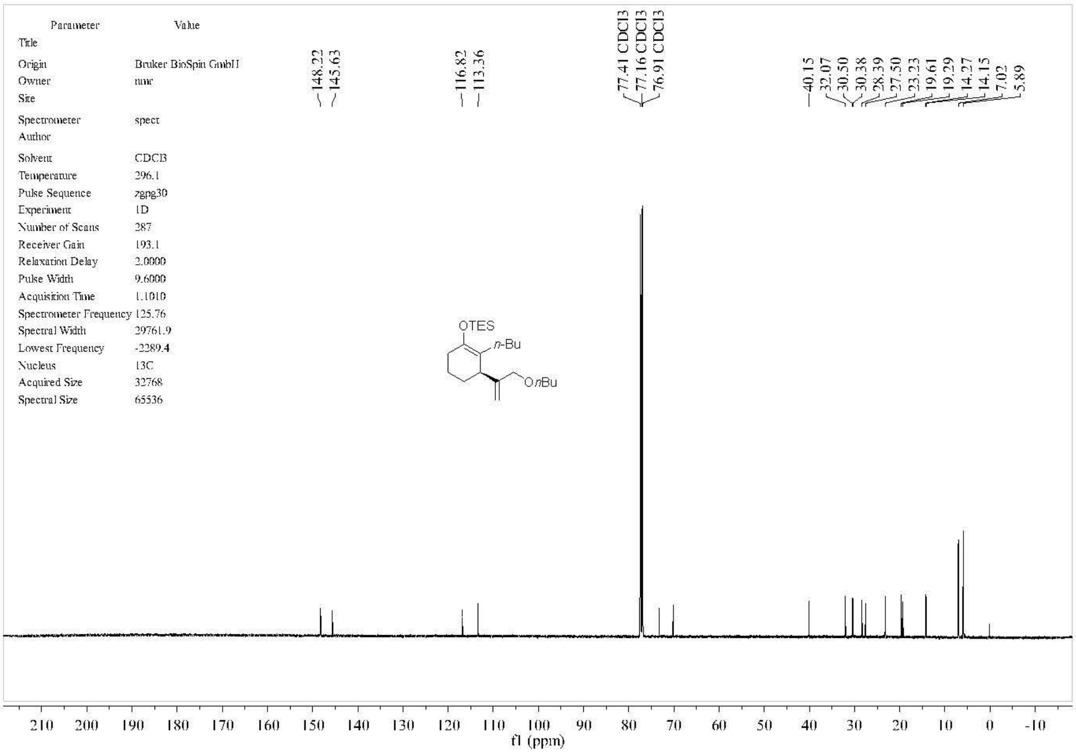

Supplementary Figure 132 ^{13}C NMR of 3jd

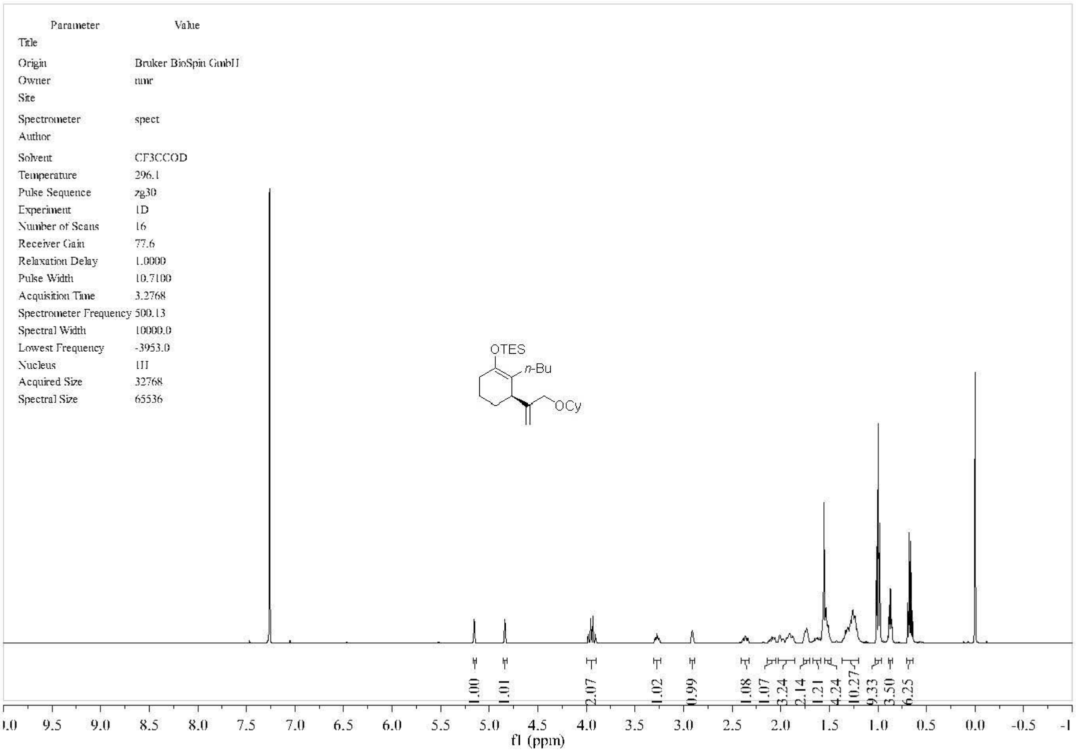

Supplementary Figure 133 ^{1}H NMR of 3je

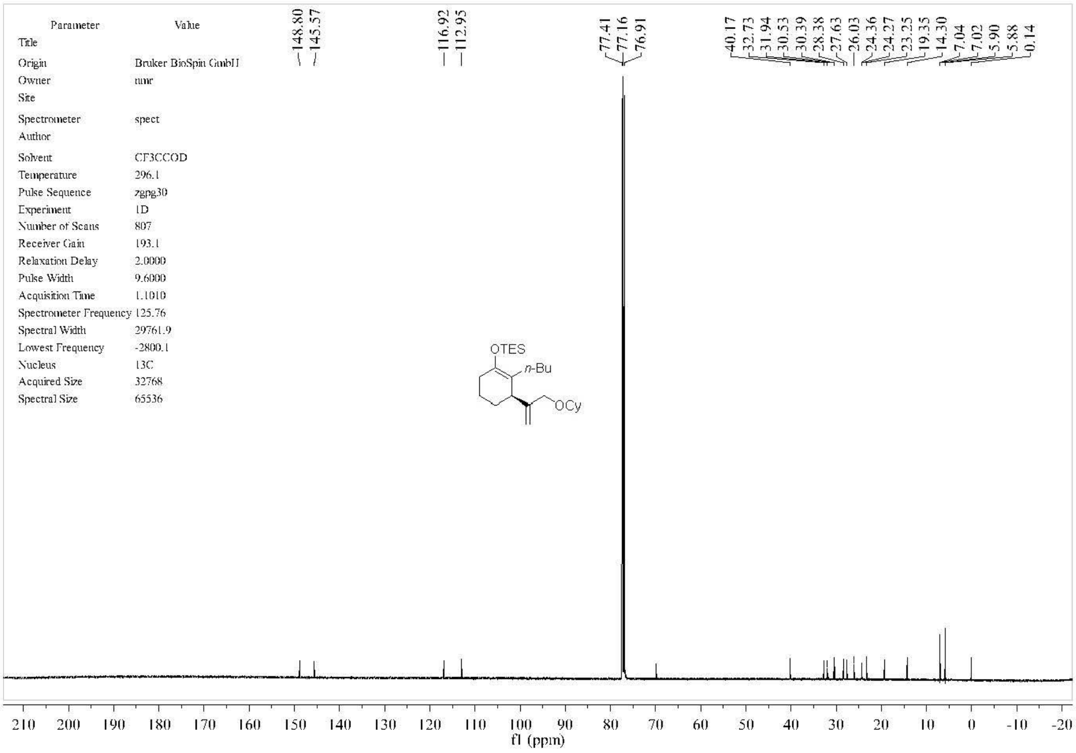

Supplementary Figure 134 ^{13}C NMR of 3je

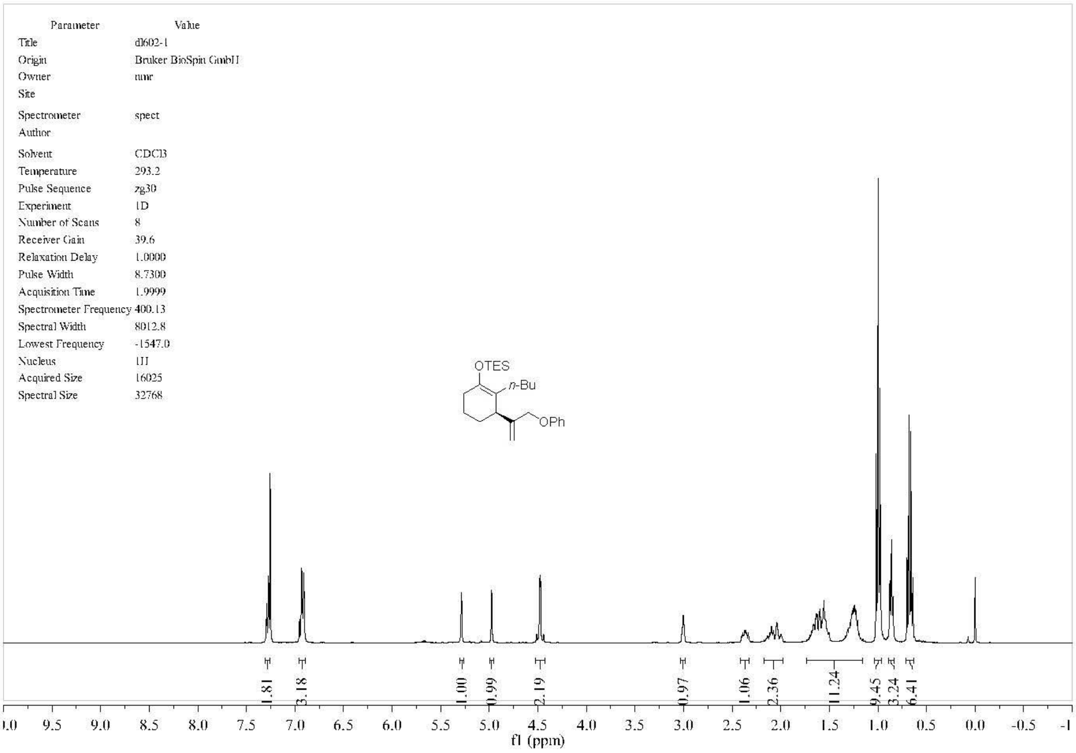

Supplementary Figure 135 ^{1}H NMR of 3jb

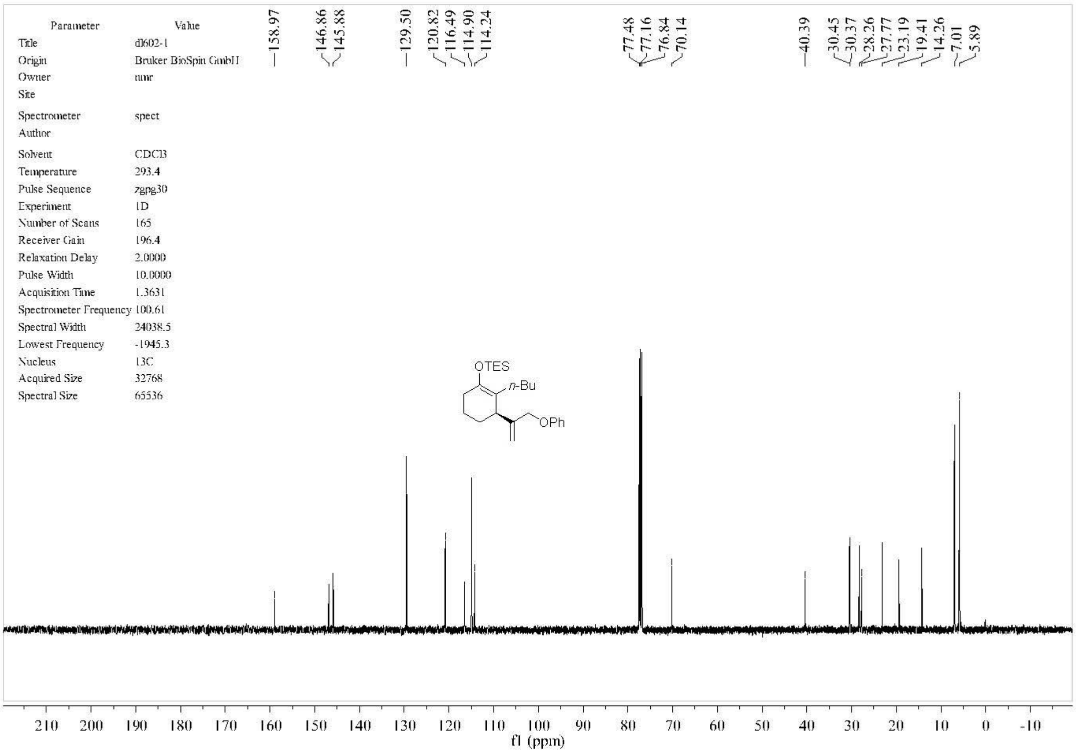

Supplementary Figure 136 ^{13}C NMR of 3jb

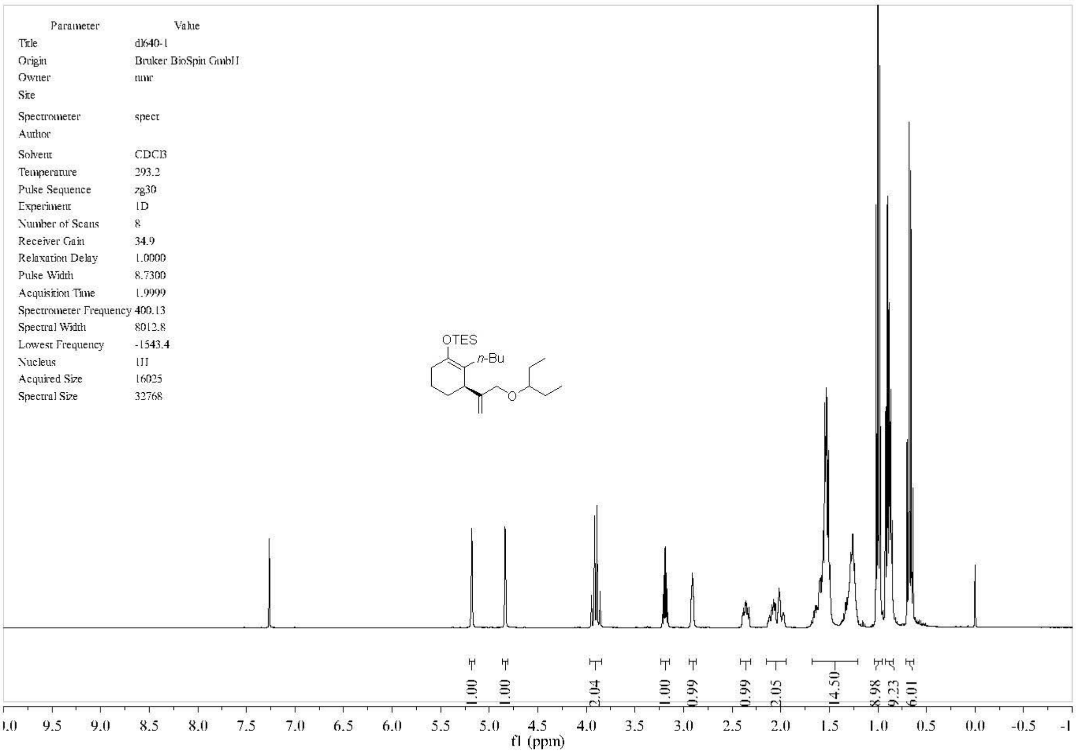

Supplementary Figure 137 ^{1}H NMR of 3jf

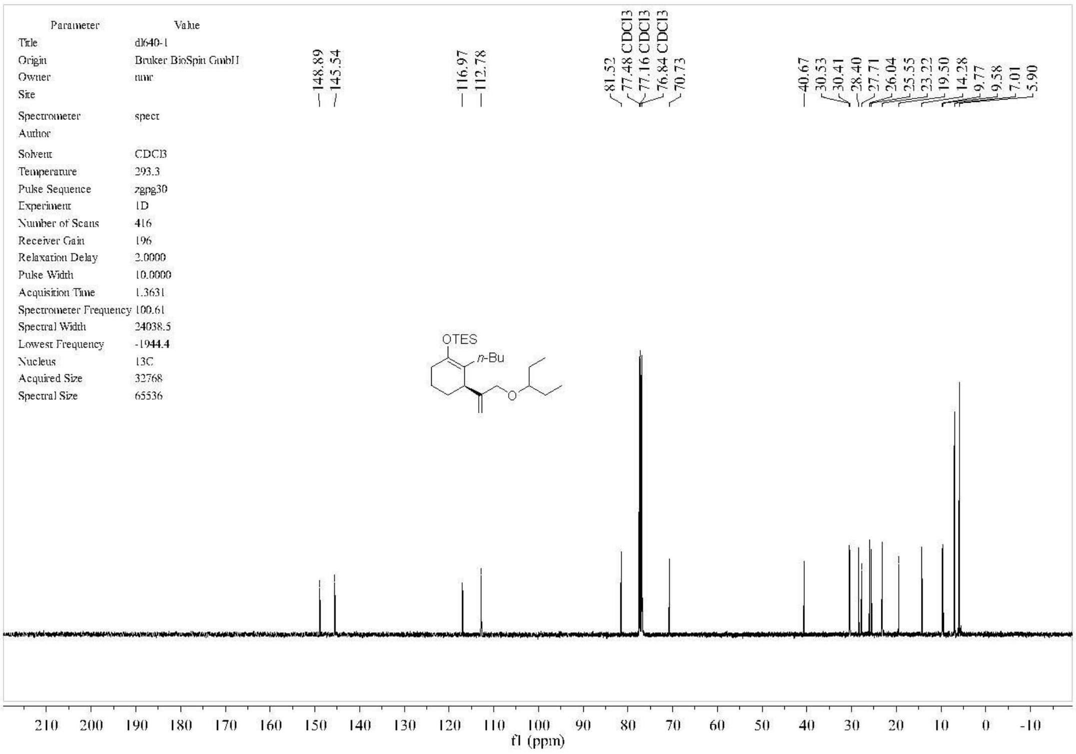

Supplementary Figure 138 ^{13}C NMR of 3jf

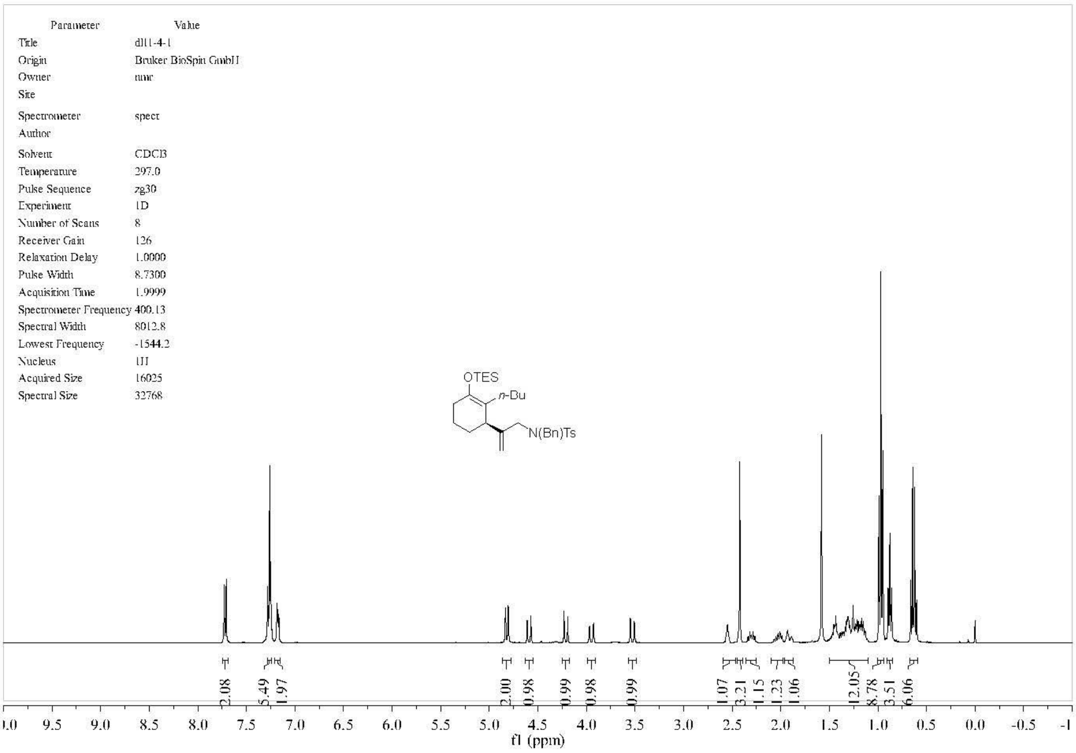

Supplementary Figure 139 ^{1}H NMR of 3jh

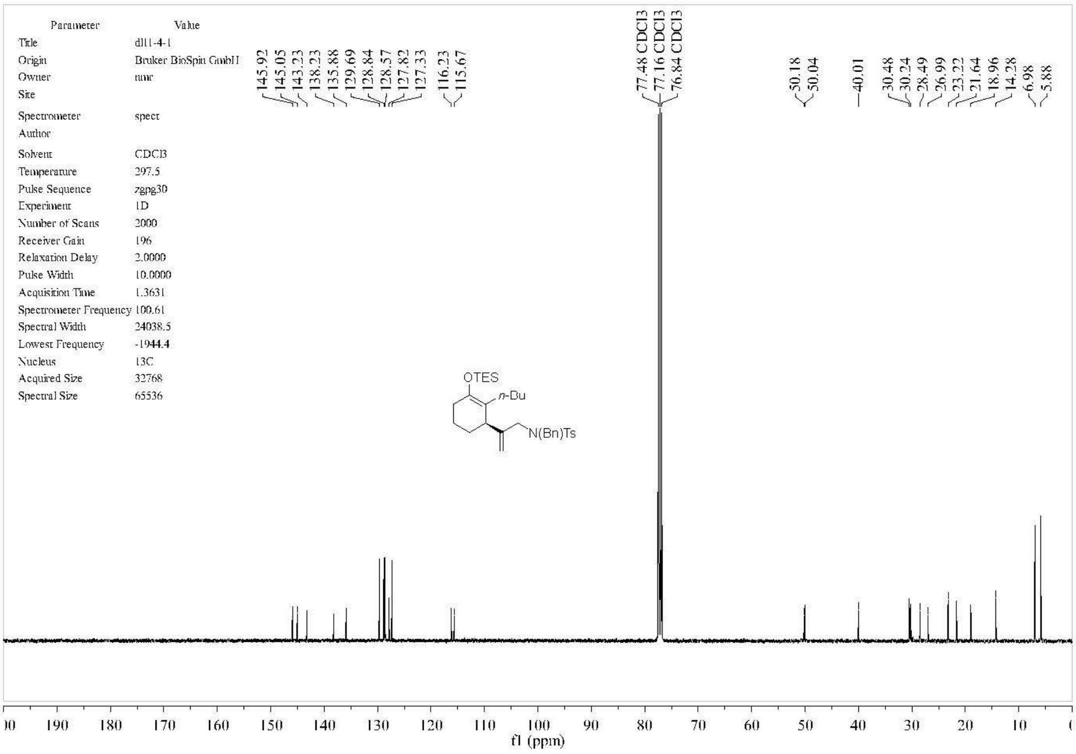

Supplementary Figure 140 ^{13}C NMR of 3jh

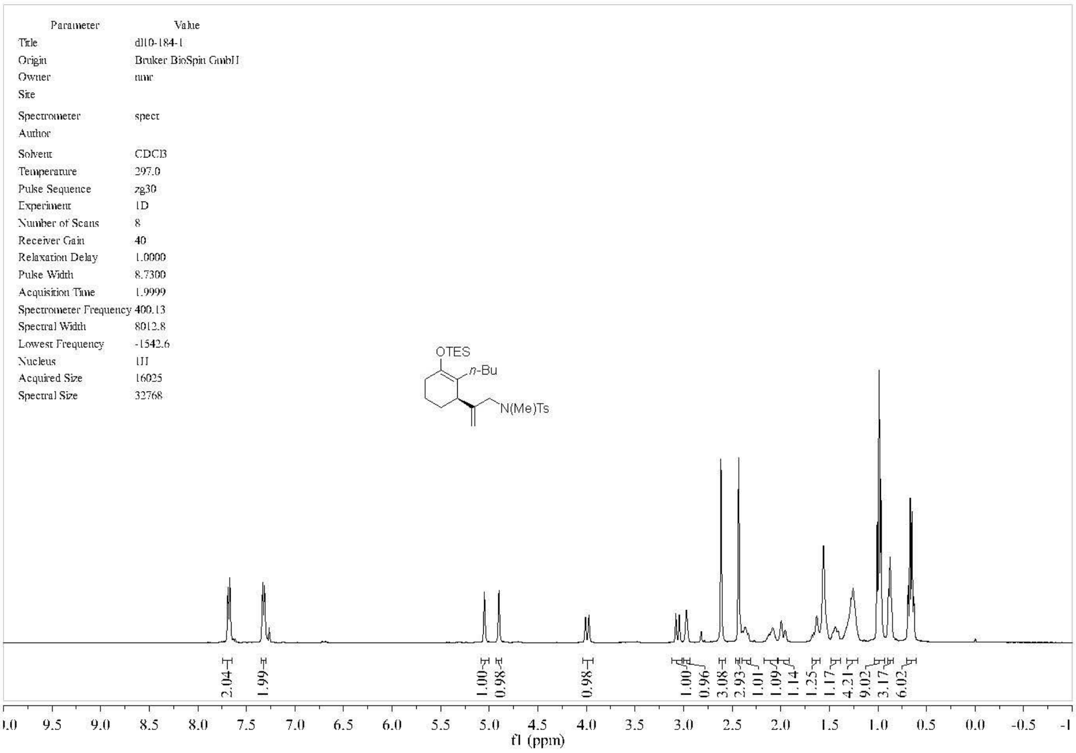

Supplementary Figure 141 ^{1}H NMR of 3jc

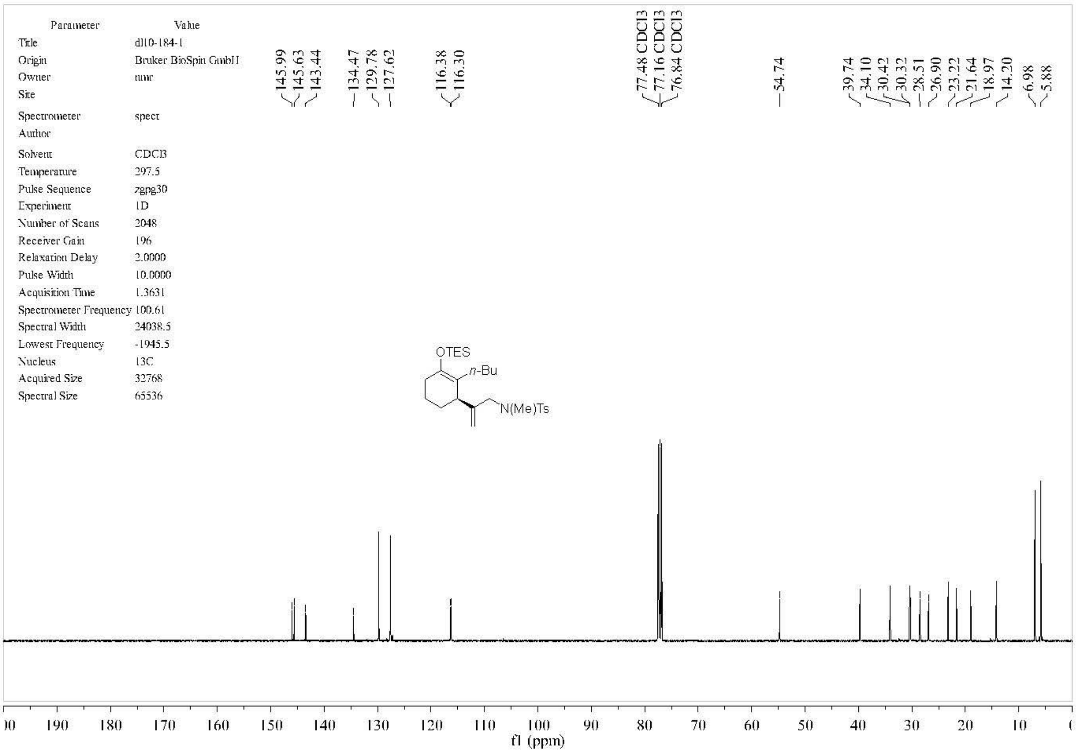

Supplementary Figure 142 ^{13}C NMR of 3jc

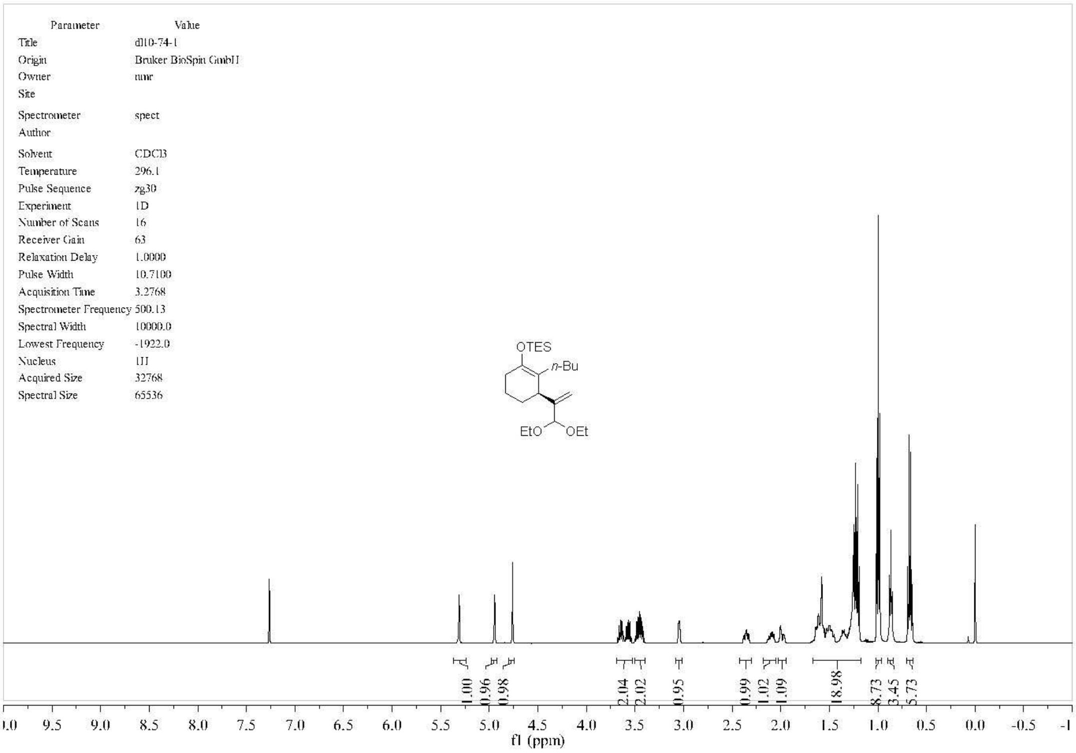

Supplementary Figure 143 ^{1}H NMR of 3ji

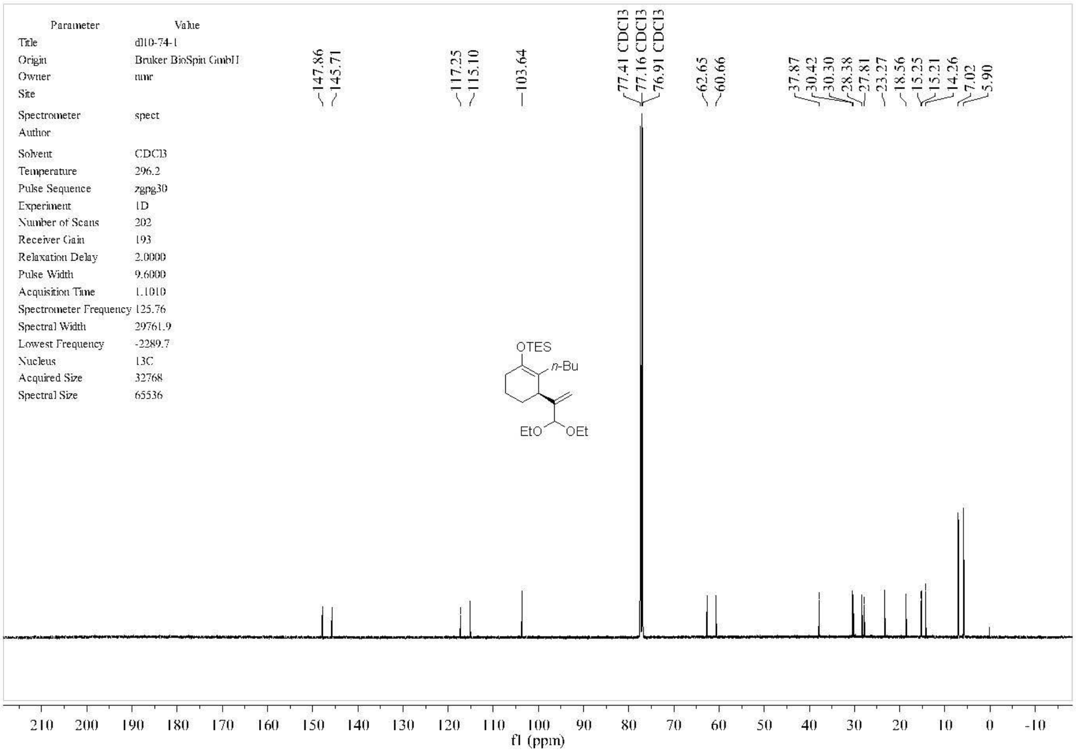

Supplementary Figure 144 ^{13}C NMR of 3jc

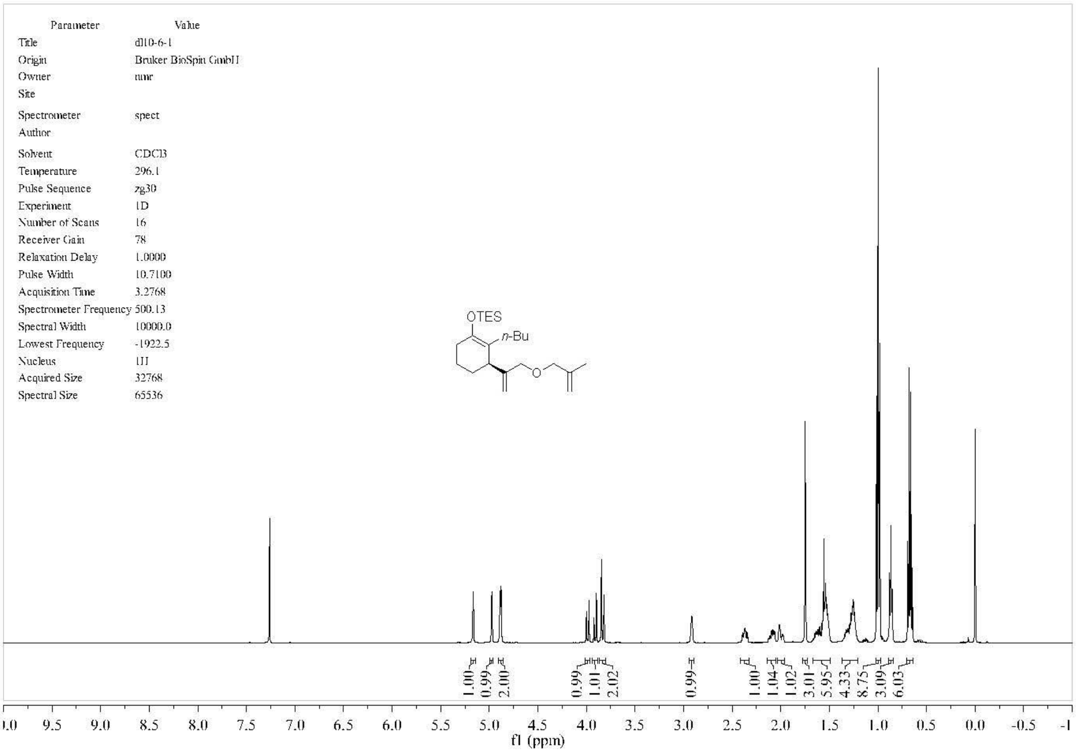

Supplementary Figure 145 ^{1}H NMR of 3ji

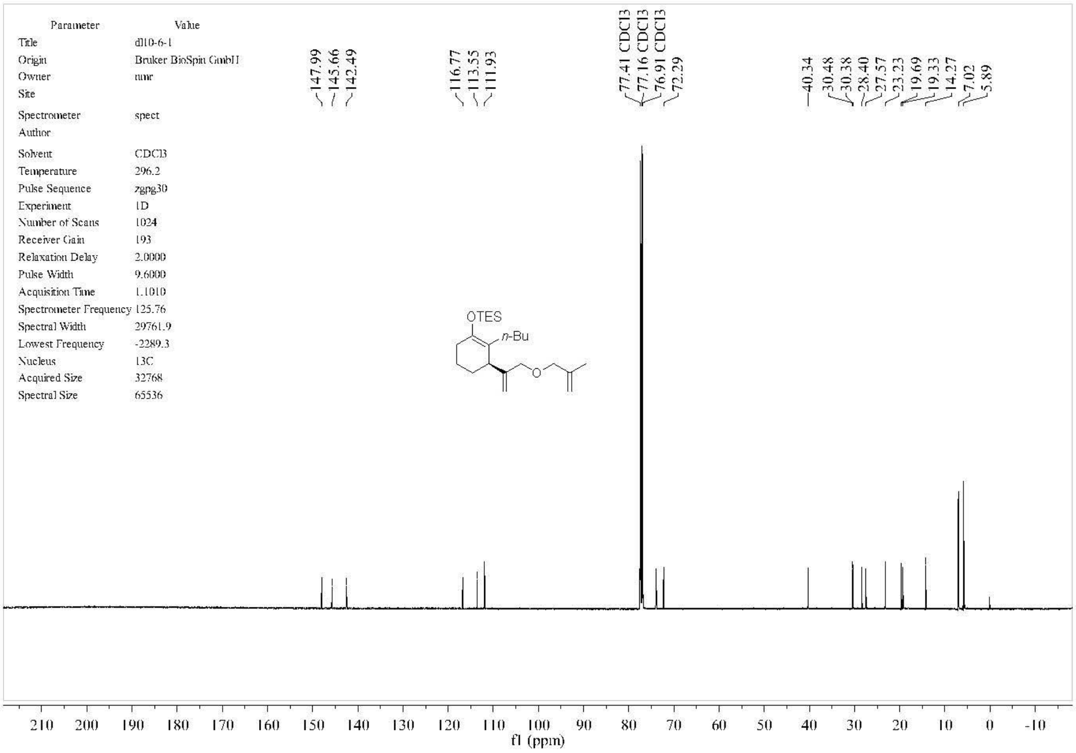

Supplementary Figure 146 ^{13}C NMR of 3ji

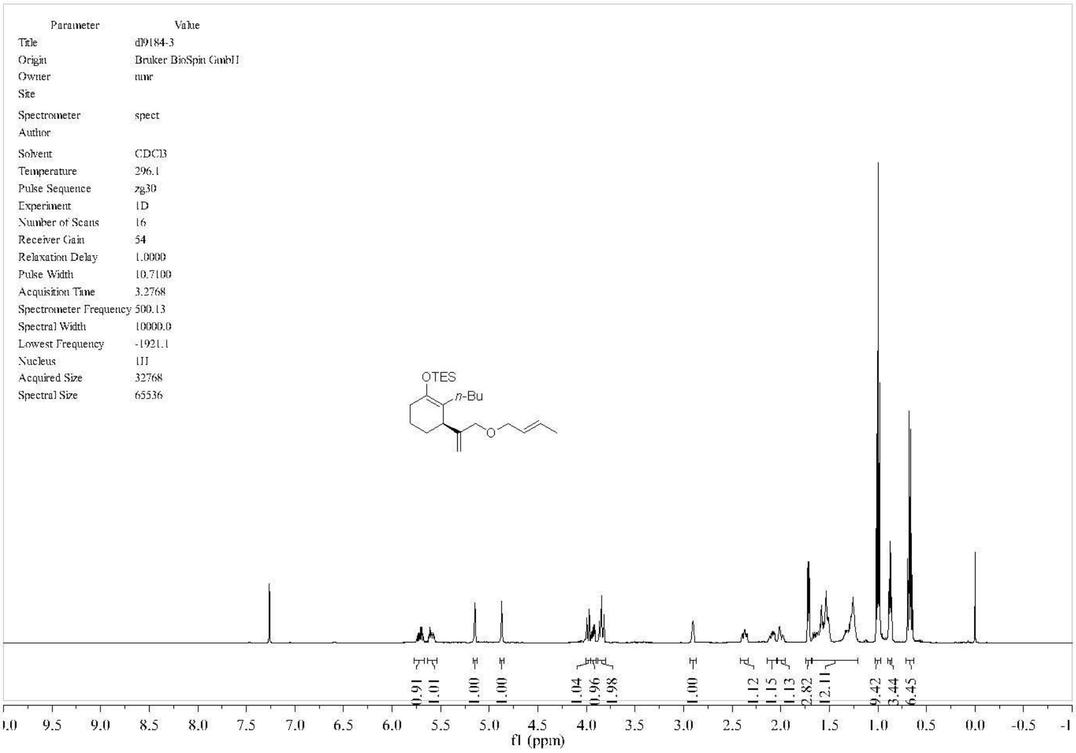

Supplementary Figure 147 ^{1}H NMR of 3jk

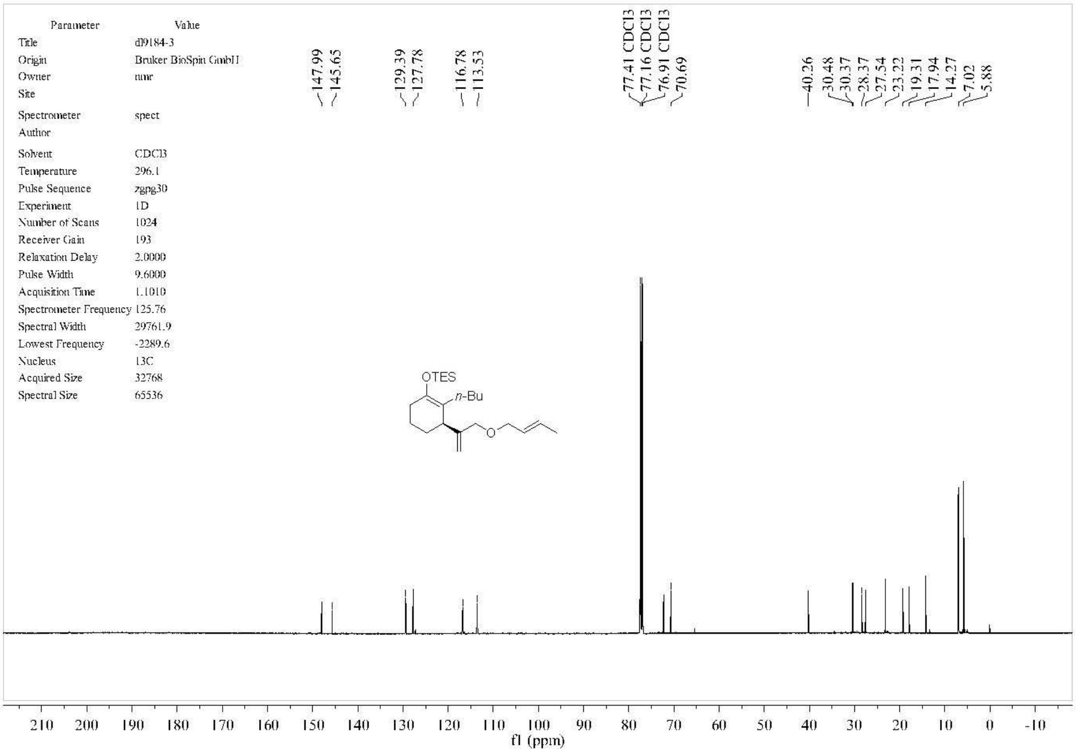

Supplementary Figure 148 ^{13}C NMR of 3jk

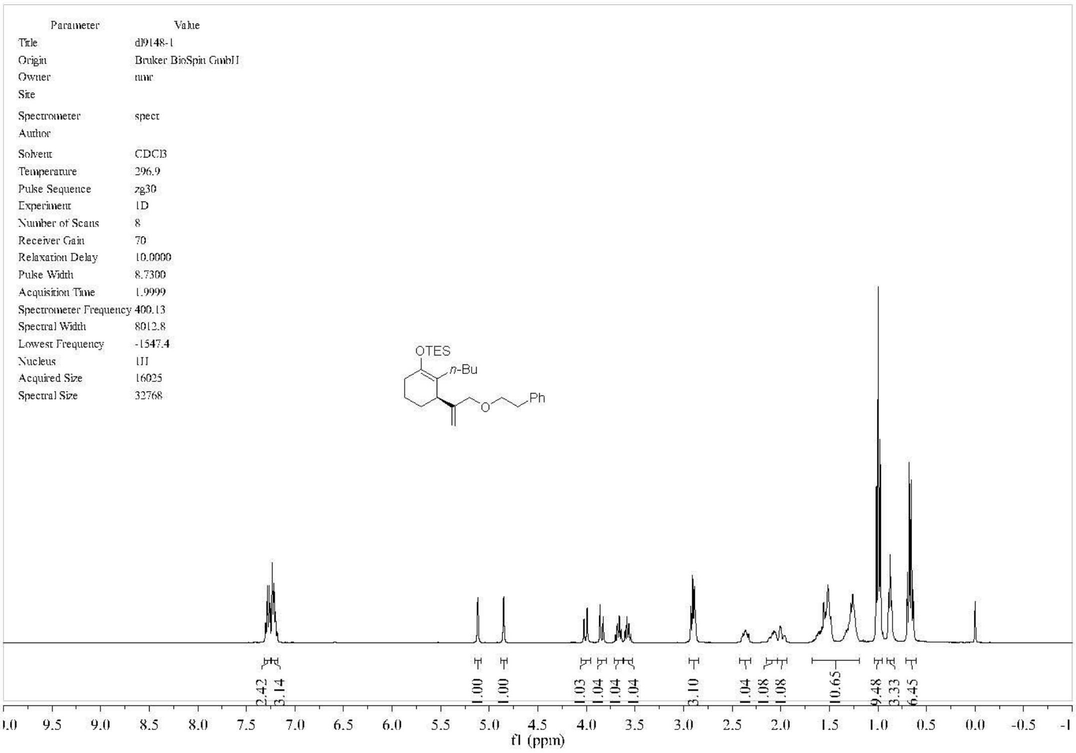

Supplementary Figure 149 ^{1}H NMR of 3jk

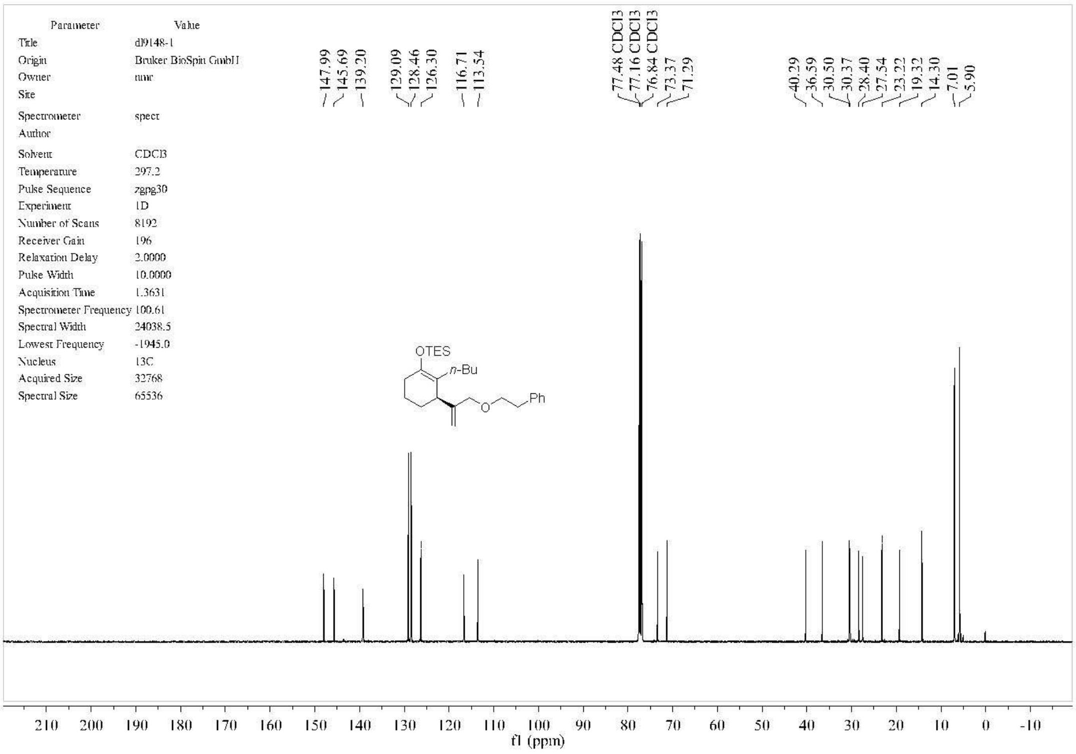

Supplementary Figure 150 ^{13}C NMR of 3jk

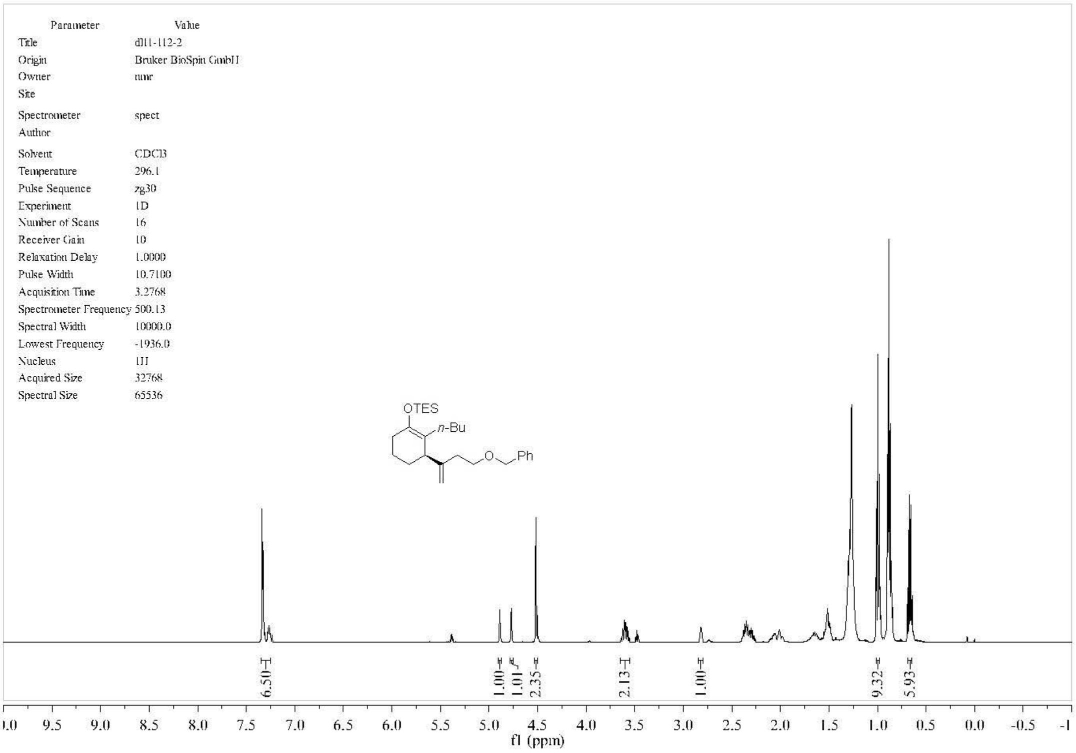

Supplementary Figure 151 ^{1}H NMR of 3jm

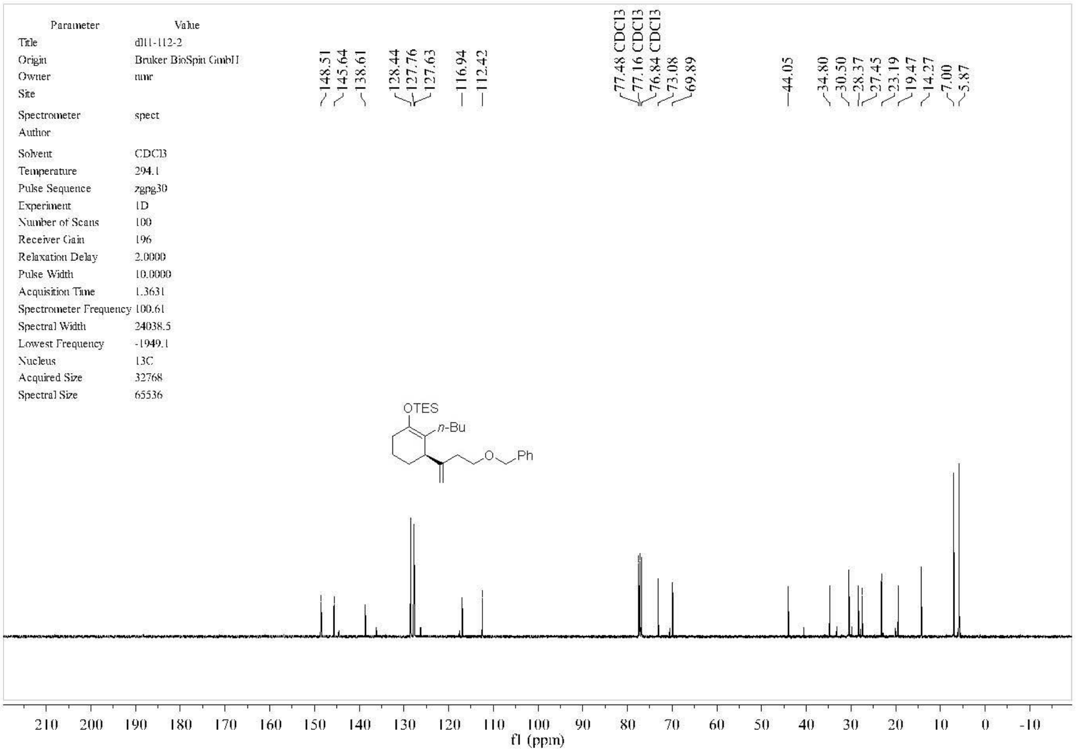

Supplementary Figure 152 ^{13}C NMR of 3jk

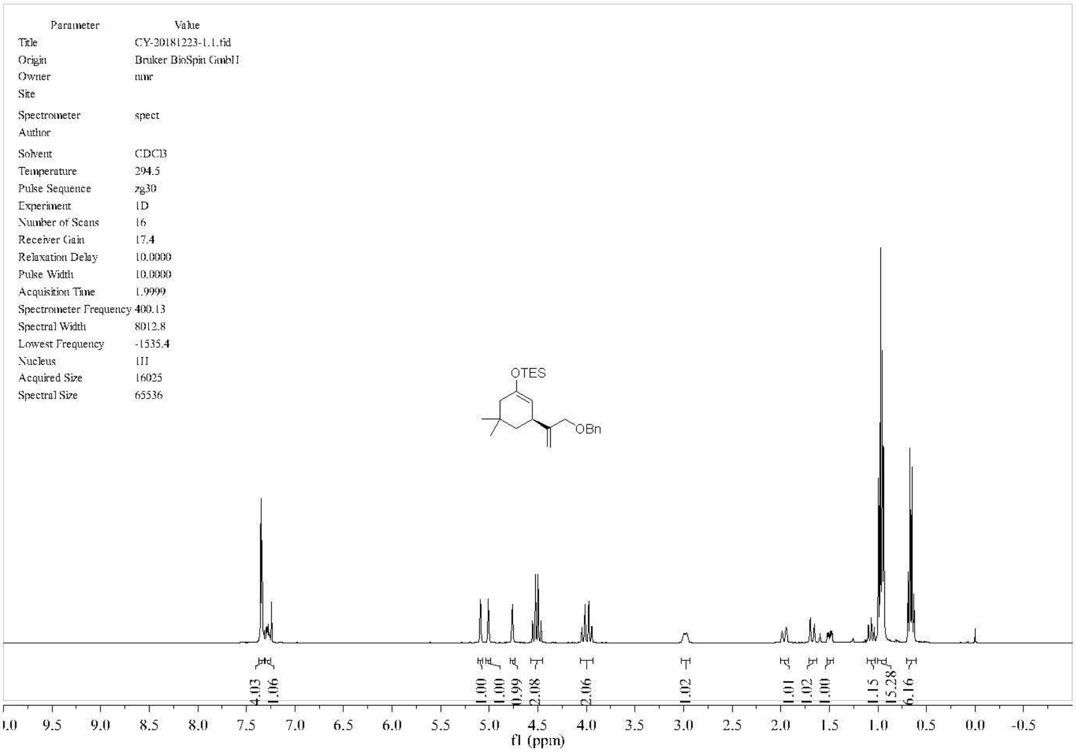

Supplementary Figure 153 ^{1}H NMR of 3qa

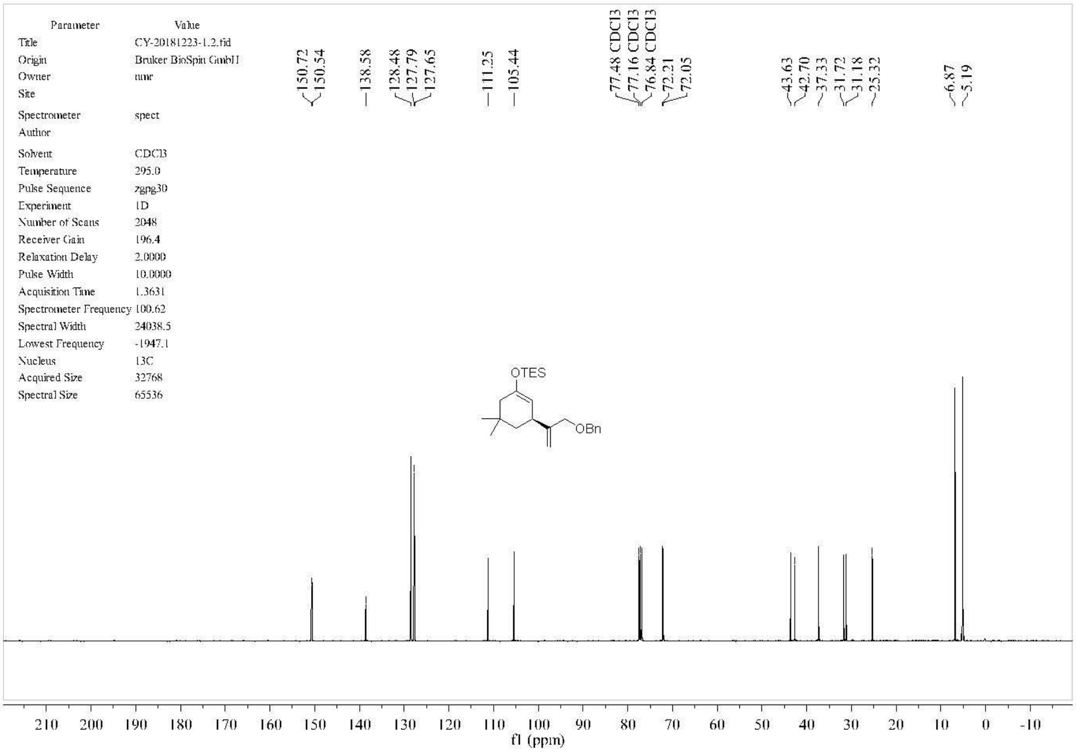

Supplementary Figure 154 ^{13}C NMR of 3qa

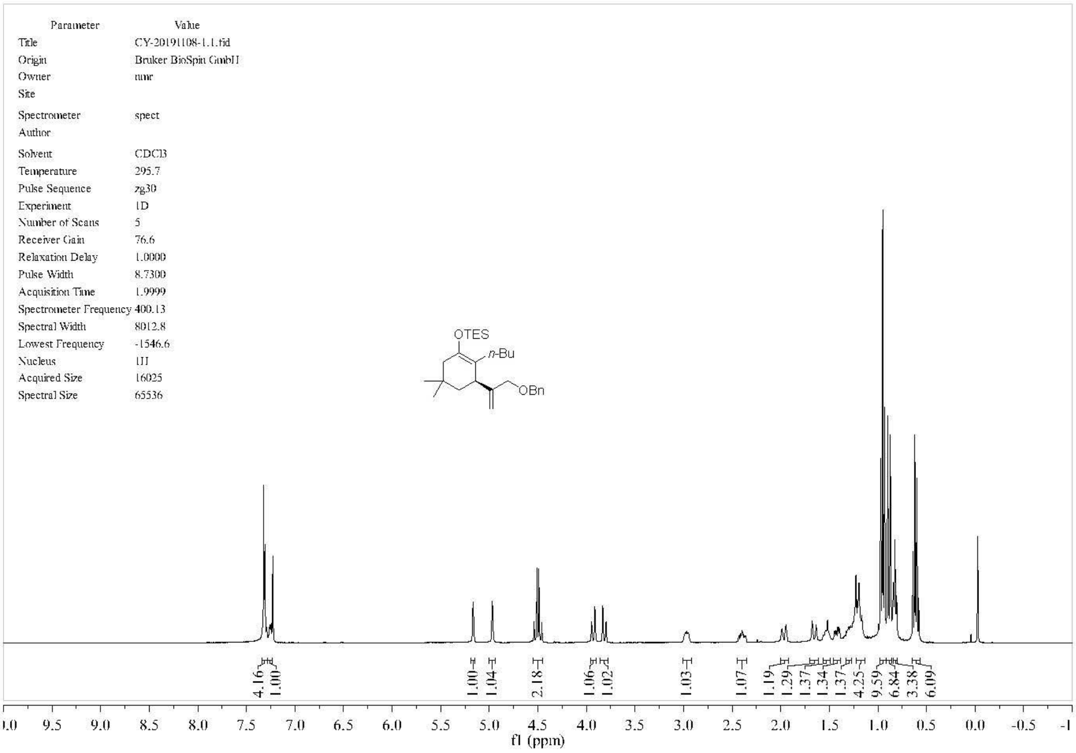

Supplementary Figure 155 ^{1}H NMR of 3ra

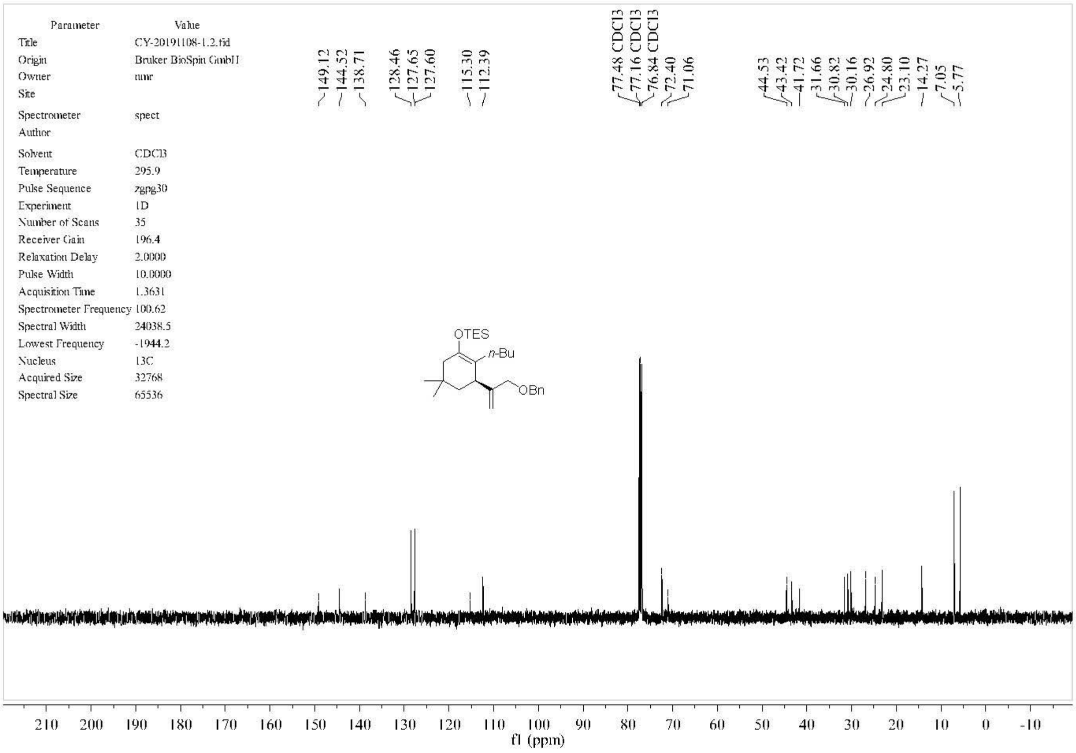

Supplementary Figure 156 ^{13}C NMR of 3ra

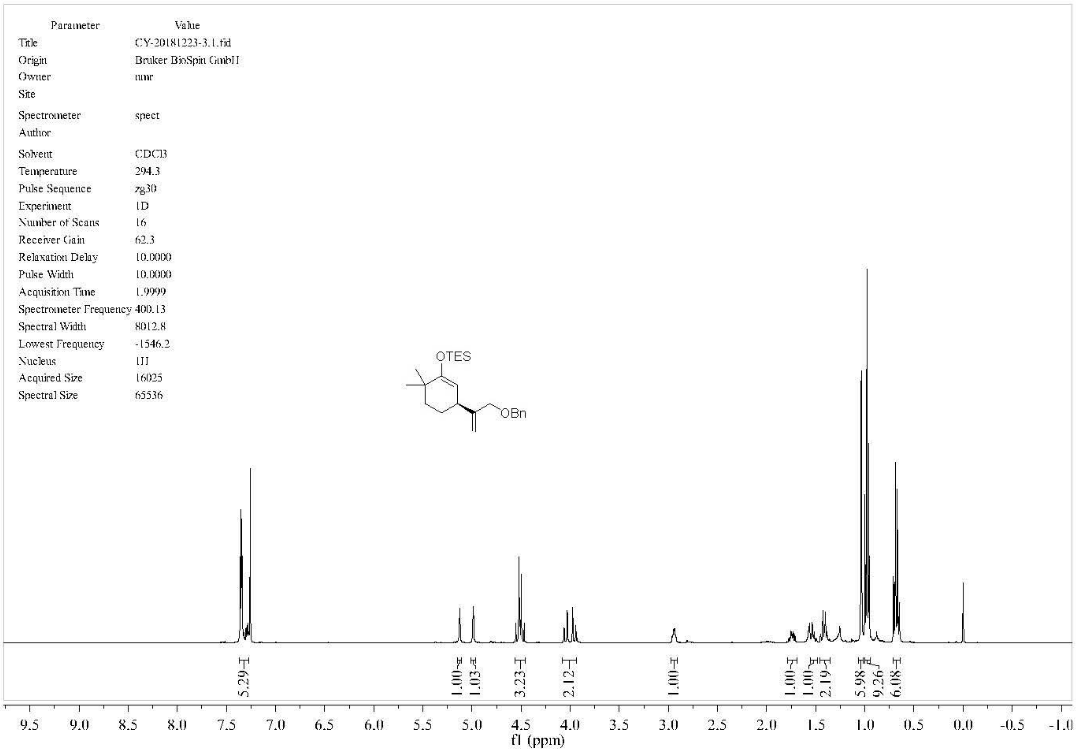

Supplementary Figure 157 ^{1}H NMR of 3sa

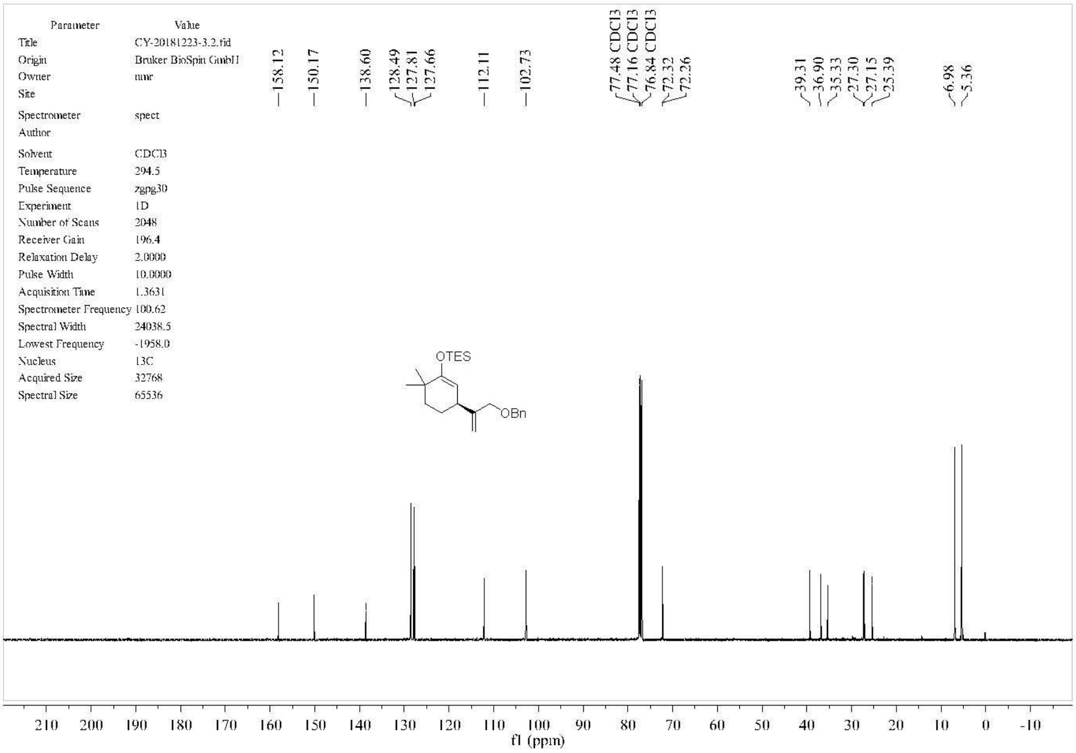

Supplementary Figure 158 ^{13}C NMR of 3sa

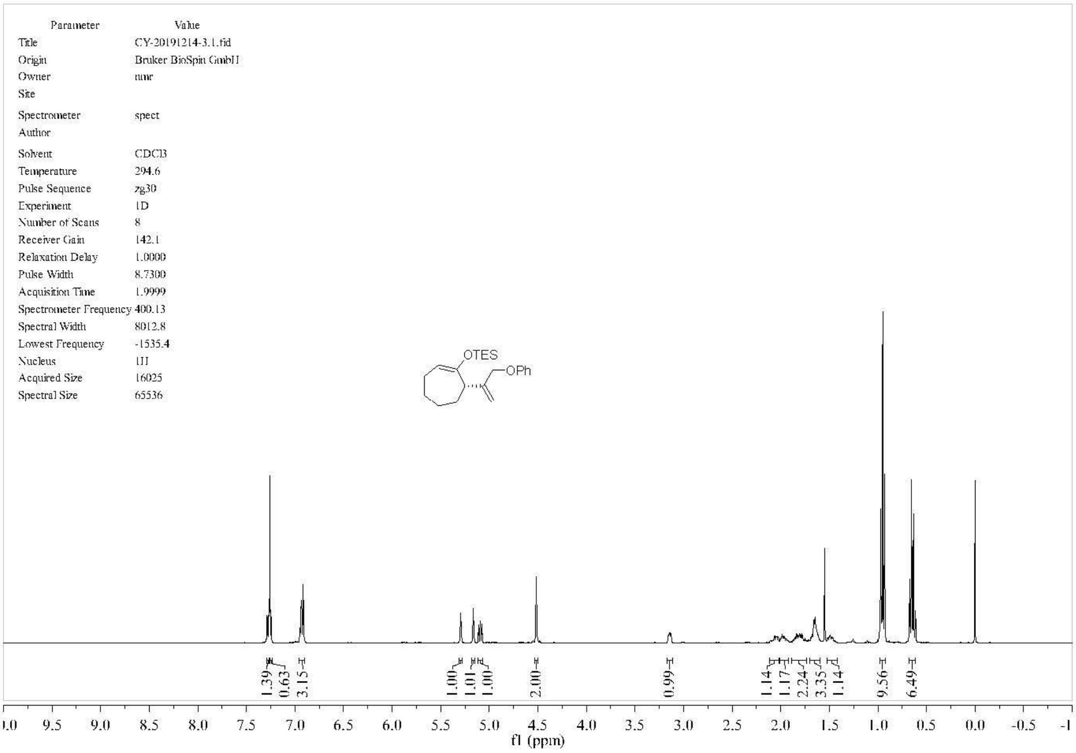

Supplementary Figure 159 ^{1}H NMR of 3tb

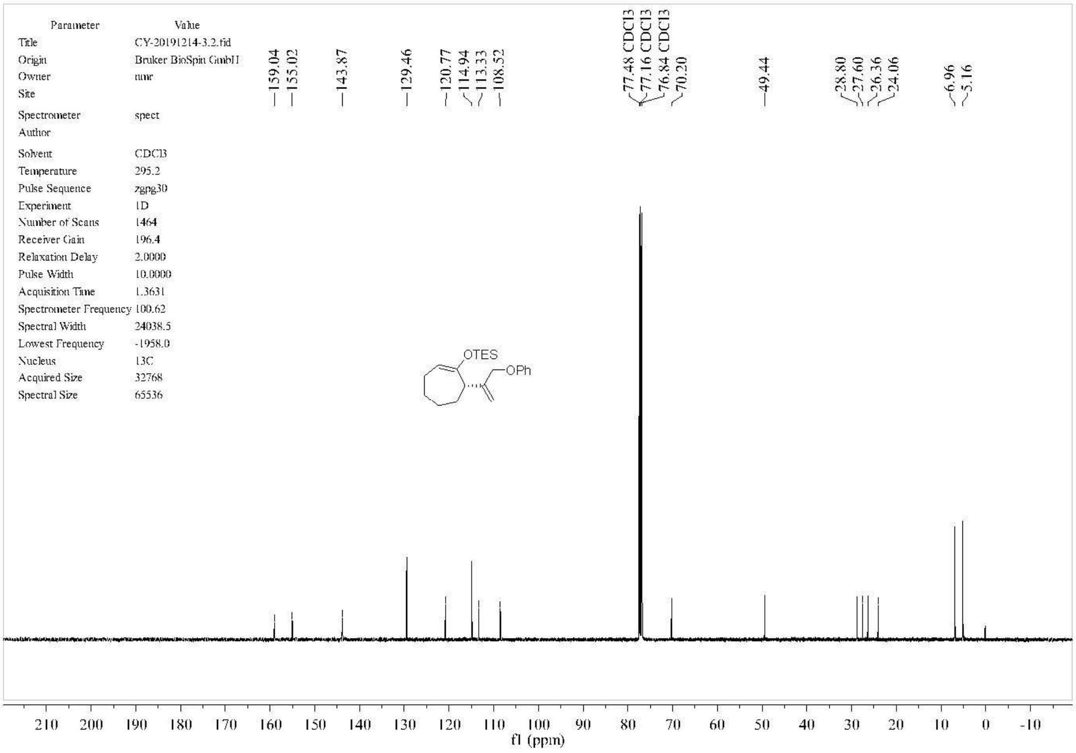

Supplementary Figure 160 ^{13}C NMR of 3tb

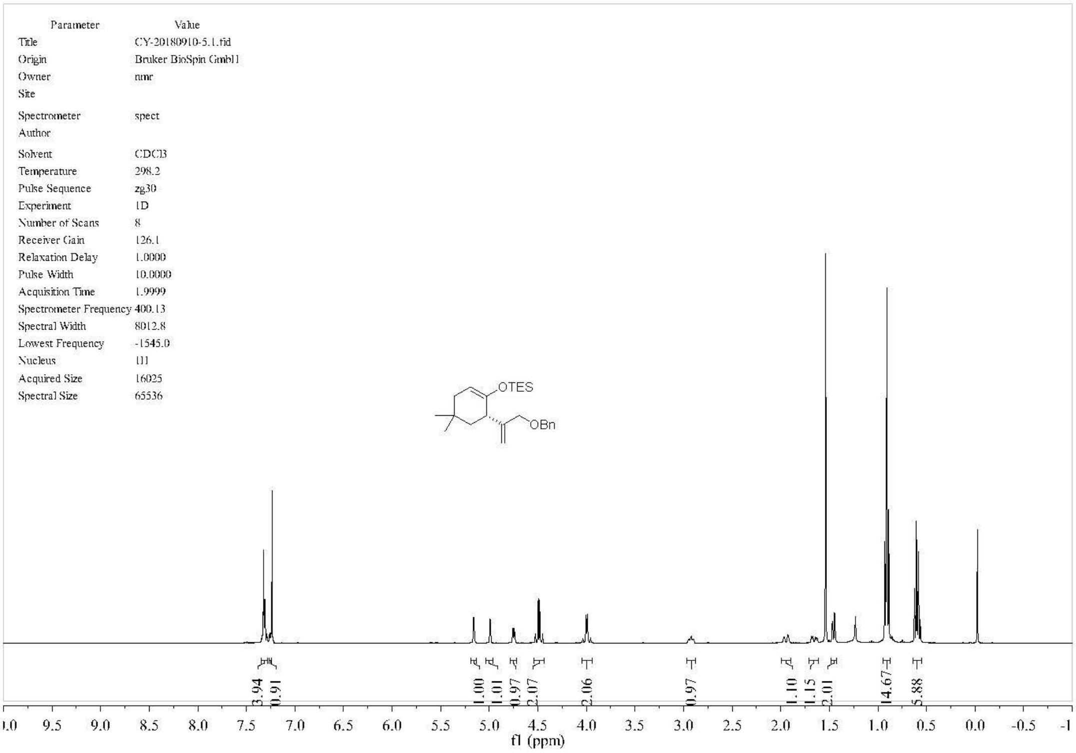

Supplementary Figure 161 ^{1}H NMR of 3ua

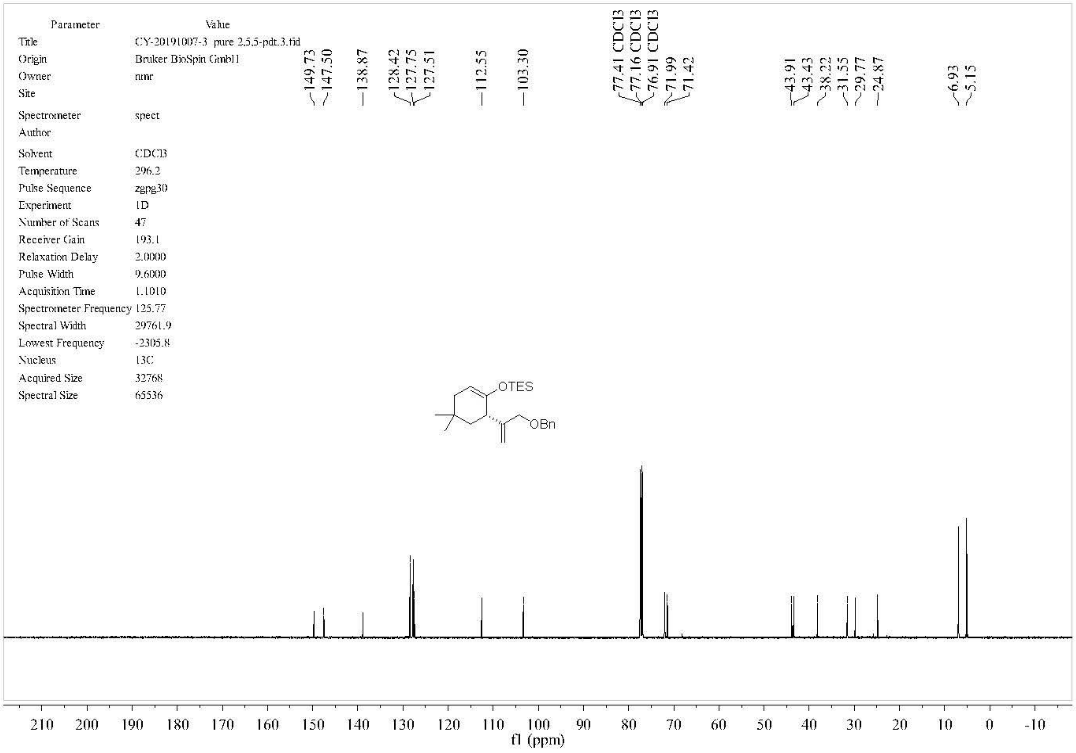

Supplementary Figure 162 ^{13}C NMR of 3ua

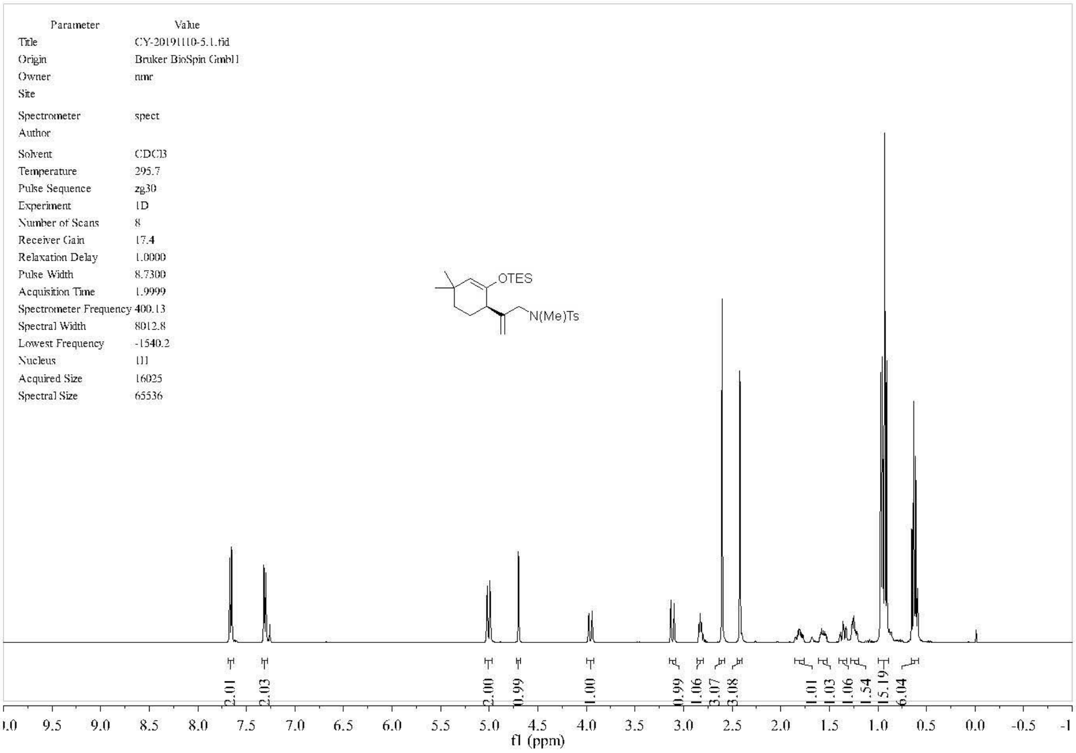

Supplementary Figure 163 ^{1}H NMR of 3vc

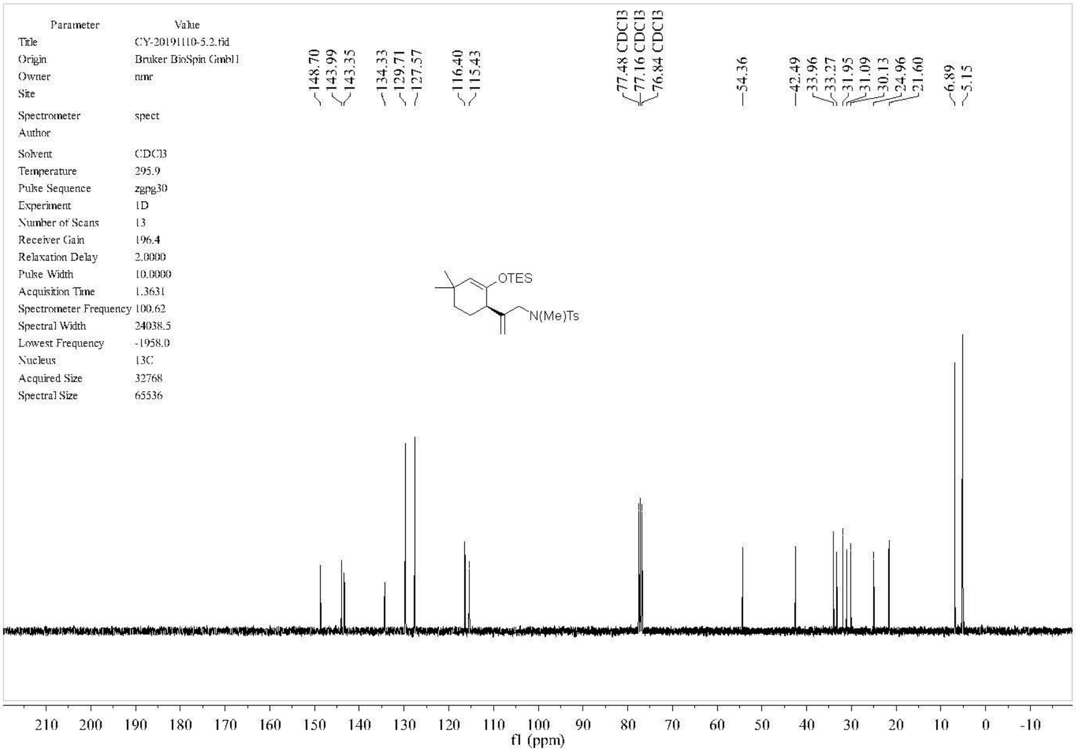

Supplementary Figure 164 ^{13}C NMR of 3vc

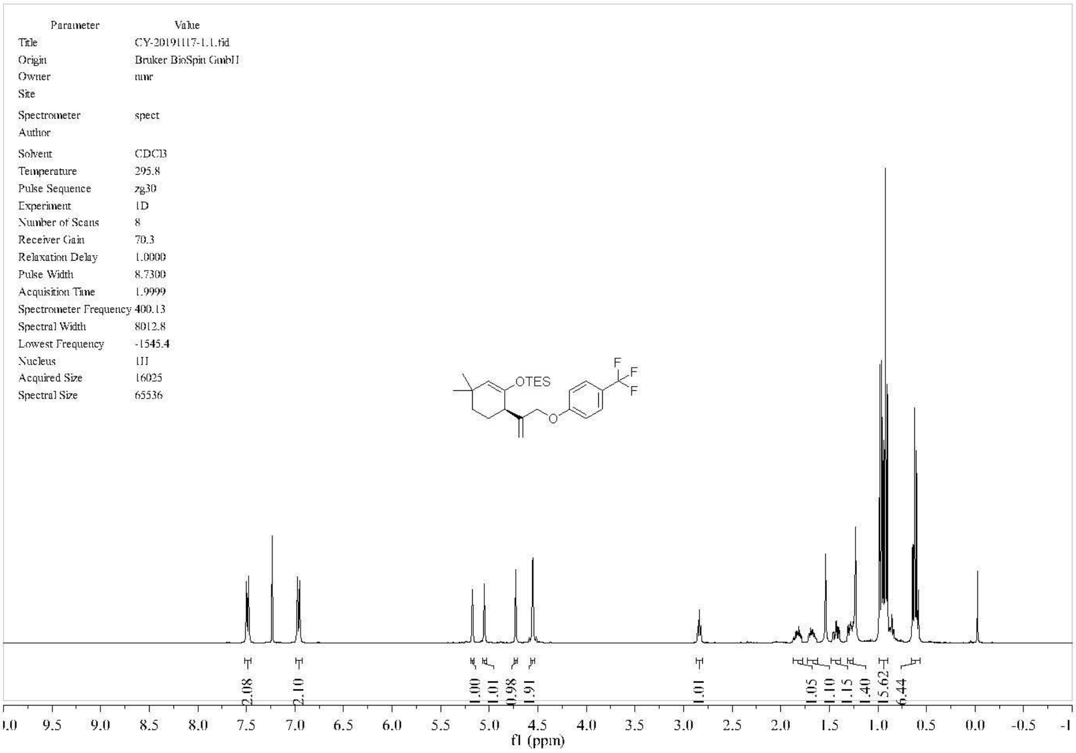

Supplementary Figure 165 ^{1}H NMR of 3vn

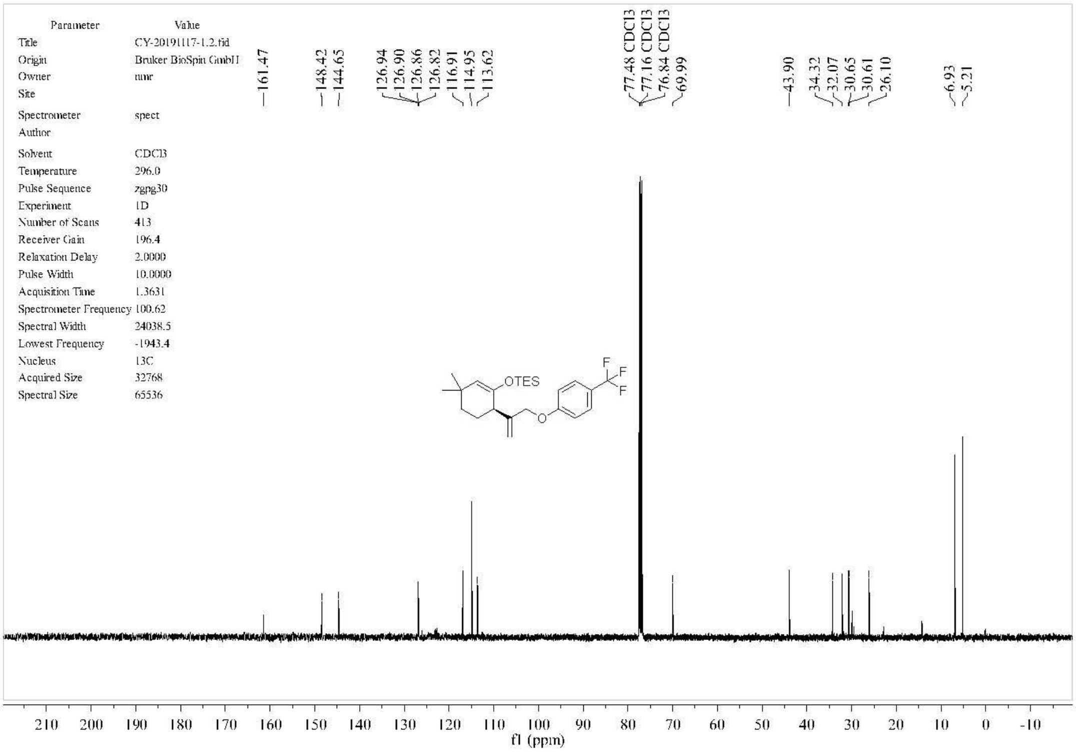

Supplementary Figure 166 ^{13}C NMR of 3vn

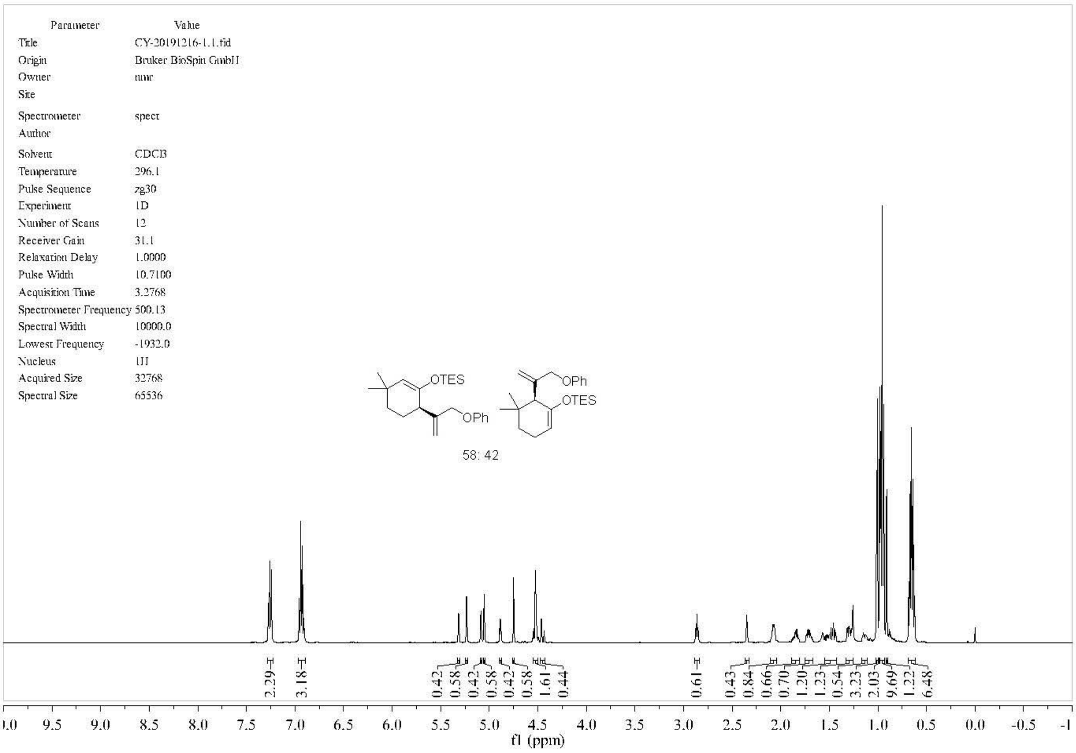

Supplementary Figure 167 ^{1}H NMR of 3vb

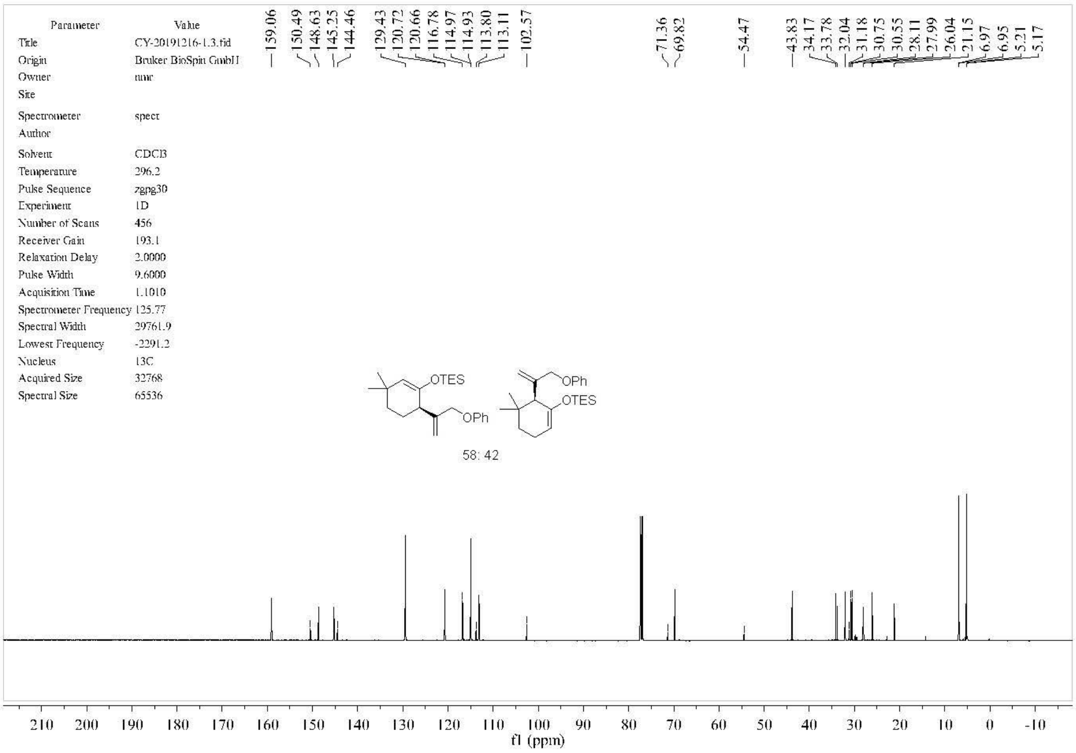

Supplementary Figure 168 ^{13}C NMR of 3vb

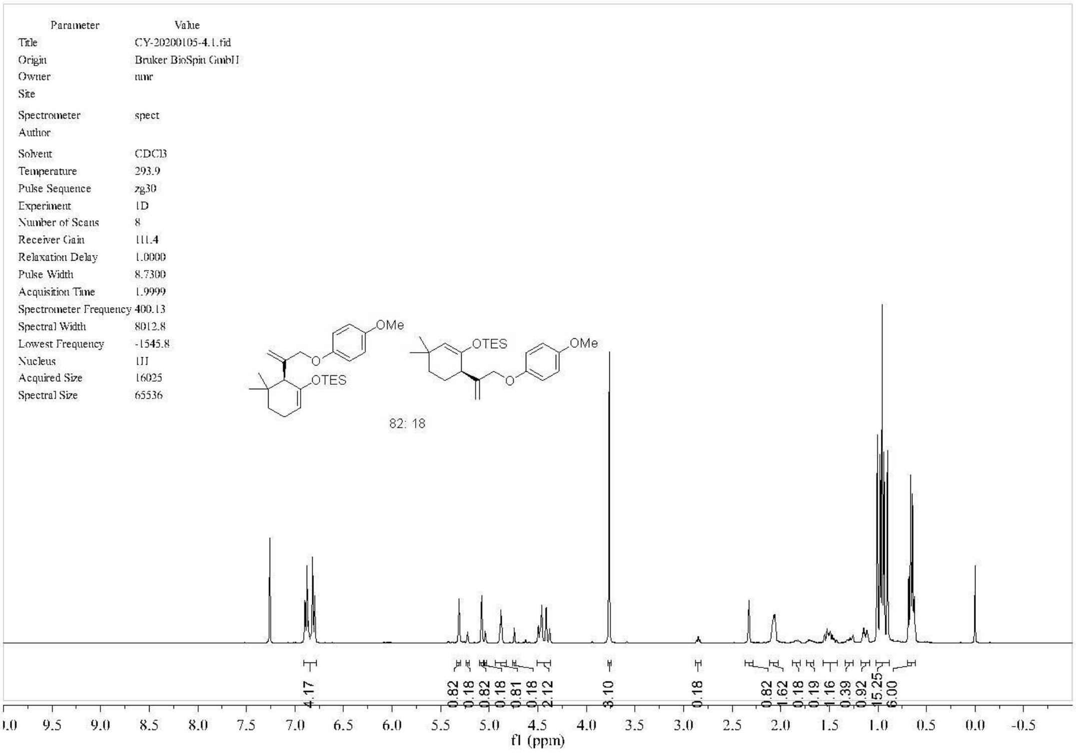

Supplementary Figure 169 ^{1}H NMR of 3vo

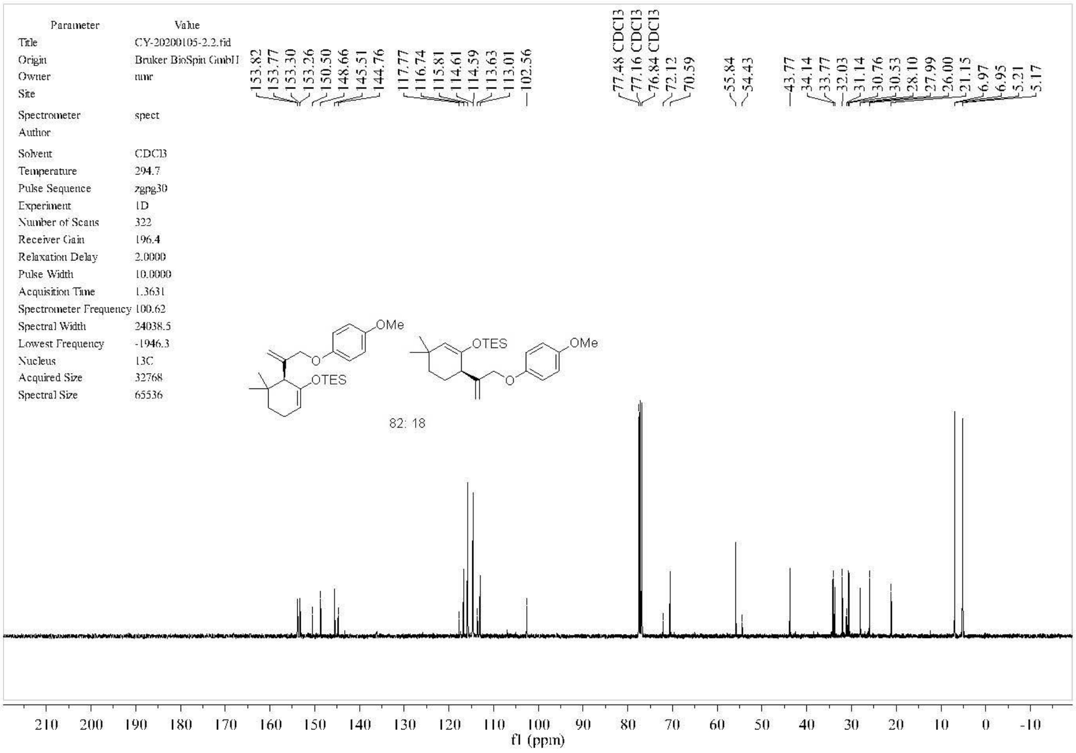

Supplementary Figure 170 ^{13}C NMR of 3vo

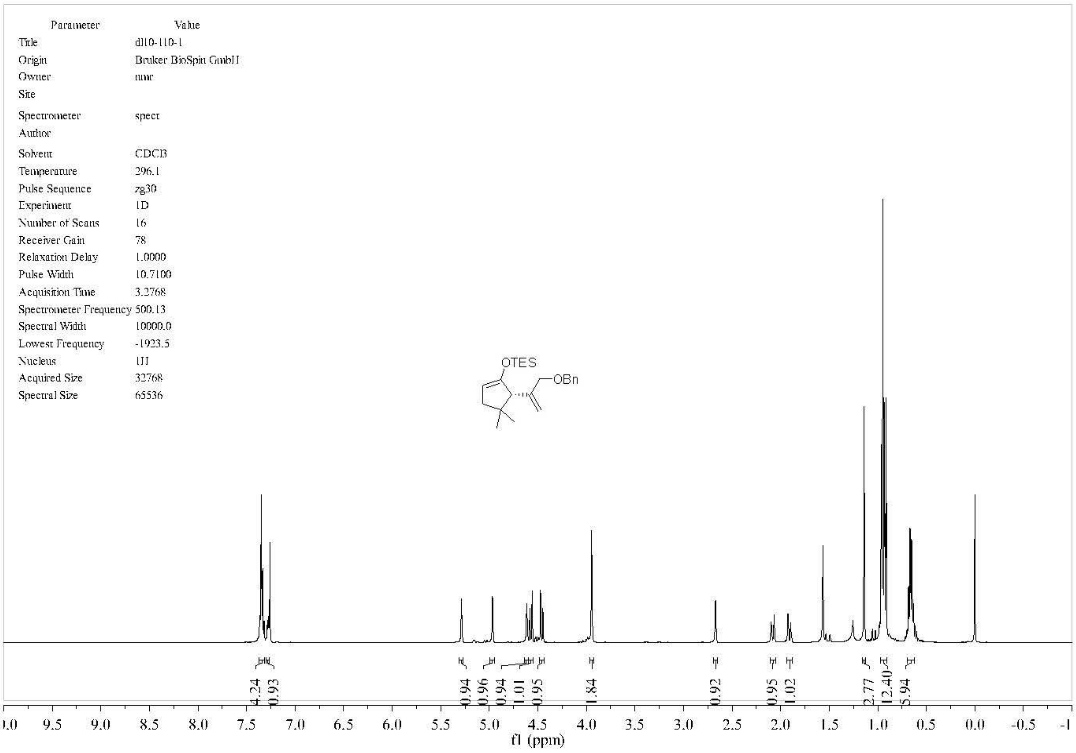

Supplementary Figure 171 ^{1}H NMR of 3wa

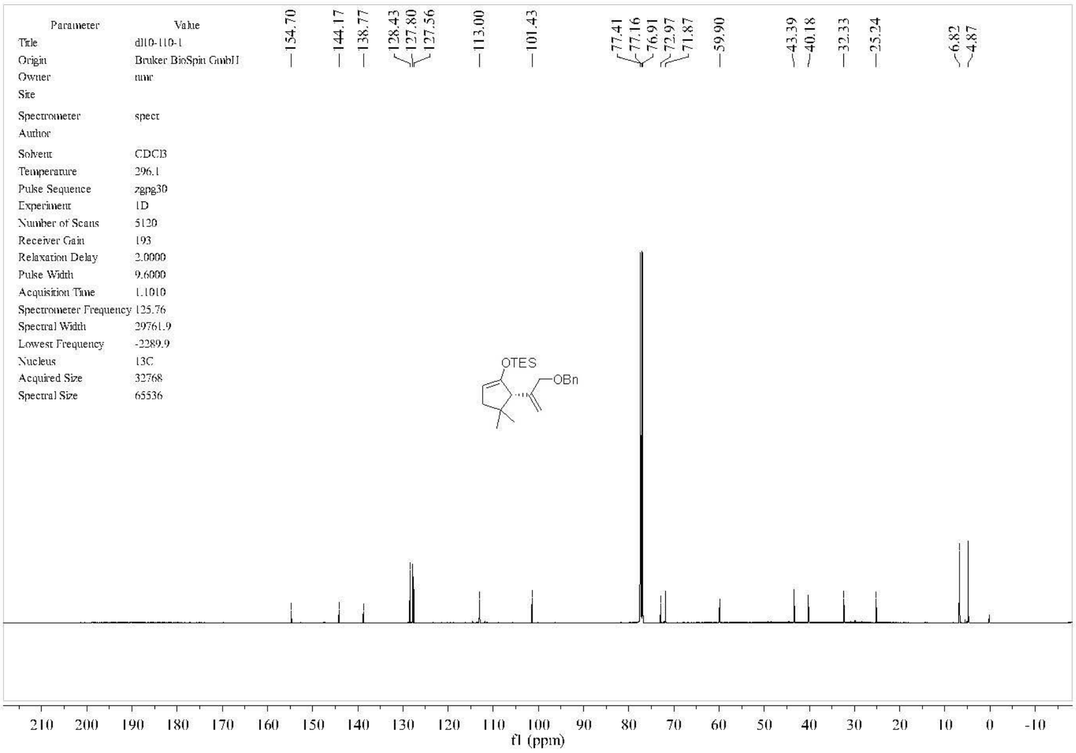

Supplementary Figure 172 ^{13}C NMR of 3wa

Deprotection Product

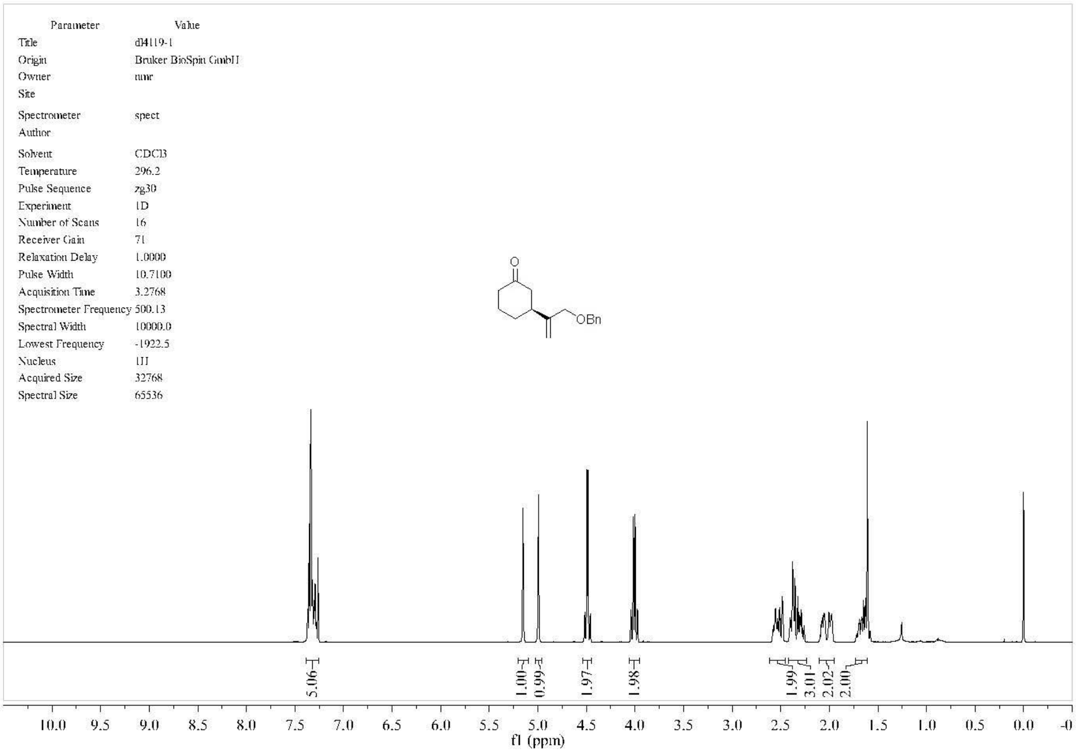

Supplementary Figure 173 ^{1}H NMR of 3ea’s deprotection product

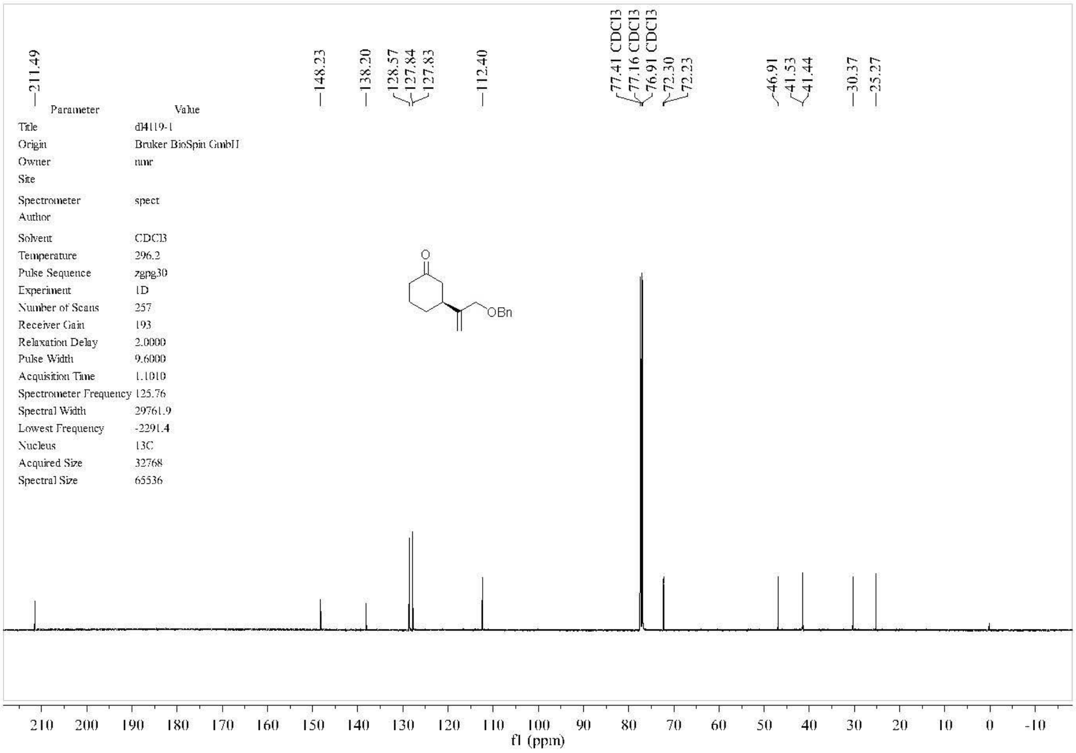

Supplementary Figure 174 ^{13}C NMR of 3ea's deprotection product

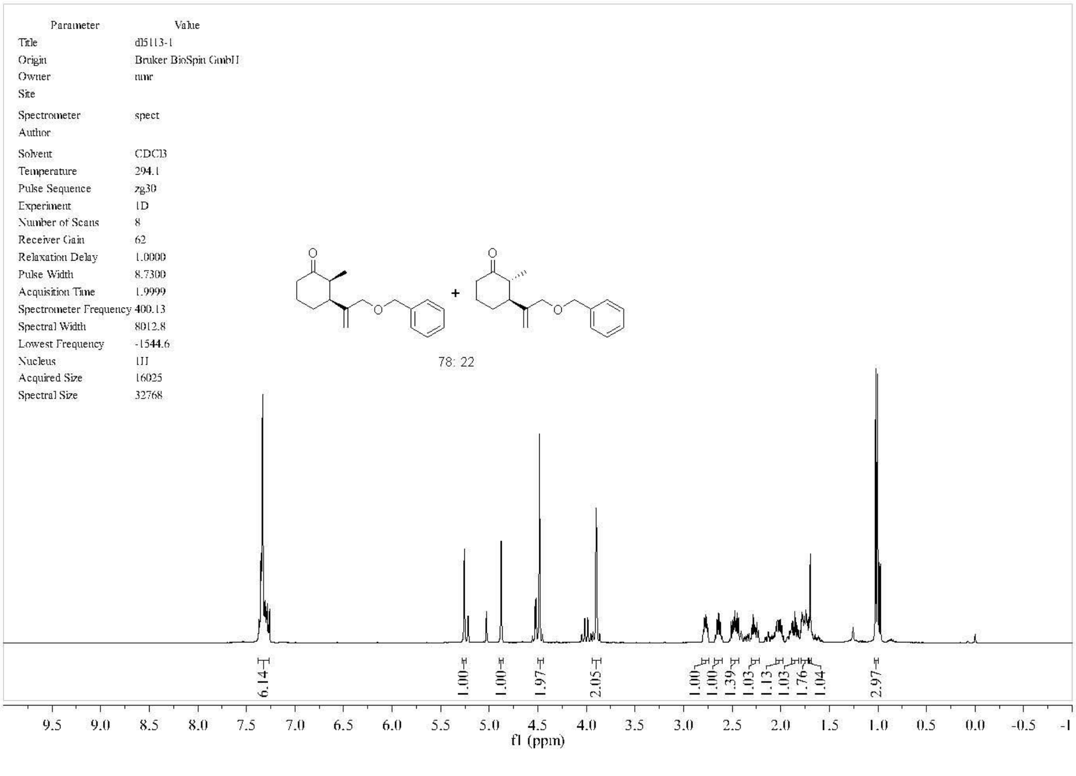

Supplementary Figure 175 ^{1}H NMR of 3ia's deprotection product (*syn*-)

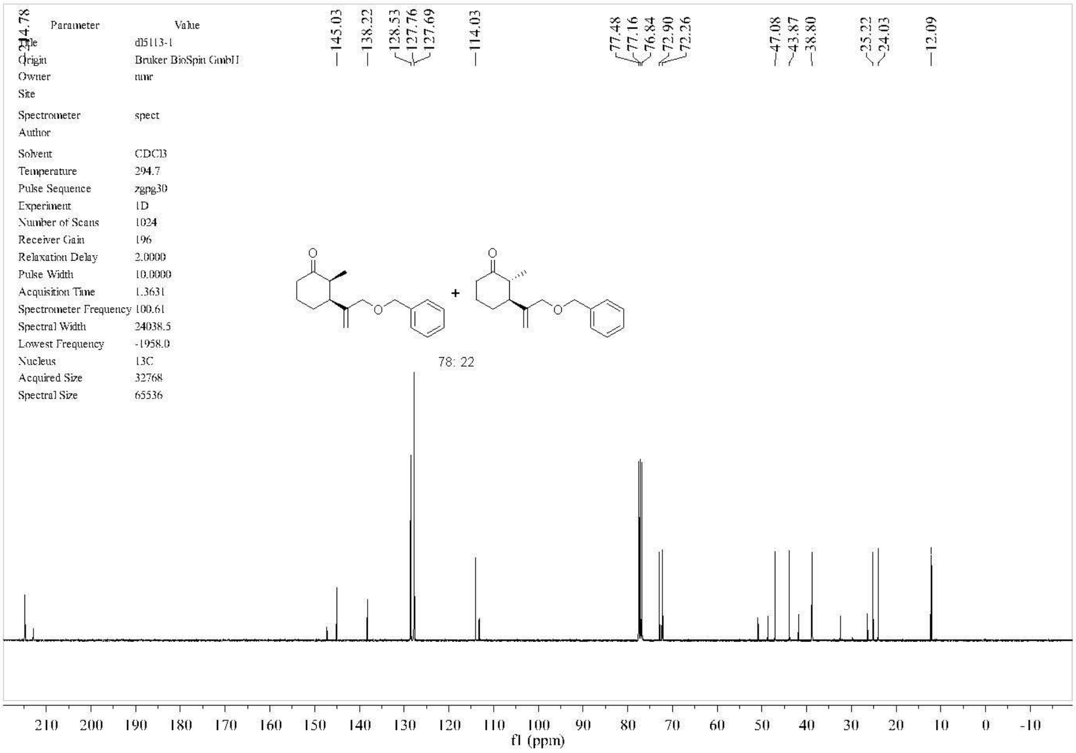

Supplementary Figure 176 ^{13}C NMR of 3ia's deprotection product (*syn-*)

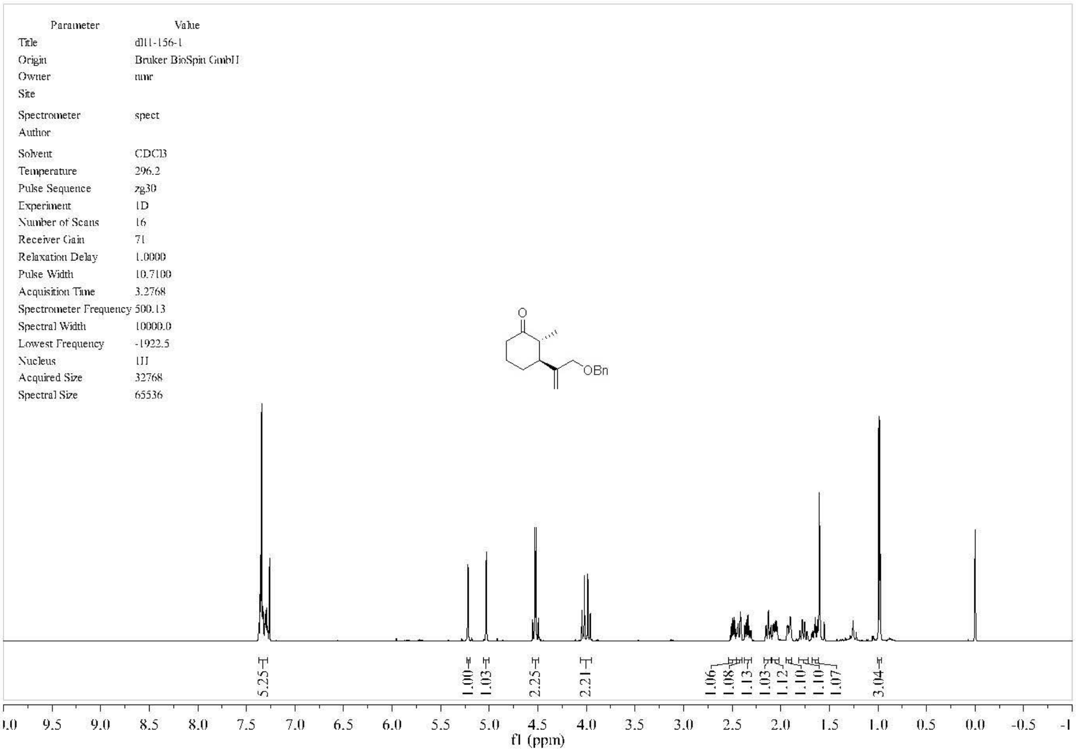

Supplementary Figure 177 ^{1}H NMR of 3ia's deprotection product (*anti-*)

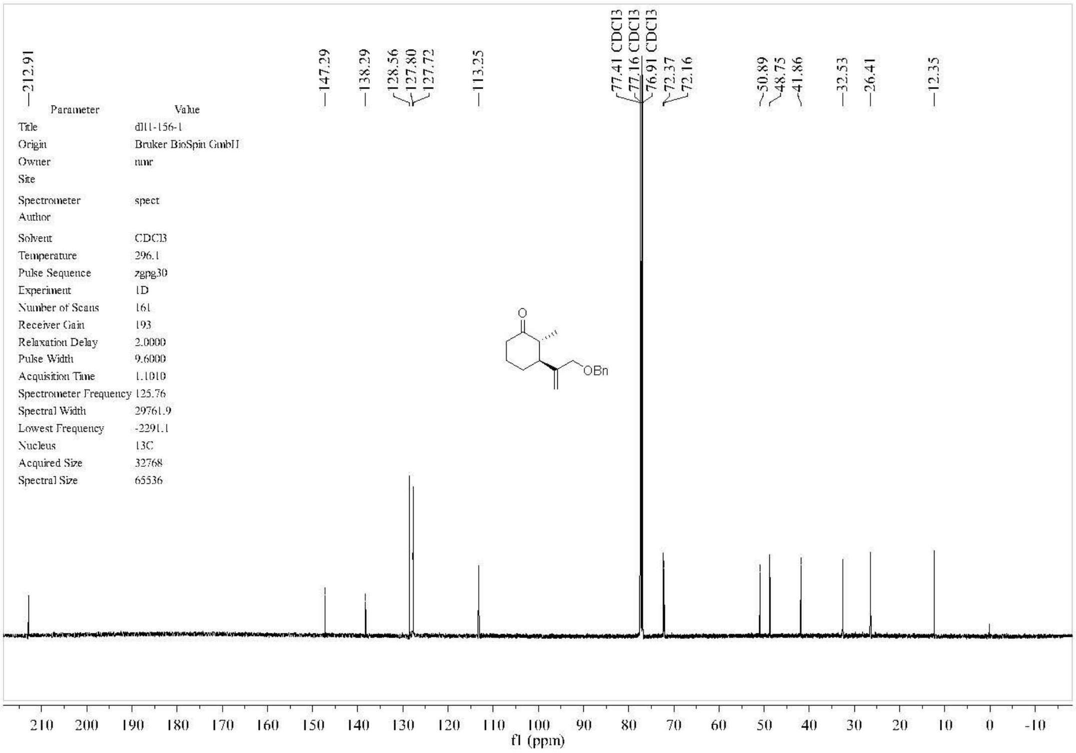

Supplementary Figure 178 ^{13}C NMR of 3ia's deprotection product (*anti*-)

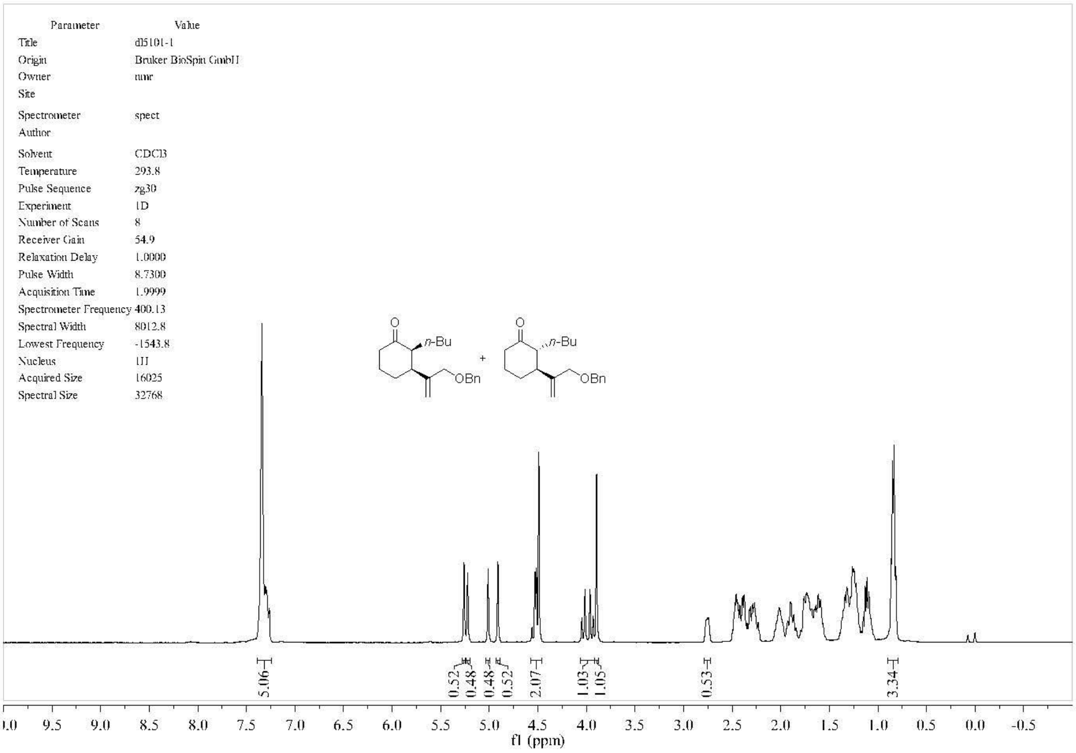

Supplementary Figure 179 ^{1}H NMR of 3ja's deprotection product

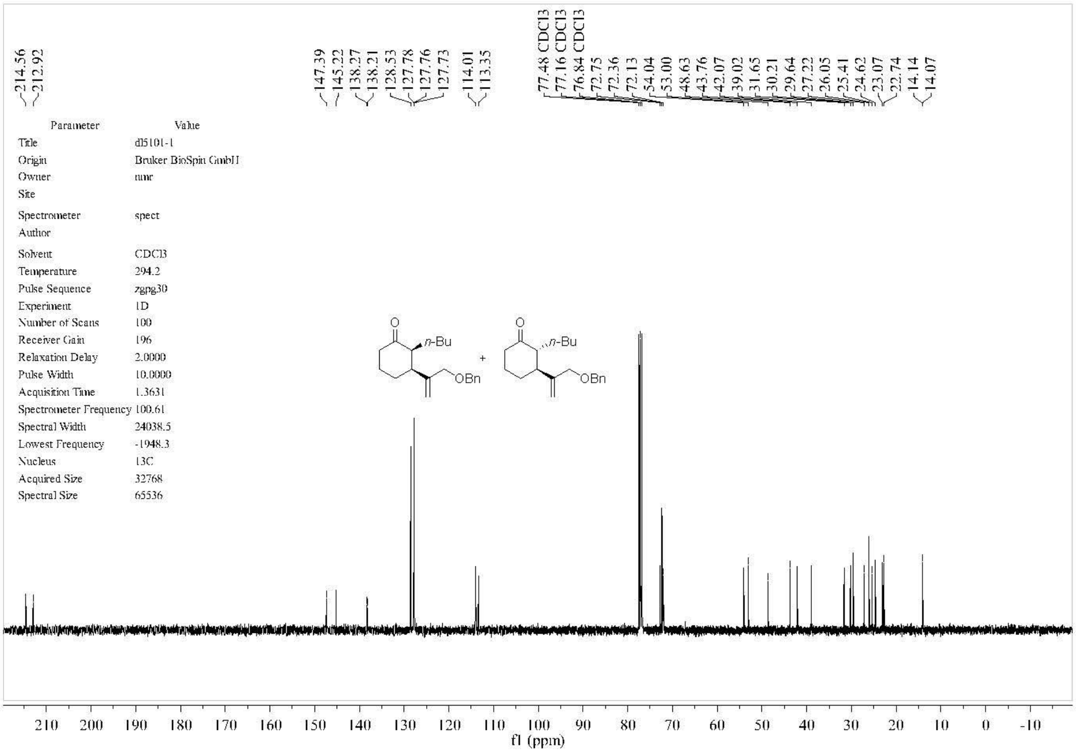

Supplementary Figure 180 ^{13}C NMR of 3ja's deprotection product

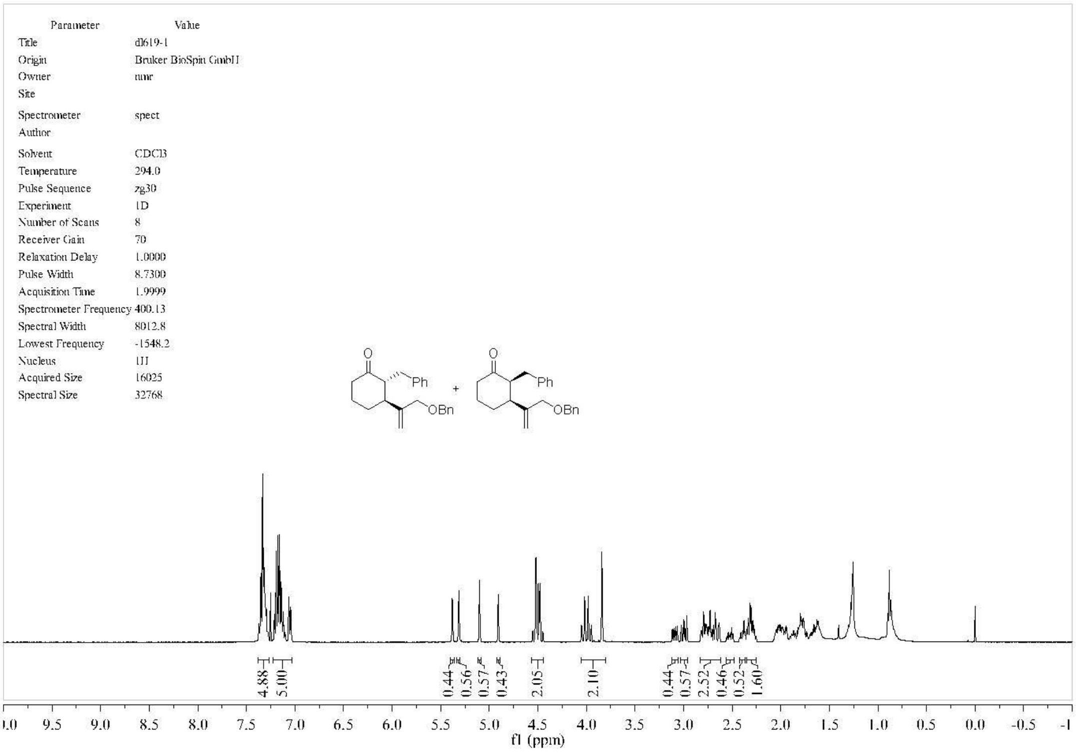

Supplementary Figure 181 ^{1}H NMR of 3la's deprotection product

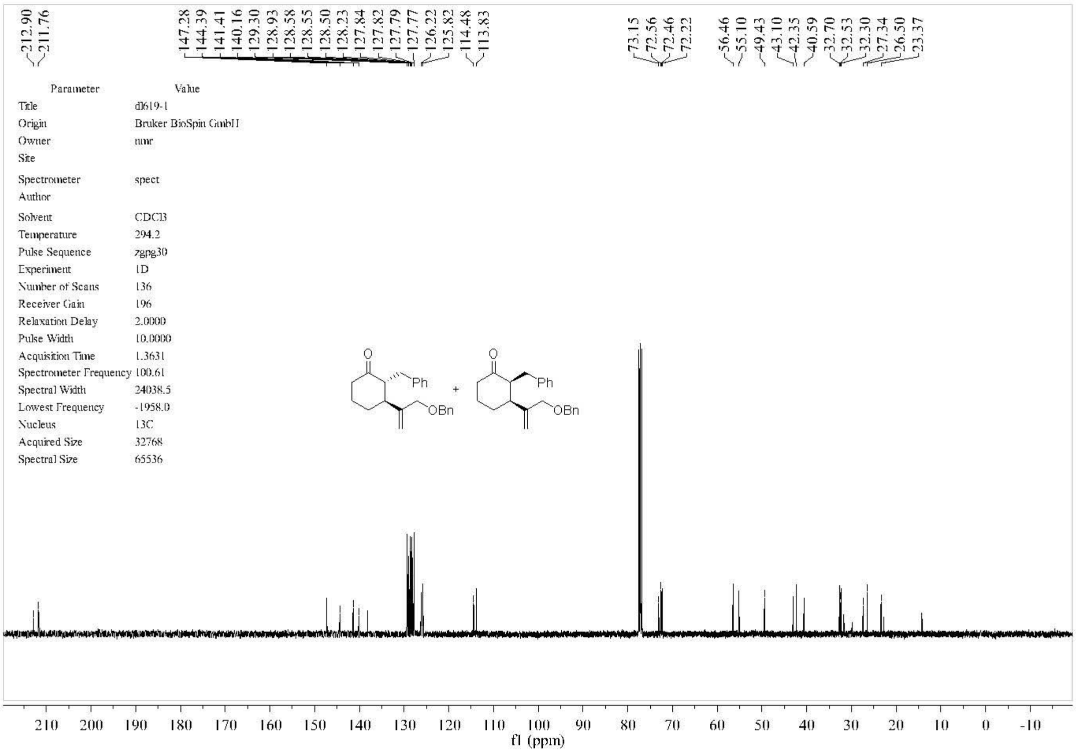

Supplementary Figure 182 ^{13}C NMR of 3la's deprotection product

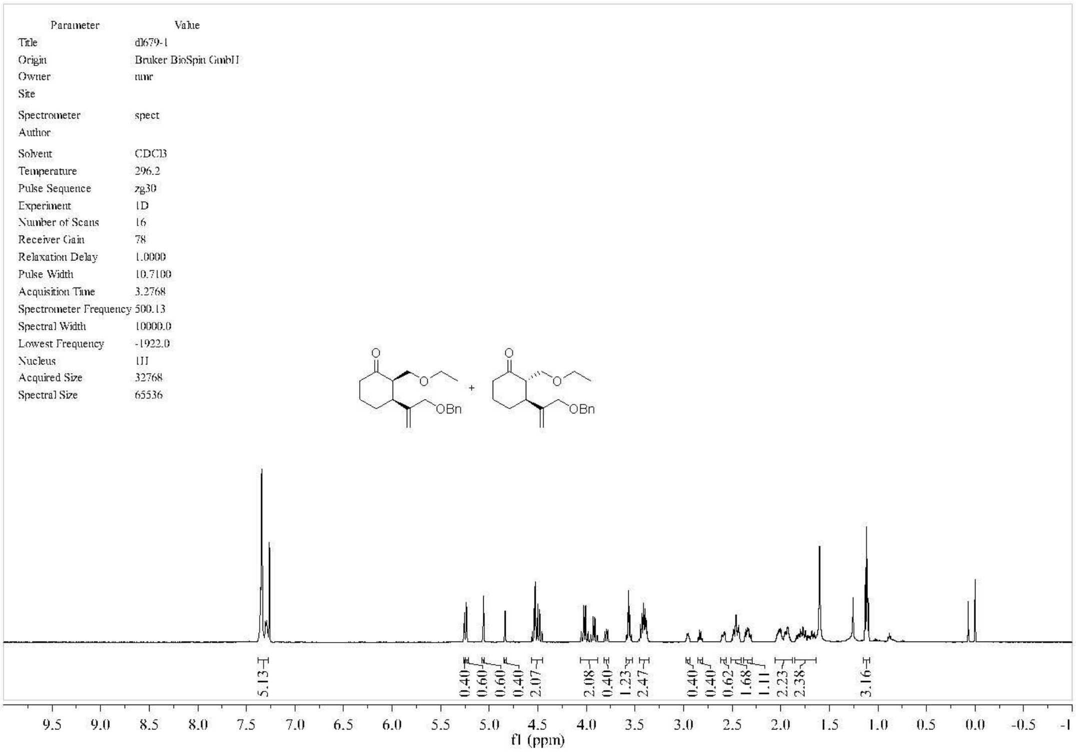

Supplementary Figure 183 ^{1}H NMR of 3ma's deprotection product

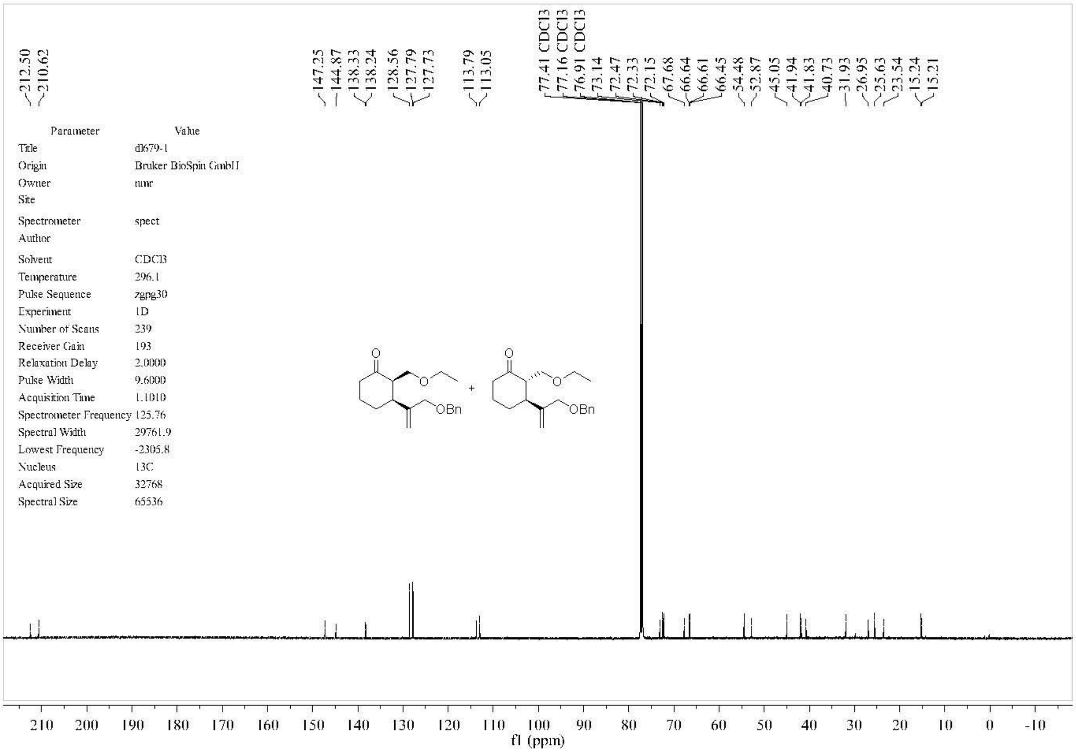

Supplementary Figure 184 ^{13}C NMR of 3ma's deprotection product

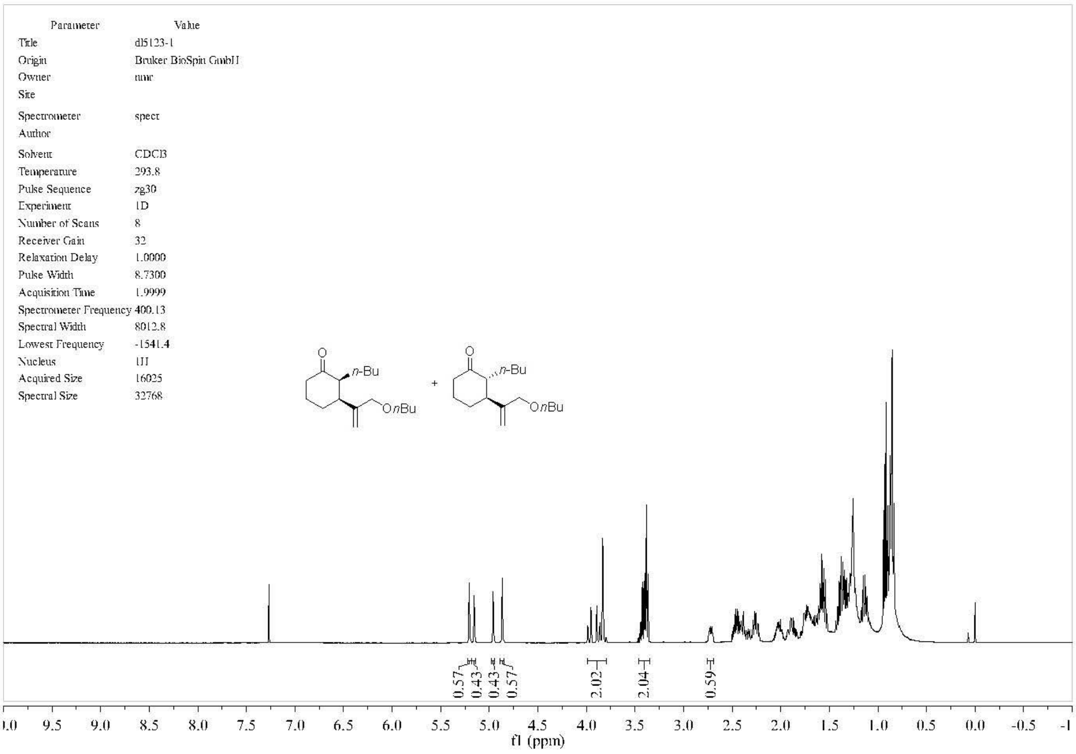

Supplementary Figure 185 ^{1}H NMR of 3jd's deprotection product

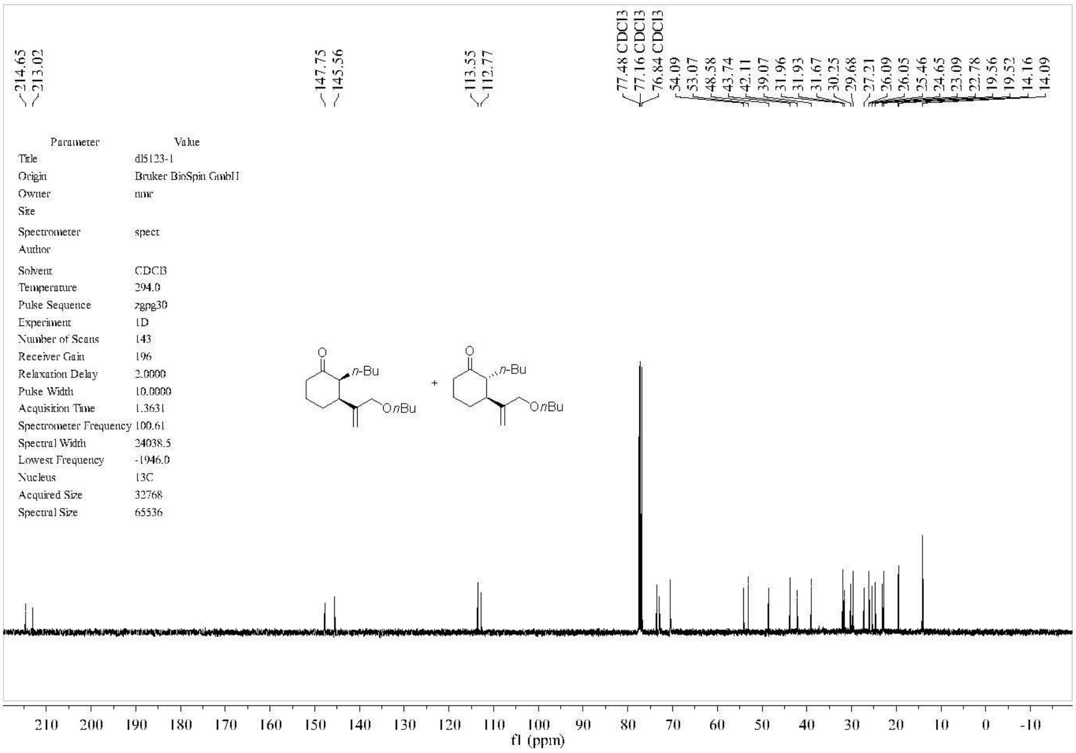

Supplementary Figure 186 ^{13}C NMR of 3jd's deprotection product

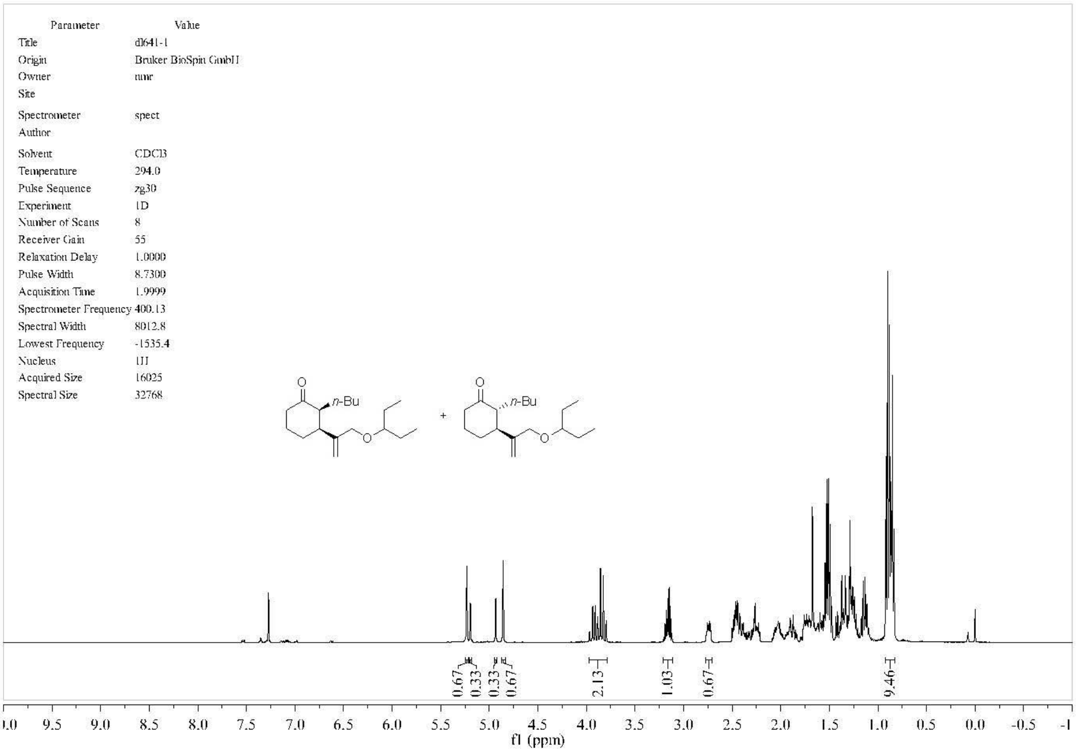

Supplementary Figure 187 ^{1}H NMR of 3jf's deprotection product

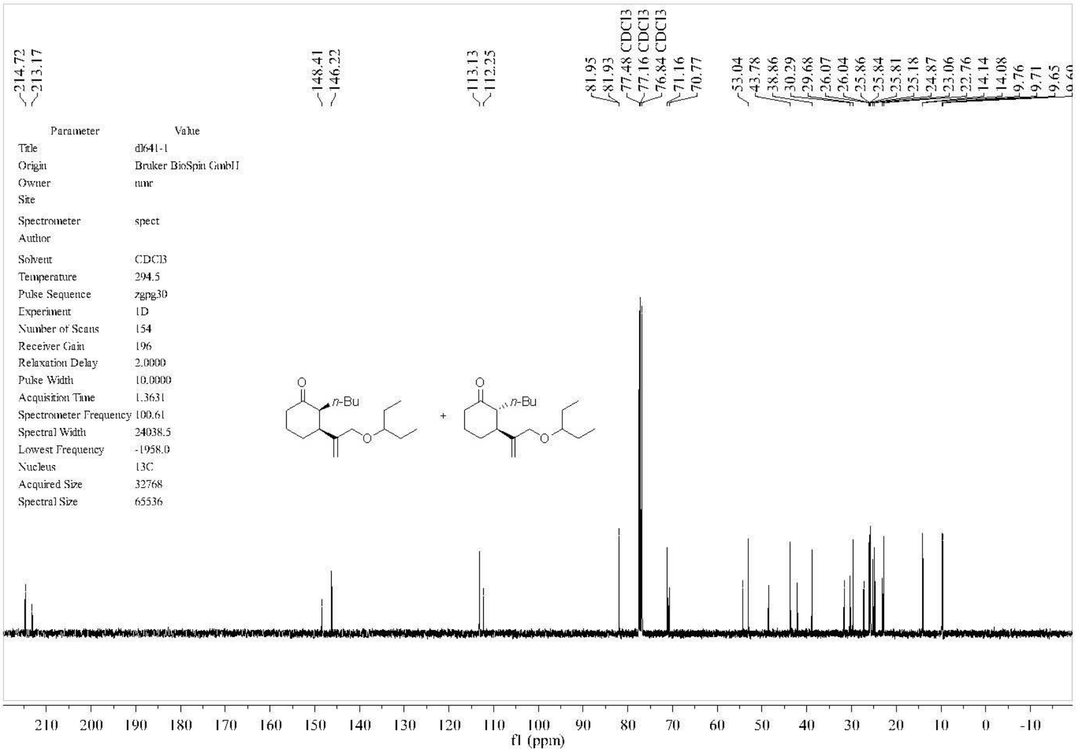

Supplementary Figure 188 ^{13}C NMR of 3jf's deprotection product

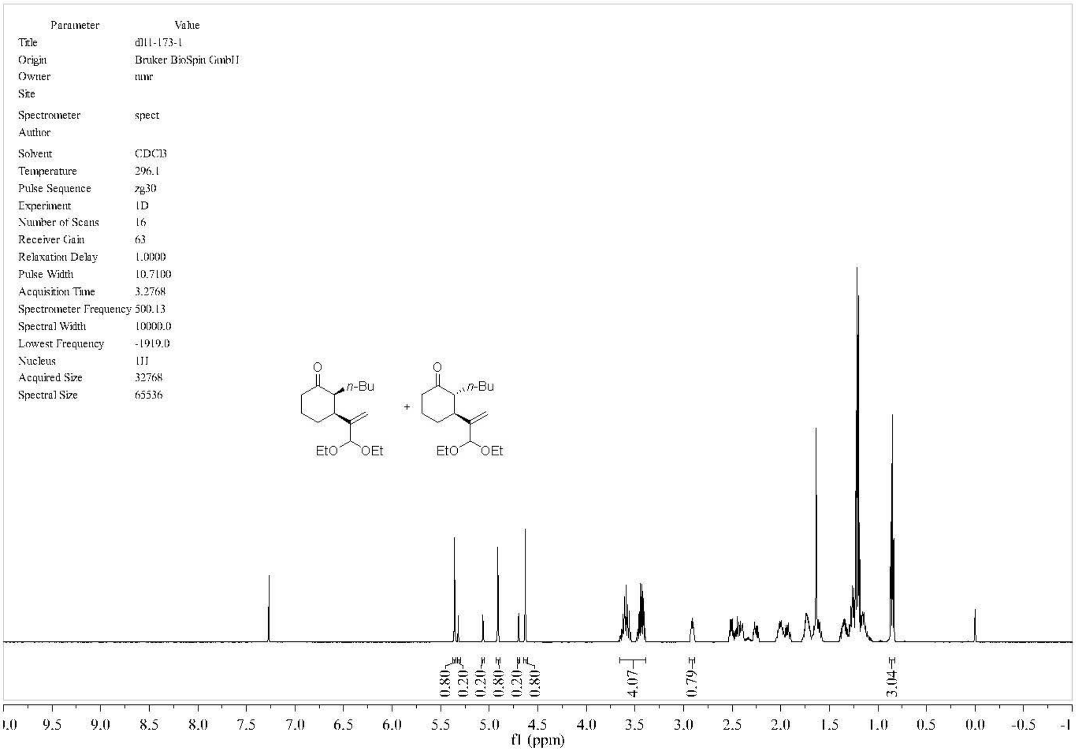

Supplementary Figure 189 ^{1}H NMR of 3ji's deprotection product

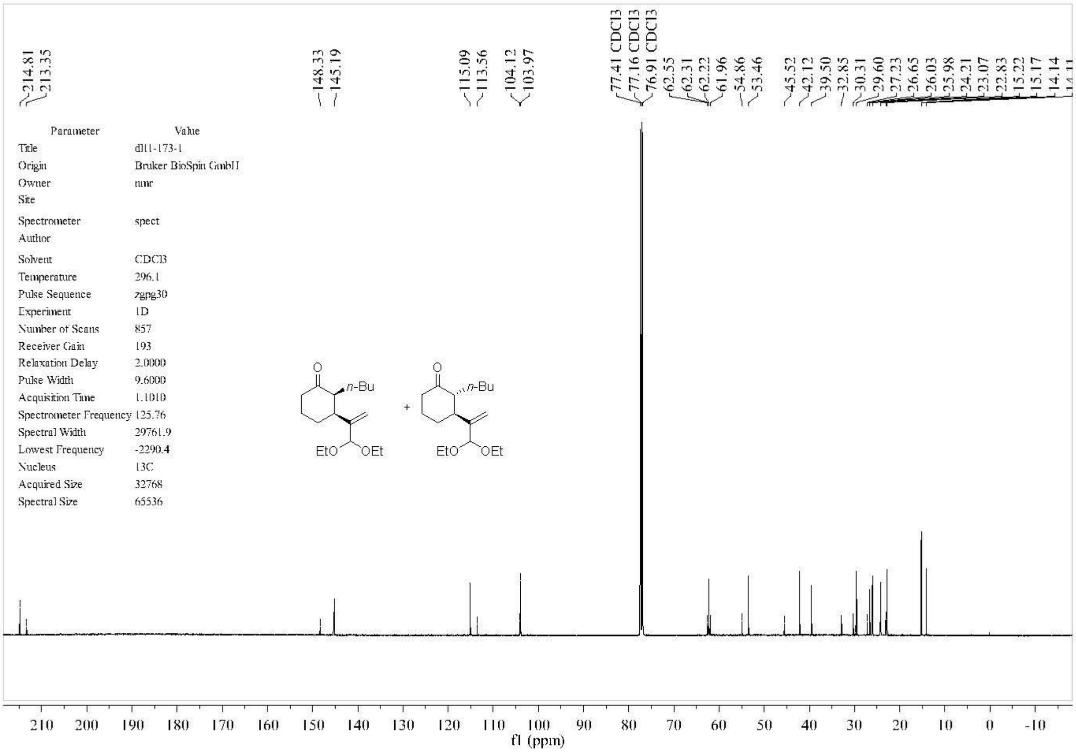

Supplementary Figure 190 ^{13}C NMR of 3ji's deprotection product

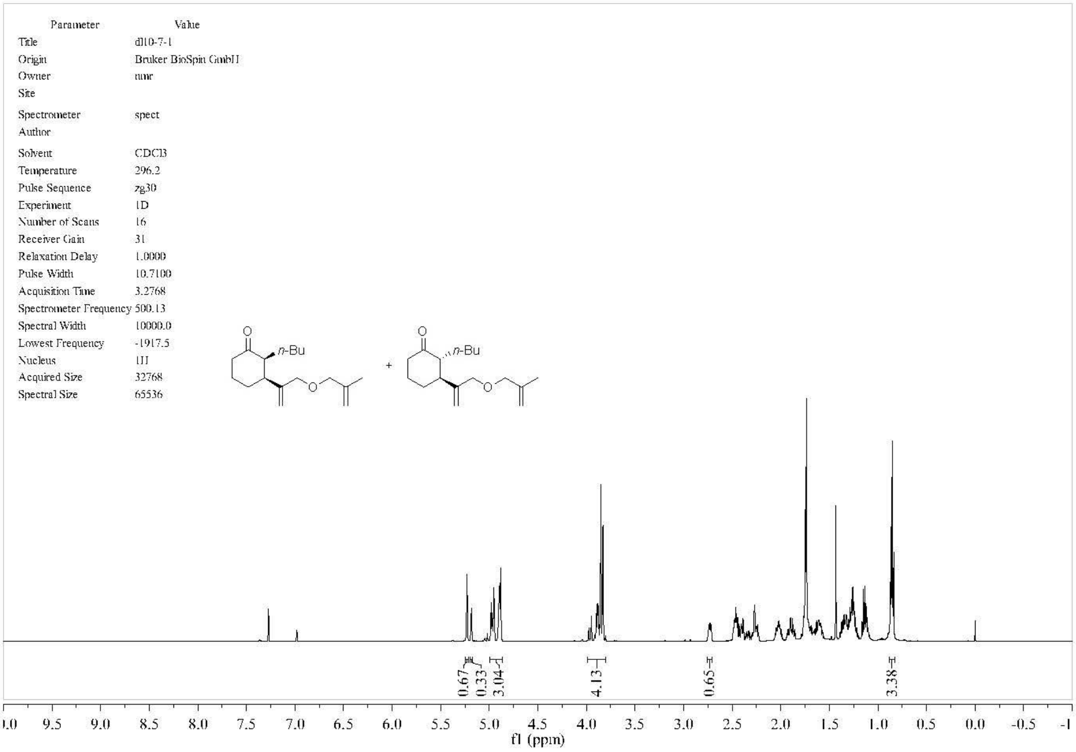

Supplementary Figure 191 ^{1}H NMR of 3jj's deprotection product

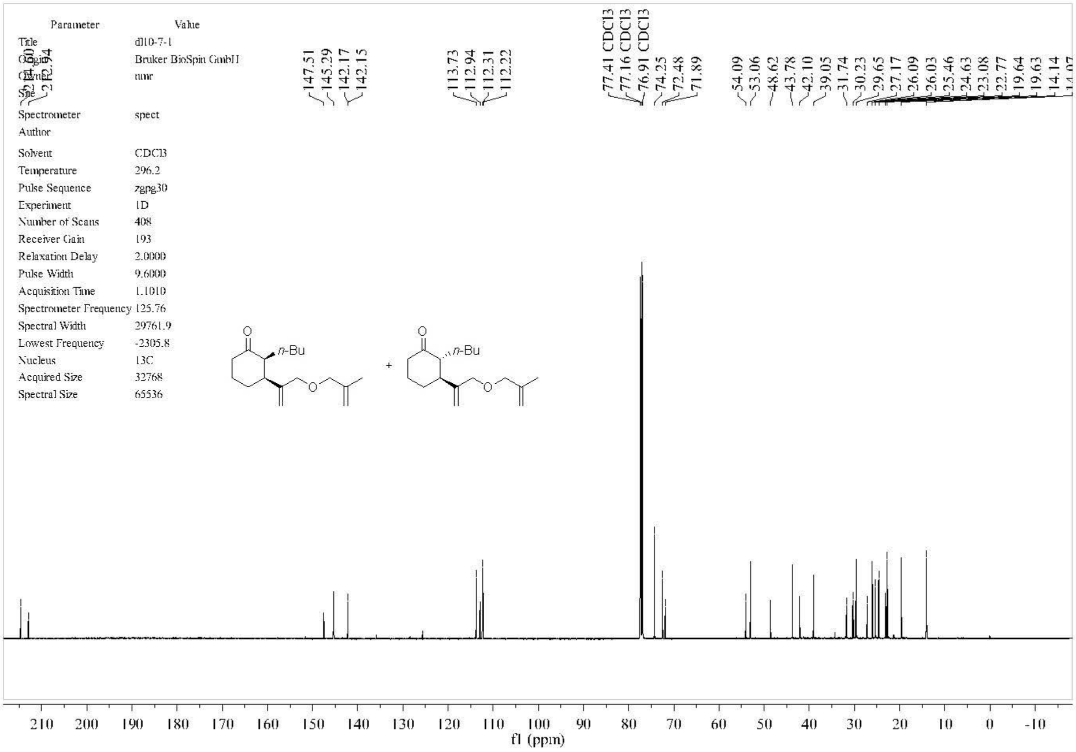

Supplementary Figure 192 ^{13}C NMR of 3jj's deprotection product

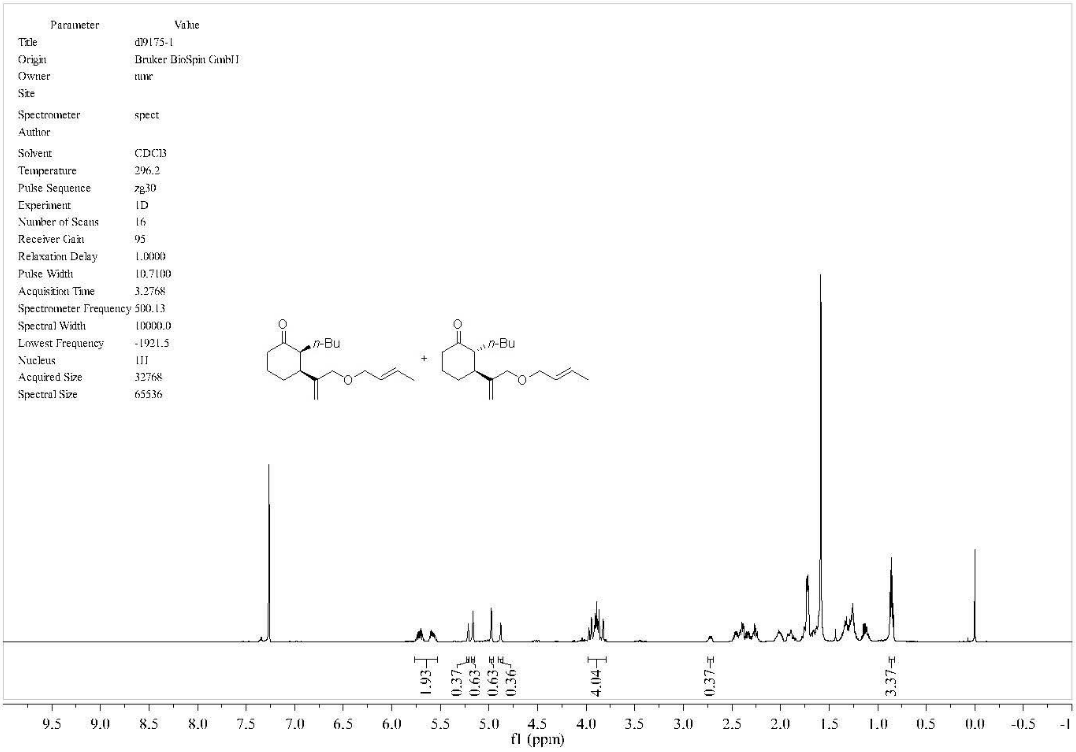

Supplementary Figure 193 ^{1}H NMR of 3jk's deprotection product

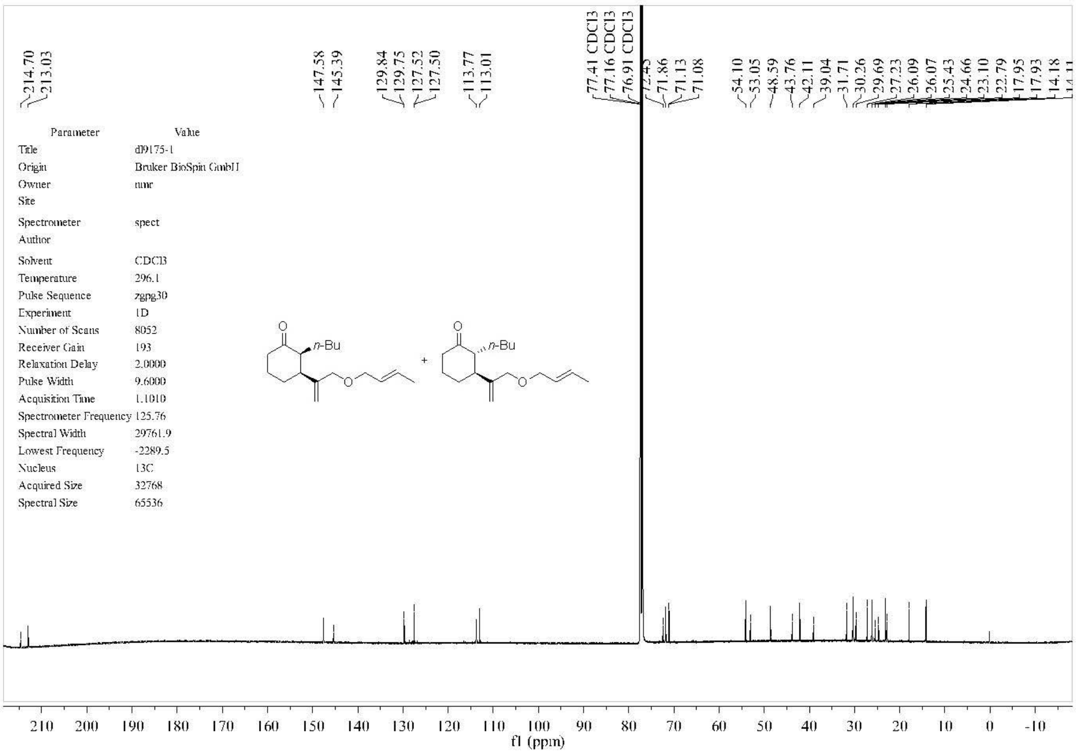

Supplementary Figure 194 ^{13}C NMR of 3jk's deprotection product

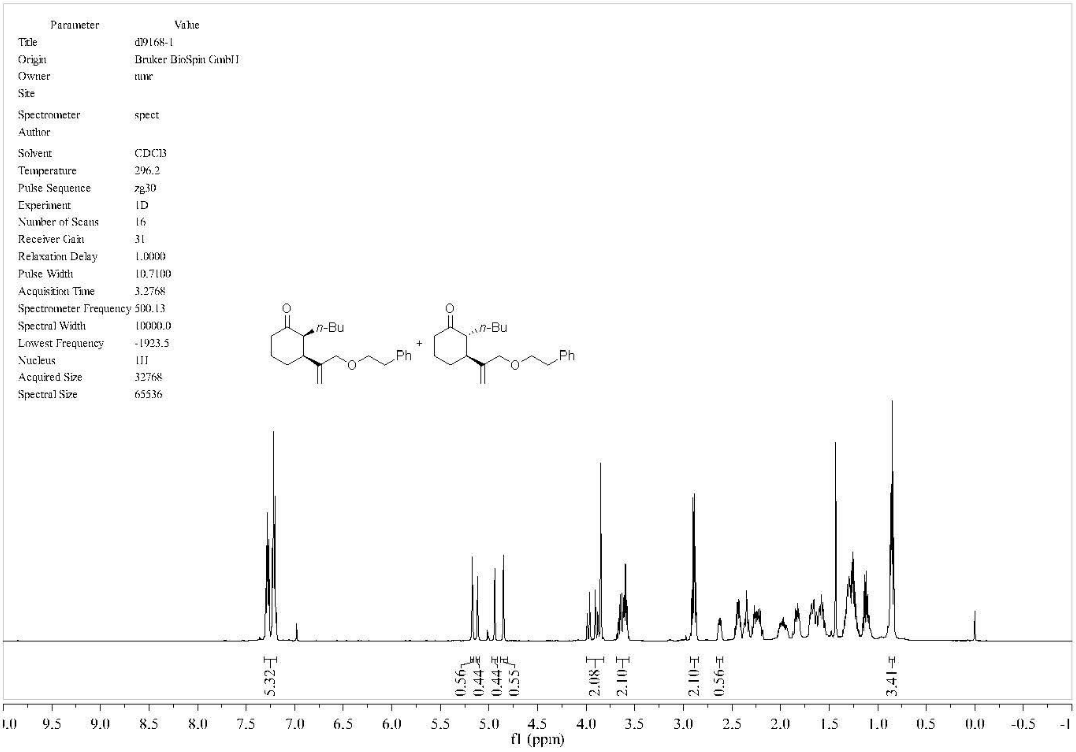

Supplementary Figure 195 ^{1}H NMR of 3jl's deprotection product

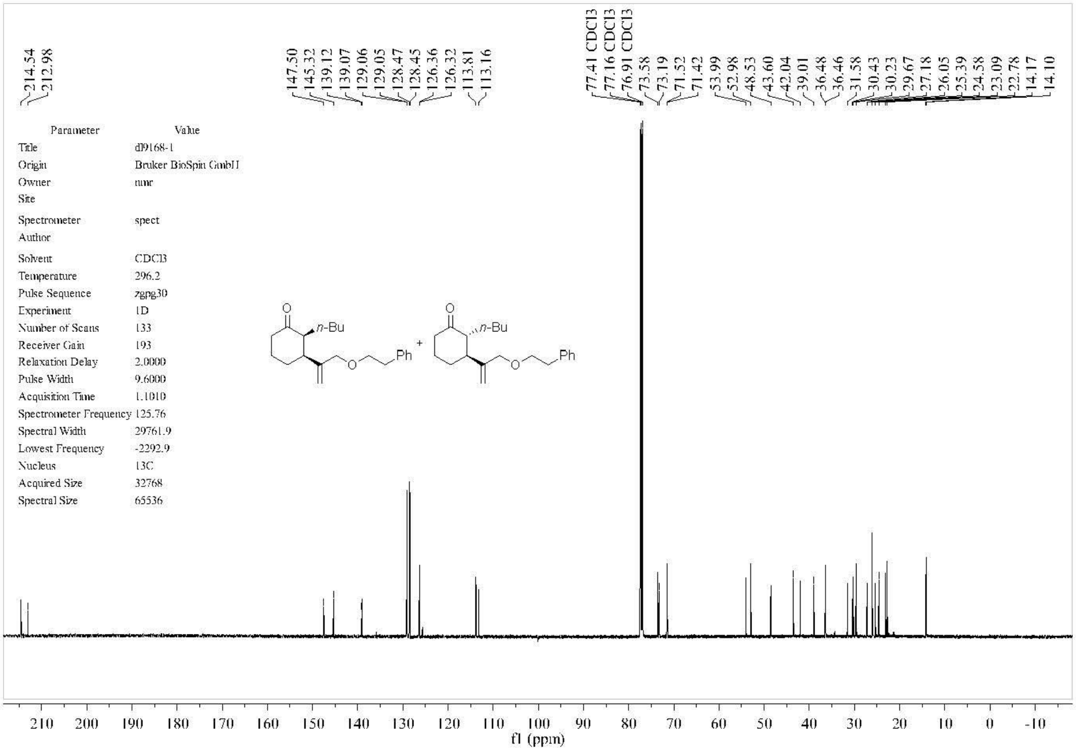

Supplementary Figure 196 ^{13}C NMR of 3jl's deprotection product

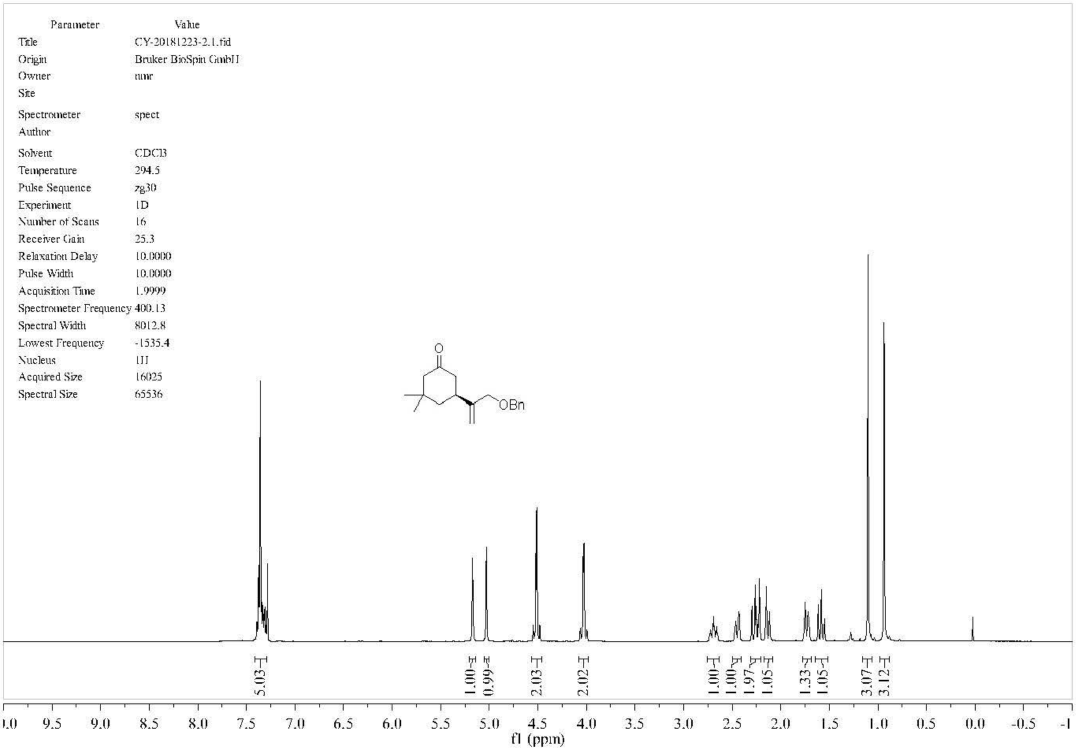

Supplementary Figure 197 ^{1}H NMR of 3qa's deprotection product

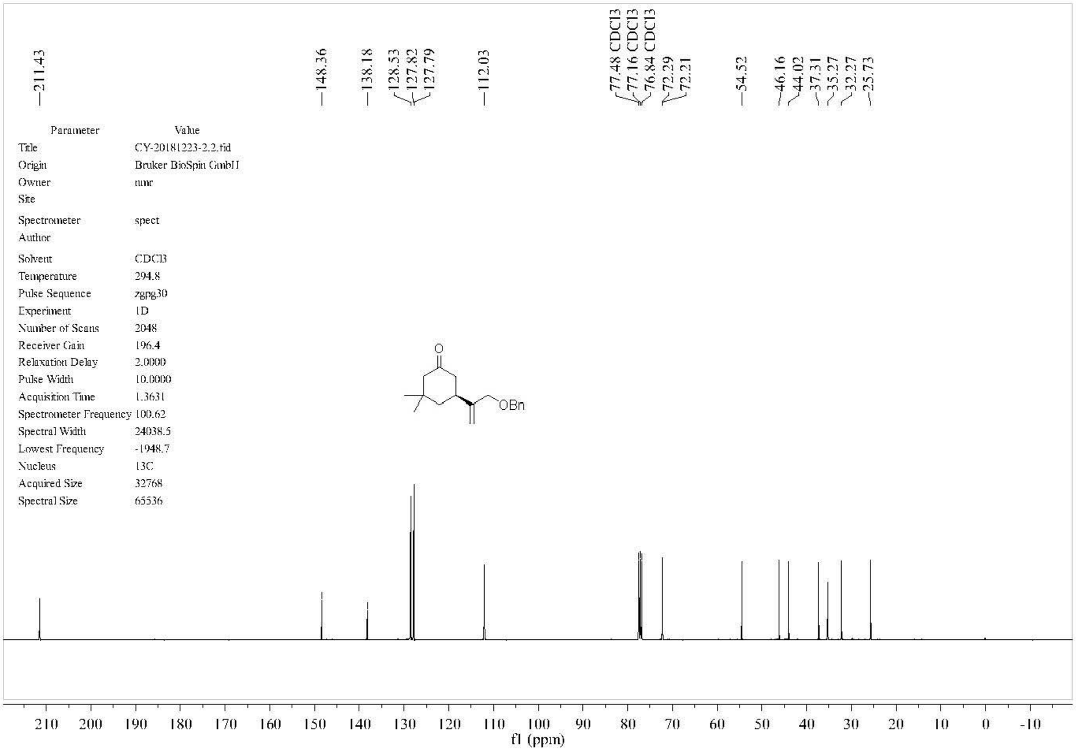

Supplementary Figure 198 ^{13}C NMR of 3qa's deprotection product

Supplementary References

1. Murakami, H., Minami, T. & Ozawa, F. Facile and Selective Deallylation of Allyl Ethers Using Diphosphinidenecyclobutene-Coordinated Palladium Catalysts. *J. Org. Chem.* **69**, 4482-4486 (2004).

2. Yagunov, S.E., Khol'shin, S.V., Kandalintseva, N.V. & Prosenko, A.E. Synthesis and antioxidant activity of 5-hydroxycoumarans, 6-hydroxychromanes and sulfur-containing derivatives on their base. *Russ Chem Bull* **62**, 1395-1400 (2013).

3. Huo, X., *et al.* Hydrogen-Bond-Activated Palladium-Catalyzed Allylic Alkylation via Allylic Alkyl Ethers: Challenging Leaving Groups. *Org. Lett.* **16**, 1570-1573 (2014).

4. Borg, T., Tuzina, P. & Somfai, P. Lewis Acid-Promoted Addition of 1,3-Bis(silyl)propenes to Aldehydes: A Route to 1,3-Dienes. *J. Org. Chem.* **76**, 8070-8075 (2011).

5. Rudolph, J., *et al.* Indanylacetic Acid Derivatives Carrying 4-Thiazolyl-phenoxy Tail Groups, a New Class of Potent PPAR α/γ/δ Pan Agonists: Synthesis, Structure−Activity Relationship, and In Vivo Efficacy. *J. Med. Chem.* **50**, 984-1000 (2007).

6. Schmidt, A.M. & Eilbracht, P. Tandem hydroformylation–hydrazone formation–Fischer indole synthesis: a novel approach to tryptamides. *Org. Biomol. Chem.* **3**, 2333-2343 (2005).

7. Sarkar, N., Banerjee, A. & Nelson, S.G. [4 + 2] Cycloadditions of N-Alkenyl Iminium Ions: Structurally Complex Heterocycles from a Three-Component Diels−Alder Reaction Sequence. *J. Am. Chem. Soc.* **130**, 9222-9223 (2008).

8. Ning, X.-S., Wang, M.-M., Yao, C.-Z., Chen, X.-M. & Kang, Y.-B. tert-Butyl Nitrite: Organic Redox Cocatalyst for Aerobic Aldehyde-Selective Wacker–Tsuji Oxidation. *Org. Lett.* **18**, 2700-2703 (2016).

9. Dible, B.R. & Sigman, M.S. Steric Effects in the Aerobic Oxidation of π-Allylnickel(II) Complexes with N-Heterocyclic Carbenes. *Inorg Chem* **45**, 8430-8441 (2006).

10. Dible, B.R. & Sigman, M.S. Unusual Reactivity of Molecular Oxygen with π-Allylnickel(N-heterocyclic carbene) Chloride Complexes. *J. Am. Chem. Soc.***125**, 872-873 (2003).

11. Ho, C.-Y., Chan, C.-W. & He, L. Catalytic Asymmetric Hydroalkenylation of Vinylarenes: Electronic Effects of Substrates and Chiral N-Heterocyclic Carbene Ligands. *Angew. Chem. Int. Ed.* **54**, 4512-4516 (2015).

12. Lian, X., Chen, W., Dang, L., Li, Y. & Ho, C.-Y. (NHC)NiH-Catalyzed Intermolecular Regio- and Diastereoselective Cross-Hydroalkenylation of Endocyclic Dienes with α-Olefins. *Angew. Chem. Int. Ed.* **56**, 9048-9052 (2017).

13. Chen, W., Li, Y., Chen, Y. & Ho, C.-Y. (NHC)NiH-Catalyzed Regiodivergent Cross-Hydroalkenylation of Vinyl Ethers with α-Olefins: Syntheses of 1,2- and 1,3-Disubstituted Allyl Ethers. *Angew. Chem. Int. Ed.* **57**, 2677-2681 (2018).

14. Huang, J.-Q. & Ho, C.-Y. [(NHC)NiIIH]-Catalyzed Cross-Hydroalkenylation of Cyclopropenes with Alkynes: Cyclopentadiene Synthesis by [(NHC)NiII]-Assisted C−C Rearrangement. *Angew. Chem. Int. Ed.* **58**, 5702-5706 (2019).

15. Chaulagain, M.R., Sormunen, G.J. & Montgomery, J. New N-Heterocyclic Carbene Ligand and Its Application in Asymmetric Nickel-Catalyzed Aldehyde/Alkyne Reductive Couplings. *J. Am. Chem. Soc.* **129**, 9568-9569 (2007).

16. Marques, F.A.*, et al.* Structure Confirmation of a Bioactive Lactone Isolated from Otoba parvifolia through the Synthesis of a Model Compound. *J. Nat. Prod.* **67**, 1939-1941 (2004).

17. Nicolaou, K.C., Ding, H., Richard, J.-A. & Chen, D.Y.K. Total Synthesis of Echinopines A and B. *J. Am. Chem. Soc.* **132**, 3815-3818 (2010).

18. Kotoku, N., Sumii, Y. & Kobayashi, M. Stereoselective Synthesis of Core Structure of Cortistatin A. *Org. Lett.* **13**, 3514-3517 (2011).

19. Gatri, R., Rezgui, F. & Gaïed, M.M.E. Regioselective reaction of a Cyclic Baylis-Hillman Adduct with Alcohols and Thiols. *J. Chem. Res.* **2002**, 366-367 (2002).

20. Tanemura, K., Suzuki, T., Nishida, Y., Satsumabayashi, K. & Horaguchi, T. A mild and efficient procedure for α-bromination of ketones using N-bromosuccinimide catalysed by ammonium acetate. *Chem. Commun.* 470-471 (2004).

21. Hua, D.H.*, et al.* A One-Pot Condensation of Pyrones and Enals. Synthesis of 1H,7H-5a,6,8,9-Tetrahydro-1-oxopyrano[4,3-b][1]benzopyrans. *J. Org. Chem.* **62**, 6888-6896 (1997).

22. Artiom, C., Rune, R. & Baran, P.S. 11-Step Total Synthesis of (−)-Maoecrystal V. *J. Am. Chem. Soc*. **138**, 9425-9428 (2016).

23. Zhang, G.-B.*, et al.* Toward the Total Synthesis of Palhinine A: Expedient Assembly of Multifunctionalized Isotwistane Ring System with Contiguous Quaternary Stereocenters. *Org. Lett.* **14**, 3696-3699 (2012).

24. Ramachary, D.B. & Kishor, M. Organocatalytic Sequential One-Pot Double Cascade Asymmetric Synthesis of Wieland−Miescher Ketone Analogues from a Knoevenagel/Hydrogenation/Robinson Annulation Sequence: Scope and Applications of Organocatalytic Biomimetic Reductions. *J. Org. Chem.* **72**, 5056-5068 (2007).

25. Spiccia, N.D.*, et al.* A Formal Synthesis of (−)-Perhydrohistrionicotoxin Using a Cross Metathesis–Hydrogenation Approach. *J. Org. Chem.* **82**, 8725-8732 (2017).

26. Brenninger, C., Pöthig, A. & Bach, T. Brønsted Acid Catalysis in Visible-Light-Induced [2+2] Photocycloaddition Reactions of Enone Dithianes. *Angew. Chem. Int. Ed.* **56**, 4337-4341 (2017).

27. Johnson, T., Pultar, F., Menke, F. & Lautens, M. Palladium-Catalyzed α-Arylation of Vinylogous Esters for the Synthesis of γ,γ-Disubstituted Cyclohexenones. *Org. Lett.* **18**, 6488-6491 (2016).

28. Feierfeil, J., Grossmann, A. & Magauer, T. Ring Opening of Bicyclo[3.1.0]hexan-2-ones: A Versatile Synthetic Platform for the Construction of Substituted Benzoates. *Angew. Chem. Int. Ed.* **54**, 11835-11838 (2015).

29. Huang, Z.*, et al.* A Total Synthesis of Bifidenone. *J. Org. Chem.* **82**, 4235-4241 (2017).

30. Day, J.I., Singh, K., Trinh, W. & Weaver, J.D. Visible Light Mediated Generation of trans-Arylcyclohexenes and Their Utilization in the Synthesis of Cyclic Bridged Ethers. *J. Am. Chem. Soc.* **140**, 9934-9941 (2018).

31. Yi, C.S., Martinelli, L.C. & Blanton, C.D. Synthesis of N-methyl-1-oxa-5-aza[10]paracyclophane: a conformationally restricted analog of phenoxypropylamines. *J. Org. Chem.* **43**, 405-409 (1978).

32. Johnson, C.R. & Kozak, J. Chemoenzymic Synthesis of 3-Deoxy-D-arabino-heptulosonic Acid from Cycloheptatriene. *J. Org. Chem.* **59**, 2910-2912 (1994).

33. Baldwin, J.E., Adlington, R.M. & Robertson, J. Carbocyclic ring expansion reactiuon VIA radical chain processes. *Tetrahedron* **45**, 909-922 (1989).

34. Liu, X., Chen, X. & Mohr, J.T. Copper-Catalyzed γ-Sulfonylation of α,β-Unsaturated Carbonyl Compounds by Means of Silyl Dienol Ethers. *Org. Lett.* **17**, 3572-3575 (2015).

35. Romanski, S.*, et al.* Acyloxybutadiene tricarbonyl iron complexes as enzyme-triggered CO-releasing molecules (ET-CORMs): a structure–activity relationship study. *Dalton Trans.* **41**, 13862-13875 (2012).

36. Fleming, I., Goldhill, J. & Paterson, I. γ-Sulphenylation of αβ-unsaturated aldehydes, ketones, and esters: the use of O-silylated dienolates. *Tetrahedron Lett.* **20**, 3205-3208 (1979).

37. Ashenhurst, J.A., Isakovic, L. & Gleason, J.L. Application of a [6+4] cycloaddition strategy toward the total synthesis of CP-225,917. *Tetrahedron* **66**, 368-378 (2010).

38. Cahiez, G., Habiak, V. & Gager, O. Efficient Preparation of Terminal Conjugated Dienes by Coupling of Dienol Phosphates with Grignard Reagents under Iron Catalysis. *Org. Lett.* **10**, 2389-2392 (2008).

39. Renom-Carrasco, M.*, et al.* Asymmetric Hydrogenation of 3-Substituted Pyridinium Salts. *Chem. Eur. J.* **22**, 9528-9532 (2016).

40. Krow, G.R., Huang, Q., Szczepanski, S.W., Hausheer, F.H. & Carroll, P.J. Stereoselectivity in Diels−Alder Reactions of Diene-Substituted N-Alkoxycarbonyl-1,2-dihydropyridines. *The J. Org. Chem.* **72**, 3458-3466 (2007).

41. Henderson, K.W., Kerr, W.J. & Moir, J.H. Magnesium amide base-mediated enantioselective deprotonation processes. *Tetrahedron* **58**, 4573-4587 (2002).

42. Brémond, P., Vanthuyne, N. & Audran, G. Synthesis of (+)-striatene: confirmation of its stereostructure. *Tetrahedron Lett.* **50**, 5723-5725 (2009).

43. Knopff, O. & Alexakis, A. Tandem Asymmetric Conjugate Addition−Silylation of Enantiomerically Enriched Zinc Enolates. Synthetic Importance and Mechanistic Implications. *Org. Lett.* **4**, 3835-3837 (2002).

44. Larionov, O.V. & Corey, E.J. Ni(II)-Catalyzed Enantioselective Conjugate Addition of Acetylenes to α,β-Enones. *Org. Lett.* **12**, 300-302 (2010).

45. Paquette, L.A., Wang, H.-L., Su, Z. & Zhao, M. Enantiospecific Total Synthesis of Natural (+)-Taxusin. 2. Functionalization of the A-Ring and Arrival at the Target. *J. Am. Chem. Soc*. **120**, 5213-5225 (1998).

46. Aoyama, H.*, et al.* Hydrolysis of Alkenyl Esters and Ethers Catalyzed by Metal Complexes. *Org. Lett.* **6**, 509-512 (2004).
